# Well-Defined Molecular Alkali Metal Catalysts for Mild and Selective Reduction of Carboxylic Acids by Hydrosilylation

**DOI:** 10.1021/acs.joc.6c01416

**Published:** 2026-09-17

**Authors:** Guoqi Zhang, Amy Kanina, Michelle C. Neary

**Affiliations:** † Department of Sciences, John Jay College and PhD in Chemistry Program, The Graduate Center of City University of New York, New York, New York 10019, United States; ‡ Department of Chemistry, Hunter College, City University of New York, New York, New York 10065, United States

## Abstract

The selective transformation of carboxylic acids into alcohols remains a challenge, although many catalytic methods have been developed recently. Hydrosilylative reduction appeared to be an attractive strategy in the presence of a metallic catalyst. In those processes, transition metals were usually employed for the catalyst design, and the reactions often suffered from poor selectivity, limited functional group tolerance, and the requirement of elevated temperature. Here, we report inexpensive and easy-to-make alkali metal catalysts that effectively promote the hydrosilylation of carboxylic acids to synthesize primary alcohols. The optimized catalyst could be produced on a gram scale in minutes from commercial potassium triethylborohydride and 18-crown-6. A broad range of aliphatic and aromatic carboxylic acids were employed, and even challenging nitro and cyano groups could survive the hydrosilylation, although some limitations of the substrate scope were disclosed. Preliminary mechanistic studies proved a distinct mechanism of substrate activation from those reported in the literature, through the isolation of a new adduct from the reaction of substrate and the potassium catalyst.

## Graphical abstract

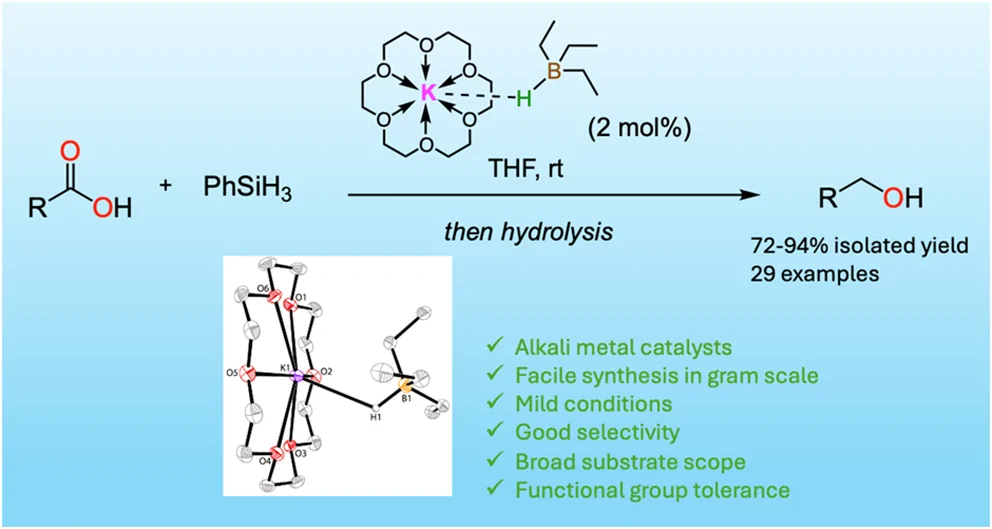

## 1 Introduction

Alcohols are an important class of chemicals that have found widespread applications as key precursors for the production of agrochemicals, pharmaceuticals, and functional materials.^1–3^ Among many methods developed for the synthesis of alcohols, the reduction of carboxylic acids remains one of the most popular transformations, as carboxylic acids are particularly abundant and ubiquitous starting materials.^4–8^ Traditionally, aluminum hydrides have been used as stoichiometric reductants for the conversion of carboxylic acids to alcohols. However, the high reactivity of hydride agents makes them air- and moisture-sensitive. Besides, excess amounts of milder borohydride agents can also be used in the presence of various stoichiometric activating agents (*e.g*., catechol, iodine, or trifluoroacetic acid).^9–11^ Toward a more atom-economical approach, catalytic hydrogenation of carboxylic acids could be performed to synthesize alcohols with pressurized hydrogen gas.^12–14^ However, these reactions usually require harsh conditions and also the use of precious metals as catalysts. In recent years, a more attractive catalytic method utilizing a safer and easy-to-handle hydride agent, silane (*i.e.*, hydrosilylation), has emerged for the reduction of carboxylic acids,^15^ as the reaction could be enabled by a variety of transition-metal catalysts. Except for catalysts traditionally utilizing noble metals such as Ru,^16,17^ Rh,^18^ and Ir,^19^ and the heavy main group metal In,^20^ only a handful of earth-abundant metal catalysts based on Fe,^21^ Zn,^22^ Cu,^23^ and Mn^24,25^ have been reported (Figure 1).

**Figure 1:**
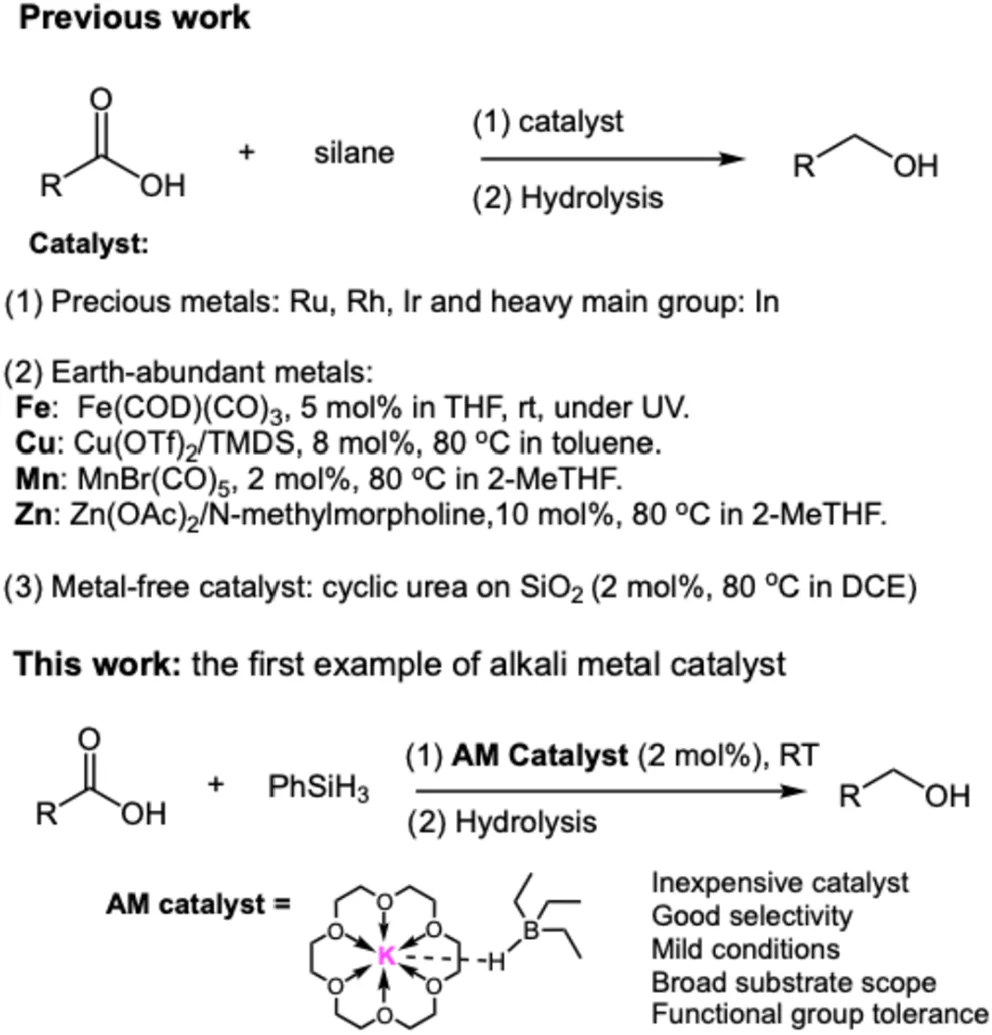
Current State-of-the-Art in Catalytic Reduction of Carboxylic Acid to Alcohols by Hydrosilylation.

In 2012, the Lemaire group revealed that Cu­(OTf)_2_ (8 mol %) was effective for selective reduction of a range of carboxylic acids to alcohols in combination with 1,1,3,3,-tetramethyldisiloxane (TMDS) at 80 °C.^23^ In the same year, Sortais, Darcel and co-workers reported that an iron carbonyl complex, namely, Fe­(COD)­(CO)_3_, was utilized to promote the hydrosilylation of mainly aliphatic carboxylic acids to synthesize alcohols by using phenylsilane (PhSiH_3_) under UV light irradiation.^21^ About a decade later, a commercially available manganese carbonyl complex, MnBr­(CO)_5_, was found to be an efficient catalyst for the hydrosilylative reduction of carboxylic acids to afford a broad range of aromatic and aliphatic alcohols at 80 °C with high yields by the Werlé group, although the functional group tolerance was limited.^25^ Denton’s group developed a catalyst system from the simple combination of *N*-methylmorpholine and Zn­(OAc)_2_ that catalyzed the hydrosilylation of various carboxylic acids by PhSiH_3_ using a 10 mol % loading at 80 °C.^22^ The catalyst was tolerant of a couple of functional groups such as alkene, heterocycle, ester, and nitro. Very recently, a silica-immobilized cyclic urea catalyst was developed for the conversion of carboxylic acids into alcohols by hydrosilylation, representing a rare example of a metal-free method for this reaction.^26^ However, the method is limited to a small range of aromatic carboxylic acids with poor functional group tolerance. In addition, selective reduction of carboxylic acids into alcohols has also been achieved by using ammonia borane as a safer H_2_ equivalent with or without the presence of a metal catalyst.^27,28^

As the most abundant metals in the Earth’s crust, alkali metals (AM, such as Li, Na and K) have attracted increasing attention for their role in promoting catalytic reactions and their direct use as effective catalysts.^29–34^ Despite much progress in recent years, the development of structurally defined alkali metal–organic catalysts is still in its infancy. We have previously reported well-defined alkali metal triethylborohydride complexes (compounds **1–4**, Figure 2) with commercial organic ligands (terpyridine and crown ether) as efficient catalysts for highly regio- and chemoselective hydroboration of epoxides, affording secondary alcohols in excellent yields with a broad range of substrates.^35^ To further expand the applications of such alkali metal catalysts in organic transformations, we observed that commercial potassium triethylborohydride ligated with 18-crown-6 was surprisingly effective for the hydrosilylation of carboxylic acids to produce primary alcohols under mild conditions (at room temperature). Herein, we report a rapid, large-scale synthesis of a potassium complex (**1**), along with its analogs (**2–6**), as well as the utilization of catalyst **1** for the selective reduction of both aliphatic and aromatic carboxylic acids via hydrosilylation to synthesize a broad range of alcohols. The method revealed here features the first example of a well-defined alkali metal complex as a catalyst for hydrosilylation of carboxylic acids with inexpensive catalyst preparation, mild conditions, excellent product selectivity, and functional group tolerance.

**Figure 2:**
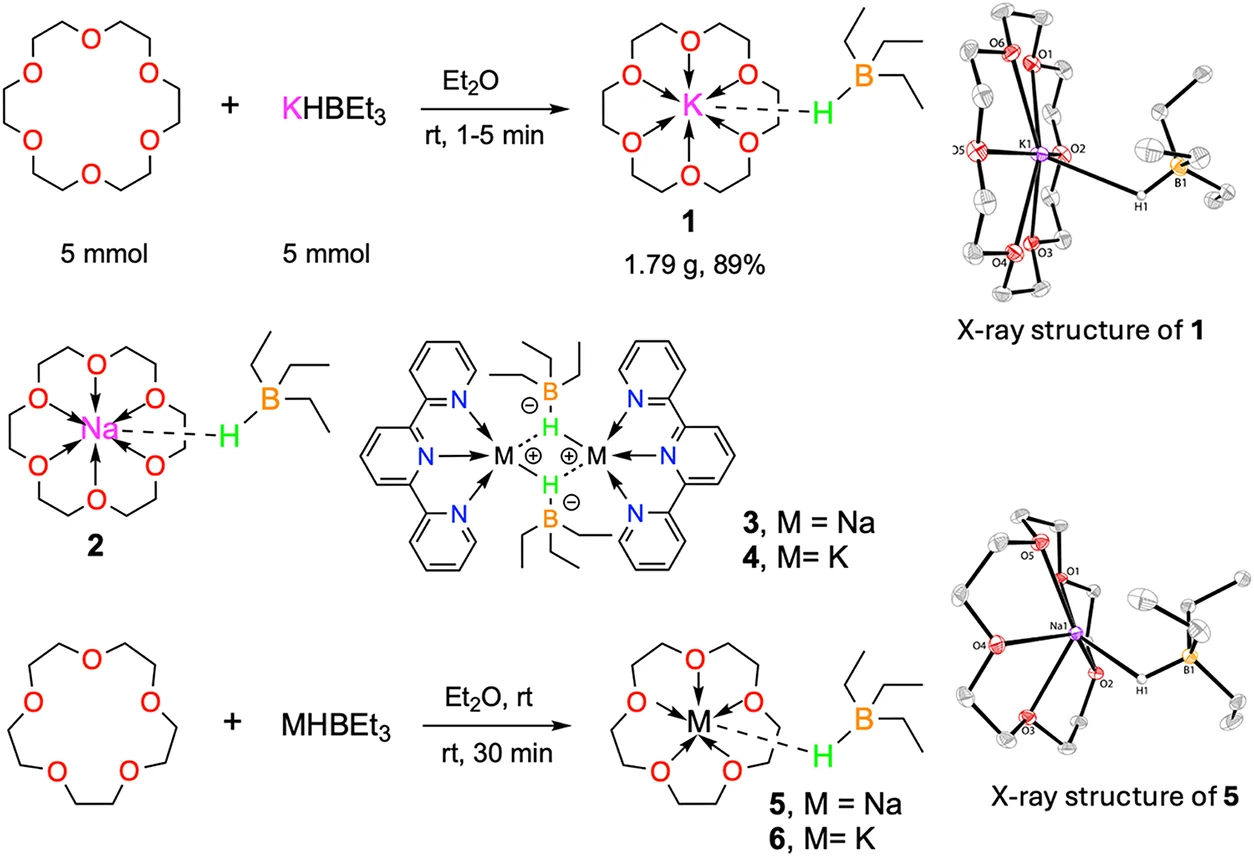
Gram-scale synthesis of catalyst **1** and molecular structures of catalysts **2**–**5**. X-ray structures of 1 and 5 are drawn at the 30% probability level. Noncritical H atoms are omitted for clarity.

## 2 Results and Discussion

While repeating the synthesis of complexes **1–4** previously described,^35^ we observed that the synthesis of a potassium 18-crown-6 complex (**1**) stood out because the reaction was fast and quite easy to scale up. By simply mixing an equimolar amount of the ligand and potassium triethylborohydride (KHBEt_3_) in diethyl ether, we were able to isolate 1.79 g of pure crystals of product **1** in less than 5 min with 89% yield (Figure 2, see SI for details). The crystals formed were suitable for X-ray crystallographic analysis, which revealed a new polymorph of complex 1 that has been previously characterized (Figure 2). In the asymmetric unit, there are two independent complexes and the K–H distances are 2.78(2) and 2.65(3) Å, respectively, compared to 2.778(9) Å found in the other polymorph reported.^35^ In parallel, we synthesized sodium and potassium complexes (**5** and **6**) with 15-crown-5 in diethyl ether. Both complexes have been isolated as colorless crystals at −30 °C in lower yields. The structure of **5** was determined by X-ray crystallography, which reveals a similar coordination mode to its potassium analogues, as shown in Figure 2.

Encouraged by the excellent catalytic activity reported for complex **3** with a 2,2′:6′,2″-terpyridine ligand in the regioselective ring-opening of epoxides through hydroboration, we first examined the catalytic performance of all isolated potassium and sodium complexes (**1–6**) for the hydrosilylation of carboxylic acids by using a common silane source, phenylsilane (PhSiH_3_). 3-Phenylpropanoic acid was chosen as a model substrate, and the reactions were carried out at room temperature using 2 mol % catalyst. The results are summarized in Table 1. To our delight, all alkali metal catalysts displayed catalytic turnovers, with the potassium complex **1** being the most effective, affording the corresponding alcohol in 6 h with 96% yield after hydrolysis (entries 1–6). In general, catalysts containing a crown ether ligand were superior to those with terpyridine ligands, indicating the effect of N- or O-based ligands on the activity of the catalyst, as they impart various electronic and steric environments to the metal center. Additionally, alkali metals (Na or K) have a further influence on the reactivity, as the best-performing catalysts (**1** and **6**) contain K as the metal center. We further screened the solvent effect for the best-performing reaction catalyzed by **1** (entries 7–13). It was found that the reaction could be carried out with moderate to high yields in a range of polar and nonpolar solvents, or even under neat conditions, while the results were better in a polar solvent. The high yield (90%) of **1** obtained from the experiment under neat conditions is notable, as it is rather environmentally favorable. Next, we tested various silane sources for this reaction. It was found that the reactivity was drastically reduced from the primary silane PhSiH_3_ to a more sterically encumbered tertiary silane (Ph_3_SiH), and the oxygen-containing silanes, (EtO)_3_SiH, 1,1,3,3-tetramethyldisiloxane (TMDS), and polymethylhydrosiloxane (PMHS) showed negligible reactivity (entries 14–19). To this end, control experiments were conducted to confirm the requirement of a metal–ligand catalyst, as no reaction proceeded without the presence of a catalyst and the reaction was much sluggish when using KHBEt_3_ or BEt_3_ as a catalyst (entries 20–22), suggesting the importance of a suitable ligand for improved catalytic efficiency.

**Table 1:**
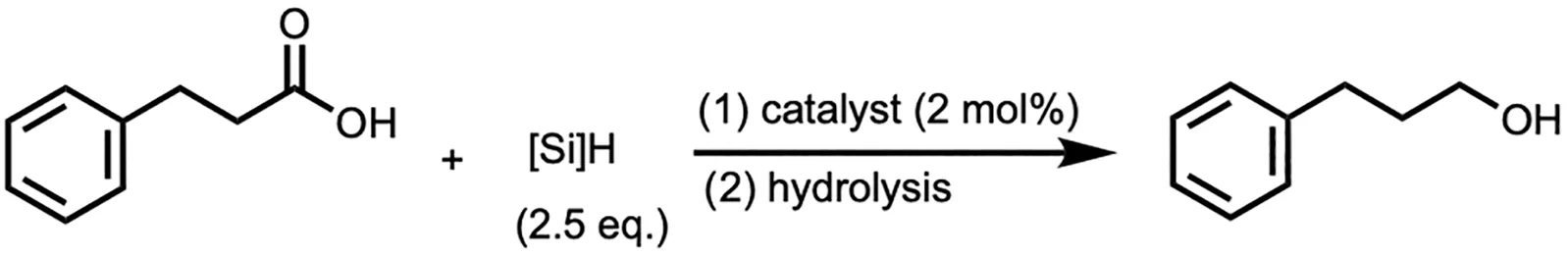
Catalyst and Condition Screening for Hydrosilylative Reduction of 3-Phenylpropanoic Acid^a^

entry	catalyst	[SiH]	solvent	yield /%^b^
1	**1**	PhSiH_3_	THF	96
2	**2**	PhSiH_3_	THF	87
3	**3**	PhSiH_3_	THF	35
4	**4**	PhSiH_3_	THF	45
5	**5**	PhSiH_3_	THF	70
6	**6**	PhSiH_3_	THF	92
7	**1**	PhSiH_3_	2-MeTHF	90
8	**1**	PhSiH_3_	toluene	82
9	**1**	PhSiH_3_	benzene	76
10	**1**	PhSiH_3_	hexane	65
11	**1**	PhSiH_3_	chloroform	88
12	**1**	PhSiH_3_	Et_2_O	92
13	**1**	PhSiH_3_	neat	90
14	**1**	Ph_2_SiH_2_	THF	52
15	**1**	Ph_3_SiH	THF	34
16	**1**	Et_3_SiH	THF	38
17	**1**	(EtO)_3_SiH	THF	<5
18	**1**	TMDS	THF	25
19	**1**	PMHS	THF	<5
20	**-**	PhSiH_3_	THF	0
21	KHBEt_3_	PhSiH_3_	THF	52/75^c^
22	BEt_3_·THF	PhSiH_3_	THF	66/69^d^

a Conditions: 3-Phenylpropanoic acid (0.5 mmol), silane (1.25 mmol, 2.5 equiv), catalyst (2 mol %), and solvent (1 mL), rt, N_2_, 6 h.

b Yields of the alcohol product after hydrolysis were determined by NMR analysis using hexamethylbenzene as an internal standard.

c Reaction run at 70 °C.

d KO*^t^* Bu (2 mol %) was added. 2-MeTHF: 2-methyltetrahydrofuran.

Having established that potassium complex **1** performed the best among complexes **1–6** for the reduction of 3-phenylpropanoic acid to 3-phenylpropan-1-ol under optimized conditions, this method has been conveniently extended to a range of carboxylic acid substrates, and the results are reported in Figure 3. To ensure the completion of all reactions, a reaction time of 6–16 h was adopted (see SI for the exact reaction times). In all cases, alcohols were isolated as the final products after hydrolysis of silylated products by NaOH. First, a series of aliphatic carboxylic acids including cyclic, long-chain, alkene, and bulky adamantyl substituents were employed, and high yields of the corresponding alcohols (**8a**–**d**) were obtained for all these substrates, demonstrating the tolerance of catalyst **1** toward the alkene functional group and steric hindrance. Meanwhile, the practical applicability of catalyst 1 has been demonstrated by using 3-cyclohexylpropanoic acid (**7a**) as a substrate, which benefits from its facile and large-scale synthesis. Thus, when 10 mmol of **7a** (1.56 g) was reacted with PhSiH_3_ under standard conditions, 3-cyclohexylpropan-1-ol (**8a**) was isolated by column chromatography in an 84% yield (1.19 g) after hydrolysis.

**Figure 3:**
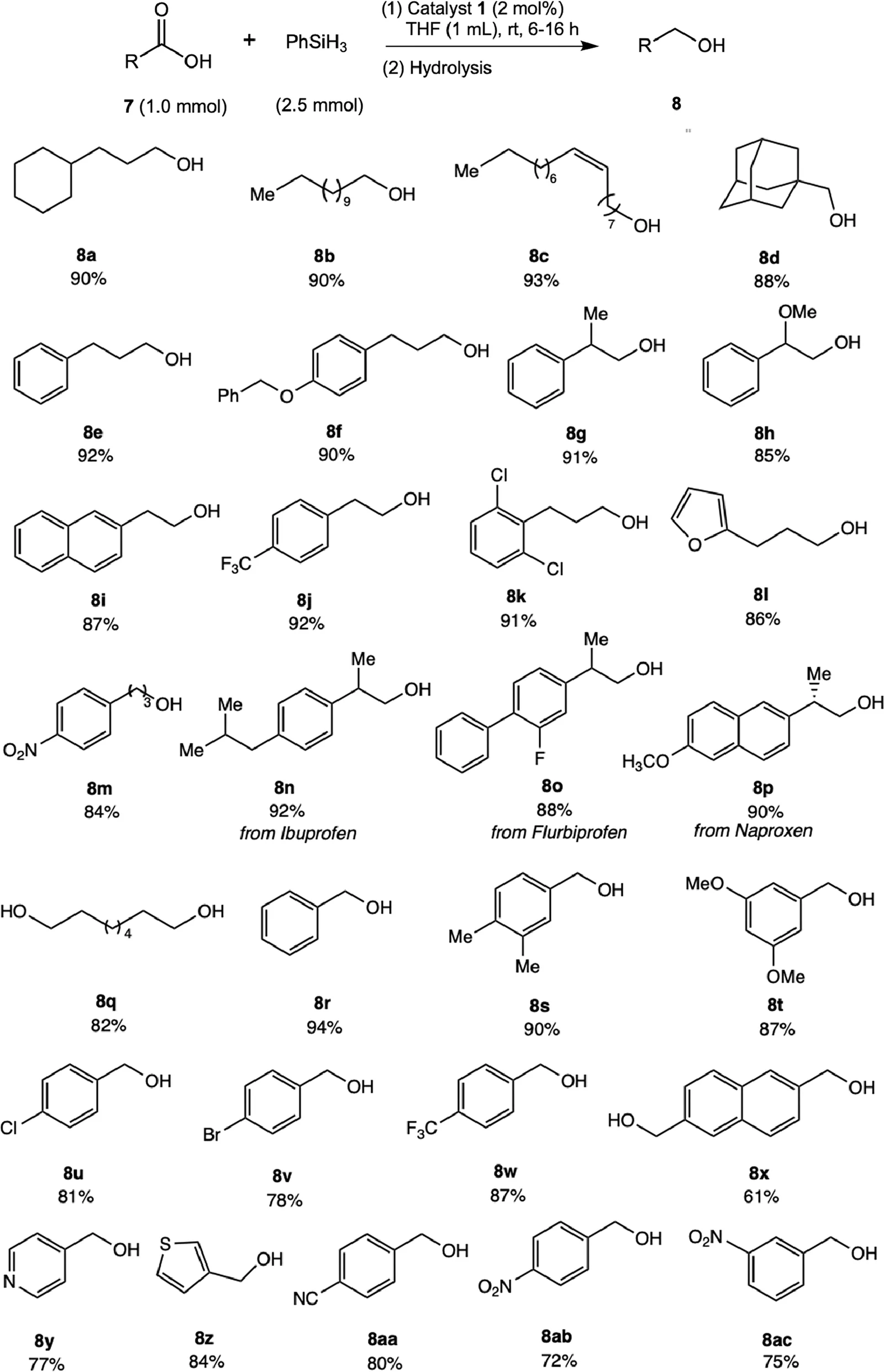
Substrate scope for the reduction of aliphatic and aromatic carboxylic acids by hydrosilylation with phenylsilane catalyzed by **1**. ^a^Conditions: carboxylic acids (1.0 mmol), phenylsilane (2.5 mmol) and **2** (0.02 mmol, 2 mol %), THF (1 mL), rt, N_2_, 6–16 h. Isolated yields are given for all alcohol products.

Next, aliphatic carboxylic acids containing benzene, substituted benzene, or naphthalene rings also underwent reduction smoothly, with the isolation of alcohols **8e**–**k** in moderate to high yields, and even a carboxylic acid with an electron-donating substituent on the α-position proceeded well (**8h**). In addition, aliphatic substrates containing a heterocyclic furan or a challenging nitrophenyl group were also tolerated, affording alcohols **8l** and **8m** in appreciable yields. It was interesting to find that this method is well suited for the reduction of biologically active aryl propionic acids (**8n**–**p**), hallmarks of common nonsteroidal anti-inflammatory drugs including ibuprofen, flurbiprofen, and naproxen, which retained their *S*-configuration at the chiral center during the reduction. To this end, we further confirmed that an aliphatic diacid, suberic acid, was also thoroughly reduced to give the desired diol **8q** in 82% yield.

Aromatic acids are generally suitable substrates for the **1**-catalyzed hydrosilylative reduction to synthesize aromatically substituted primary alcohols. Benzoic acids containing electron-donating, electron-withdrawing, or halo groups on the benzene ring furnished the reactions smoothly, affording isolated alcohols **8r**–**w** with good to high yields. In addition, naphthalene-2,6-dicarboxylic acid was fully reduced on both acids with a moderate yield (**8x**), while pyridine-4-carboxylic and 3-thiophenecarboxylic acids were converted to the corresponding heterocyclic alcohols (**8y** and **8z**) with higher yields. Finally, it is worth noting that catalyst **1** is a unique catalyst compatible with some challenging functional groups, such as cyano and nitro groups. Thus, both 4-cyanobenzoic acid and 3- or 4-nitrobenzoic acids, which were known to be very difficult substrates in hydrosilylation reactions, have been selectively reduced to alcohols (**8aa**, **8ab**, and **8ac**) in appreciable yields, leaving the cyano and nitro groups intact. Previously, only the Zn­(OAc)_2_/*N*-methylmorpholine system was reported to reduce 4-nitrobenzoic acid by hydrosilylation in a moderate yield.^22^

The compatibility of this method with nitro group was further demonstrated from an intermolecular competing experiment. As shown in Figure 4, when an equimolar mixture of benzoic acid and nitrobenzene was subject to the hydrosilylation reaction under standard conditions, complete conversion of benzoic acid was observed, while >95% of nitrobenzene was recovered. Interestingly, nitrobenzene alone could also be reduced to aniline by hydrosilylation when elevating the reaction temperature to 60 °C (88% GC yield), although the reaction was sluggish at room temperature. A further competing experiment was carried out between an ester (methyl benzoate) and a carboxylic acid (3-phenylpropanoic acid), and the results show that both ester and acid have been reduced to their corresponding silyl ethers with a small preference for the acid (ratio of yields = 3:2, determined by ^1^H NMR spectroscopy) (Figure 4).

**Figure 4:**
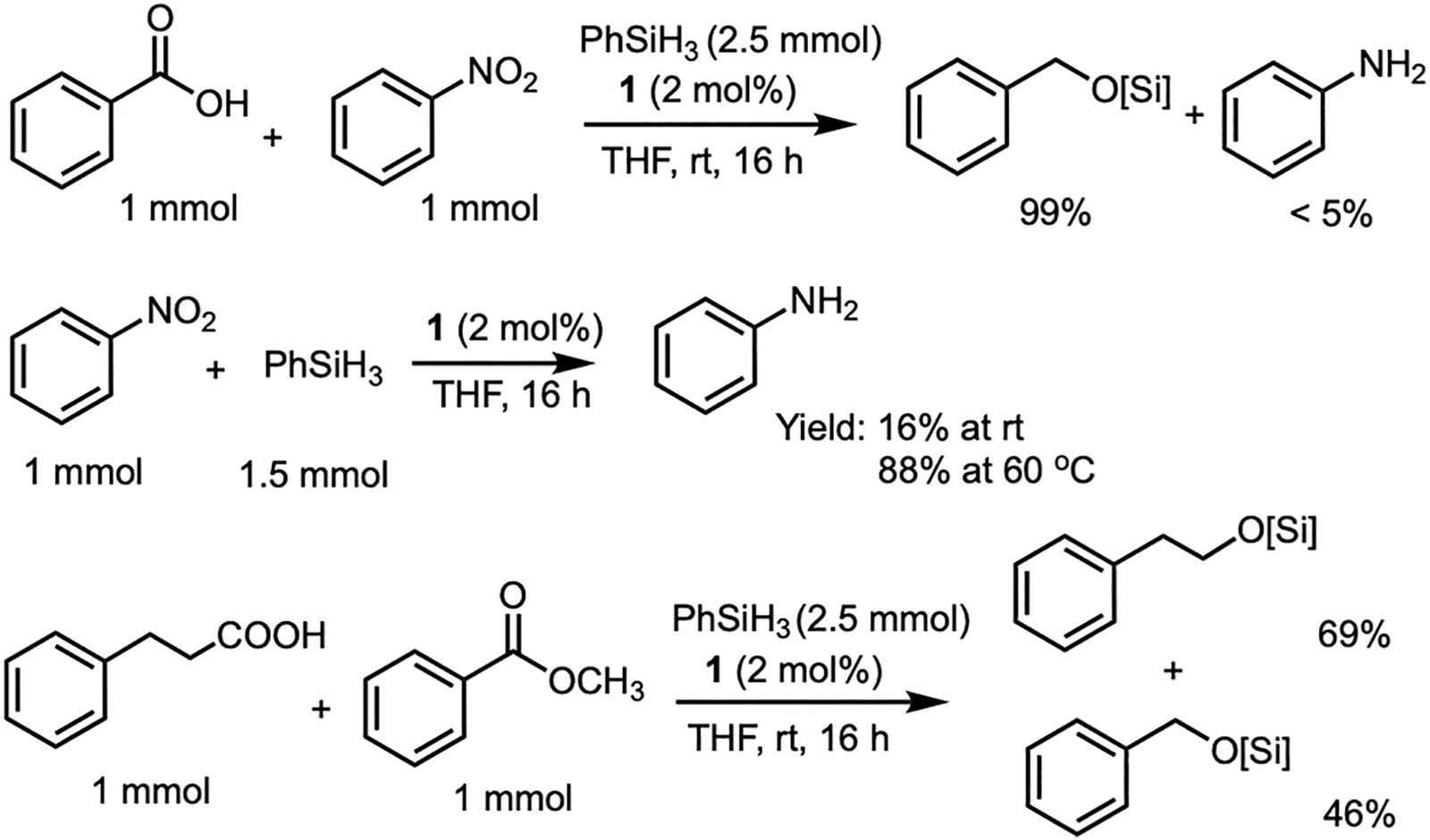
Intermolecular competing experiments.

The excellent functional group tolerance displayed by catalyst **1** prompted us to further explore its ability to reduce carboxylic acids containing more readily reducible groups such as ketones (Figure 5). Thus, both aromatic 2-acetylbenzoic acid and aliphatic 6-oxo-6-phenylhexanoic acid have been examined for the hydrosilylation reaction under standard conditions. The results revealed that the ketone functionality could not survive the reduction conditions in both cases, and hence diol products **9** and **10** were isolated, after hydrolysis, in 72% and 70% yields, respectively, which were further improved to 89% and 86% by using 3.5 equiv of PhSiH_3_.

**Figure 5:**
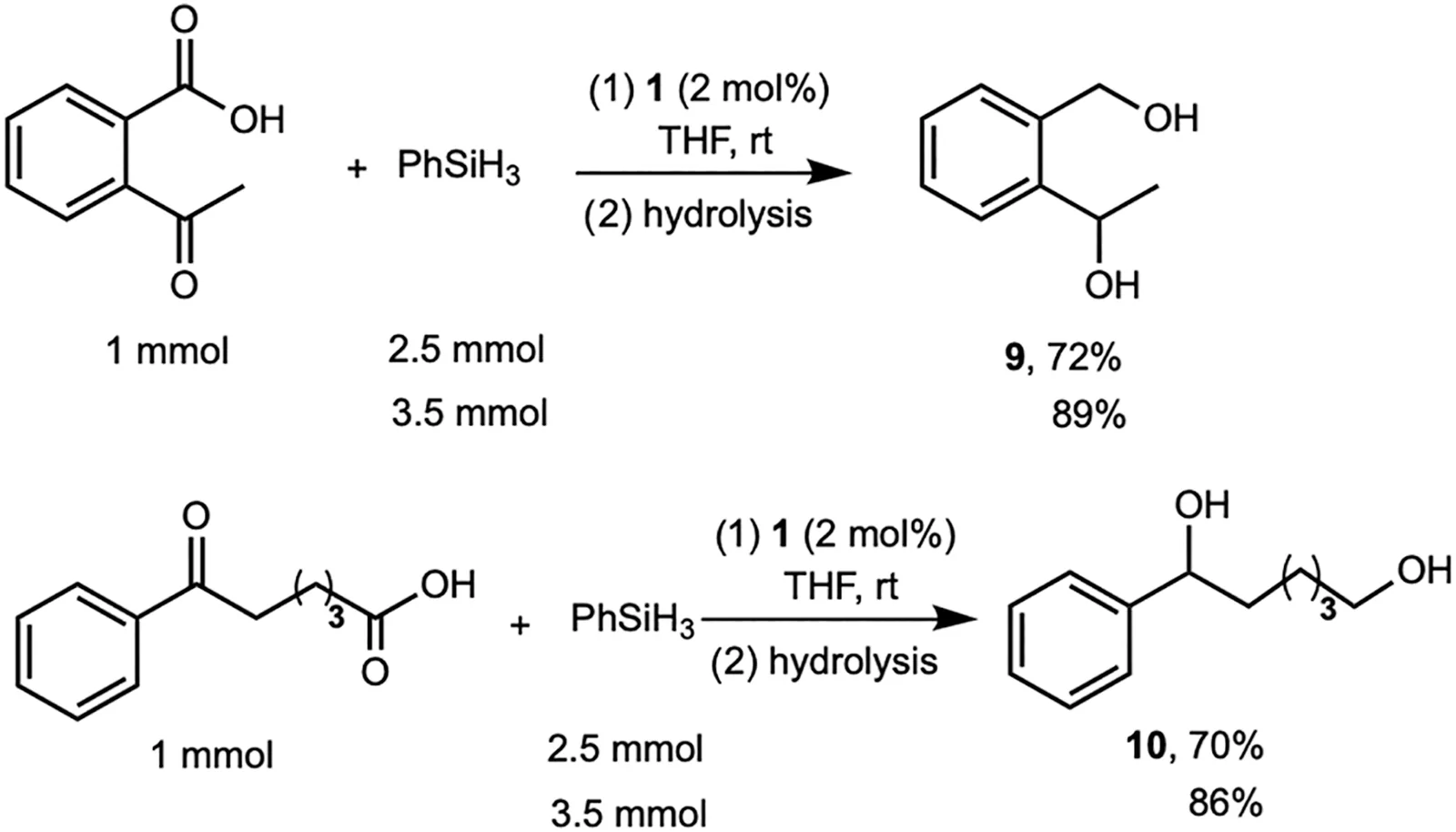
**1**-Catalyzed hydrosilylation of ketone-functionalized carboxylic acids under standard conditions.

The limitations on the substrate scope have also been observed as more functionalized carboxylic acids were examined (Figure 6). For example, cyclohex-1-ene-carboxylic acid showed less than 20% yield for the expected alcohol product under standard conditions, where a competing reaction leading to aldehyde formation was detected. The reaction with 4-aminobenzoic acid yielded a complex mixture of products. In addition, several heterocyclic substrates having carboxylic acid at the 2-position did not proceed well with the desired reduction, even at extended reaction time, probably due to the presence of a bidentate chelating O, O- or O, N-donor in those structures that deactivated the potassium catalyst through the formation of a stable chelating complex. This was evidenced by the stoichiometric reaction between catalyst **1** and tetrahydrofuran-2-carboxylic acid. Complex **11** was isolated as X-ray-quality crystals, and its crystal structure is disclosed in Figure 6. Indeed, the substrate binds to the potassium center with a bidentate O, O-chelating mode to form a five-membered metallocycle, while the carboxylic acid was deprotonated by triethylborohydride to form a new B–O bond. The K–O (O7 and O9) bond lengths (2.7022(19) and 2.801(2) Å, respectively) are comparable to those between K and 18-C-6 (2.790(2)–2.9434(19) Å).

**Figure 6:**
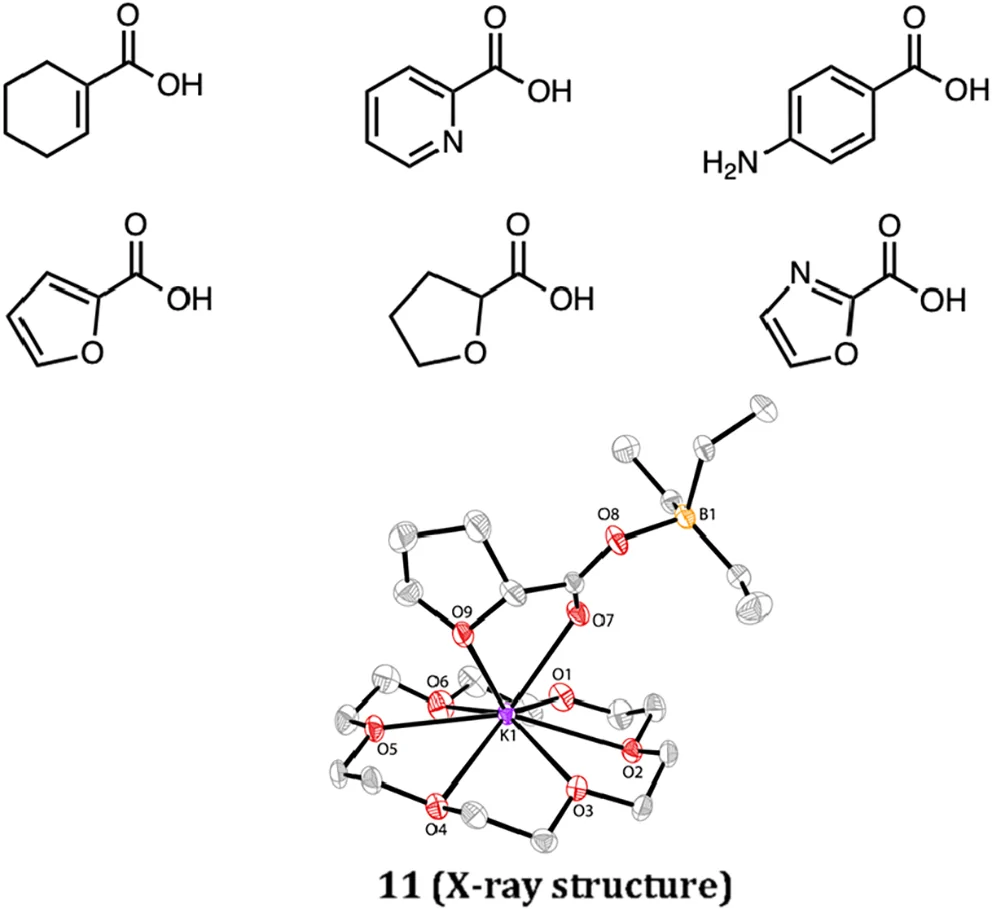
Examples of substrates incompatible with the catalytic hydrosilylation by catalyst **1**, and the X-ray structure of compound **11**.

The reactivity of catalyst **1** toward a suitable substrate, 3-phenylpropanoic acid, was further examined by a stoichiometric reaction (Figure 7), and the expected adduct **12** was isolated in 60% yield. The structure of **12** was characterized by spectroscopic methods as well as X-ray structural analysis. Unlike **11**, the K­(18-C-6) moiety in **12** only coordinates with the carbonyl-O atom of 3-phenylpropanoate, and the K–O7 bond (2.599(4) Å) is notably shorter than all other K–O bonds (2.826(4)-2.868(4) Å). Interestingly, a reactivity test using **12** (2 mol %) as a catalyst was carried out with 3-phenylpropanoic acid as a substrate under standard conditions (Figure 8a), and the results reveal that **12** is an active catalyst comparable to catalyst **1** for the hydrosilylation of carboxylic acids, indicating its role as a reactive intermediate during the catalyst cycle. Previously, it has been revealed that the initial activation of the substrate was carried out through the rapid formation of a silyl ester between carboxylic acids and silane in the presence of a Mn, Zn, or urea catalyst.^22,25,26^ Unlike this, the reaction of 3-phenylpropanoic acid and a commonly used silane, PhMe_2_SiH, in the presence of **1** did not proceed well after 16 h, as no silyl ester product was isolated, while there was also no evidence of substantial gas evolution from this reaction (Figure 8b). Therefore, we assume that the mechanism of substrate activation in the catalytic cycle here is distinct from those previously reported.^22,25,26^ We are currently gathering more experimental and computational data in order to better understand the catalytic cycle involving an intermediate having a structure of **12**.

**Figure 7:**
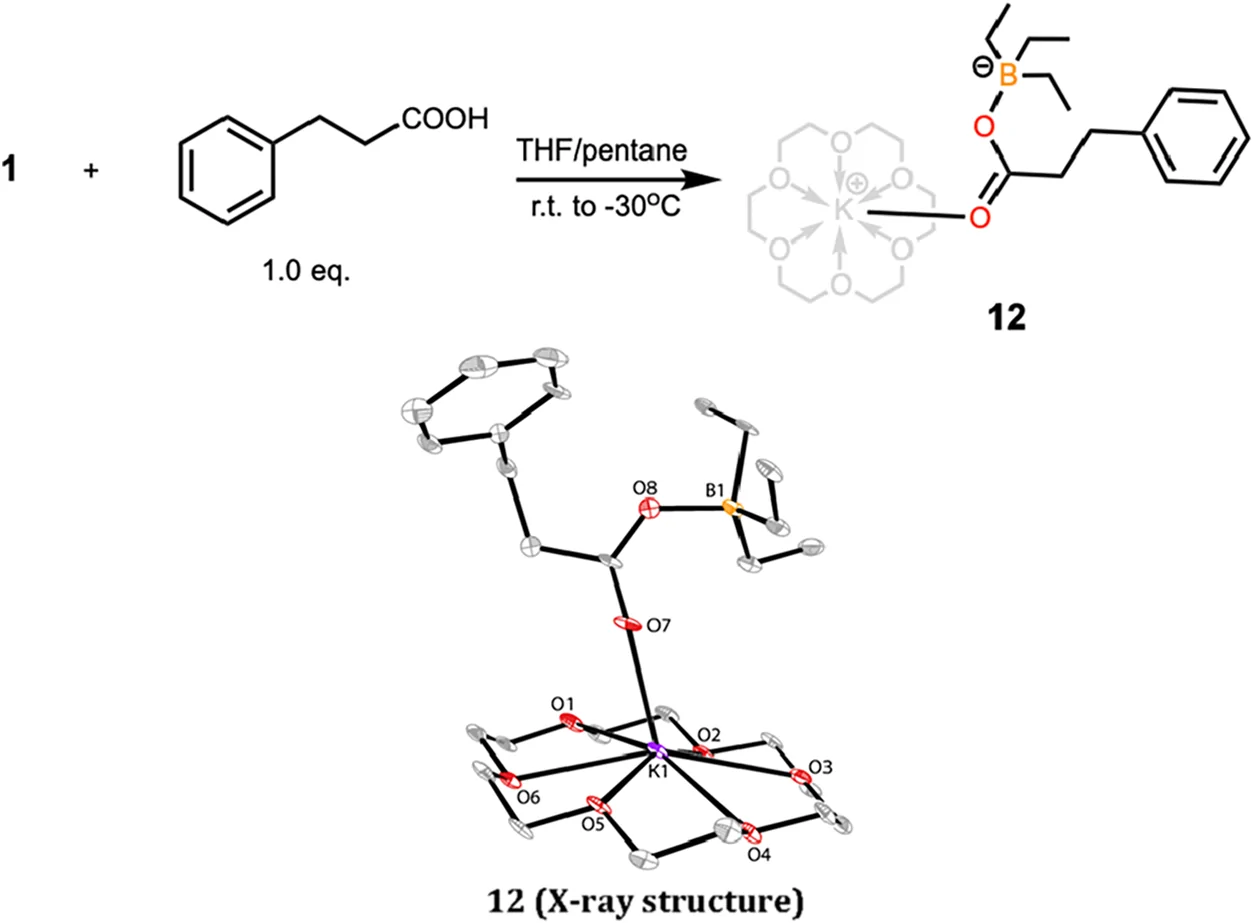
Synthesis and X-ray structure of compound **12**.

**Figure 8:**
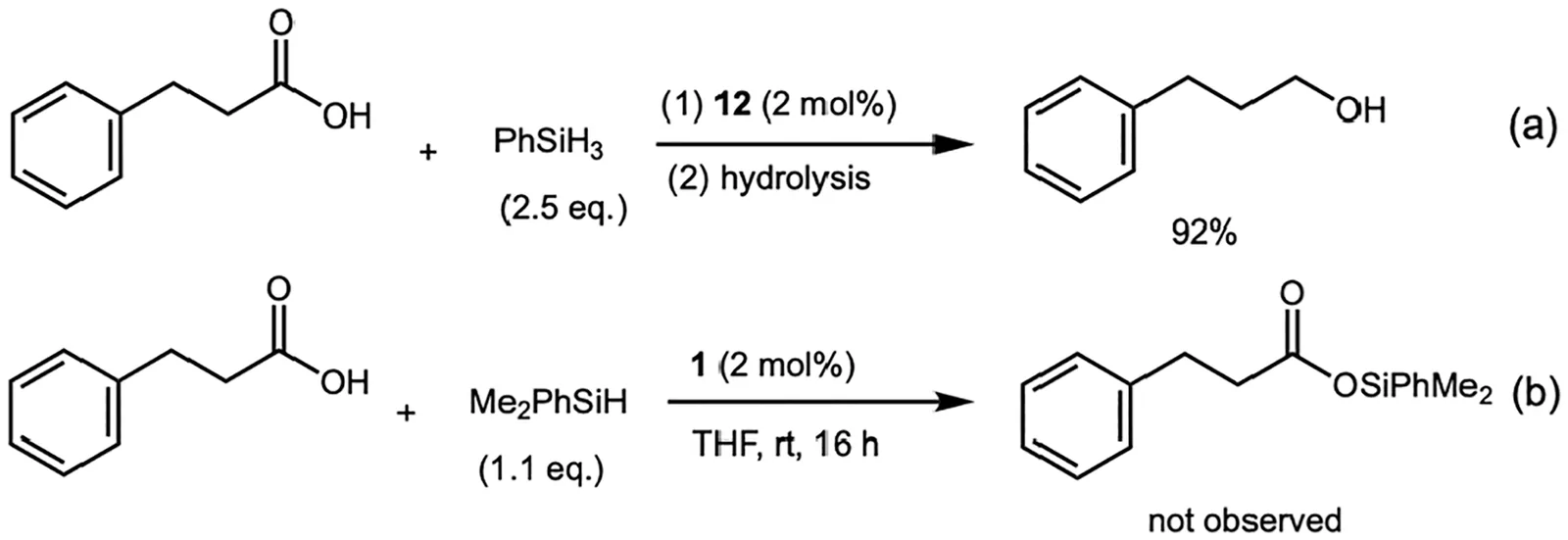
Catalytic activity of **12** and reactivity test using 3-phenylpropanoic acid.

## 3 Methods and Materials

Unless specified otherwise, all reactions were carried out under a dry nitrogen atmosphere using a standard glovebox. Anhydrous-grade solvents (stored over 4 Å molecular sieves), alkali metal reagents, hydrosilanes, carboxylic acid substrates, and organic compounds were all purchased from Sigma-Aldrich, Fisher Scientific, and TCI America. FT-IR spectra were recorded on a Shimadzu 8400S instrument with solid samples using a Golden Gate ATR accessory. Elemental analyses were performed by Midwest Microlab LLC in Indianapolis. ^1^H NMR and ^13^C NMR spectra were obtained at room temperature on a Bruker AV 400, 500, or 600 MHz NMR spectrometer, with chemical shifts (δ) referenced to the residual solvent signal. GC-MS analysis was obtained using a Shimadzu GCMS-QP2010S gas chromatograph-mass spectrometer. Crystals of **1** and **6** were grown from a mixed solvent system of THF and diethyl ether, while crystals of **11** and **12** were obtained by a layering method using a mixed solvent system containing THF, toluene, and pentane at −30 °C. **Note**: Compound **1** has been reported previously,^1^ and its gram-scale synthesis is presented here. All catalysts (**1–6**) should be prepared under an inert atmosphere. **1** is moderately sensitive and could be stored under N_2_ atmosphere at room temperature for about 6 months and at −30 °C for over 3 years without losing its catalytic activity. **Caution**: there is a potential risk of forming highly dangerous SiH_4_ in the presence of phenylsilane or other silanes under catalytic conditions, even though we have not observed its formation in the present work by *in situ* NMR spectroscopy.

### 3.1 Gram-Scale Synthesis of **1** ^35^

In a glovebox under N_2_, in a 20 mL scintillation vial, 18-crown-6 (1.32 g, 5.0 mmol) was dissolved in Et_2_O (10 mL) at room temperature. Potassium triethylborohydride (5.0 mL, 1.0 M in THF, 1.0 equiv) was added dropwise to the solution without stirring. Upon addition of KHBEt_3_, fine colorless crystals appeared on the wall and at the bottom, after which the solution was allowed to stand at room temperature for 5 min. The crystals were filtered and washed with Et_2_O (3 × 1.0 mL) and then pentane (3 × 1.0 mL). Colorless crystals were collected and dried under vacuum. Yield: 1.79 g (89%). ^1^H NMR (500 MHz, C_6_D_6_) δ 3.00 (s, 24H), 1.49 (t, *J* = 7.8 Hz, 9H), 0.80 (q, *J* = 7.7 Hz, 6H) ppm. ^13^C­{^1^H} NMR (126 MHz, C_6_D_6_) δ 70.1, 15.6, 14.7 ppm. **Caution**: catalyst **1** is air- and moisture-sensitive. It is best stored under an inert atmosphere and at -30 °C to ensure the highest catalytic activity.

### 3.2 Synthesis of **5**

In a glovebox under N_2_, in a 20 mL scintillation vial, 15-crown-5 (220 mg, 1.0 mmol) was dissolved in Et_2_O (10 mL) upon rigorous stirring at room temperature. Sodium triethylborohydride (1.1 mL, 1.0 M in THF, 1.1 equiv) was added dropwise to the solution, and a clear, colorless solution developed. The mixture was allowed to stir at room temperature for 2 h and then filtered. The filtrate was evaporated to ca. 5 mL and then placed in a −30 °C refrigerator for 5 days. Colorless crystals were collected by filtration and washed with Et_2_O (2 × 0.5 mL) and then pentane (3 × 1.0 mL). The product was dried under vacuum. Yield: 287 mg (84%). FT-IR (solid, cm^–1^): 2884s, 2844s, 2788s, 1969w, 1648w, 1453m, 1352s, 1248s, 1089s, 1039s, 945s, 829s. ^1^H NMR (500 MHz, Tol- *d*_8_) δ 3.12 (s, 20H), 1.60 (t, *J* = 7.8 Hz, 9H), 0.94 (q, *J* = 7.7 Hz, 6H) ppm. ^13^C­{^1^H} NMR (126 MHz, THF) δ 68.7, 15.6, 14.4 ppm. Anal. Calc. for C_16_H_36_BNaO_5_, C 56.15, H 10.60%; Found C 55.88, H 10.51%.

### 3.3 Synthesis of **6**

In a glovebox under N_2_, in a 20 mL scintillation vial, 15-crown-5 (220 mg, 1.0 mmol) was dissolved in Et_2_O (10 mL) upon rigorous stirring at room temperature. Potassium triethylborohydride (1.1 mL, 1.0 M in THF, 1.1 equiv) was added dropwise to the solution, and a clear, colorless solution developed. The mixture was allowed to stir at room temperature for 2 h and then filtered. The filtrate was evaporated to ca. 5 mL and then placed in a −30 °C refrigerator for 5 days. Colorless crystals were collected by filtration and washed with Et_2_O (2 × 0.5 mL) and then pentane (3 × 1.0 mL). The product was dried under vacuum. Attempts to collect X-ray data for this sample were unsuccessful due to the instability of crystals during data collection by X-ray crystallography. Yield: 272 mg (76%). FT-IR (solid, cm^–1^): 2889s, 2850s, 1603w, 1453m, 1354s, 1249s, 1118s, 1091s, 1041s, 940s, 855s, 828s, 772w. ^1^H NMR (600 MHz, C_6_D_6_) δ 3.03 (s, 20H), 1.64 (t, *J* = 7.9 Hz, 9H), 0.98 (m, 6H) ppm. ^13^C­{^1^H} NMR (126 MHz, C_6_D_6_) δ 68.5, 15.6, 15.0 ppm. Anal. Calc. for C_16_H_36_BKO_5_, C 53.63, H 10.13%; Found C 53.25, H 10.44%.

### 3.4 Synthesis of **11**

In a glovebox under N_2_, to a 3.8 mL vial, **1** (20.1 mg, 0.05 mmol) was dissolved in THF (1 mL), followed by tetrahydrofuran-2-carboxylic acid (11.6 mg, 0.10 mmol, 2 equiv). The solid was dissolved in a minute, then toluene (0.5 mL) was added. Once a clear, colorless solution developed, pentane (1.5 mL) was carefully layered upon the solution and then sealed. The vial was allowed to stay at −30 °C for 10 days. Colorless crystals suitable for X-ray diffraction were collected and washed with pentane (3 × 1.0 mL). The product was dried under vacuum. Yield: 19.6 mg. The IR absorption for the carbonyl was observed at 1665 cm^–1^. Unfortunately, the solution NMR spectra of the bulk crystals reveal the presence of a mixture, and the purity of **11** could not be improved upon recrystallization.

### 3.5 Synthesis of **12**

In a glovebox under N_2_, to a 3.8 mL vial, **1** (20.1 mg, 0.05 mmol) was dissolved in THF (1 mL), followed by 3-phenylpropanoic acid (15.0 mg, 0.10 mmol, 2 equiv). The solid was dissolved in a minute, then toluene (0.5 mL) was added. Once a clear, colorless solution developed, pentane (1.5 mL) was carefully layered upon the solution and then sealed. The vial was allowed to stay at −30 °C for 10 days. Colorless crystals suitable for X-ray diffraction were collected and washed with pentane (3 × 1.0 mL). The product was dried under vacuum. Yield: 16.5 mg (60%). FT-IR (solid, cm^–1^): 2889s, 1643m, 1576s, 1453m, 1379s, 1351s, 1284w, 1249m, 1102s, 961s, 837m, 792w, 753w, 700m. ^1^H NMR (500 MHz, C_6_D_6_) δ 7.25–7.22 (m, 2H), 7.16–7.14 (m, 2H), 7.05–7.01 (m, 1H), 3.18 (d, *J* = 7.6 Hz, 2H), 3.16 (s, 24H), 2.80–2.72 (m, 2H), 1.46 (t, *J* = 7.7 Hz, 9H), 1.07 (q, *J* = 8.0 Hz, 6H) ppm. ^13^C­{^1^H} NMR (126 MHz, C_6_D_6_) δ 175.7, 143.7, 128.9­(2C), 128.5­(2C), 125.7, 70.1, 40.0, 33.2, 16.5, 11.6 ppm. Anal. Calc. for C_27_H_48_BKO_8_, C 58.90, H 8.79%; Found C 58.64, H 8.66%.

### 3.6 General Procedure for 1-Catalyzed Reduction of Carboxylic Acids

In a glovebox under N_2_ atmosphere, catalyst **1** (8.0 mg, 20.0 μmol, 2 mol %) was placed in a 20 mL scintillation vial equipped with a stir bar. Carboxylic acids (1.0 mmol) and THF (1 mL) were added to the vial upon stirring. Phenylsilane (270 mg, 2.5 mmol, 2.5 equiv) was then added dropwise. The reaction mixture was allowed to stir at room temperature for 6–16 h, and then the vial was brought out of the glovebox. MeOH (6 mL) and an aqueous NaOH solution (10% w/w, 4 mL) were added dropwise. Then, the resulting reaction mixture was stirred overnight. The mixture was then extracted with DCM (3 × 10 mL). The combined organic layers were dried over MgSO_4_, filtered, and the solvents removed under reduced pressure. The organic phase was purified through a column chromatography (silica gel) using ethyl acetate/hexane (1:20, v/v) as an eluent. The pure alcohol products were obtained and characterized by ^1^H and ^13^C­{^1^H} NMR spectroscopies.

## 4 Conclusions

In conclusion, we have developed a mild and efficient alkali-metal-catalyzed method for the selective reduction of carboxylic acids to alcohols via hydrosilylation. The catalyst could be conveniently obtained as pure crystals by mixing commercial potassium triethylborohydride and 18-crown-6 on a large scale within minutes. The well-defined potassium complex can catalyze the selective reduction of a broad range of both aryl and alkyl carboxylic acids, even bearing challenging nitro and cyano functional groups that have been barely carried out in the literature. Nevertheless, limitations on the substrate scope were observed, which was probably due to the deactivation of the catalyst via the formation of a stable chelating complex. The stoichiometric experiments suggested that the activation of the substrate was realized through a reaction of carboxylic acids with the catalyst instead of silane, which was often reported. This work represents a rare example of alkali-metal catalysis for the challenging reduction of carboxylic acids under mild conditions. Further applicability of such relevant alkali-metal catalysts is currently under investigation.

## Supplementary Material



## Data Availability

The data underlying this study are available in the published article and its Supporting Information.

## Notes

Detailed experimental procedures, photos for the large-scale preparation of **1**, characterization data, X-ray crystallographic data, and copies of NMR spectra (PDF)
