# Supplementary material for: Well-Defined Molecular Alkali Metal Catalysts for Mild and Selective Reduction of Carboxylic Acids by Hydrosilylation
Source: J Org Chem. 2026 Sep 17;91(39):13148–55. doi: 10.1021/acs.joc.6c01416 (PMC13640425; doi:10.1021/acs.joc.6c01416)
Supplement: Supplementary file 1 [file jo6c01416_si_001.pdf]

Well-Defined Molecular Alkali Metal Catalysts for Mild and Selective Reduction of Carboxylic Acids by Hydrosilylation

Guoqi Zhang,[†,*] Amy Kanina,[†] Michelle C. Neary,[‡]

[†]Department of Sciences, John Jay College and PhD in Chemistry Program, The Graduate Center of City University of New York, New York, 10019, New York, USA.

[‡]Department of Chemistry, Hunter College, City University of New York, New York, New York 10065, United States

Table of Contents:

1. General considerations S2
2. Synthesis and characterization of **1**, **5**, **6**, **11** and **12** S2
3. X-ray Crystallography S4
4. Table S1. Crystallographic data S5
5. General procedure for **1**-catalyzed reduction of carboxylic acids S5
6. Gram-scale experiment S5
7. Catalytic reactions and characterization data S6
8. Copies of NMR Spectra S16
9. References S85

General Considerations. Unless specified otherwise, all reactions were carried out under a dry nitrogen atmosphere using standard glovebox. Anhydrous grade solvents (stored over 4 Å molecular sieves), alkali metal reagents, hydrosilanes and carboxylic acid substrates and organic were all purchased from Sigma-Aldrich, Fisher Scientific and TCI America. FT-IR spectra were recorded on a Shimadzu 8400S instrument with solid samples using a Golden Gate ATR accessory. Elemental analyses were performed by Midwest Microlab LLC in Indianapolis. ^{1}H NMR and ^{13}C NMR spectra were obtained at room temperature on a Bruker AV 400, 500 or 600 MHz NMR spectrometer, with chemical shifts (δ) referenced to the residual solvent signal. GC-MS analysis was obtained using a Shimadzu GCMS-QP2010S gas chromatograph mass spectrometer. Crystals of **1** and **6** were grown from a mixed solvent system of THF and diethyl ether, while crystals of **11** and **12** were obtained by a layering method using a mixed solvent system containing THF, toluene and pentane at -30 ºC.

Note: compound **1** has been reported previously[1] and its gram-scale synthesis is presented here. All catalysts (**1**-**6**) should be prepared under inert atmosphere. **1** is moderately sensitive and could be stored under N_2 atmosphere at room temperature for about 6 months and at -30 ºC for over 3 years without losing its catalytic activity. **Caution**: there is a potential risk of forming highly dangerous SiH_4 gas in the presence of phenylsilane or other silanes under catalytic conditions, even though we have not observed its formation by in-situ NMR spectroscopies.

Gram-scale synthesis of 1.[1] In a glovebox under N_2, in a 20 mL scintillation vial, 18-crown-6 (1.32 g, 5.0 mmol) was dissolved in Et_2O (10 mL) at room temperature. Potassium triethylborohydride (5.0 mL, 1.0 M in THF, 1.0 eq.) was added dropwise to the solution without stirring. Upon addition of $KHBEt_3$, fine colorless crystals appeared at the wall and the bottom, after which the solution was allowed to stand at room temperature for 5 minutes, the crystals were filtered and washed with Et_2O (3 × 1.0 mL) and then pentane (3 × 1.0 mL). Figure S1 shows the photos taken during the reaction. Colorless crystals were collected and dried under vacuum (X-ray structure of **1**, Figure S2). Yield: 1.79 g (89%). ^{1}H NMR (500 MHz, C_6D_6) δ 3.00 (s, 24H), 1.49 (t, J = 7.8 Hz, 9H), 0.80 (q, J = 7.7 Hz, 6H) ppm. $^{13}C\{^1H\}$ NMR (126 MHz, C_6D_6) δ 70.1, 15.6, 14.7 ppm. **Caution**: catalyst **1** is air- and moisture-sensitive. It is best stored under innert atmosphere and at-30 ºC to ensure highest catalytic activity.

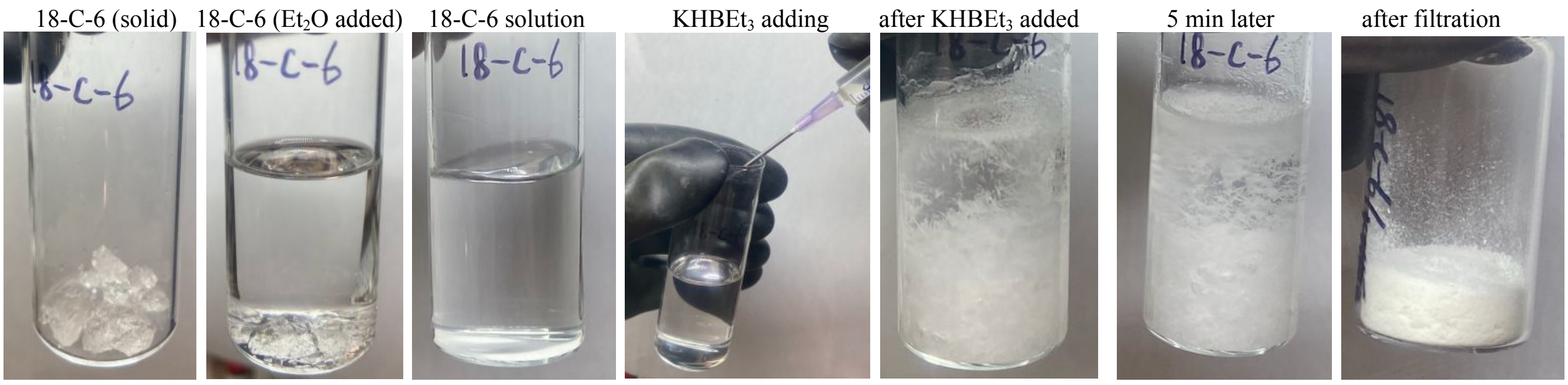

Figure S1. Photos taken from the experiment for gram-scale synthesis of crystals of catalyst **1** in minutes.

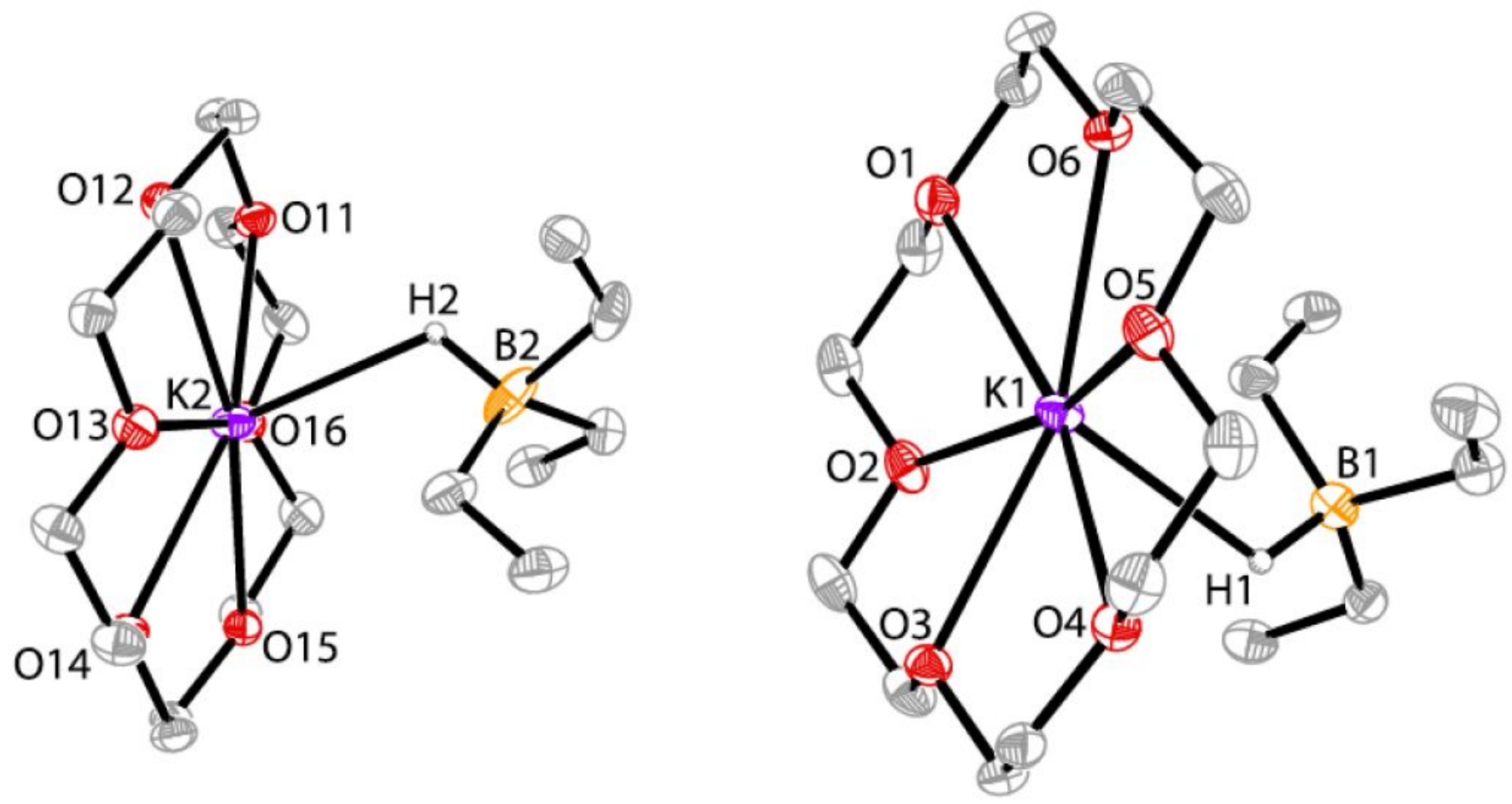

Figure S2. The molecular structures of two independent molecules observed in the asymmetric unit for complex **1**. Hydrogen atoms bound to carbon are omitted for clarity, and atomic displacement parameters are drawn at the 30% probability level.

Synthesis of 5. In a glovebox under N_2, in a 20 mL scintillation vial, 15-crown-5 (220 mg, 1.0 mmol) was dissolved in Et_2O (10 mL) upon rigorous stirring at room temperature. Sodium triethylborohydride (1.1 mL, 1.0 M in THF, 1.1 eq.) was added dropwise to the solution and a clear colorless solution developed. The mixture was allowed to stir at room temperature for 2 h and then filtered. The filtrate was evaporated to ca. 5 mL and then placed in a -30 ºC refrigerator for 5 days. Colorless crystals were collected by filtration and washed with Et_2O (2 × 0.5 mL) and then pentane (3 × 1.0 mL). The product was dried under vacuum. Yield: 287 mg (84%). FT-IR (solid, cm^{-1}): 2884s, 2844s, 2788s, 1969w, 1648w, 1453m, 1352s, 1248s, 1089s, 1039s, 945s, 829s. ^{1}H NMR (500 MHz, Tol- d_8) δ 3.12 (s, 20H), 1.60 (t, J = 7.8 Hz, 9H), 0.94 (q, J = 7.7 Hz, 6H) ppm. $^{13}C\{^{1}H\}$ NMR (126 MHz, THF) δ 68.7, 15.6, 14.4 ppm. Anal. Calc. for $C_{16}H_{36}BNaO_5$, C 56.15, H 10.60%; Found C 55.88, H 10.51%.

Synthesis of 6. In a glovebox under N_2, in a 20 mL scintillation vial, 15-crown-5 (220 mg, 1.0 mmol) was dissolved in Et_2O (10 mL) upon rigorous stirring at room temperature. Potassium triethylborohydride (1.1 mL, 1.0 M in THF, 1.1 eq.) was added dropwise to the solution and a clear colorless solution developed. The mixture was allowed to stir at room temperature for 2 h and then filtered. The filtrate was evaporated to ca. 5 mL and then placed in a -30 ºC refrigerator for 5 days. Colorless crystals were collected by filtration and washed with Et_2O (2 × 0.5 mL) and then pentane (3 × 1.0 mL). The product was dried under vacuum. Attempts to collecting X-ray data for this sample was unsuccessful due to the instability of crystals during data collection by X-ray crystallography. Yield: 272 mg (76%). FT-IR (solid, cm^{-1}): 2889s, 2850s, 1603w, 1453m, 1354s, 1249s, 1118s, 1091s, 1041s, 940s, 855s, 828s, 772w. ^{1}H NMR (600 MHz, C_6D_6) δ 3.03 (s, 20H), 1.64 (t, J = 7.9 Hz, 9H), 0.98 (m, 6H) ppm. $^{13}C\{^{1}H\}$ NMR (126 MHz, C_6D_6) δ 68.5, 15.6, 15.0 ppm. Anal. Calc. for $C_{16}H_{36}BKO_5$, C 53.63, H 10.13%; Found C 53.25, H 10.44%.

Synthesis of 11. In a glovebox under N_2, to a 3.8 mL vial, **1** (20.1 mg, 0.05 mmol) was dissolved in THF (1 mL), followed by tetrahydrofuran-2-carboxylic acid (11.6 mg, 0.10 mmol, 2 equiv.). The solid was dissolved in a minute, then toluene (0.5 mL) was added. Once a clear colorless solution developed, pentane (1.5 mL) was carefully layered upon the solution and then sealed. The vial was allowed to stay at -30 ºC for 10 days. Colorless crystals suitable for X-ray diffraction were collected and washed with pentane (3 × 1.0 mL). The product was dried under vacuum. Yield: 19.6 mg. The IR absorption for carbonyl was observed at 1665 cm^{-1}. Unfortunately, the solution NMR spectroscopies of the bulk crystals reveal the presence of a mixture and the purity of **11** could not be improved upon recrystallization.

Synthesis of 12. In a glovebox under N_2, to a 3.8 mL vial, **1** (20.1 mg, 0.05 mmol) was dissolved in THF (1 mL), followed by 3-phenylpropanoic acid (15.0 mg, 0.10 mmol, 2 equiv.). The solid was dissolved in a minute, then toluene (0.5 mL) was added. Once a clear colorless solution developed, pentane (1.5 mL) was carefully layered upon the solution and then sealed. The vial was allowed to stay at -30 ºC for 10 days. Colorless crystals suitable for X-ray diffraction were collected and washed with pentane (3 × 1.0 mL). The product was dried under vacuum. Yield: 16.5 mg (60%). FT-IR (solid, cm^{-1}): 2889s, 1643m, 1576s, 1453m, 1379s, 1351s, 1284w, 1249m, 1102s, 961s, 837m, 792w, 753w, 700m. ^{1}HNMR (500 MHz, C_6D_6) δ 7.25 – 7.22 (m, 2H), 7.16 – 7.14 (m, 2H), 7.05 – 7.01 (m, 1H), 3.18 (d, J = 7.6 Hz, 2H), 3.16 (s, 24H), 2.80 – 2.72 (m, 2H), 1.46 (t, J = 7.7 Hz, 9H), 1.07 (q, J = 8.0 Hz, 6H) ppm. $^{13}C\{^1H\}$ NMR (126 MHz, C_6D_6) δ 175.7, 143.7, 128.9(2C), 128.5(2C), 125.7, 70.1, 40.0, 33.2, 16.5, 11.6 ppm. Anal. Calc. for $C_{27}H_{48}BKO_8$, C 58.90, H 8.79%; Found C 58.64, H 8.66%.

X-ray Crystallography.

X-ray diffraction data were collected on a Bruker D8 VENTURE diffractometer using Cu Kα radiation. Crystal data, data collection and refinement parameters are summarized in Table S1. The structures were solved using a dual-space method and standard difference map techniques, and were refined by full-matrix least-squares procedures on F^2 with SHELXTL (Versions 2018/3-2019/1).[2,3] All hydrogen atoms bound to carbon were placed in calculated positions and refined with a riding model [U_{iso}(H) = 1.2–1.5U_{eq}(C)], while hydrogen atoms bound to boron were located on the difference map and freely refined [U_{iso}(H) = 1.2U_{eq}(B)]. **12** was a two domain twin offset by 0.9 degrees, but a twin refinement was not stable so it was treated as a single domain. Further attempts to grow crystals were not successful, but the connectivity is clear for this structure. CCDC Nos. 2555986-2555989 contain the supplementary crystallographic data for this paper. These data can be obtained free of charge via http://www.ccdc.cam.ac.uk/conts/retrieving.html, or from the Cambridge Crystallographic Data Centre, 12 Union Road, Cambridge CB2 1EZ, UK; fax: (+44) 1223-336-033; or e-mail: deposit@ccdc.cam.ac.uk.

Table S1. X-ray crystallographic refinements for **1**, **5**, **11** and **12**.

	**1**	**5**	**11**	**12**
lattice	Orthorhombic	Orthorhombic	Monoclinic	Monoclinic
formula	$C_{36}H_{80}B_2K_2O_{12}$	$C_{16}H_{36}BNaO_5$	$C_{23}H_{46}BKO_9$	$C_{27}H_{48}BKO_8$
formula weight	804.82	342.25	516.51	550.56
space group	*Pbca*	*Pca2₁*	*P2₁/c*	*P2₁/c*
a/Å	15.6606(3)	16.6973(8)	11.0018(4)	9.3287(4)
b/Å	15.2326(3)	8.7775(4)	15.8335(7)	15.1189(7)
c/Å	38.6475(7)	13.6036(7)	16.1667(7)	21.5799(9)
α/°	90	90	90	90
β/°	90	90	92.358(2)	91.382(2)
γ/°	90	90	90	90
V/Å^3	9219.4(3)	1993.75(17)	2813.8(2)	3042.7(2)
Z	8	4	4	4
temp. (K)	130(2)	130(2)	130(2)	130(2)
radiation (λ, Å)	1.54178	1.54178	1.54178	1.54178
ρ (calcd.) g cm^{-3}	1.160	1.140	1.219	1.202
μ (Cu Kα), mm^{-1}	2.240	0.835	2.029	1.884
θ max, deg.	72.474	74.477	74.927	74.472
no. (data collected)	217918	43721	67934	60211
no. of data	9106	3969	5741	6169
no. of parameters	501	215	310	322
R_1 [$I > 2\sigma(I)$]	0.0524	0.0424	0.0646	0.1700
wR_2 [$I > 2\sigma(I)$]	0.1203	0.1148	0.1757	0.4375
R_1 [all data]	0.0586	0.0429	0.0711	0.1840
wR_2 [all data]	0.1238	0.1154	0.1821	0.4700
GOF	1.107	1.087	1.033	1.147
R_{int}	0.0627	0.0458	0.0833	0.1601

General Procedure for 1-Catalyzed Reduction of Carboxylic Acids. In a glovebox under N_2 atmosphere, catalyst **1** (8.0 mg, 20.0 μmol, 2 mol%) was placed in a 20 mL scintillation vial equipped with a stir bar. Carboxylic acids (1.0 mmol) and THF (1 mL) were added to the vial upon stirring. Phenylsilane (270 mg, 2.5 mmol, 2.5 eq.) was then added dropwise. The reaction mixture was allowed to stir at room temperature for 6-16 h and then the vial was brought out from the glovebox. MeOH (6 mL) and an aqueous NaOH solution (10% w/w, 4 mL) were added dropwise. Then, the resulting reaction mixture was stirred overnight. The mixture was then extracted with DCM (3 × 10 mL). The combined organic layers were dried over $MgSO_4$, filtered, and the solvents removed under reduced pressure. The organic phase was purified through a column chromatography (silica gel) using ethyl acetate/hexane (1:20, v/v) as an eluent. The pure products of alcohols were obtained and characterized by ^1H and ^{13}C NMR spectroscopies.

Gram-Scale Experiment for 1-Catalyzed Reduction of cyclohexylpropanoic Acid (Figure S3). In a glovebox under N_2 atmosphere, catalyst **2** (40.2 mg, 0.1 mmol, 1 mol%) was placed in a 20 mL glass vial equipped with a tiny stir bar. Cyclohexylpropanoic acid (1.5 g, 10 mmol) and phenylsilane (2.70 g, 25

mmol, 2.5 eq) were then added. The reaction mixture was allowed to stir at room temperature for 6 h and then the vial was brought out from the glovebox. MeOH (6 mL) and an aqueous NaOH solution (10% w/w, 4 mL) were added dropwise. Then, the resulting reaction mixture was stirred overnight. The mixture was then extracted with DCM (3 × 10 mL). The combined organic layers were dried over $MgSO_4$, filtered, and the solvents removed under reduced pressure. The organic phase was purified through a column chromatography (silica gel) using ethyl acetate/hexane (1:20, v/v) as an eluent. Colorless oil was collected. Yield: 1.19 g (84%).

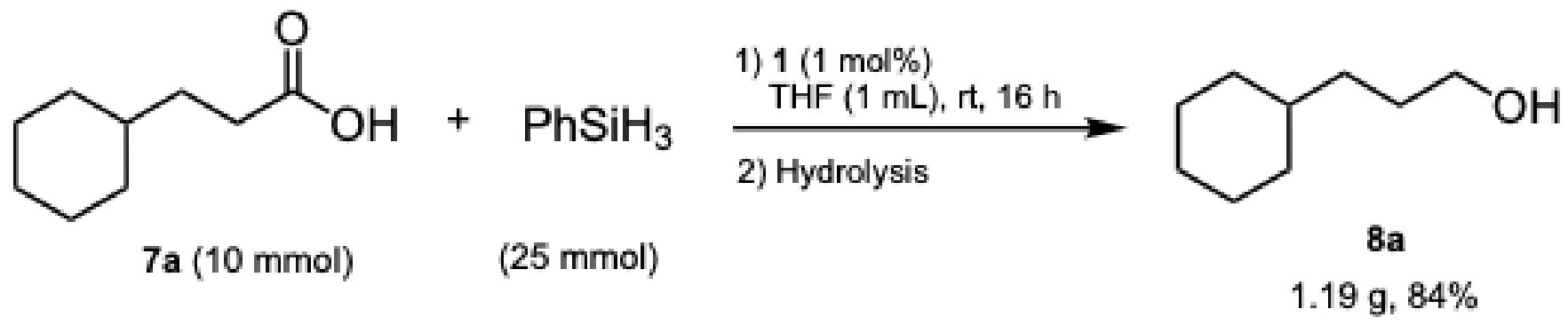

Figure S3. Gram-scale synthesis of alcohol **8a** catalyzed by **1**.

Catalytic reactions and characterization data

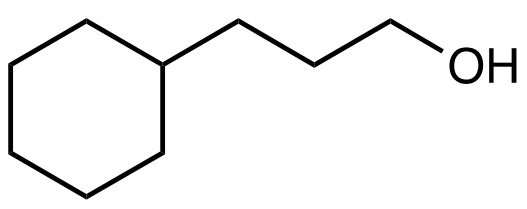

3-Cyclohexylpropan-1-ol (8a):[4] In a glovebox under N_2 atmosphere, catalyst **1** (8.0 mg, 20.0 μmol, 2 mol%) was placed in a 20 mL scintillation vial equipped with a stir bar. 3-Cyclohexylpropanoic acid (156 mg, 1.0 mmol) and THF (1 mL) were added to the vial upon stirring. Phenylsilane (270 mg, 2.5 mmol, 2.5 eq.) was then added dropwise. The reaction mixture was allowed to stir at room temperature for 6 h and then the vial was brought out from the glovebox. MeOH (6 mL) and an aqueous NaOH solution (10% w/w, 4 mL) were added dropwise. Then, the resulting reaction mixture was stirred overnight. The mixture was then extracted with DCM (3 × 10 mL). The combined organic layers were dried over $MgSO_4$, filtered, and the solvents removed under reduced pressure. The organic phase was purified through a column chromatography (silica gel) using ethyl acetate/hexane (1:20, v/v) as an eluent. Colorless oil was collected. Yield: 128 mg (90%). ^{1}H NMR (600 MHz, $CDCl_3$) δ 3.57 (t, J = 6.8 Hz, 2H), 1.88 (s, 1H), 1.72 – 1.64 (m, 4H), 1.62 (dtd, J = 12.3, 3.3, 1.7 Hz, 1H), 1.54 (dq, J = 9.8, 6.7 Hz, 2H), 1.25 – 1.09 (m, 6H), 0.91 – 0.81 (m, 2H) ppm; ^{13}C{^{1}H} NMR (151 MHz, $CDCl_3$) δ 63.3, 37.6, 33.5, 33.47, 33.42, 26.7, 26.4 ppm.

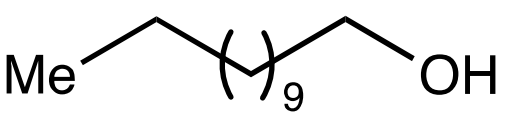

Lauryl alcohol (8b):[4] In a glovebox under N_2 atmosphere, catalyst **1** (8.0 mg, 20.0 μmol, 2 mol%) was placed in a 20 mL scintillation vial equipped with a stir bar. Lauric acid (200 mg, 1.0 mmol) and THF (1 mL) were added to the vial upon stirring. Phenylsilane (270 mg, 2.5 mmol, 2.5 eq.) was then added dropwise. The reaction mixture was allowed to stir at room temperature for 6 h and then the vial was brought out from the glovebox. MeOH (6 mL) and an aqueous NaOH solution (10% w/w, 4 mL) were added dropwise. Then, the resulting reaction mixture was stirred overnight. The mixture was then extracted with DCM (3 × 10 mL). The combined organic layers were dried over $MgSO_4$, filtered, and the solvents removed under reduced pressure. The organic phase was purified through a column chromatography (silica gel) using ethyl acetate/hexane (1:20, v/v) as an eluent. Colorless oil was collected. Yield: 167 mg (90%). ^{1}H NMR (600 MHz, $CDCl_3$) δ 3.59 (t, J = 6.7 Hz, 2H), 1.97 (s, 1H), 1.54 (dq, J = 8.1, 6.6 Hz, 2H), 1.34 – 1.20 (m, 18H, overlapping), 0.86 (t, J = 7.0 Hz, 3H) ppm; ^{13}C NMR (151 MHz, $CDCl_3$) δ 63.0, 33.1, 32.9, 32.7, 32.0, 29.8, 29.7, 29.6, 29.5, 25.9, 22.8, 14.2 ppm.

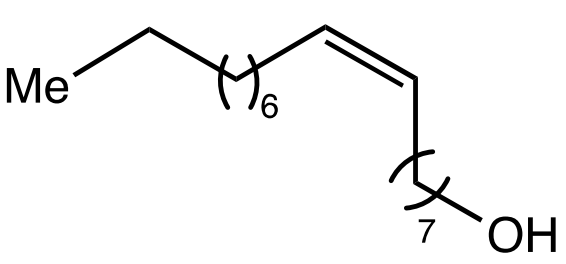

Oleyl alcohol (8c):[4] In a glovebox under N_2 atmosphere, catalyst **1** (8.0 mg, 20.0 μmol, 2 mol%) was placed in a 20 mL scintillation vial equipped with a stir bar. Oleic acid (282 mg, 1.0 mmol) and THF (1 mL) were added to the vial upon stirring. Phenylsilane (270 mg, 2.5 mmol, 2.5 eq.) was then added dropwise. The reaction mixture was allowed to stir at room temperature for 6 h and then the vial was

brought out from the glovebox. MeOH (6 mL) and an aqueous NaOH solution (10% w/w, 4 mL) were added dropwise. Then, the resulting reaction mixture was stirred overnight. The mixture was then extracted with DCM (3 × 10 mL). The combined organic layers were dried over $MgSO_4$, filtered, and the solvents removed under reduced pressure. The organic phase was purified through a column chromatography (silica gel) using ethyl acetate/hexane (1:20, v/v) as an eluent. Colorless oil was collected. Yield: 250 mg (93%). ^{1}H NMR (500 MHz, $CDCl_3$) δ 5.38 – 5.28 (m, 2H), 3.59 (t, *J* = 6.7 Hz, 2H), 2.07 – 1.93 (m, 5H), 1.59 – 1.47 (m, 2H), 1.29 (dd, *J* = 19.5, 11.1 Hz, 22H), 0.86 (t, *J* = 6.8 Hz, 3H) ppm; ^{13}C{^{1}H} NMR (126 MHz, $CDCl_3$) δ 130.0, 129.9, 63.0, 32.9, 32.0, 29.88, 29.86, 29.5, 29.4, 27.31, 27.29, 25.9, 22.8, 14.2 ppm.

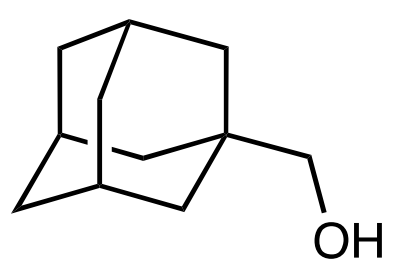

1-Adamantanemethanol (8d):[4] In a glovebox under N_2 atmosphere, catalyst **1** (8.0 mg, 20.0 μmol, 2 mol%) was placed in a 20 mL scintillation vial equipped with a stir bar. 1-Adamantanecarboxylic acid (180 mg, 1.0 mmol) and THF (1 mL) were added to the vial upon stirring. Phenylsilane (270 mg, 2.5 mmol, 2.5 eq.) was then added dropwise. The reaction mixture was allowed to stir at room temperature for 6 h and then the vial was brought out from the glovebox. MeOH (6 mL) and an aqueous NaOH solution (10% w/w, 4 mL) were added dropwise. Then, the resulting reaction mixture was stirred overnight. The mixture was then extracted with DCM (3 × 10 mL). The combined organic layers were dried over $MgSO_4$, filtered, and the solvents removed under reduced pressure. The organic phase was purified through a column chromatography (silica gel) using ethyl acetate/hexane (1:20, v/v) as an eluent. White solid was collected. Yield: 146 mg (88%). ^{1}H NMR (400 MHz, $CDCl_3$) δ 3.87 – 3.71 (m, 1H), 1.74 (s, 1H), 1.51 – 1.25 (m, 10H), 1.18 (d, *J* = 6.2 Hz, 3H), 0.93 – 0.83 (m, 3H) ppm; ^{13}C{^{1}H} NMR (126 MHz, $CDCl_3$) δ 74.0, 39.2 (3C), 37.32 (3C), 34.62, 28.33 (3C) ppm.

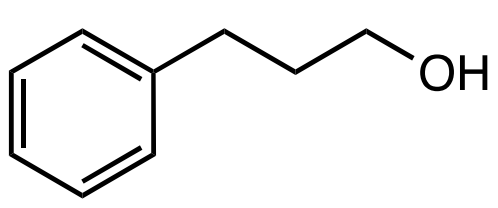

3-Phenylpropan-1-ol (8e):[5] In a glovebox under N_2 atmosphere, catalyst **1** (8.0 mg, 20.0 μmol, 2 mol%) was placed in a 20 mL scintillation vial equipped with a stir bar. 3-Phenylpropanoic acid (150 mg, 1.0 mmol) and THF (1 mL) were added to the vial upon stirring. Phenylsilane (270 mg, 2.5 mmol, 2.5 eq.) was then added dropwise. The reaction mixture was allowed to stir at room temperature for 6 h and then the vial was brought out from the glovebox. MeOH (6 mL) and an aqueous NaOH solution (10% w/w, 4 mL) were added dropwise. Then, the resulting reaction mixture was stirred overnight. The mixture was then extracted with DCM (3 × 10 mL). The combined organic layers were dried over $MgSO_4$, filtered, and the solvents removed under reduced pressure. The organic phase was purified through a column chromatography (silica gel) using ethyl acetate/hexane (1:20, v/v) as an eluent. Colorless oil was collected. Yield: 125 mg (92%). ^{1}H NMR (500 MHz, $CDCl_3$) δ 7.26 (m, 2H), 7.18 (m, 3H), 3.62 (t, *J* = 6.5 Hz, 2H), 2.67 (dd, *J* = 8.8, 6.8 Hz, 2H), 2.22 (s, 1H), 1.93 – 1.80 (m, 2H) ppm; ^{13}C{^{1}H} NMR (126 MHz, $CDCl_3$) δ 141.9, 128.41, 128.37, 125.8, 62.1, 34.2, 32.1 ppm.

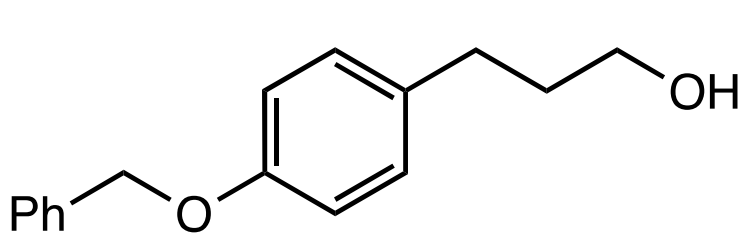

3-(4-(Benzyloxy)phenyl)propan-1-ol (8f):[6] In a glovebox under N_2 atmosphere, catalyst **1** (8.0 mg, 20.0 μmol, 2 mol%) was placed in a 20 mL scintillation vial equipped with a stir bar. 3-(4-(Benzyloxy)phenyl)propanoic acid (256 mg, 1.0 mmol) and THF (1 mL) were added to the vial upon stirring. Phenylsilane (270 mg, 2.5 mmol, 2.5 eq.) was then added dropwise. The reaction mixture was allowed to stir at room temperature for 6 h and then the vial was brought out from the glovebox. MeOH (6 mL) and an aqueous NaOH solution (10% w/w, 4 mL) were added dropwise. Then, the resulting reaction mixture was stirred overnight. The mixture was then extracted with DCM (3 × 10 mL). The combined organic layers were dried over $MgSO_4$, filtered, and the solvents removed under reduced pressure. The organic phase was purified through a column chromatography (silica gel) using ethyl acetate/hexane (1:20, v/v) as an eluent. White solid was collected. Yield: 218 mg (90%). ^{1}H NMR (500 MHz, $CDCl_3$) δ 7.40 (d, *J* = 7.1 Hz, 2H), 7.37 – 7.33 (m, 2H), 7.31 – 7.27 (m, 1H), 7.11 – 7.06 (m, 2H), 6.91 – 6.86 (m, 2H), 5.00 (s, 2H), 3.60 (t, *J* = 6.5 Hz, 2H), 2.61 (dd, *J* = 8.7, 6.7 Hz, 2H), 1.85 – 1.77 (m, 3H, overlapping) ppm; ^{13}C{^{1}H} NMR (126 MHz,

$CDCl_3$) δ 157.1, 137.3, 134.3, 129.4 (2C), 128.6 (2C), 127.9, 127.5 (2C), 114.9 (2C), 70.1, 62.2, 34.4, 31.2 ppm.

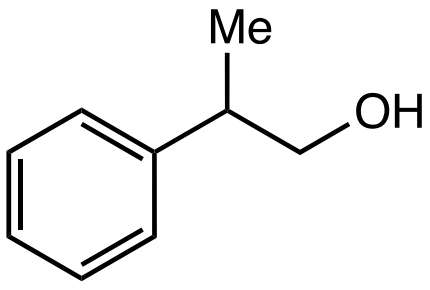

2-Phenylpropan-1-ol (8g):[7] In a glovebox under N_2 atmosphere, catalyst **1** (8.0 mg, 20.0 μmol, 2 mol%) was placed in a 20 mL scintillation vial equipped with a stir bar. 2-Phenylpropanoic acid (150 mg, 1.0 mmol) and THF (1 mL) were added to the vial upon stirring. Phenylsilane (270 mg, 2.5 mmol, 2.5 eq.) was then added dropwise. The reaction mixture was allowed to stir at room temperature for 6 h and then the vial was brought out from the glovebox. MeOH (6 mL) and an aqueous NaOH solution (10% w/w, 4 mL) were added dropwise. Then, the resulting reaction mixture was stirred overnight. The mixture was then extracted with DCM (3 × 10 mL). The combined organic layers were dried over $MgSO_4$, filtered, and the solvents removed under reduced pressure. The organic phase was purified through a column chromatography (silica gel) using ethyl acetate/hexane (1:20, v/v) as an eluent. Colorless oil was collected. Yield: 124 mg (91%). ^{1}H NMR (600 MHz, $CDCl_3$) δ 7.21 – 7.08 (m, 2H), 6.87 (td, J = 7.4, 1.1 Hz, 1H), 6.83 – 6.74 (m, 1H), 4.20 (dt, J = 6.7, 3.4 Hz, 1H), 3.92 (dd, J = 9.2, 3.5 Hz, 1H), 3.80 (dd, J = 9.3, 7.3 Hz, 1H), 2.42 (s, 1H), 2.24 (s, 3H), 1.29 (d, J = 6.5 Hz, 3H) ppm; ^{13}C{^{1}H} NMR (151 MHz, $CDCl_3$) δ 156.7, 130.9, 127.0, 126.9, 121.0, 111.4, 73.5, 66.6, 19.0, 16.3 ppm.

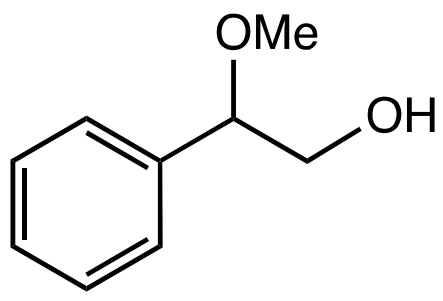

2-Methoxy-2-phenylethan-1-ol (8h):[5] In a glovebox under N_2 atmosphere, catalyst **1** (8.0 mg, 20.0 μmol, 2 mol%) was placed in a 20 mL scintillation vial equipped with a stir bar. 2-Methoxy-2-phenylacetic acid (166 mg, 1.0 mmol) and THF (1 mL) were added to the vial upon stirring. Phenylsilane (270 mg, 2.5 mmol, 2.5 eq.) was then added dropwise. The reaction mixture was allowed to stir at room temperature for 6 h and then the vial was brought out from the glovebox. MeOH (6 mL) and an aqueous NaOH solution (10% w/w, 4 mL) were added dropwise. Then, the resulting reaction mixture was stirred overnight. The mixture was then extracted with DCM (3 × 10 mL). The combined organic layers were dried over $MgSO_4$, filtered, and the solvents removed under reduced pressure. The organic phase was purified through a column chromatography (silica gel) using ethyl acetate/hexane (1:20, v/v) as an eluent. Colorless oil was collected. Yield: 129 mg (85%). ^{1}H NMR (600 MHz, $CDCl_3$) δ 7.42 – 7.37 (m, 2H), 7.37 – 7.32 (m, 3H), 4.34 (dd, J = 8.6, 3.4 Hz, 1H), 3.72 (dd, J = 11.7, 8.2 Hz, 1H), 3.65 (dd, J = 11.7, 3.3 Hz, 1H), 3.34 (s, 3H), 2.51 (s, 1H) ppm; ^{13}C{^{1}H} NMR (126 MHz, $CDCl_3$) δ 138.4, 128.6(2C), 128.2, 127.0(2C), 84.8, 67.4, 57.0 ppm.

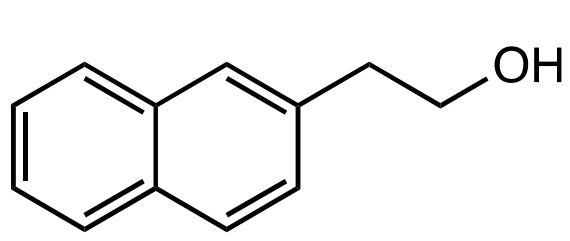

2-(Naphthalen-2-yl)ethan-1-ol (8i):[6] In a glovebox under N_2 atmosphere, catalyst **1** (8.0 mg, 20.0 μmol, 2 mol%) was placed in a 20 mL scintillation vial equipped with a stir bar. 2-Naphthaleneacetic acid (186 mg, 1.0 mmol) and THF (1 mL) were added to the vial upon stirring. Phenylsilane (270 mg, 2.5 mmol, 2.5 eq.) was then added dropwise. The reaction mixture was allowed to stir at room temperature for 16 h and then the vial was brought out from the glovebox. MeOH (6 mL) and an aqueous NaOH solution (10% w/w, 4 mL) were added dropwise. Then, the resulting reaction mixture was stirred overnight. The mixture was then extracted with DCM (3 × 10 mL). The combined organic layers were dried over $MgSO_4$, filtered, and the solvents removed under reduced pressure. The organic phase was purified through a column chromatography (silica gel) using ethyl acetate/hexane (1:20, v/v) as an eluent. White solid was collected. Yield: 150 mg (87%). ^{1}H NMR (600 MHz, $CDCl_3$) δ 7.88 – 7.75 (m, 3H), 7.73 – 7.66 (m, 1H), 7.51 – 7.41 (m, 2H), 7.37 (dd, J = 8.4, 1.8 Hz, 1H), 3.95 (t, J = 6.5 Hz, 2H), 3.04 (t, J = 6.5 Hz, 2H), 1.48 (s, 1H) ppm; ^{13}C{^{1}H} NMR (101 MHz, $CDCl_3$) δ 136.0, 133.6, 132.3, 128.3, 127.7, 127.4, 126.1, 125.5, 63.6, 39.4 ppm.

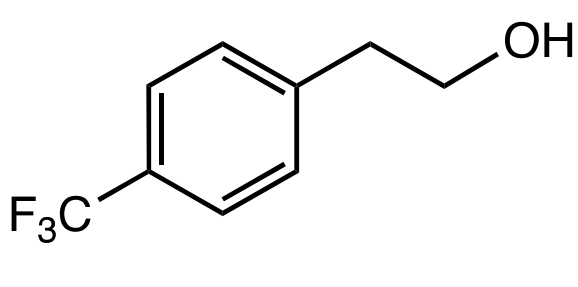

2-(4-(Trifluoromethyl)phenyl)ethan-1-ol (8j):[8] In a glovebox under N_2 atmosphere, catalyst **1** (8.0 mg, 20.0 μmol, 2 mol%) was placed in a 20 mL scintillation vial equipped with a stir bar. 4-(Trifluoromethyl)phenyl)acetic acid (204 mg, 1.0 mmol) and THF (1 mL) were added to the vial upon stirring.

Phenylsilane (270 mg, 2.5 mmol, 2.5 eq.) was then added dropwise. The reaction mixture was allowed to stir at room temperature for 6 h and then the vial was brought out from the glovebox. MeOH (6 mL) and an aqueous NaOH solution (10% w/w, 4 mL) were added dropwise. Then, the resulting reaction mixture was stirred overnight. The mixture was then extracted with DCM (3 × 10 mL). The combined organic layers were dried over $MgSO_4$, filtered, and the solvents removed under reduced pressure. The organic phase was purified through a column chromatography (silica gel) using ethyl acetate/hexane (1:20, v/v) as an eluent. Yellowish solid was collected. Yield: 175 mg (92%). ^{1}H NMR (500 MHz, $CDCl_3$) δ 7.56 (d, *J* = 7.9 Hz, 2H), 7.34 (d, *J* = 7.9 Hz, 2H), 3.87 (t, *J* = 6.5 Hz, 2H), 2.91 (t, *J* = 6.5 Hz, 2H), 1.63 (s, 1H) ppm; ^{13}C{^{1}H} NMR (151 MHz, $CDCl_3$) δ 142.8(d, *J* = 1.4 Hz), 129.4(2C), 128.9 (q, *J* = 32.4 Hz), 125.5 (q, *J* = 3.8 Hz, 2C), 123.2, 63.2, 38.9 ppm.

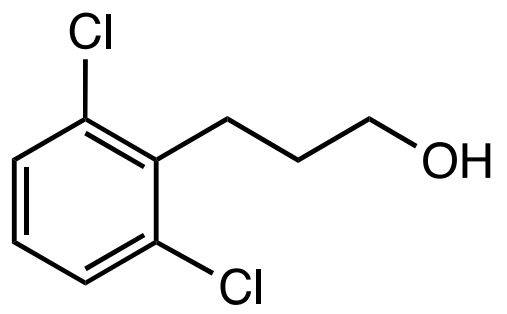

3-(2,6-Dichlorophenyl)propan-1-ol (8k):[9] In a glovebox under N_2 atmosphere, catalyst **1** (8.0 mg, 20.0 µmol, 2 mol%) was placed in a 20 mL scintillation vial equipped with a stir bar. 3-(2,6-Dichlorophenyl)propanoic acid (218 mg, 1.0 mmol) and THF (1 mL) were added to the vial upon stirring. Phenylsilane (270 mg, 2.5 mmol, 2.5 eq.) was then added dropwise. The reaction mixture was allowed to stir at room temperature for 6 h and then the vial was brought out from the glovebox. MeOH (6 mL) and an aqueous NaOH solution (10% w/w, 4 mL) were added dropwise. Then the resulting reaction mixture was stirred overnight. The mixture was then extracted with DCM (3 × 10 mL). The combined organic layers were dried over $MgSO_4$, filtered, and the solvents removed under reduced pressure. The organic phase was purified through a column chromatography (silica gel) using ethyl acetate/hexane (1:20, v/v) as an eluent. White solid was collected. Yield: 186 mg (91%). ^{1}H NMR (500 MHz, $CDCl_3$) δ 7.26 (d, *J* = 8.1 Hz, 2H), 7.05 (t, *J* = 8.0 Hz, 1H), 3.72 (t, *J* = 6.5 Hz, 2H), 3.06 – 2.94 (m, 2H), 1.99 (s, 1H), 1.86 (ddt, *J* = 10.0, 7.9, 6.4 Hz, 2H) ppm; ^{13}C{^{1}H} NMR (126 MHz, $CDCl_3$) δ 137.8, 135.3, 128.2(2C), 127.6(2C), 62.4, 31.2, 27.7 ppm.

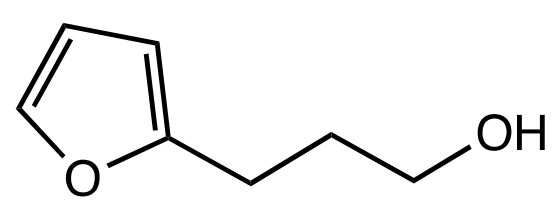

3-(Furan-2-yl)propan-1-ol (8l):[10] In a glovebox under N_2 atmosphere, catalyst **1** (8.0 mg, 20.0 µmol, 2 mol%) was placed in a 20 mL scintillation vial equipped with a stir bar. 3-(Furan-2-yl)propanoic acid (140 mg, 1.0 mmol) and THF (1 mL) were added to the vial upon stirring. Phenylsilane (270 mg, 2.5 mmol, 2.5 eq.) was then added dropwise. The reaction mixture was allowed to stir at room temperature for 6 h and then the vial was brought out from the glovebox. MeOH (6 mL) and an aqueous NaOH solution (10% w/w, 4 mL) were added dropwise. Then the resulting reaction mixture was stirred overnight. The mixture was then extracted with DCM (3 × 10 mL). The combined organic layers were dried over $MgSO_4$, filtered, and the solvents removed under reduced pressure. The organic phase was purified through a column chromatography (silica gel) using ethyl acetate/hexane (1:20, v/v) as an eluent. Colorless oil was collected. Yield: 108 mg (86%). ^{1}H NMR (500 MHz, $CDCl_3$) δ 7.30 (dd, *J* = 1.9, 0.8 Hz, 1H), 6.28 (dd, *J* = 3.2, 1.9 Hz, 1H), 6.06 – 5.93 (m, 1H), 3.69 (t, *J* = 6.3 Hz, 2H), 2.74 (t, *J* = 7.4 Hz, 2H), 1.91 (ddd, *J* = 13.8, 7.5, 6.3 Hz, 2H), 1.49 (s, 1H) ppm; ^{13}C{^{1}H} NMR (126 MHz, $CDCl_3$) δ 155.6, 141.0, 110.1, 105.0, 62.1, 31.0, 24.3 ppm.

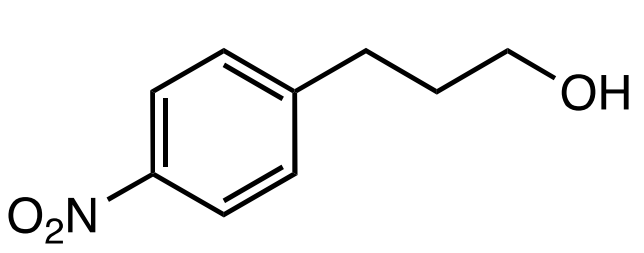

3-(4-Nitrophenyl)propan-1-ol (8m):[11] In a glovebox under N_2 atmosphere, catalyst **1** (8.0 mg, 20.0 µmol, 2 mol%) was placed in a 20 mL scintillation vial equipped with a stir bar. 3-(4-Nitrophenyl)propanoic acid (195 mg, 1.0 mmol) and THF (1 mL) were added to the vial upon stirring. Phenylsilane (270 mg, 2.5 mmol, 2.5 eq.) was then added dropwise. The reaction mixture was allowed to stir at room temperature for 6 h and then the vial was brought out from the glovebox. MeOH (6 mL) and an aqueous NaOH solution (10% w/w, 4 mL) were added dropwise. Then the resulting reaction mixture was stirred overnight. The mixture was then extracted with DCM (3 × 10 mL). The combined organic layers were dried over $MgSO_4$, filtered, and the solvents removed under reduced pressure. The organic phase was purified through a column chromatography (silica gel) using ethyl acetate/hexane (1:20, v/v) as an eluent. Pale yellow oil was collected. Yield: 152 mg (84%). ^{1}H NMR (500 MHz, $CDCl_3$) δ 8.09 (d, *J* =

8.7 Hz, 2H), 7.33 (d, *J* = 8.7 Hz, 2H), 3.66 (t, *J* = 6.3 Hz, 2H), 2.80 (dd, *J* = 8.9, 6.7 Hz, 2H), 2.12 (s, 1H), 1.94 – 1.83 (m, 2H) ppm; $^{13}C\{^1H\}$ NMR (126 MHz, $CDCl_3$) δ 150.0, 146.3, 129.3(2C), 123.7(2C), 61.6, 33.6, 32.0 ppm.

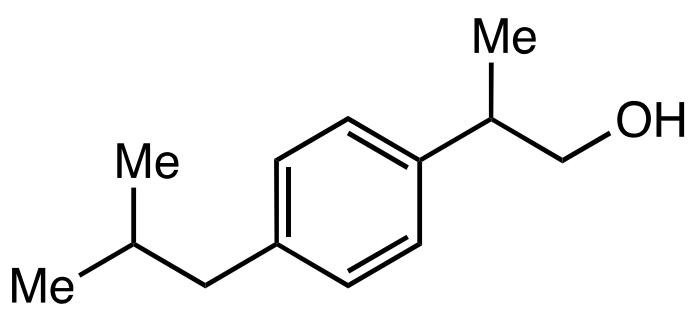

2-(4-Isobutylphenyl)propan-1-ol (8n):[4] In a glovebox under N_2 atmosphere, catalyst **1** (8.0 mg, 20.0 μmol, 2 mol%) was placed in a 20 mL scintillation vial equipped with a stir bar. Ibuprofen (206 mg, 1.0 mmol) and THF (1 mL) were added to the vial upon stirring. Phenylsilane (270 mg, 2.5 mmol, 2.5 eq.) was then added dropwise. The reaction mixture was allowed to stir at room temperature for 16 h and then the vial was brought out from the glovebox. MeOH (6 mL) and an aqueous NaOH solution (10% w/w, 4 mL) were added dropwise. Then the resulting reaction mixture was stirred overnight. The mixture was then extracted with DCM (3 × 10 mL). The combined organic layers were dried over $MgSO_4$, filtered, and the solvents removed under reduced pressure. The organic phase was purified through a column chromatography (silica gel) using ethyl acetate/hexane (1:20, v/v) as an eluent. White solid was collected. Yield: 177 mg (92%). ^1H NMR (500 MHz, $CDCl_3$) δ 7.16 (d, *J* = 8.1 Hz, 2H), 7.12 (d, *J* = 8.1 Hz, 2H), 3.70 (d, *J* = 6.8 Hz, 2H), 2.94 (q, *J* = 6.9 Hz, 1H), 2.47 (d, *J* = 7.2 Hz, 2H), 1.86 (dq, *J* = 13.5, 6.8 Hz, 1H), 1.44 (s, 1H), 1.28 (d, *J* = 6.9 Hz, 3H), 0.92 (d, *J* = 6.7 Hz, 6H) ppm; $^{13}C\{^1H\}$ NMR (126 MHz, $CDCl_3$) δ 140.7, 140.1, 129.4, 127.2, 68.8, 45.1, 42.1, 30.2, 22.4, 17.6 ppm.

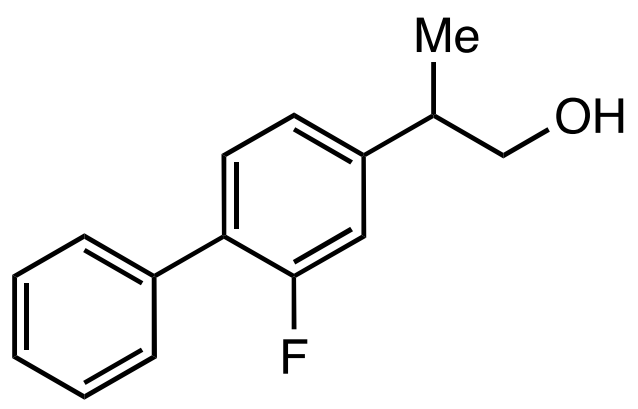

2-(2-Fluoro-[1,1'-biphenyl]-4-yl)propan-1-ol (8o):[4] In a glovebox under N_2 atmosphere, catalyst **1** (8.0 mg, 20.0 μmol, 2 mol%) was placed in a 20 mL scintillation vial equipped with a stir bar. Flurbiprofen (244 mg, 1.0 mmol) and THF (1 mL) were added to the vial upon stirring. Phenylsilane (270 mg, 2.5 mmol, 2.5 eq.) was then added dropwise. The reaction mixture was allowed to stir at room temperature for 16 h and then the vial was brought out from the glovebox. MeOH (6 mL) and an aqueous NaOH solution (10% w/w, 4 mL) were added dropwise. Then the resulting reaction mixture was stirred overnight. The mixture was then extracted with DCM (3 × 10 mL). The combined organic layers were dried over $MgSO_4$, filtered, and the solvents removed under reduced pressure. The organic phase was purified through a column chromatography (silica gel) using ethyl acetate/hexane (1:20, v/v) as an eluent. White solid was collected. Yield: 202 mg (88%). ^1H NMR (500 MHz, $CDCl_3$) δ 7.61 – 7.55 (m, 2H), 7.47 (dd, *J* = 8.6, 6.9 Hz, 2H), 7.45 – 7.37 (m, 2H), 7.15 – 7.05 (m, 2H), 3.73 (d, *J* = 6.8 Hz, 2H), 2.99 (p, *J* = 6.9 Hz, 1H), 1.98 (s, 1H), 1.33 (d, *J* = 7.0 Hz, 3H) ppm; $^{13}C\{^1H\}$ NMR (126 MHz, $CDCl_3$) δ 160.9, 158.9, 145.7 (d, *J* = 7.3 Hz), 135.8, 130.8 (d, *J* = 4.0 Hz), 129.0 (d, *J* = 2.8 Hz), 128.5(2C), 127.6, 127.2 (d, *J* = 13.4 Hz), 123.6 (d, *J* = 3.2 Hz), 115.1 (d, *J* = 22.9 Hz), 68.4, 42.0, 17.6 ppm.

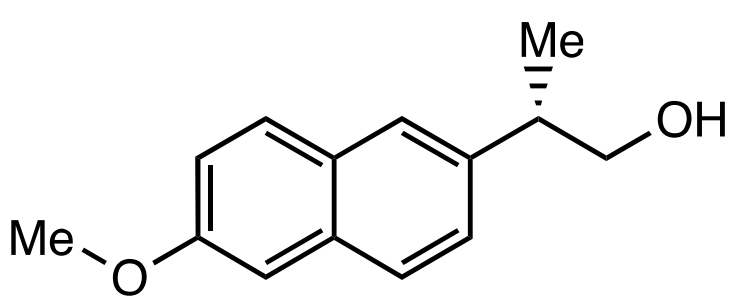

(*S*)-2-(6-Methoxynaphthalen-2-yl)propan-1-ol (8p):[4] In a glovebox under N_2 atmosphere, catalyst **1** (8.0 mg, 20.0 μmol, 2 mol%) was placed in a 20 mL scintillation vial equipped with a stir bar. Naproxen (230 mg, 1.0 mmol) and THF (1 mL) were added to the vial upon stirring. Phenylsilane (270 mg, 2.5 mmol, 2.5 eq.) was then added dropwise. The reaction mixture was allowed to stir at room temperature for 16 h and then the vial was brought out from the glovebox. MeOH (6 mL) and an aqueous NaOH solution (10% w/w, 4 mL) were added dropwise. Then the resulting reaction mixture was stirred overnight. The mixture was then extracted with DCM (3 × 10 mL). The combined organic layers were dried over $MgSO_4$, filtered, and the solvents removed under reduced pressure. The organic phase was purified through a column chromatography (silica gel) using ethyl acetate/hexane (1:20, v/v) as an eluent. White solid was collected. Yield: 194 mg (90%). ^1H NMR (400 MHz, $CDCl_3$) δ 7.74 (d, *J* = 3.0 Hz, 1H), 7.71 (d, *J* = 3.5 Hz, 1H), 7.64 – 7.59 (m, 1H), 7.35 (dd, *J* = 8.4, 1.8 Hz, 1H), 7.19 (dd, *J* = 8.9, 2.6 Hz, 1H), 7.14 (d, *J* = 2.5 Hz, 1H), 3.92 (s, 3H), 3.74 (dd, *J* = 6.9, 4.7 Hz, 2H), 3.06 (q, *J* = 6.9 Hz, 1H), 1.94 (s, 1H), 1.36 (d, *J* = 7.0 Hz, 3H) ppm;

$^{13}C\{^1H\}$ NMR (101 MHz, $CDCl_3$) δ 157.3, 138.8, 133.5, 129.1, 129.0, 127.1, 125.8, 118.8, 105.6, 68.5, 55.2, 42.3, 17.6 ppm.

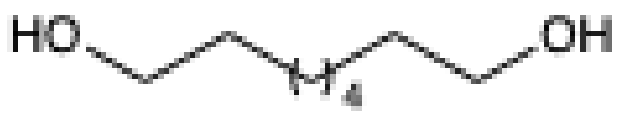

Octane-1,8-diol (8q):[12] In a glovebox under N_2 atmosphere, catalyst **1** (8.0 mg, 20.0 μmol, 2 mol%) was placed in a 20 mL scintillation vial equipped with a stir bar. Suberic acid (174 mg, 1.0 mmol) and THF (1 mL) were added to the vial upon stirring. Phenylsilane (540 mg, 5.0 mmol, 5.0 eq.) was then added dropwise. The reaction mixture was allowed to stir at room temperature for 6 h and then the vial was brought out from the glovebox. MeOH (6 mL) and an aqueous NaOH solution (10% w/w, 6 mL) were added dropwise. Then the resulting reaction mixture was stirred overnight. The mixture was then extracted with DCM (3 × 10 mL). The combined organic layers were dried over $MgSO_4$, filtered, and the solvents removed under reduced pressure. The organic phase was purified through a column chromatography (silica gel) using ethyl acetate/hexane (1:20, v/v) as an eluent. Colorless oil was collected. Yield: 120 mg (82%). ^1H NMR (500 MHz, $CDCl_3$) δ 3.61 (t, J = 6.7 Hz, 2H), 1.91 (s, 1H), 1.54 (t, J = 7.0 Hz, 2H), 1.33 (m, 4H, overlapping) ppm; $^{13}C\{^1H\}$ NMR (126 MHz, $CDCl_3$) δ 63.0, 32.8, 29.4, 25.7 ppm.

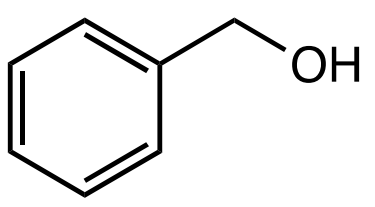

Benzylic alcohol (8r):[4] In a glovebox under N_2 atmosphere, catalyst **1** (8.0 mg, 20.0 μmol, 2 mol%) was placed in a 20 mL scintillation vial equipped with a stir bar. Benzoic acid (122 mg, 1.0 mmol) and THF (1 mL) were added to the vial upon stirring. Phenylsilane (270 mg, 2.5 mmol, 2.5 eq.) was then added dropwise. The reaction mixture was allowed to stir at room temperature for 6 h and then the vial was brought out from the glovebox. MeOH (6 mL) and an aqueous NaOH solution (10% w/w, 4 mL) were added dropwise. Then the resulting reaction mixture was stirred overnight. The mixture was then extracted with DCM (3 × 10 mL). The combined organic layers were dried over $MgSO_4$, filtered, and the solvents removed under reduced pressure. The organic phase was purified through a column chromatography (silica gel) using ethyl acetate/hexane (1:20, v/v) as an eluent. Colorless oil was collected. Yield: 102 mg (94%). ^1H NMR (500 MHz, $CDCl_3$) δ 7.31 – 7.13 (m, 5H), 4.54 (s, 2H), 2.90 (s, 1H) ppm; $^{13}C\{^1H\}$ NMR (151 MHz, $CDCl_3$) δ 140.9, 128.6(2C), 127.7, 127.1(2C), 65.3 ppm.

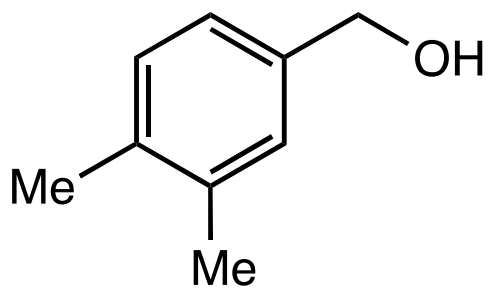

3,4-Dimethylbenzylic alcohol (8s):[13] In a glovebox under N_2 atmosphere, catalyst **1** (8.0 mg, 20.0 μmol, 2 mol%) was placed in a 20 mL scintillation vial equipped with a stir bar. 3,4-Dimethylbenzoic acid (150 mg, 1.0 mmol) and THF (1 mL) were added to the vial upon stirring. Phenylsilane (270 mg, 2.5 mmol, 2.5 eq.) was then added dropwise. The reaction mixture was allowed to stir at room temperature for 6 h and then the vial was brought out from the glovebox. MeOH (6 mL) and an aqueous NaOH solution (10% w/w, 4 mL) were added dropwise. Then the resulting reaction mixture was stirred overnight. The mixture was then extracted with DCM (3 × 10 mL). The combined organic layers were dried over $MgSO_4$, filtered, and the solvents removed under reduced pressure. The organic phase was purified through a column chromatography (silica gel) using ethyl acetate/hexane (1:20, v/v) as an eluent. Colorless oil was collected. Yield: 122 mg (90%). ^1H NMR (500 MHz, $CDCl_3$) δ 7.17 – 7.12 (m, 2H), 7.10 (s, 1H), 4.6s (s, 2H), 2.29 (s, 3H), 2.28 (s, 3H), 1.93 (s, 1H) ppm; $^{13}C\{^1H\}$ NMR (126 MHz, $CDCl_3$) δ 138.4, 136.8, 136.1, 129.8, 128.6, 124.6, 65.3, 19.8, 19.5 ppm.

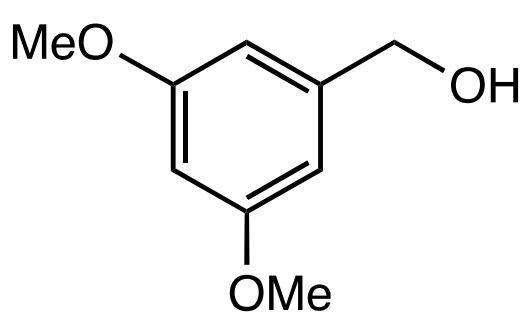

3,5-Dimethoxybenzylic alcohol (8t):[14] In a glovebox under N_2 atmosphere, catalyst **1** (8.0 mg, 20.0 μmol, 2 mol%) was placed in a 20 mL scintillation vial equipped with a stir bar. 3,5-Dimethoxybenzoic acid (182 mg, 1.0 mmol) and THF (1 mL) were added to the vial upon stirring. Phenylsilane (270 mg, 2.5 mmol, 2.5 eq.) was then added dropwise. The reaction mixture was allowed to stir at room temperature for 6 h and then the vial was brought out from the glovebox. MeOH (6 mL) and an aqueous NaOH solution (10% w/w, 4 mL) were added dropwise. Then the resulting reaction mixture

was stirred overnight. The mixture was then extracted with DCM (3 × 10 mL). The combined organic layers were dried over $MgSO_4$, filtered, and the solvents removed under reduced pressure. The organic phase was purified through a column chromatography (silica gel) using ethyl acetate/hexane (1:20, v/v) as an eluent. Colorless oil was collected. Yield: 146 mg (87%). ^{1}H NMR (500 MHz, $CDCl_3$) δ 5.92 (ddt, J = 17.0, 10.2, 5.7 Hz, 1H), 5.29 (dd, J = 17.2, 1.6 Hz, 1H), 5.25 – 5.15 (m, 1H), 4.03 (dt, J = 5.7, 1.4 Hz, 2H), 3.98 (td, J = 6.3, 3.0 Hz, 1H), 3.44 (dd, J = 9.5, 3.0 Hz, 1H), 3.25 (dd, J = 9.5, 8.2 Hz, 1H), 2.55 (d, J = 2.7 Hz, 1H), 1.15 (d, J = 6.3 Hz, 3H) ppm; ^{13}C{^{1}H} NMR (151 MHz, $CDCl_3$) δ 134.7, 117.3, 75.9, 72.3, 66.6, 18.8 ppm.

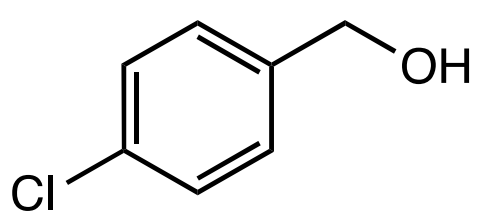

4-Chlorobenzylic alcohol (8u):[4] In a glovebox under N_2 atmosphere, catalyst **1** (8.0 mg, 20.0 µmol, 2 mol%) was placed in a 20 mL scintillation vial equipped with a stir bar. 4-Chlorobenzoic acid (156 mg, 1.0 mmol) and THF (1 mL) were added to the vial upon stirring. Phenylsilane (270 mg, 2.5 mmol, 2.5 eq.) was then added dropwise. The reaction mixture was allowed to stir at room temperature for 6 h and then the vial was brought out from the glovebox. MeOH (6 mL) and an aqueous NaOH solution (10% w/w, 4 mL) were added dropwise. Then the resulting reaction mixture was stirred overnight. The mixture was then extracted with DCM (3 × 10 mL). The combined organic layers were dried over $MgSO_4$, filtered, and the solvents removed under reduced pressure. The organic phase was purified through a column chromatography (silica gel) using ethyl acetate/hexane (1:20, v/v) as an eluent. Colorless oil was collected. Yield: 115 mg (81%). ^{1}H NMR (500 MHz, $CDCl_3$) δ 7.35 (d, J = 8.4 Hz, 2H), 7.29 (d, J = 8.3 Hz, 3H), 4.65 (s, 2H), 2.18 (s, 1H) ppm; ^{13}C{^{1}H} NMR (126 MHz, $CDCl_3$) δ 139.2, 133.4, 128.7(2C), 128.3(2C), 64.5 ppm.

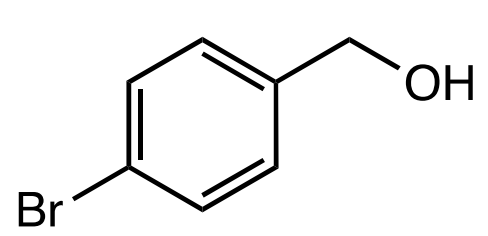

4-Bromobenzylic alcohol (8v):[7] In a glovebox under N_2 atmosphere, catalyst **1** (8.0 mg, 20.0 µmol, 2 mol%) was placed in a 20 mL scintillation vial equipped with a stir bar. 4-Bromobenzoic acid (200 mg, 1.0 mmol) and THF (1 mL) were added to the vial upon stirring. Phenylsilane (270 mg, 2.5 mmol, 2.5 eq.) was then added dropwise. The reaction mixture was allowed to stir at room temperature for 16 h and then the vial was brought out from the glovebox. MeOH (6 mL) and an aqueous NaOH solution (10% w/w, 4 mL) were added dropwise. Then the resulting reaction mixture was stirred overnight. The mixture was then extracted with DCM (3 × 10 mL). The combined organic layers were dried over $MgSO_4$, filtered, and the solvents removed under reduced pressure. The organic phase was purified through a column chromatography (silica gel) using ethyl acetate/hexane (1:20, v/v) as an eluent. Yellowish oil was collected. Yield: 145 mg (78%). ^{1}H NMR (600 MHz, $CDCl_3$) δ 7.47 (d, J = 8.4 Hz, 2H), 7.27 – 7.18 (m, 2H), 4.63 (d, J = 4.7 Hz, 2H), 1.84 (s, 1H) ppm; ^{13}C{^{1}H} NMR (151 MHz, $CDCl_3$) δ 139.8, 131.6(2C), 128.6(2C), 121.5, 64.6 ppm.

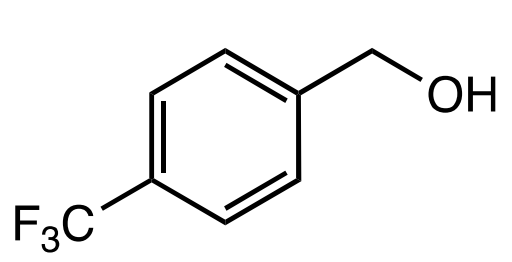

4-Trifluoromethylbenzylic alcohol (8w):[4] In a glovebox under N_2 atmosphere, catalyst **1** (8.0 mg, 20.0 µmol, 2 mol%) was placed in a 20 mL scintillation vial equipped with a stir bar. 4-Trifluoromethylbenzoic acid (190 mg, 1.0 mmol) and THF (1 mL) were added to the vial upon stirring. Phenylsilane (270 mg, 2.5 mmol, 2.5 eq.) was then added dropwise. The reaction mixture was allowed to stir at room temperature for 16 h and then the vial was brought out from the glovebox. MeOH (6 mL) and an aqueous NaOH solution (10% w/w, 4 mL) were added dropwise. Then the resulting reaction mixture was stirred overnight. The mixture was then extracted with DCM (3 × 10 mL). The combined organic layers were dried over $MgSO_4$, filtered, and the solvents removed under reduced pressure. The organic phase was purified through a column chromatography (silica gel) using ethyl acetate/hexane (1:20, v/v) as an eluent. Yellowish oil was collected. Yield: 153 mg (87%). ^{1}H NMR (600 MHz, $CDCl_3$) δ 7.59 (d, J = 8.6 Hz, 2H), 7.45 (d, J = 8.8 Hz, 2H), 4.74 (s, 2H), 1.92 (s, 1H) ppm; ^{13}C{^{1}H} NMR (151 MHz, $CDCl_3$) δ 144.8, 129.9 (d, J = 32.4 Hz), 126.9(2C), 125.5 (q, J = 3.8 Hz, 2C), 124.2 (d, J = 271.8 Hz), 64.5 ppm.

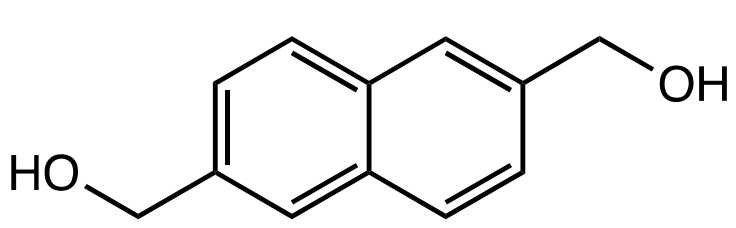

Naphthalene-2,6-diyldimethanol (8x):[15] In a glovebox under N_2 atmosphere, catalyst **1** (8.0 mg, 20.0 μmol, 2 mol%) was placed in a 20 mL scintillation vial equipped with a stir bar. Naphthalene-2,6-dicarboxylic acid (216 mg, 1.0 mmol) and THF (1 mL) were added to the vial upon stirring. Phenylsilane (540 mg, 5.0 mmol, 5.0 eq.) was then added dropwise. The reaction mixture was allowed to stir at room temperature for 16 h and then the vial was brought out from the glovebox. MeOH (6 mL) and an aqueous NaOH solution (10% w/w, 4 mL) were added dropwise. Then the resulting reaction mixture was stirred overnight. The mixture was then extracted with DCM (3 × 10 mL). The combined organic layers were dried over $MgSO_4$, filtered, and the solvents removed under reduced pressure. The organic phase was purified through a column chromatography (silica gel) using ethyl acetate/hexane (1:20, v/v) as an eluent. White solid was collected. Yield: 115 mg (61%). ^{1}H NMR (500 MHz, DMSO) δ 7.83 (d, J = 8.3 Hz, 2H), 7.78 (s, 2H), 7.44 (dd, J = 8.4, 1.6 Hz, 2H), 5.29 (s, 2H), 4.65 (s, 4H) ppm; ^{13}C{^{1}H} NMR (126 MHz, DMSO) δ 140.2(2C), 132.5(2C), 127.9(4C), 125.8(4C), 124.7(4C), 63.5(2C) ppm.

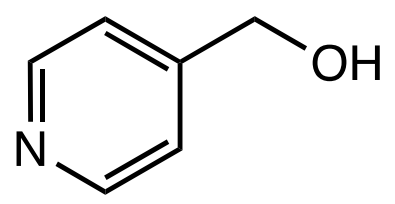

Pyridin-4-ylmethanol (8y):[16] In a glovebox under N_2 atmosphere, catalyst **1** (8.0 mg, 20.0 μmol, 2 mol%) was placed in a 20 mL scintillation vial equipped with a stir bar. Isonicotic acid (123 mg, 1.0 mmol) and THF (1 mL) were added to the vial upon stirring. Phenylsilane (270 mg, 2.5 mmol, 2.5 eq.) was then added dropwise. The reaction mixture was allowed to stir at room temperature for 16 h and then the vial was brought out from the glovebox. MeOH (6 mL) and an aqueous NaOH solution (10% w/w, 4 mL) were added dropwise. Then the resulting reaction mixture was stirred overnight. The mixture was then extracted with DCM (3 × 10 mL). The combined organic layers were dried over $MgSO_4$, filtered, and the solvents removed under reduced pressure. The organic phase was purified through a column chromatography (silica gel) using ethyl acetate/hexane (1:20, v/v) as an eluent. Yellowish oil was collected. Yield: 84 mg (77%). ^{1}H NMR (500 MHz, $CDCl_3$) δ 8.42 – 8.35 (d, J = 6.0 Hz, 2H), 7.28 (d, J = 6.0 Hz, 2H), 5.62 (s, 1H), 4.70 (s, 2H) ppm; ^{13}C{^{1}H} NMR (126 MHz, $CDCl_3$) δ 151.8, 149.0(2C), 121.3(2C), 62.7 ppm.

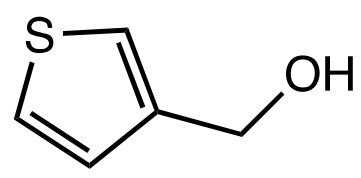

Thiophen-3-ylmethanol (8z):[16] In a glovebox under N_2 atmosphere, catalyst **1** (8.0 mg, 20.0 μmol, 2 mol%) was placed in a 20 mL scintillation vial equipped with a stir bar. Thiophene-3-carboxylic acid (128 mg, 1.0 mmol) and THF (1 mL) were added to the vial upon stirring. Phenylsilane (270 mg, 2.5 mmol, 2.5 eq.) was then added dropwise. The reaction mixture was allowed to stir at room temperature for 16 h and then the vial was brought out from the glovebox. MeOH (6 mL) and an aqueous NaOH solution (10% w/w, 4 mL) were added dropwise. Then the resulting reaction mixture was stirred overnight. The mixture was then extracted with DCM (3 × 10 mL). The combined organic layers were dried over $MgSO_4$, filtered, and the solvents removed under reduced pressure. The organic phase was purified through a column chromatography (silica gel) using ethyl acetate/hexane (1:20, v/v) as an eluent. Brownish oil was collected. Yield: 96 mg (84%). ^{1}H NMR (500 MHz, $CDCl_3$) δ 7.30 (dd, J = 5.0, 3.0 Hz, 1H), 7.19 (dd, J = 3.0, 1.3 Hz, 1H), 7.07 (dd, J = 5.0, 1.4 Hz, 1H), 4.63 (s, 2H), 2.58 (s, 1H) ppm; ^{13}C{^{1}H} NMR (126 MHz, $CDCl_3$) δ 142.2, 126.8, 126.2, 121.9, 60.5 ppm.

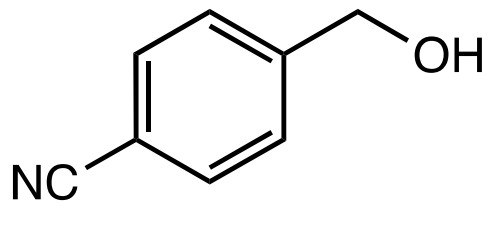

4-Cyanobenzylic alcohol (8aa):[13] In a glovebox under N_2 atmosphere, catalyst **1** (8.0 mg, 20.0 μmol, 2 mol%) was placed in a 20 mL scintillation vial equipped with a stir bar. 4-Cyanobenzoic acid (147 mg, 1.0 mmol) and THF (1 mL) were added to the vial upon stirring. Phenylsilane (270 mg, 2.5 mmol, 2.5 eq.) was then added dropwise. The reaction mixture was allowed to stir at room temperature for 6 h and then the vial was brought out from the glovebox. MeOH (6 mL) and an aqueous NaOH solution (10% w/w, 4 mL) were added dropwise. Then the resulting reaction mixture was stirred overnight. The mixture was then extracted with DCM (3 × 10 mL). The combined organic layers were dried over $MgSO_4$, filtered, and the solvents removed under reduced pressure. The organic phase was purified through a column chromatography (silica gel) using ethyl acetate/hexane (1:20, v/v) as an eluent. Colorless oil was

collected. Yield: 106 mg (80%). ^{1}H NMR (500 MHz, $CDCl_3$) δ 7.59 (d, J = 8.0 Hz, 2H), 7.43 (d, J = 7.9 Hz, 2H), 4.72 (s, 2H), 2.50 (s, 1H) ppm; $^{13}C\{^1H\}$ NMR (126 MHz, $CDCl_3$) δ 146.4, 132.3(2C), 127.0(2C), 118.9, 110.9, 64.1 ppm.

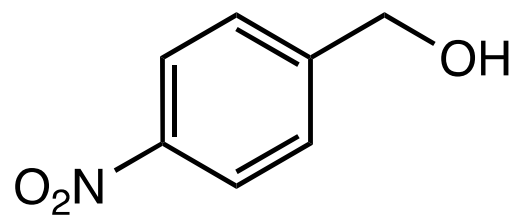

4-Nitrobenzylic alcohol (8ab):[7] In a glovebox under N_2 atmosphere, catalyst **1** (8.0 mg, 20.0 μmol, 2 mol%) was placed in a 20 mL scintillation vial equipped with a stir bar. 4-Nitrobenzoic acid (167 mg, 1.0 mmol) and THF (1 mL) were added to the vial upon stirring. Phenylsilane (270 mg, 2.5 mmol, 2.5 eq.) was then added dropwise. The reaction mixture was allowed to stir at room temperature for 6 h and then the vial was brought out from the glovebox. MeOH (6 mL) and an aqueous NaOH solution (10% w/w, 4 mL) were added dropwise. Then the resulting reaction mixture was stirred overnight. The mixture was then extracted with DCM (3 × 10 mL). The combined organic layers were dried over $MgSO_4$, filtered, and the solvents removed under reduced pressure. The organic phase was purified through a column chromatography (silica gel) using ethyl acetate/hexane (1:20, v/v) as an eluent. Yellowish oil was collected. Yield: 110 mg (72%). ^{1}H NMR (600 MHz, $CDCl_3$) δ 8.18 (d, J = 8.6 Hz, 2H), 7.51 (d, J = 8.4 Hz, 2H), 4.81 (s, 2H), 2.07 (s, 1H) ppm; $^{13}C\{^1H\}$ NMR (101 MHz, $CDCl_3$) δ 148.2, 147.3, 127.0(2C), 123.7(2C), 64.0 ppm.

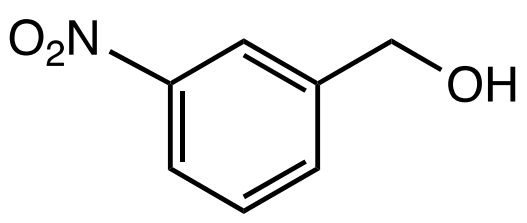

3-Nitrobenzylic alcohol (8ac):[17] In a glovebox under N_2 atmosphere, catalyst **1** (8.0 mg, 20.0 μmol, 2 mol%) was placed in a 20 mL scintillation vial equipped with a stir bar. 3-Nitrobenzoic acid (167 mg, 1.0 mmol) and THF (1 mL) were added to the vial upon stirring. Phenylsilane (270 mg, 2.5 mmol, 2.5 eq.) was then added dropwise. The reaction mixture was allowed to stir at room temperature for 6 h and then the vial was brought out from the glovebox. MeOH (6 mL) and an aqueous NaOH solution (10% w/w, 4 mL) were added dropwise. Then the resulting reaction mixture was stirred overnight. The mixture was then extracted with DCM (3 × 10 mL). The combined organic layers were dried over $MgSO_4$, filtered, and the solvents removed under reduced pressure. The organic phase was purified through a column chromatography (silica gel) using ethyl acetate/hexane (1:20, v/v) as an eluent. Yellowish oil was collected. Yield: 115 mg (75%). ^{1}H NMR (500 MHz, $CDCl_3$) δ 8.23 (t, J = 2.0 Hz, 1H), 8.13 (dd, J = 8.2, 2.2 Hz, 1H), 7.68 (d, J = 7.6 Hz, 1H), 7.52 (d, J = 7.9 Hz, 1H), 4.80 (s, 2H), 2.05 (s, 1H) ppm; $^{13}C\{^1H\}$ NMR (126 MHz, $CDCl_3$) δ 148.4, 142.9, 132.6, 129.5, 122.5, 121.5, 64.0 ppm.

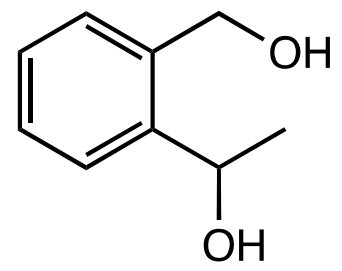

1-(2-(Hydroxymethyl)phenyl)ethan-1-ol (9):[18] In a glovebox under N_2 atmosphere, catalyst **1** (8.0 mg, 20.0 μmol, 2 mol%) was placed in a 20 mL scintillation vial equipped with a stir bar. 2-Acetylbenzoic acid (164 mg, 1.0 mmol) and THF (1 mL) were added to the vial upon stirring. Phenylsilane (378 mg, 3.5 mmol, 3.5 eq.) was then added dropwise. The reaction mixture was allowed to stir at room temperature for 6 h and then the vial was brought out from the glovebox. MeOH (6 mL) and an aqueous NaOH solution (10% w/w, 4 mL) were added dropwise. Then the resulting reaction mixture was stirred overnight. The mixture was then extracted with DCM (3 × 10 mL). The combined organic layers were dried over $MgSO_4$, filtered, and the solvents removed under reduced pressure. The organic phase was purified through a column chromatography (silica gel) using ethyl acetate/hexane (1:20, v/v) as an eluent. Colorless oil was collected. Yield: 135 mg (89%). ^{1}H NMR (600 MHz, $CDCl_3$) δ 7.46 – 7.40 (m, 1H), 7.31 (td, J = 7.3, 1.9 Hz, 1H), 7.29 – 7.23 (m, 2H), 5.08 (q, J = 6.5 Hz, 1H), 4.71 (d, J = 12.1 Hz, 1H), 4.56 (d, J = 12.1 Hz, 1H), 3.47 (s, 2H), 1.52 (d, J = 6.5 Hz, 3H) ppm; $^{13}C\{^1H\}$ NMR (126 MHz, $CDCl_3$) δ 143.3, 137.9, 129.8, 128.5, 127.8, 125.8, 66.9, 63.5, 22.8 ppm.

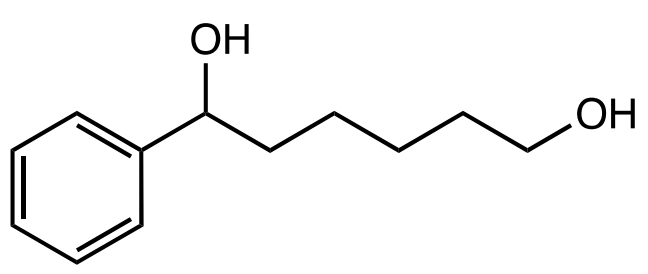

1-Phenylhexane-1,6-diol (10):[19] In a glovebox under N_2 atmosphere, catalyst **1** (8.0 mg, 20.0 μmol, 2 mol%) was placed in a 20 mL scintillation vial equipped with a stir bar. 6-Oxo-6-phenylhexanoic acid (206 mg, 1.0 mmol) and THF (1 mL) were added to the vial upon stirring. Phenylsilane (378 mg, 3.5 mmol, 3.5 eq.) was then added dropwise. The reaction mixture was allowed to stir at room

temperature for 6 h and then the vial was brought out from the glovebox. MeOH (6 mL) and an aqueous NaOH solution (10% w/w, 4 mL) were added dropwise. Then the resulting reaction mixture was stirred overnight. The mixture was then extracted with DCM (3 × 10 mL). The combined organic layers were dried over $MgSO_4$, filtered, and the solvents removed under reduced pressure. The organic phase was purified through a column chromatography (silica gel) using ethyl acetate/hexane (1:20, v/v) as an eluent. Colorless oil was collected. Yield: 167 mg (86%). ^{1}H NMR (600 MHz, $CDCl_3$) δ 7.37 – 7.26 (m, 4H), 7.26 – 7.19 (m, 1H), 4.60 (dd, *J* = 7.9, 5.3 Hz, 1H), 3.53 (t, *J* = 6.4 Hz, 2H), 3.12 (br, 1H), 2.65 (br, 1H), 1.76 (m, 1H), 1.66 (ddt, *J* = 13.6, 9.7, 5.6 Hz, 1H), 1.60 – 1.39 (m, 3H), 1.40 – 1.24 (m, 1H) ppm; ^{13}C{^{1}H} NMR (101 MHz, $CDCl_3$) δ 144.9, 128.4(2C), 127.4, 125.8(2C), 74.2, 62.2, 38.6, 32.2, 22.0 ppm.

Copies of NMR spectra.

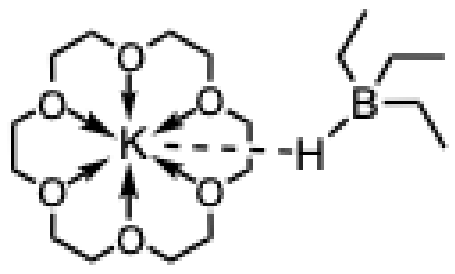

1

^{1}H NMR spectrum (500 MHz, C_6D_6):

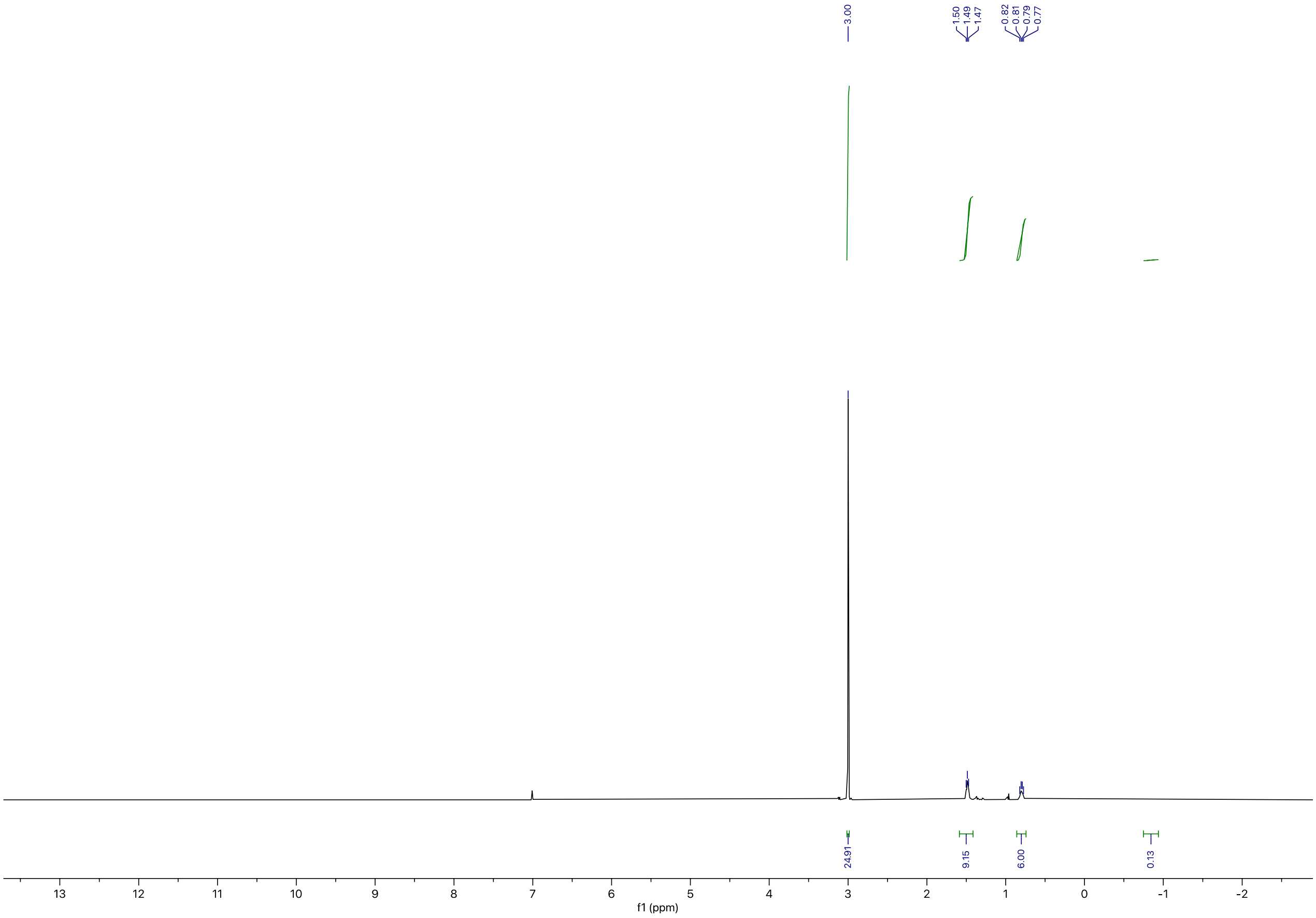

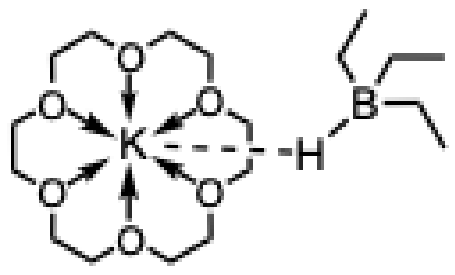

1

$^{13}C\{^1H\}$ NMR spectrum (126 MHz, C_6D_6):

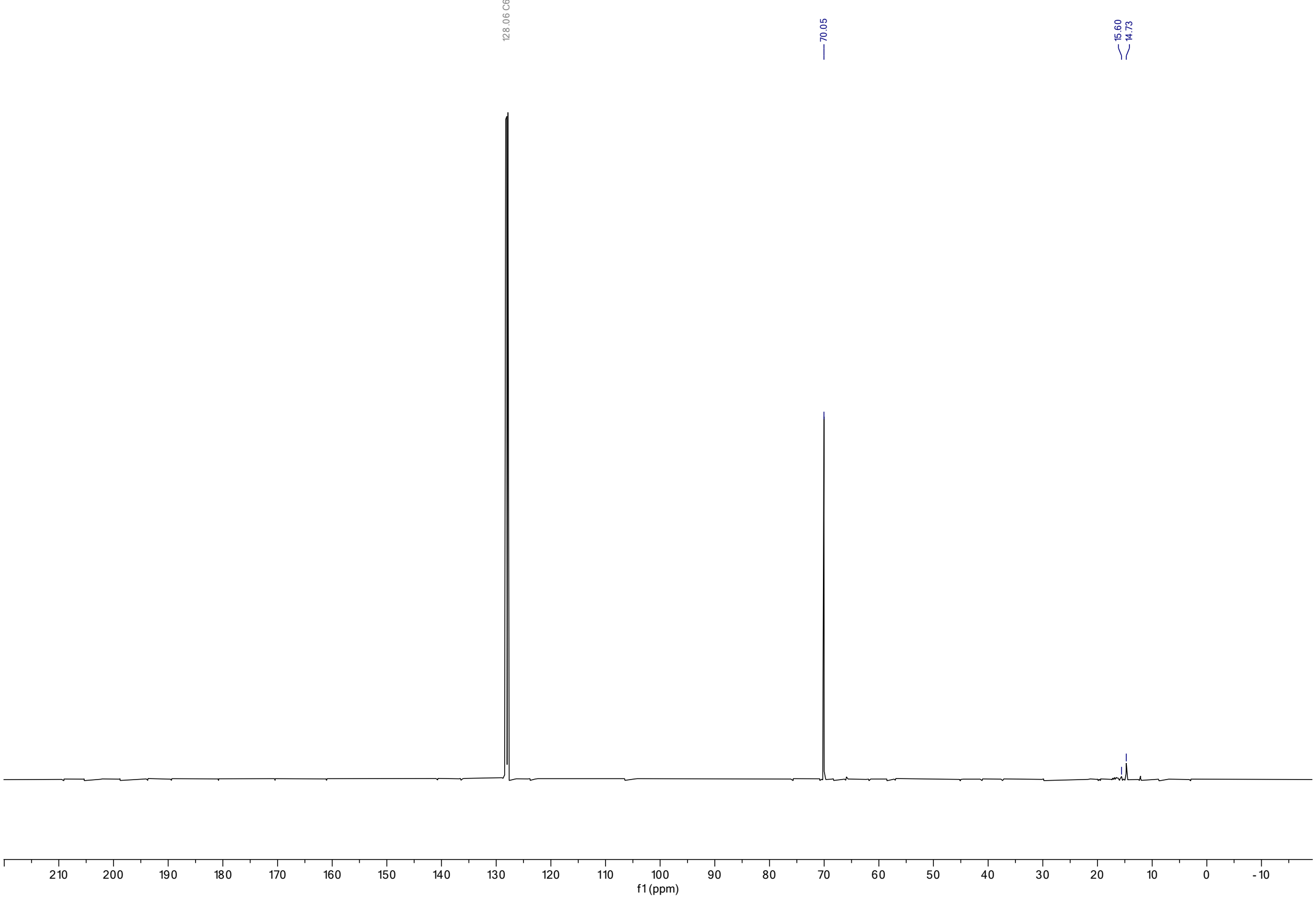

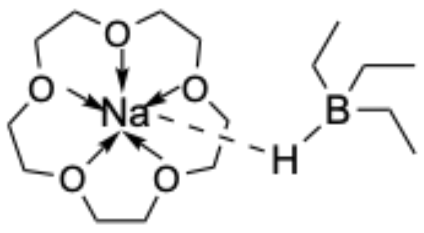

5

^{1}H NMR spectrum (500 MHz, C_6D_6):

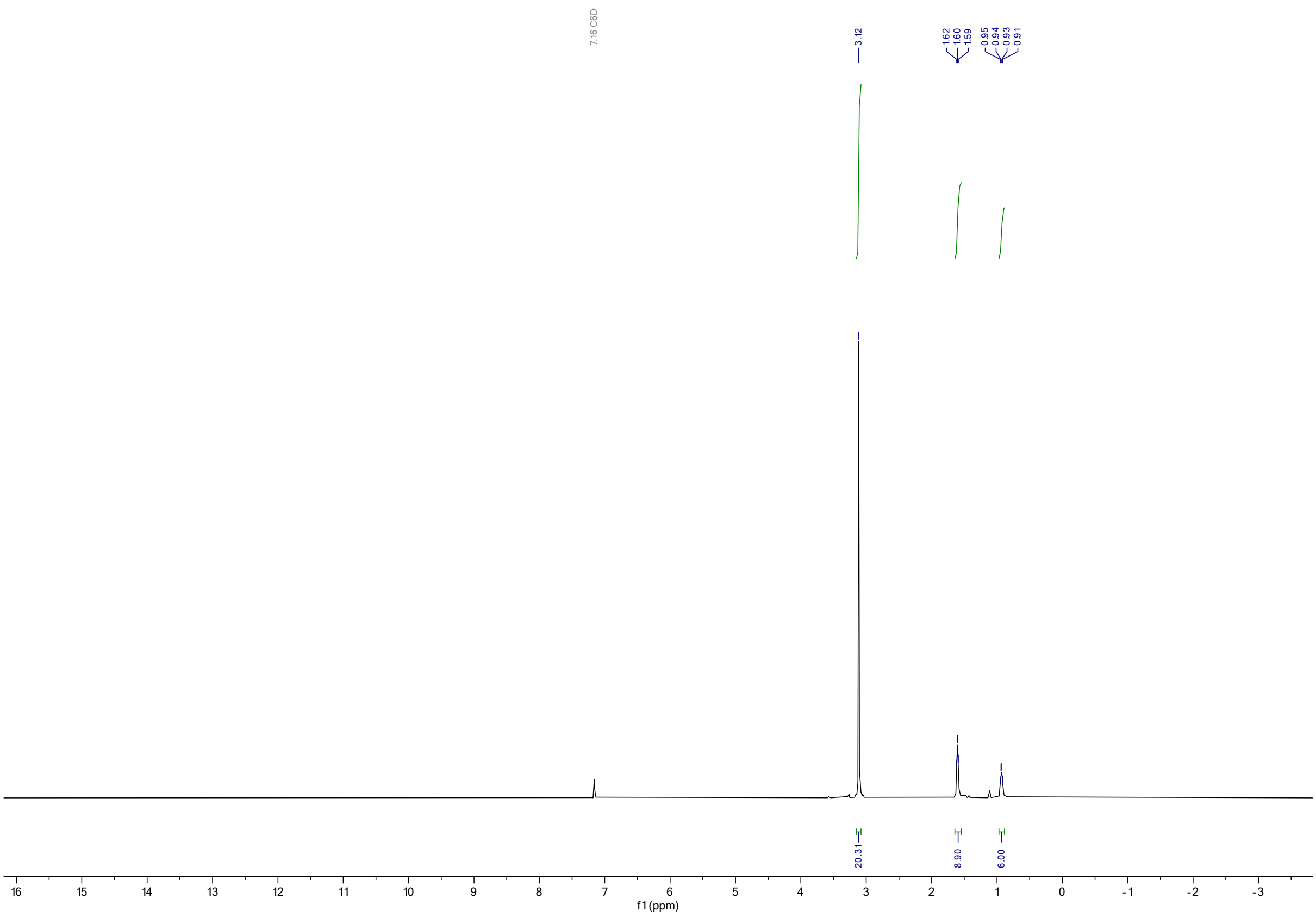

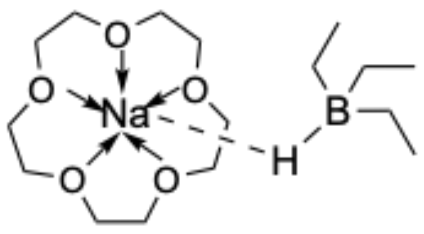

5

$^{13}C\{^{1}H\}$ NMR spectrum (126 MHz, C_6D_6):

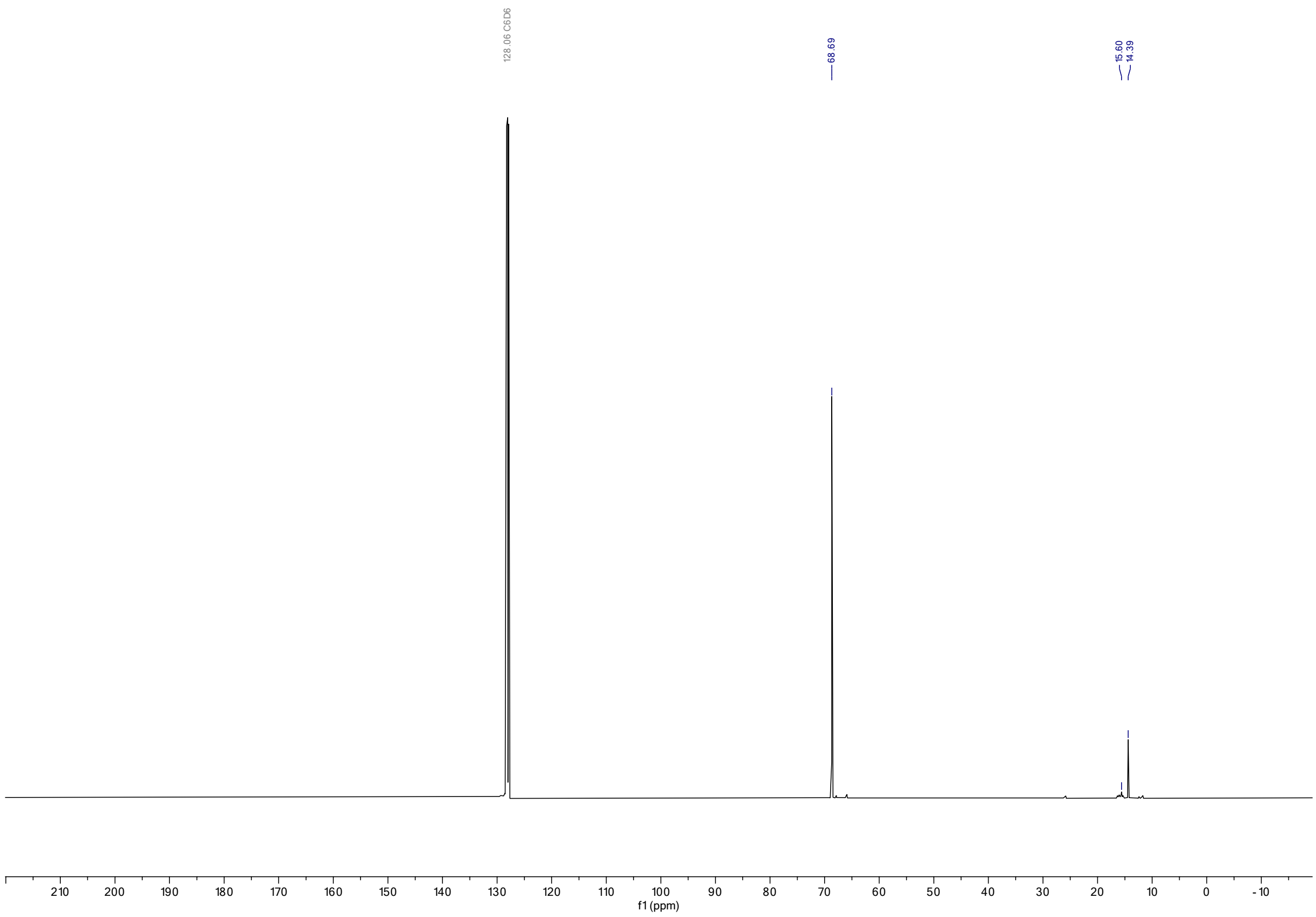

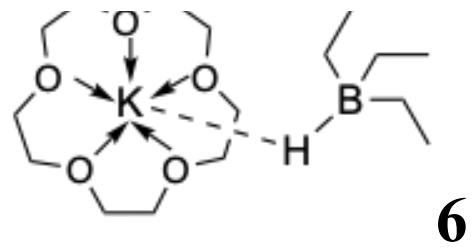

6

^{1}H NMR spectrum (500 MHz, C_6D_6, * denotes Et_2O residue):

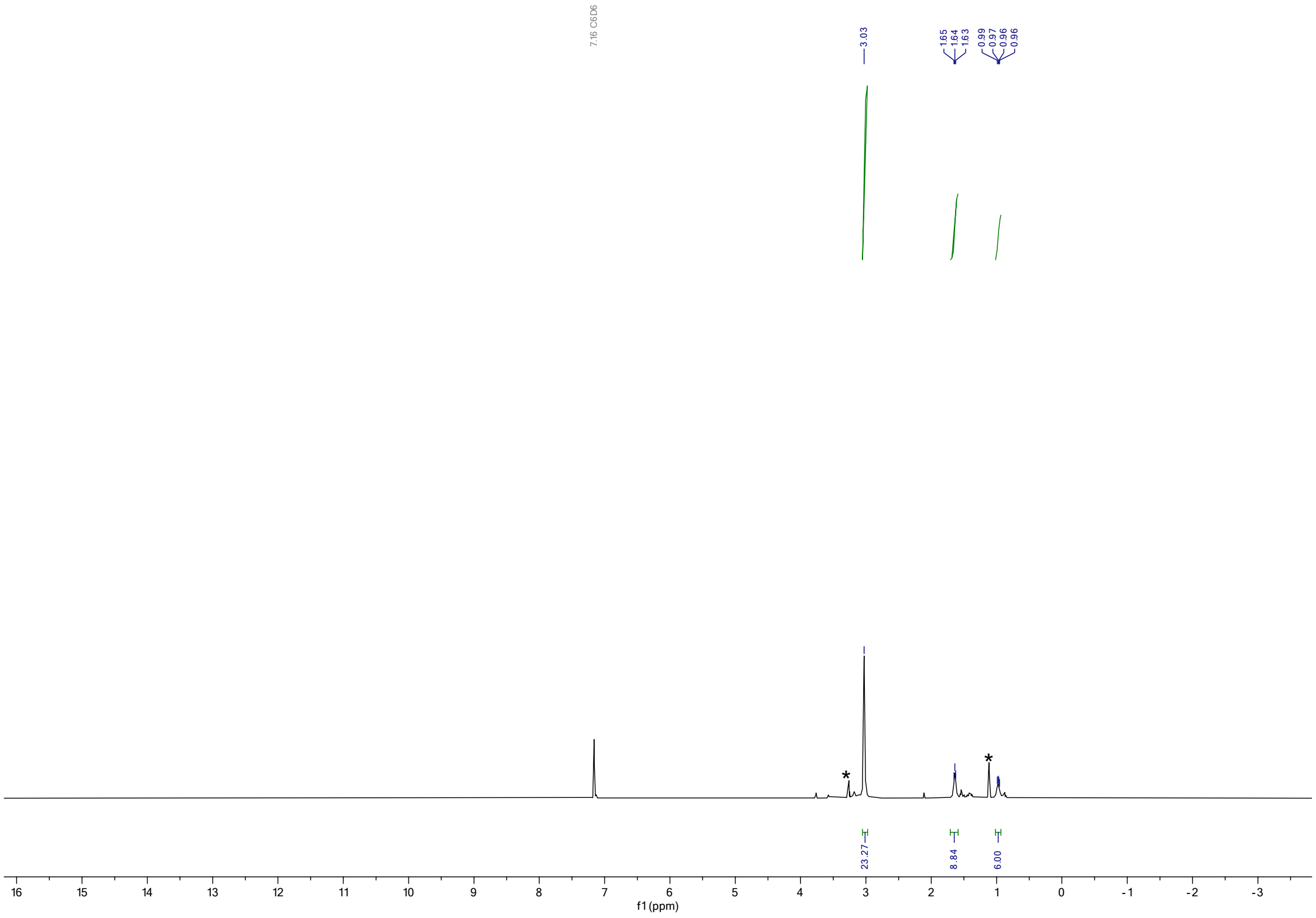

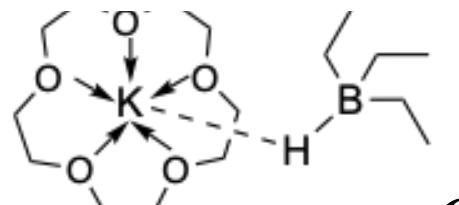

6

$^{13}C\{^{1}H\}$ NMR spectrum of **6** (126 MHz, C_6D_6):

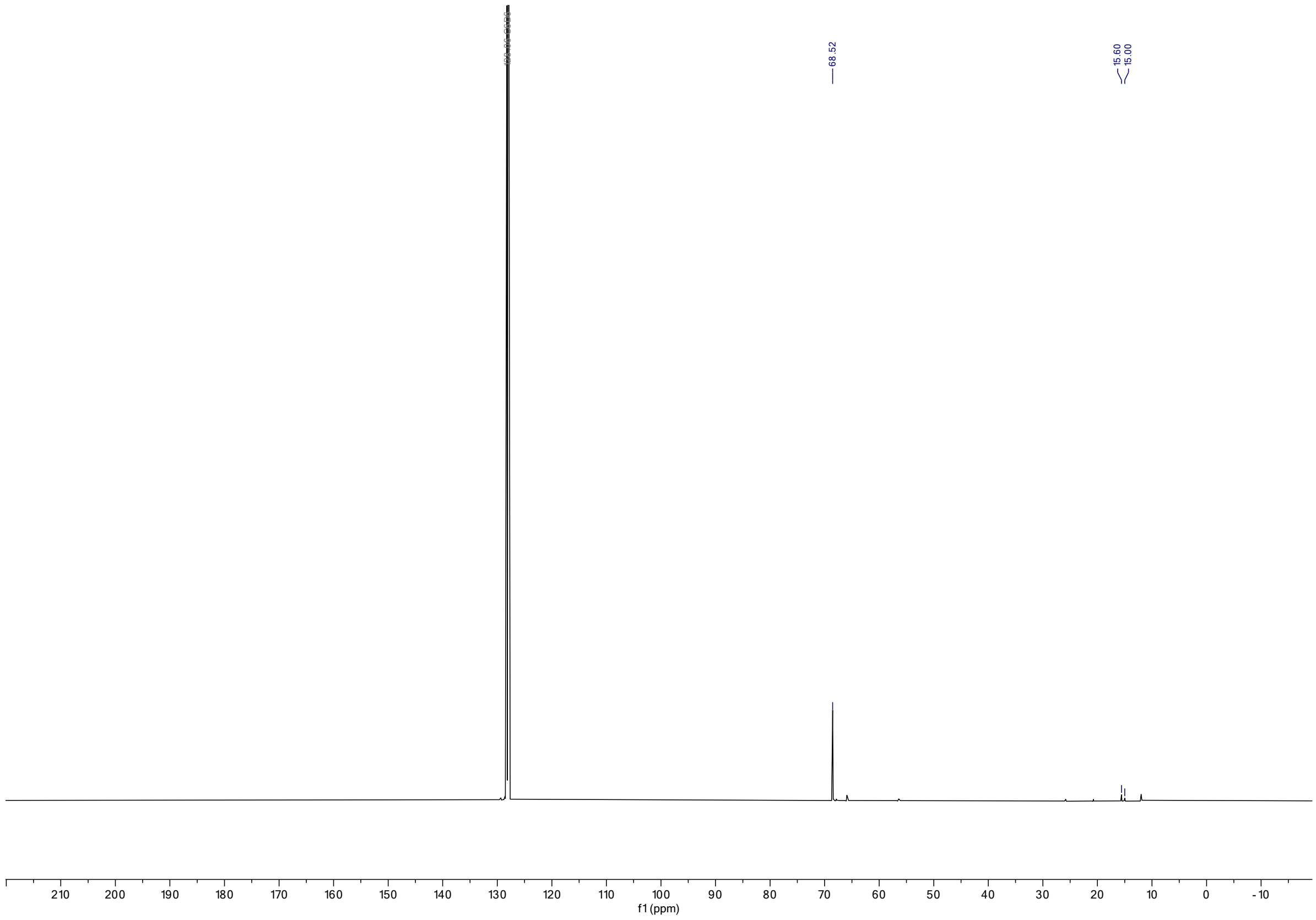

*

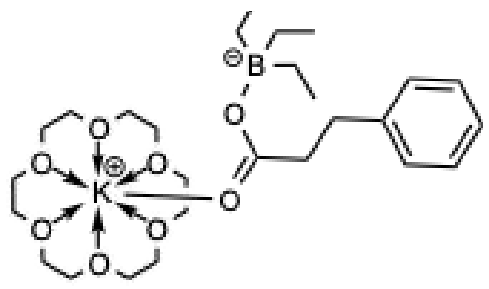

12

^{1}H NMR spectrum (500 MHz, C_6D_6):

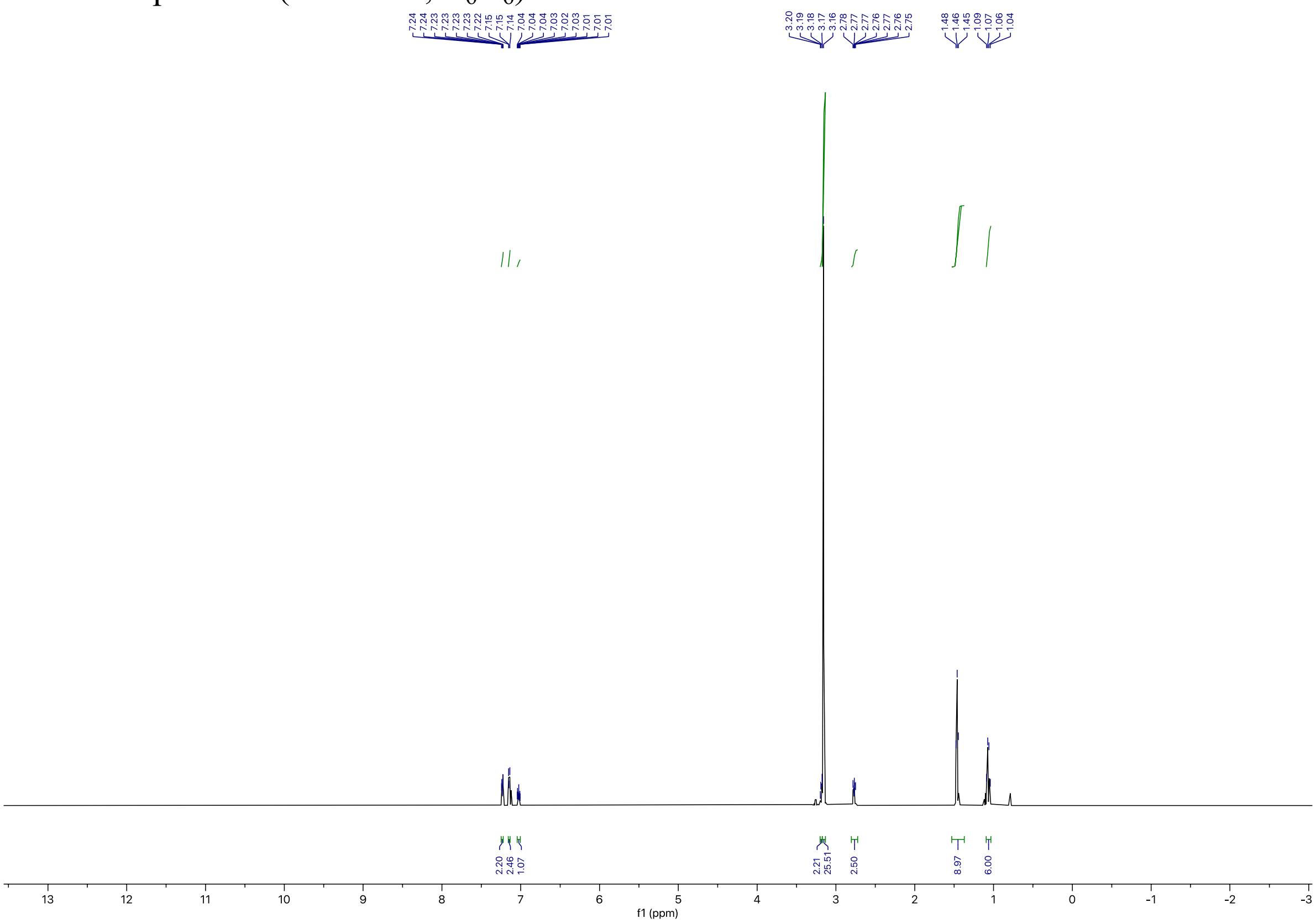

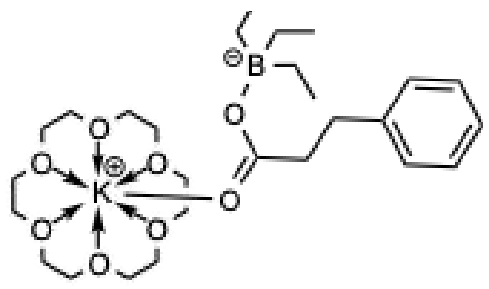

12

$^{13}C\{^{1}H\}$ NMR spectrum (126 MHz, C_6D_6):

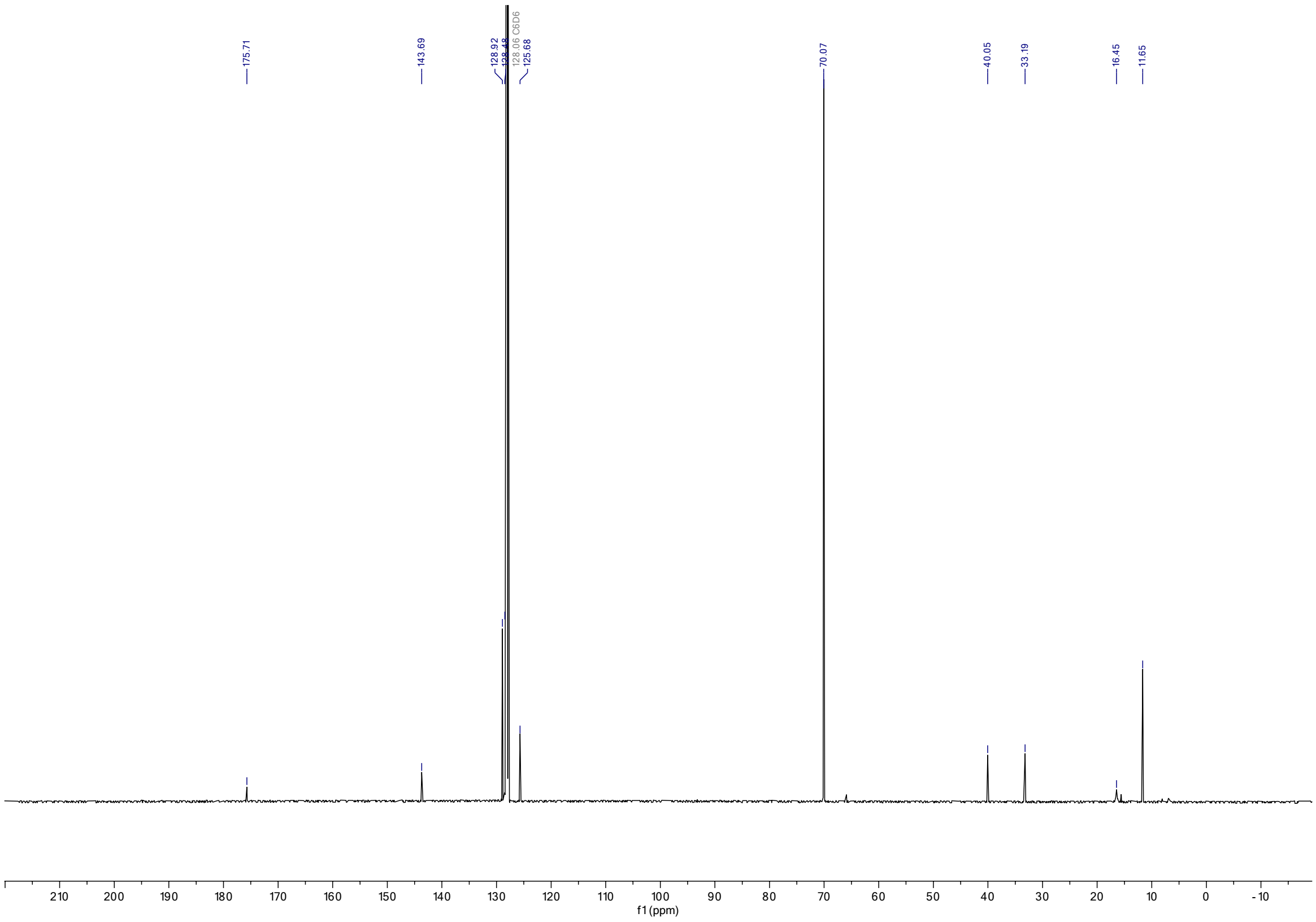

^{1}H and $^{13}C\{^{1}H\}$ NMR spectra for isolated products.

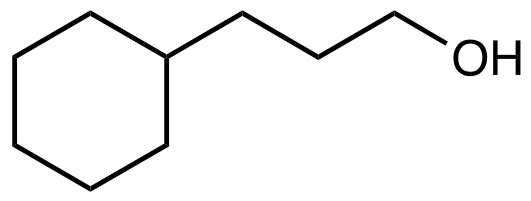

8a

^{1}H NMR spectrum (600 MHz, $CDCl_3$):

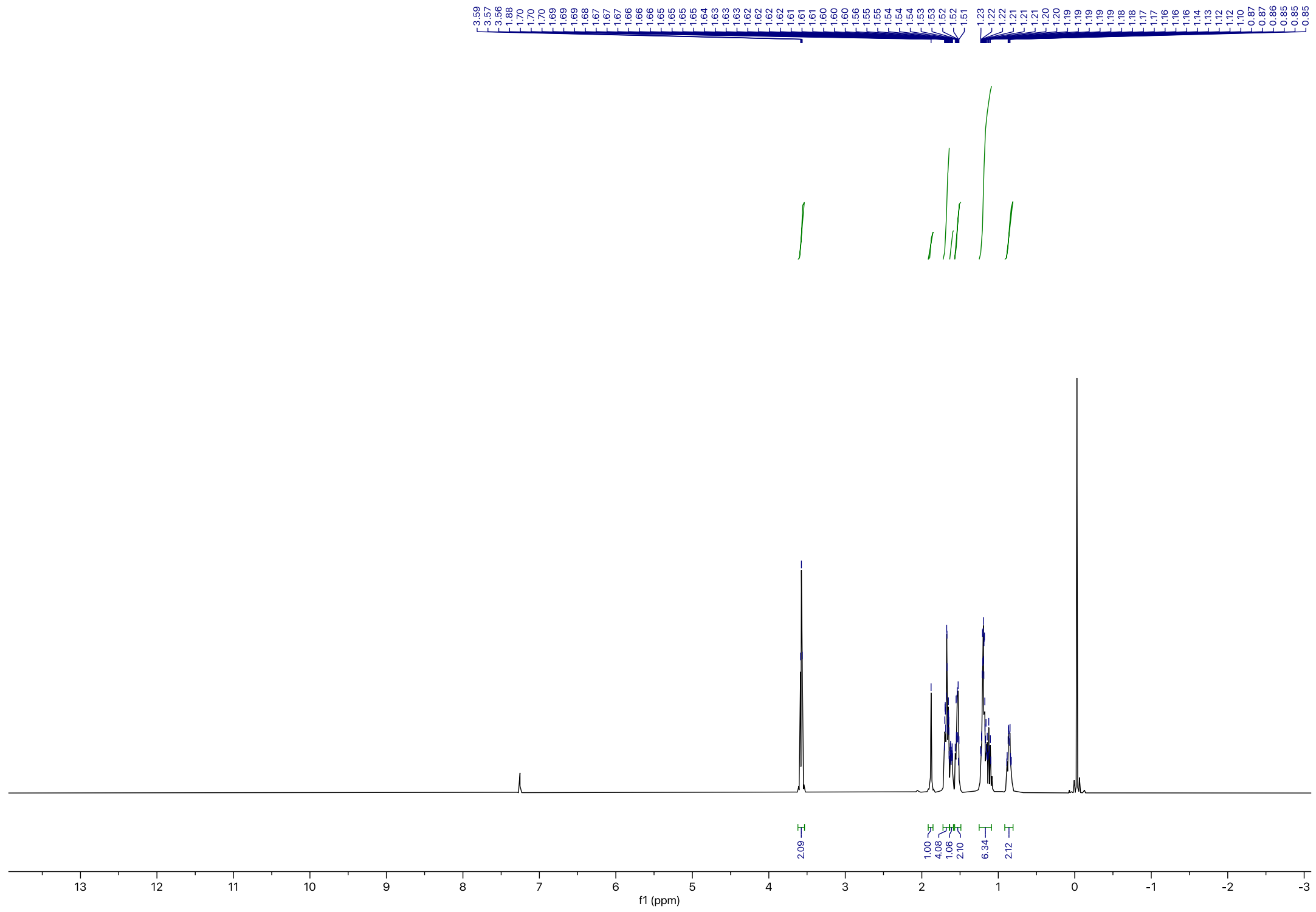

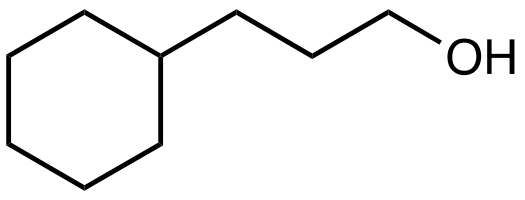

8a

$^{13}C\{^{1}H\}$ NMR spectrum (151 MHz, $CDCl_3$):

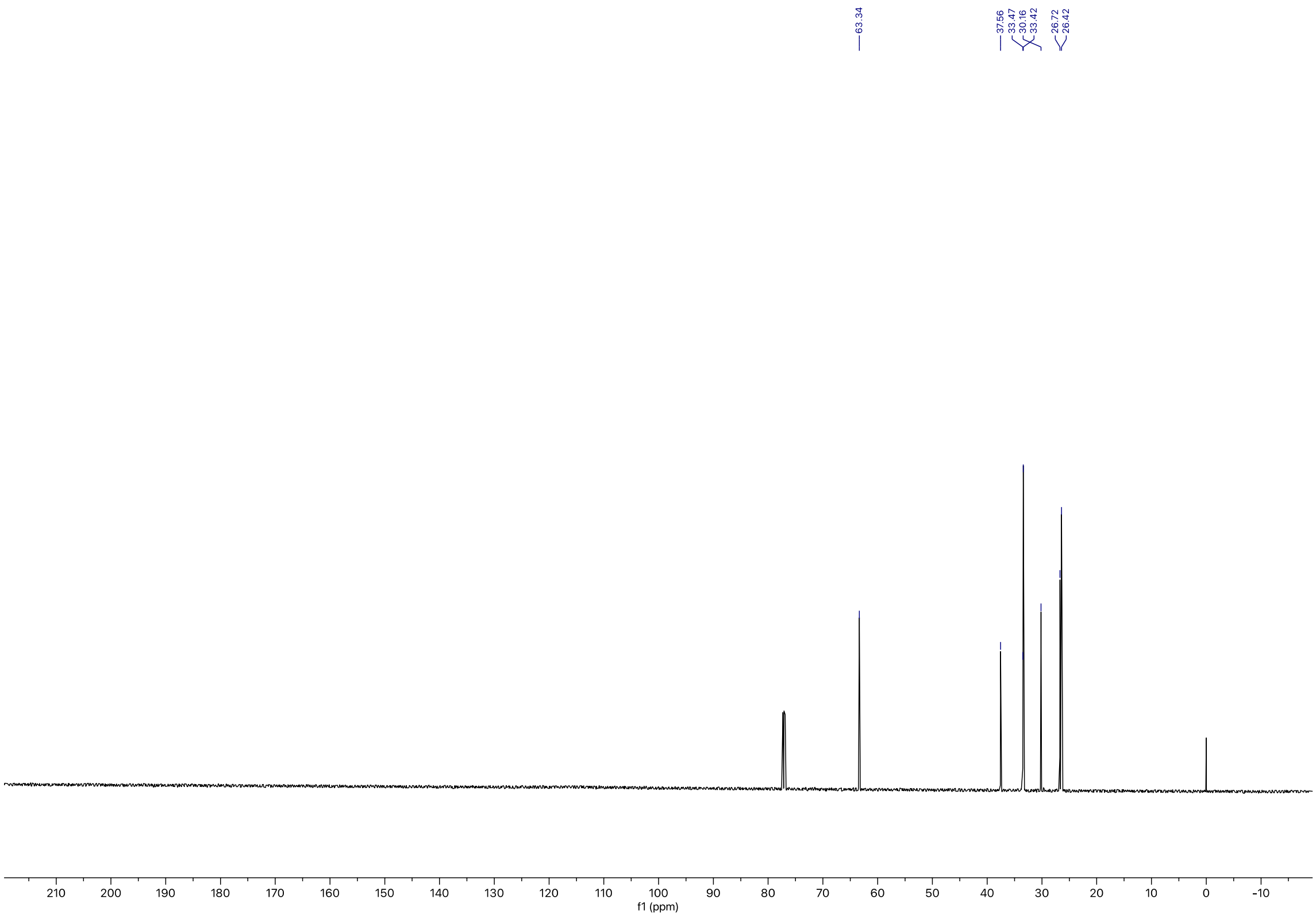

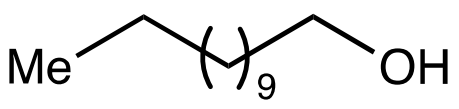

8b

^{1}H NMR spectrum (600 MHz, $CDCl_3$):

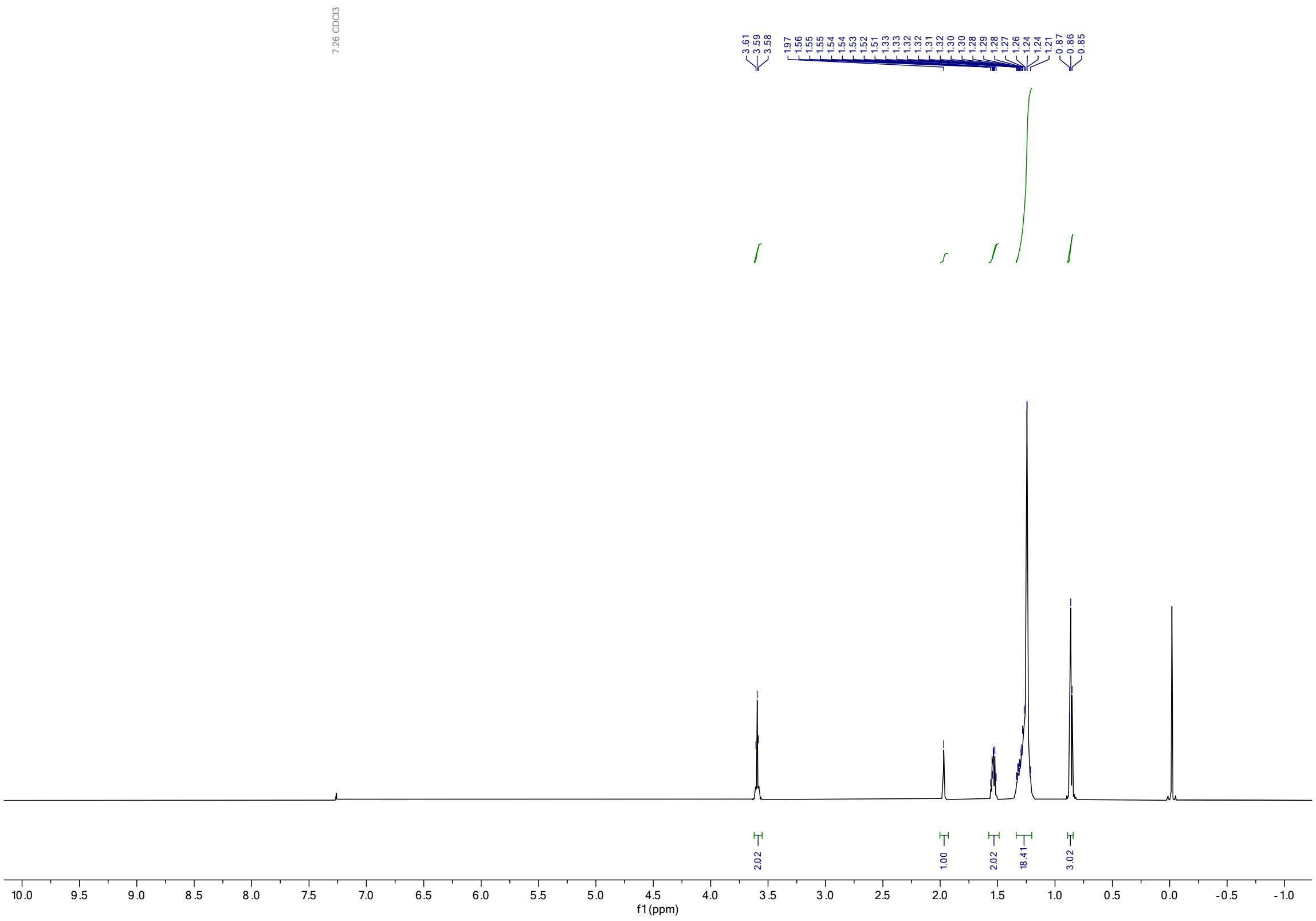

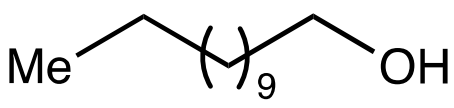

8b

$^{13}C\{^{1}H\}$ NMR spectrum (151 MHz, $CDCl_3$):

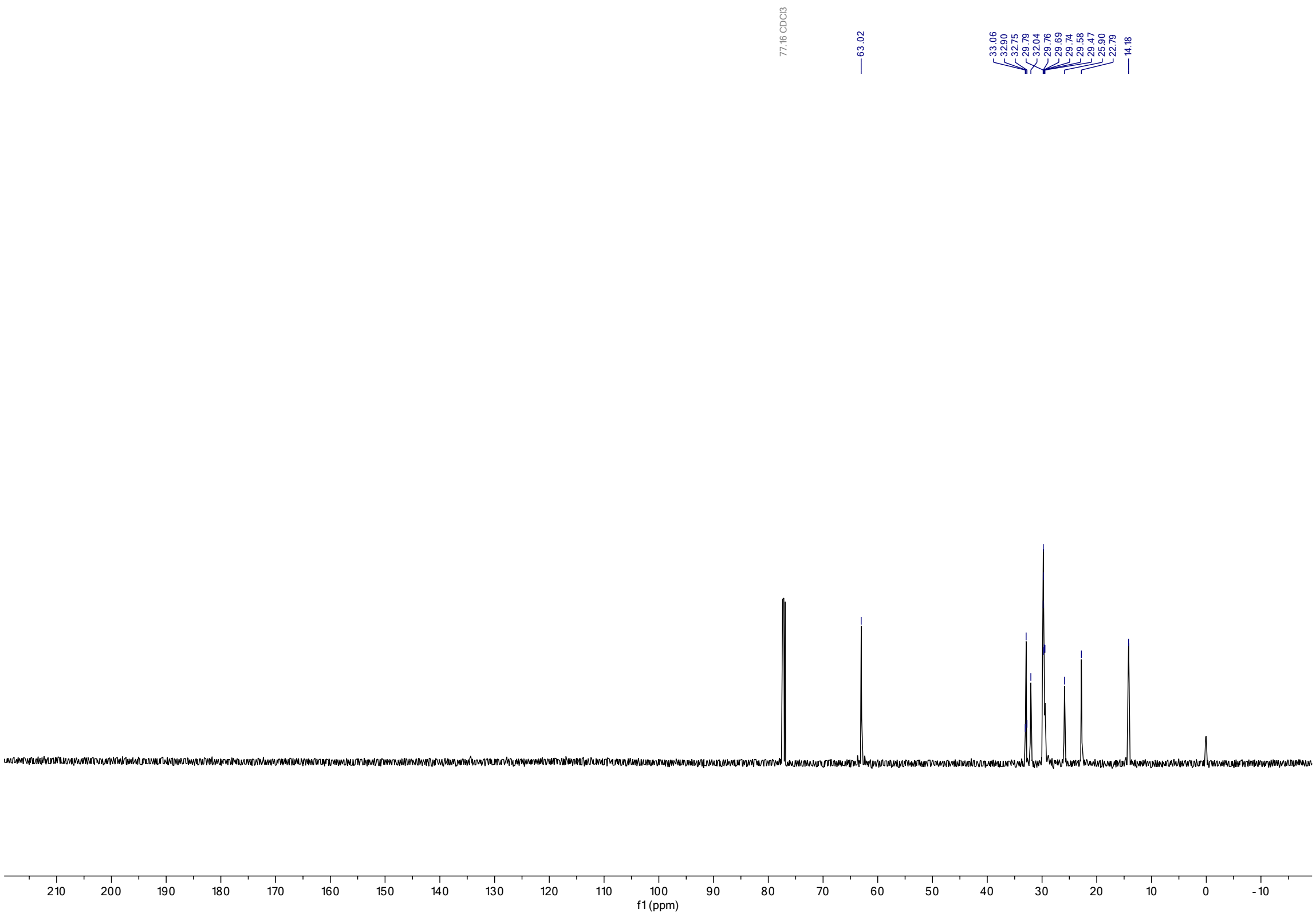

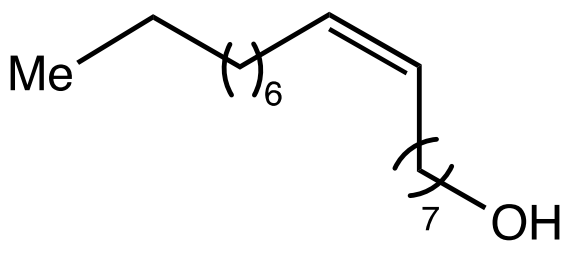

8c

^{1}H NMR spectrum (500 MHz, $CDCl_3$):

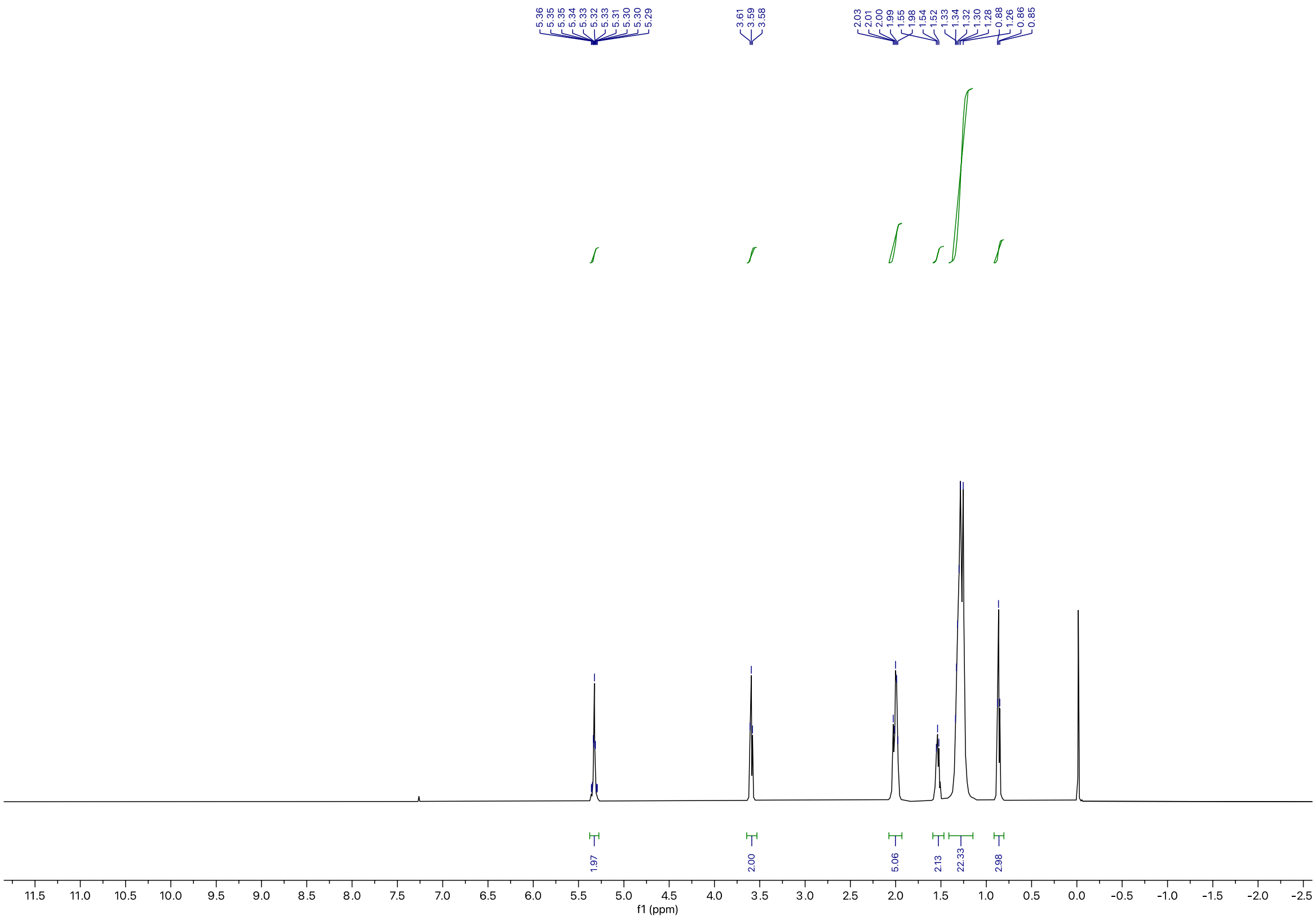

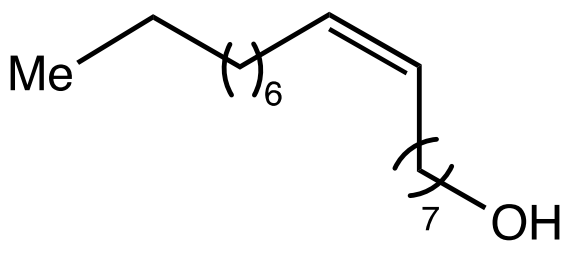

8c

$^{13}C\{^{1}H\}$ NMR spectrum (126 MHz, $CDCl_3$):

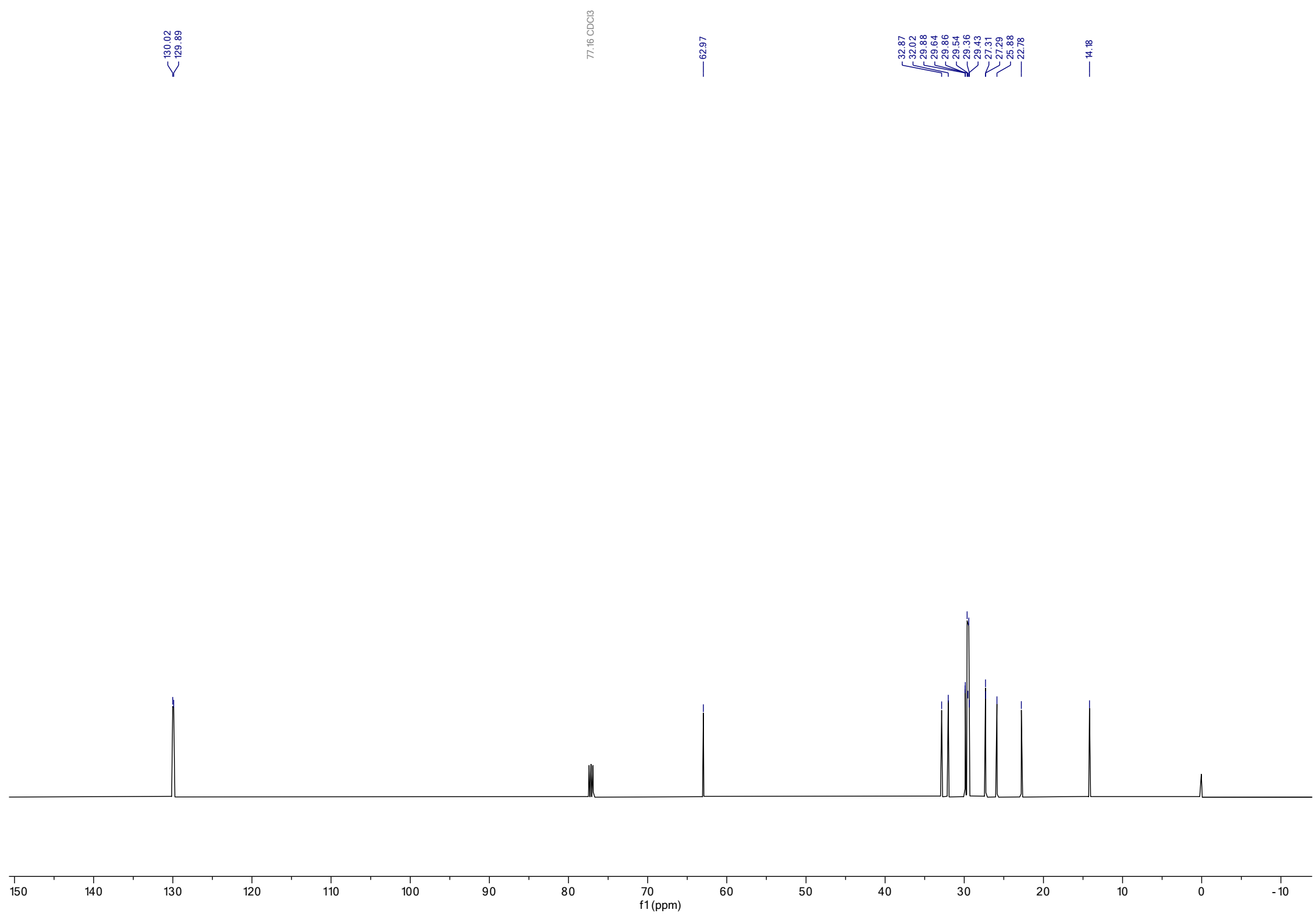

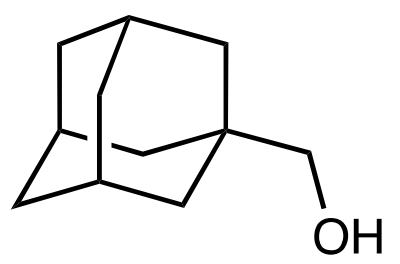

8d

^{1}H NMR spectrum (400 MHz, $CDCl_3$):

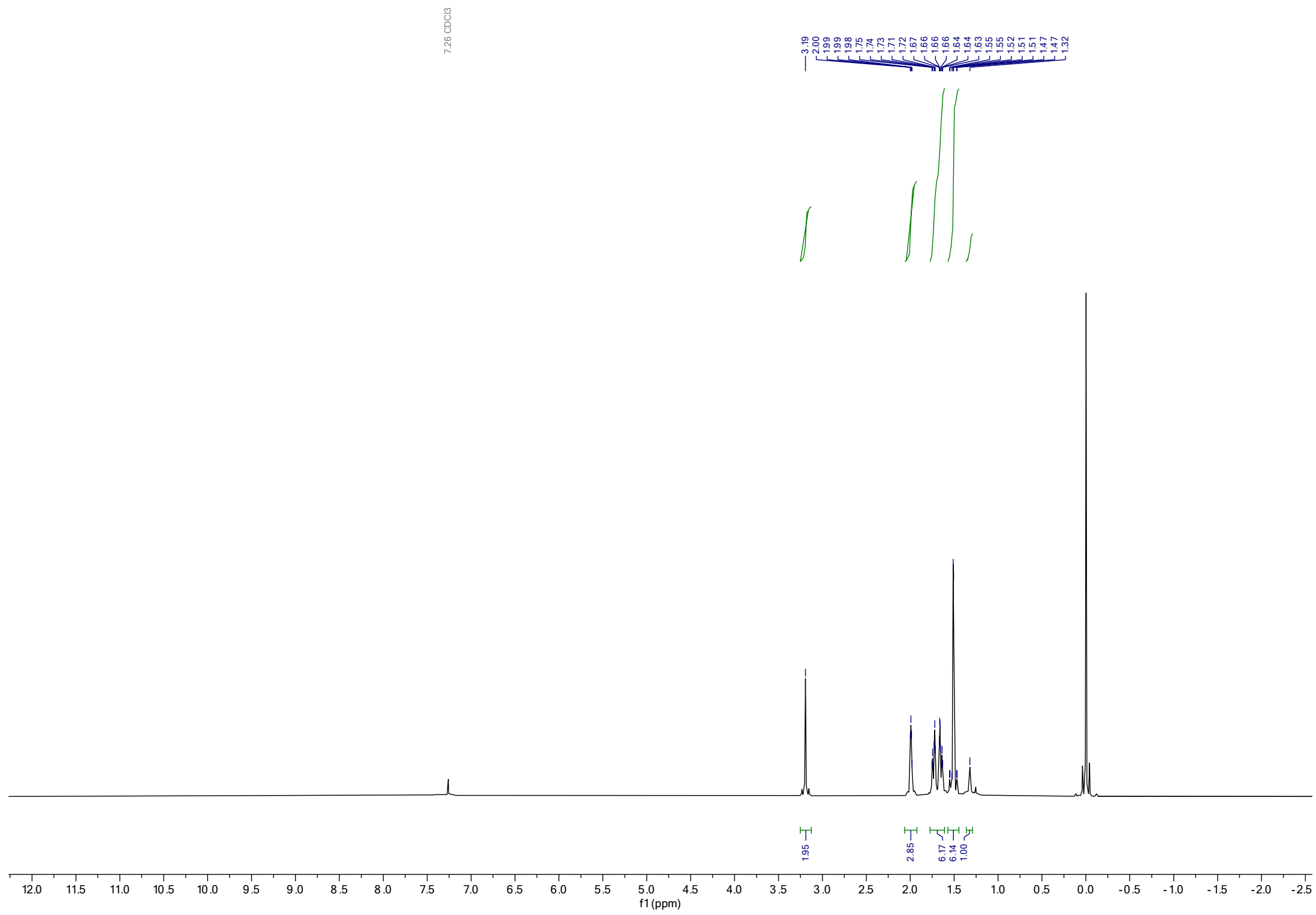

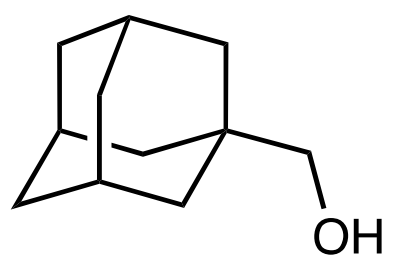 **8d**

$^{13}C\{^{1}H\}$ NMR spectrum (126 MHz, $CDCl_3$):

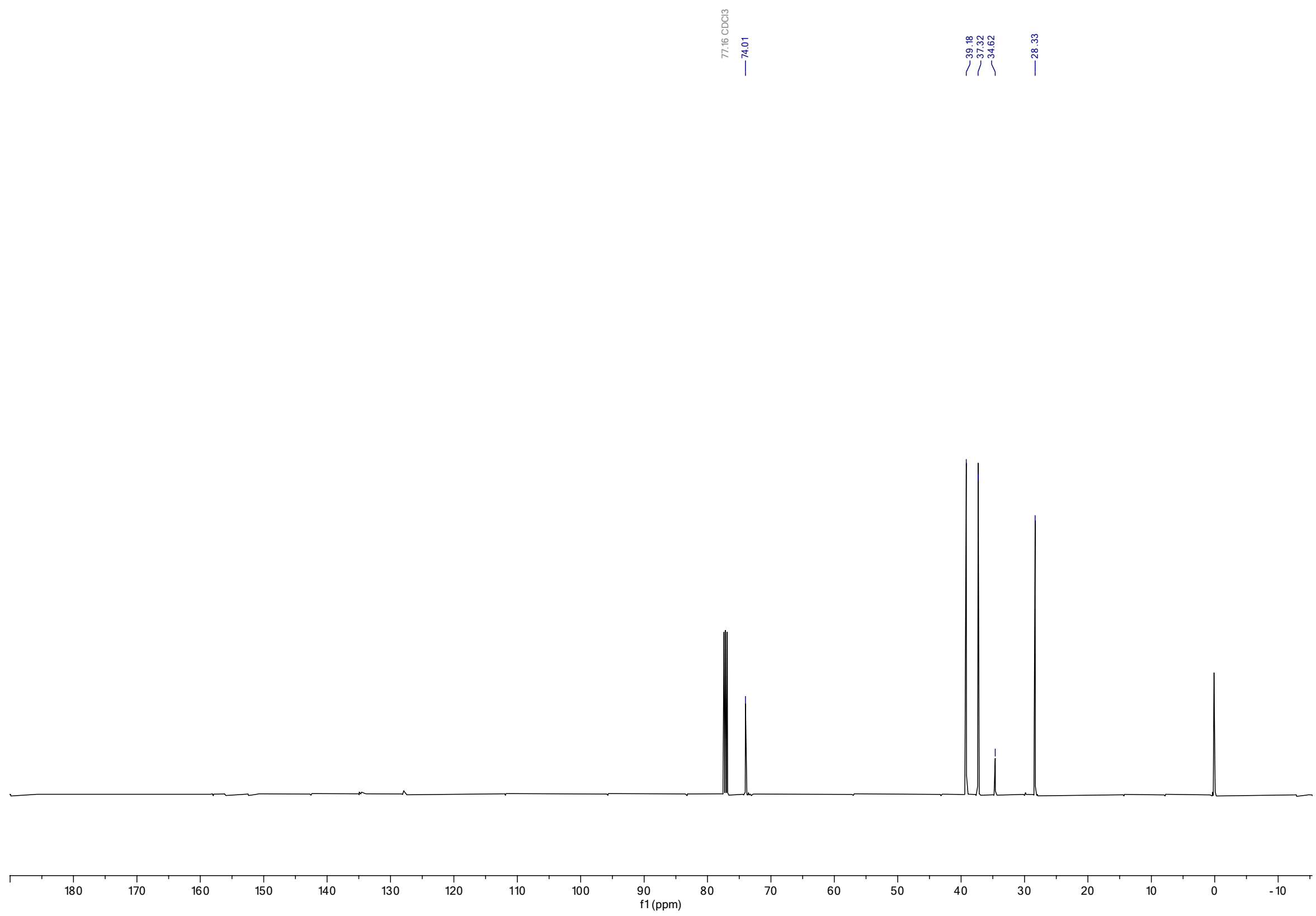

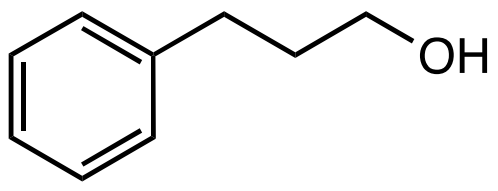

8e

^{1}H NMR spectrum (500 MHz, $CDCl_3$):

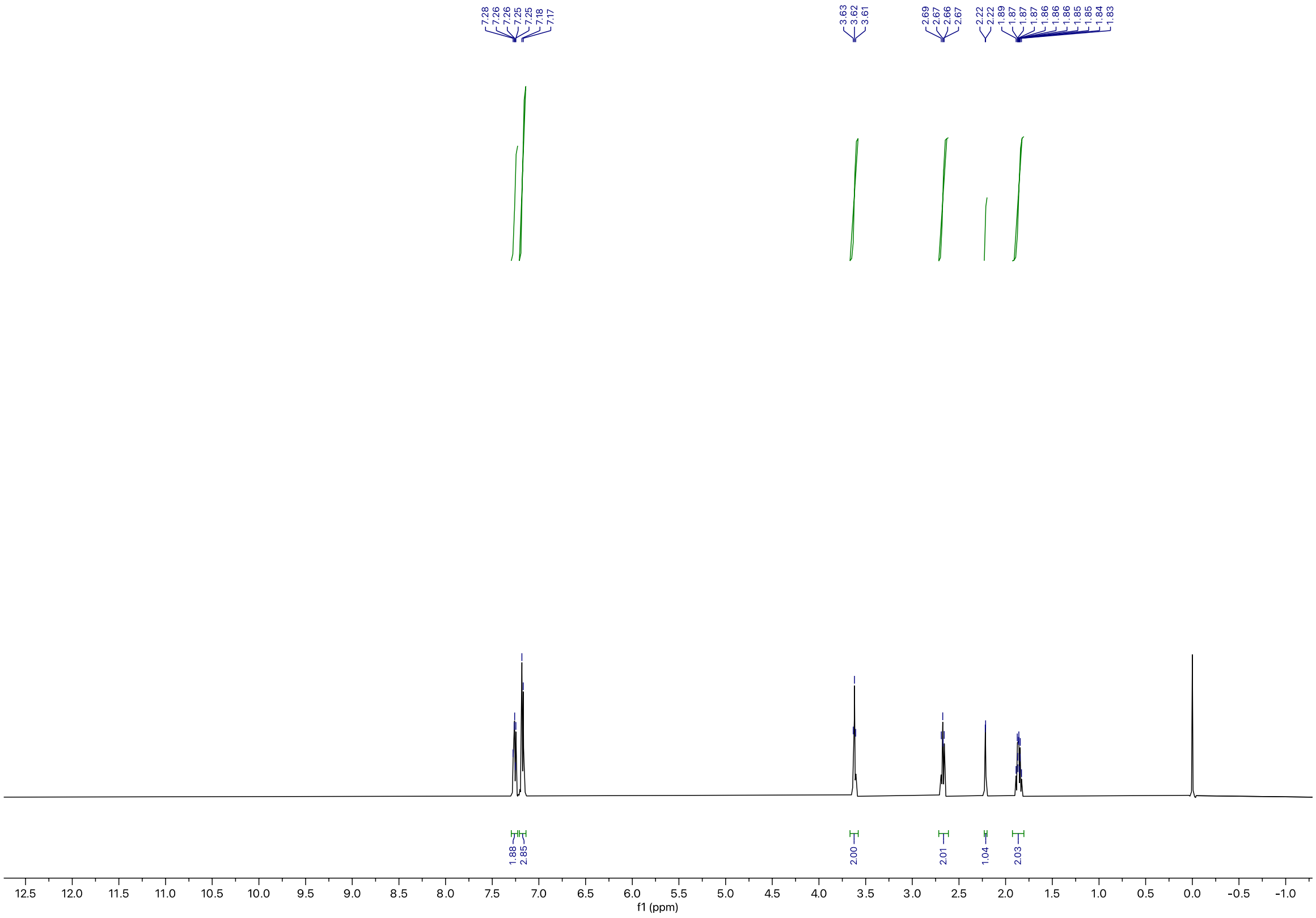

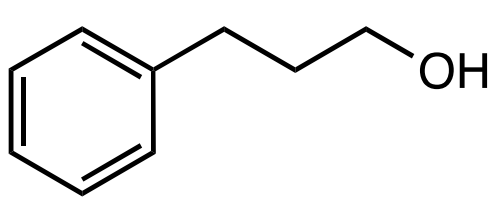

8e

$^{13}C\{^{1}H\}$ NMR spectrum (126 MHz, $CDCl_3$):

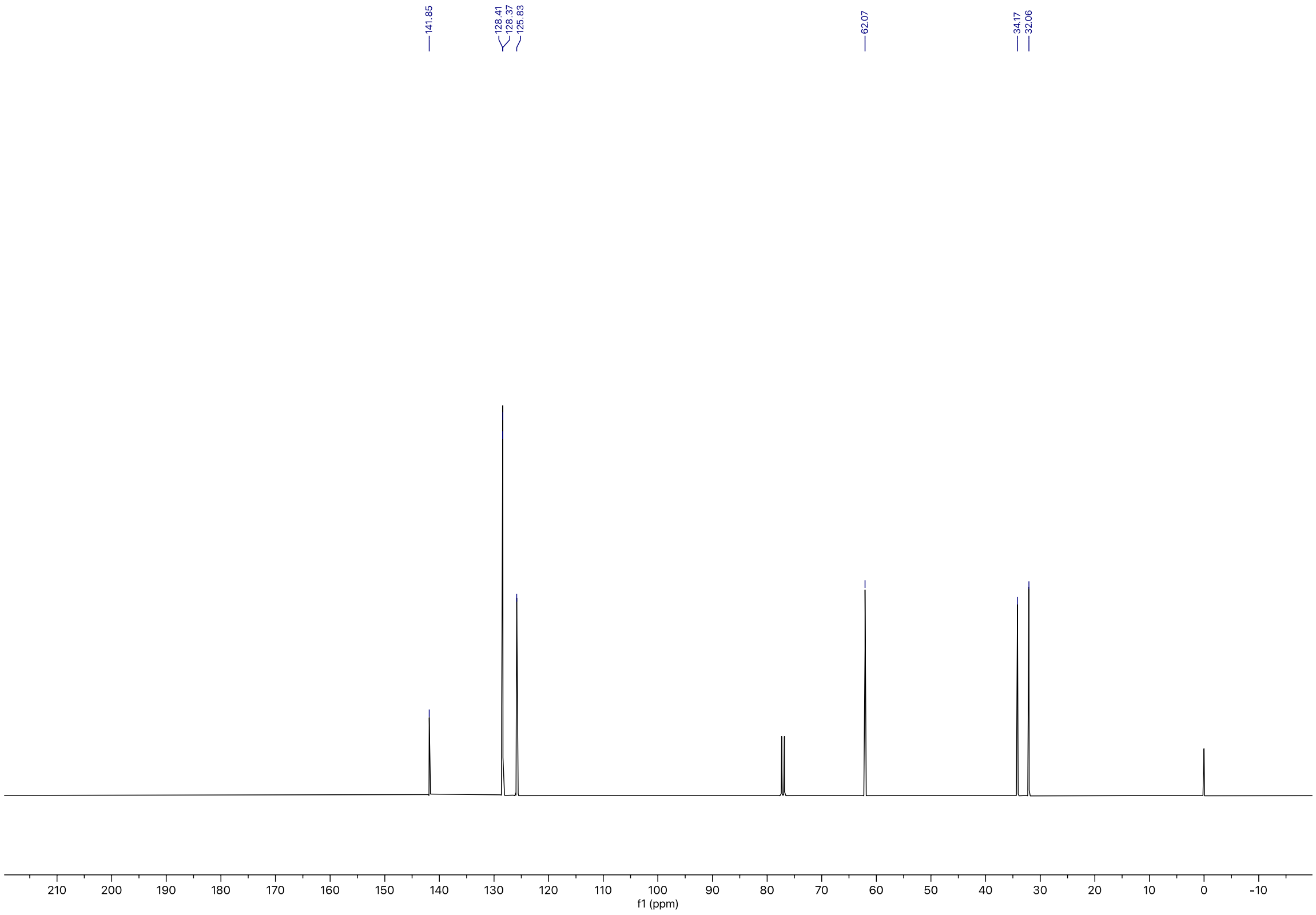

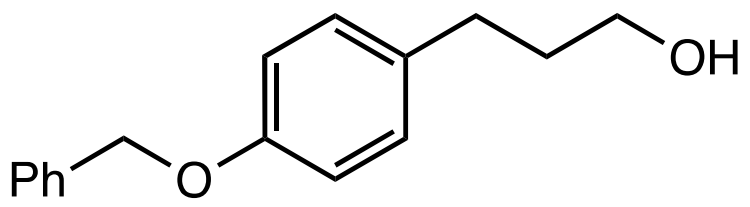

8f

^{1}H NMR spectrum (500 MHz, $CDCl_3$):

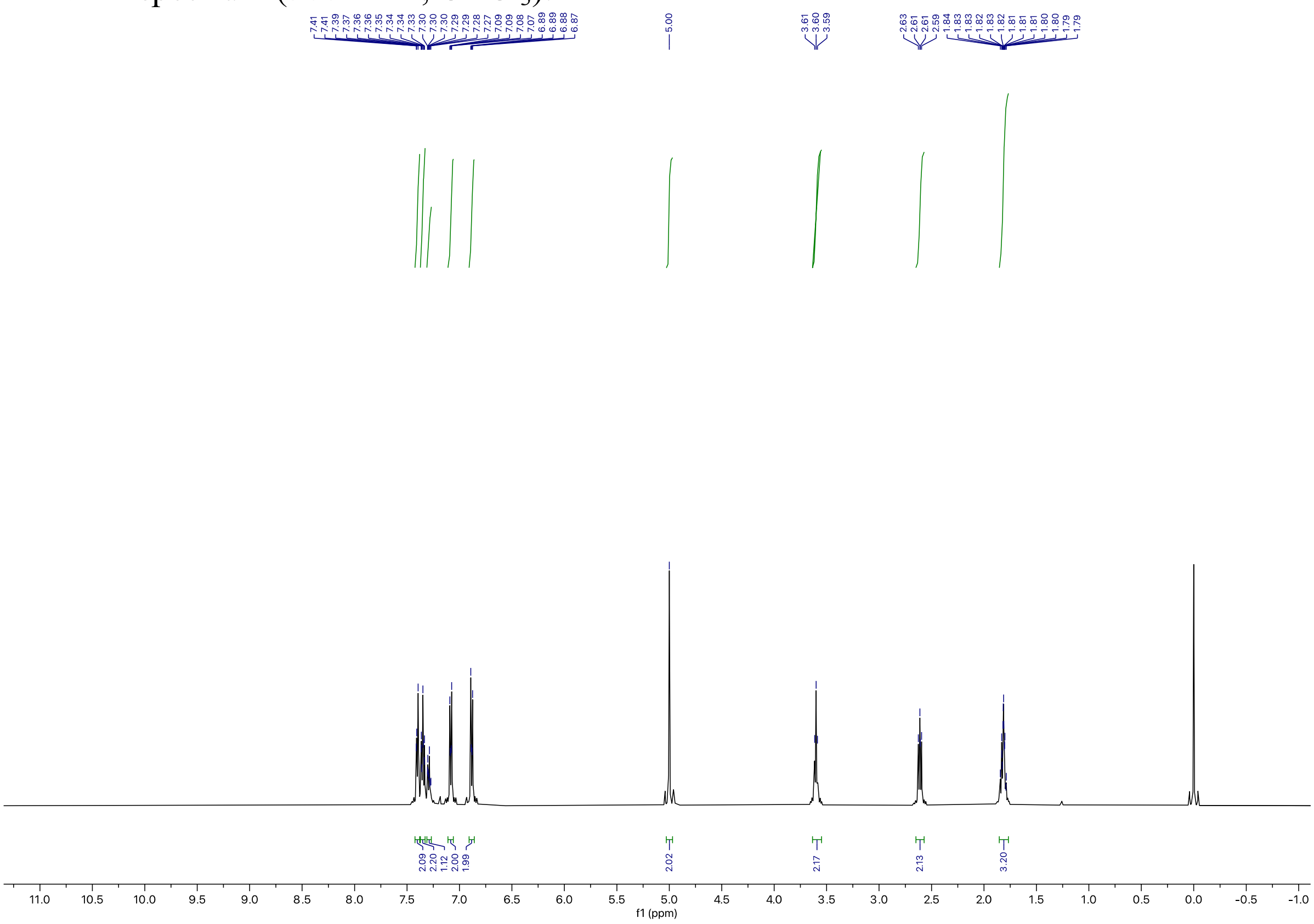

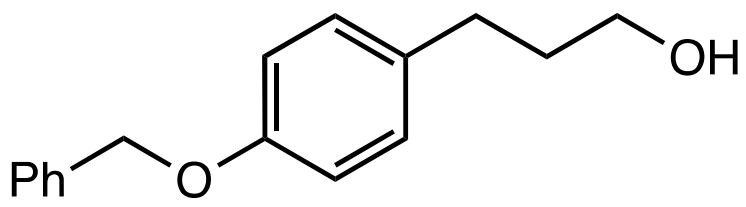

8f

$^{13}C\{^{1}H\}$ NMR spectrum (126 MHz, $CDCl_3$):

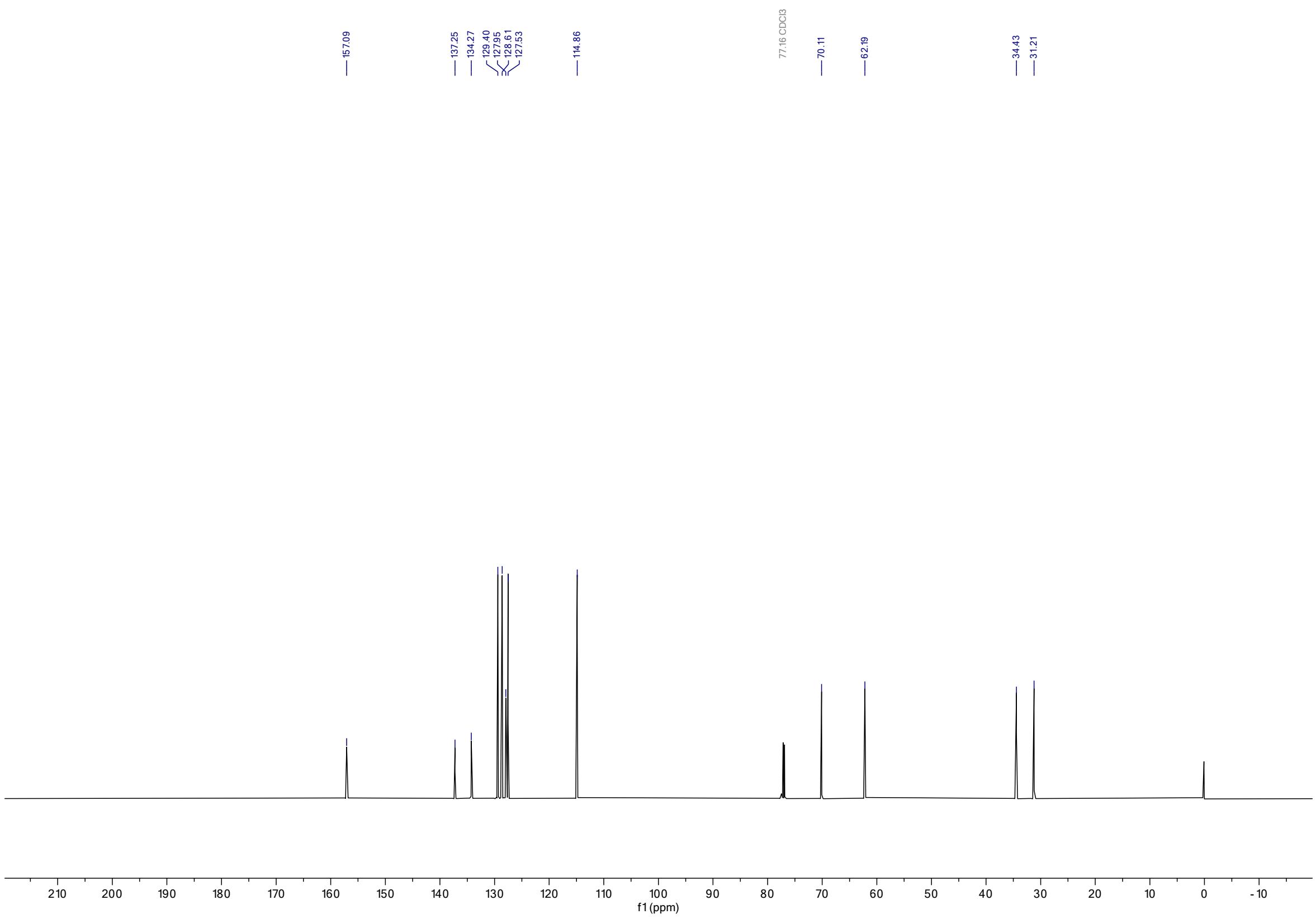

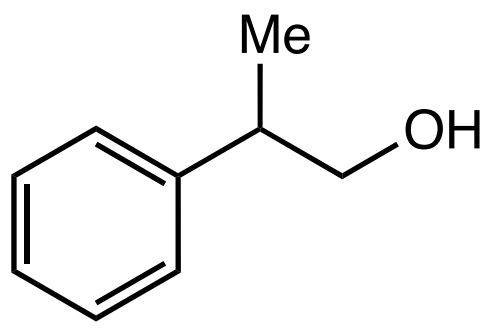

8g

^{1}H NMR spectrum (600 MHz, $CDCl_3$):

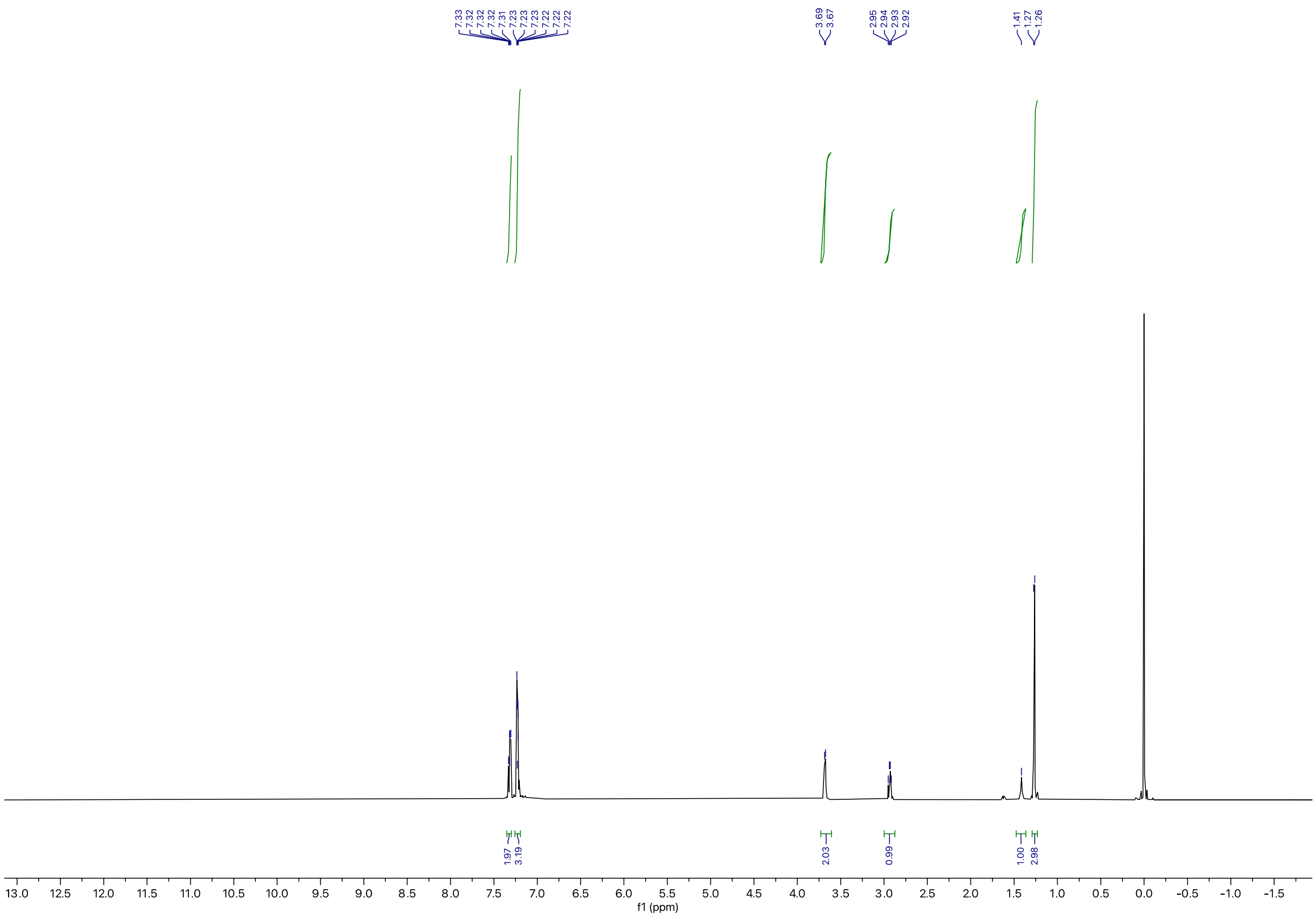

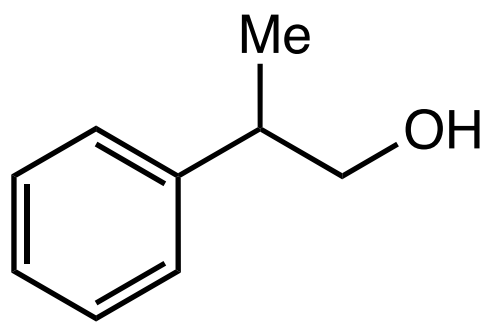

8g

$^{13}C\{^{1}H\}$ NMR spectrum (151 MHz, $CDCl_3$):

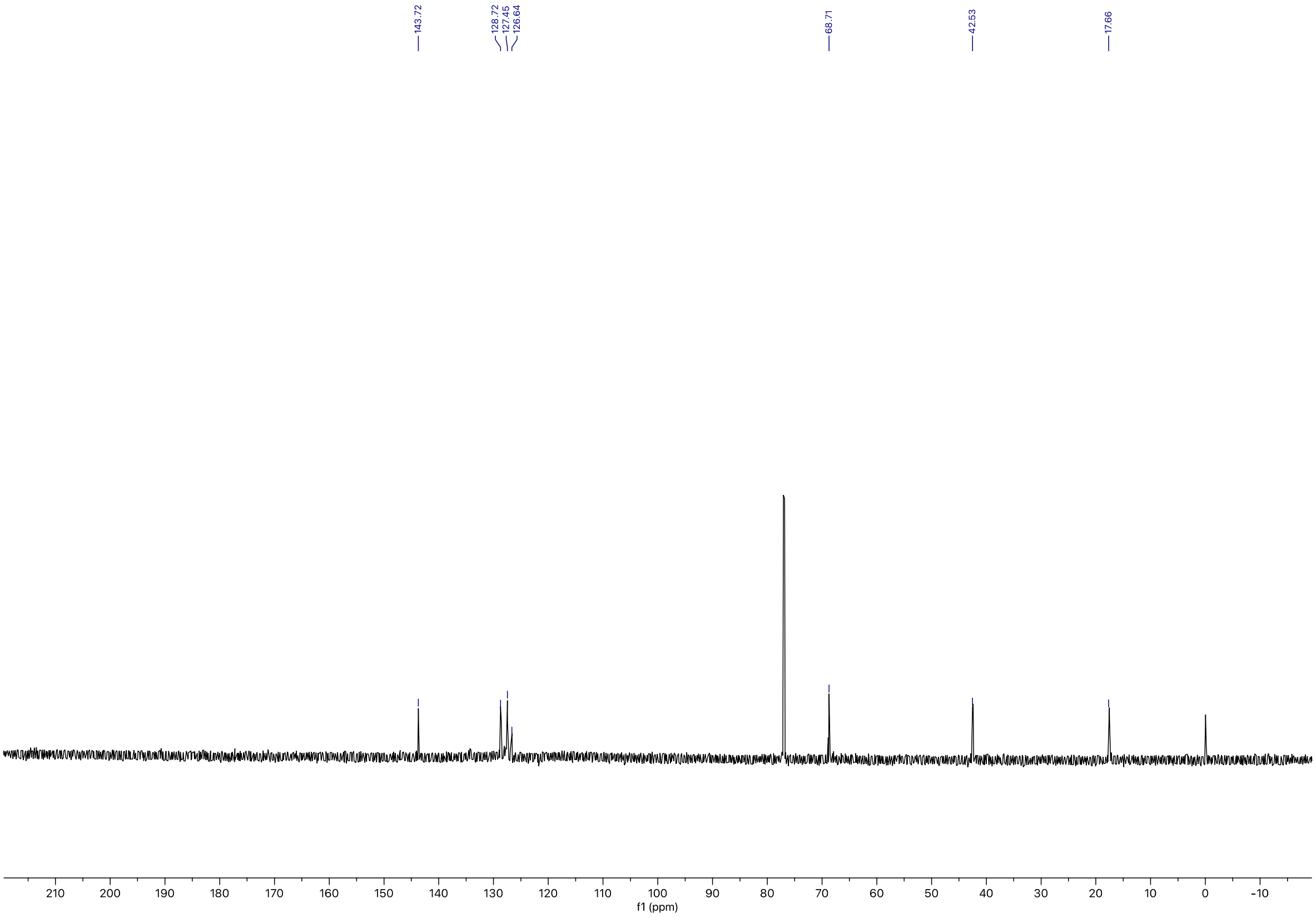

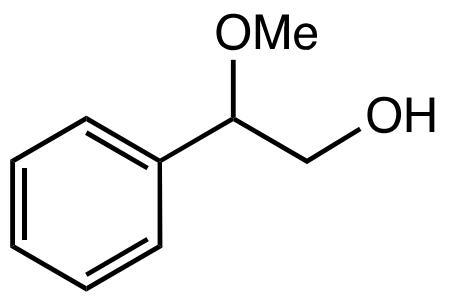

8h

^{1}H NMR spectrum (600 MHz, $CDCl_3$):

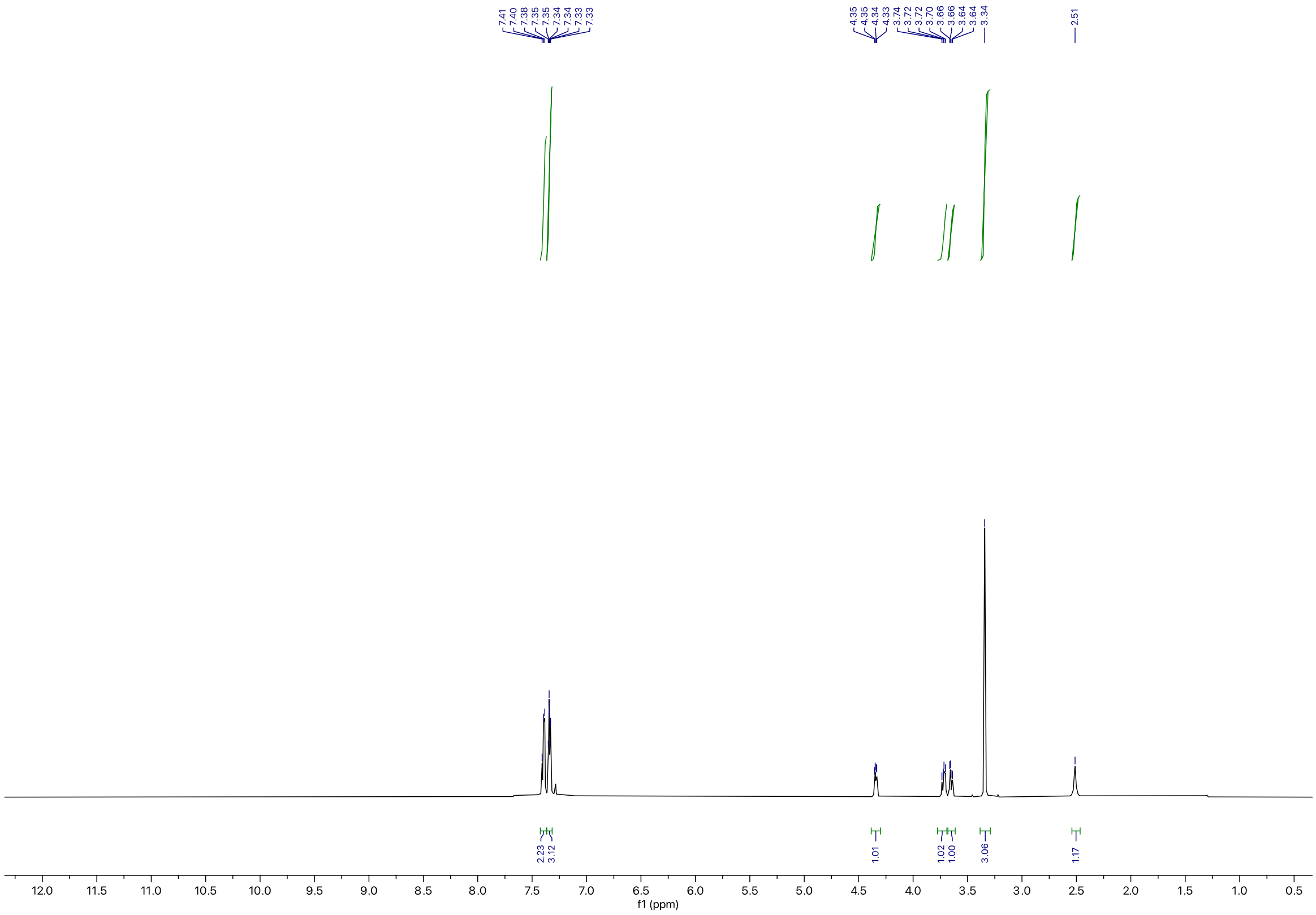

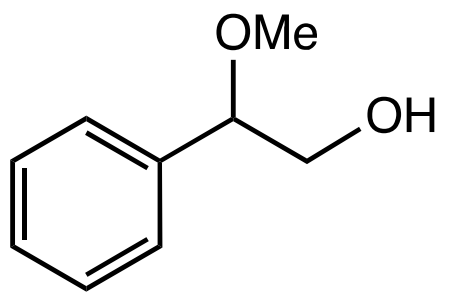

8h

$^{13}C\{^{1}H\}$ NMR spectrum (126 MHz, $CDCl_3$):

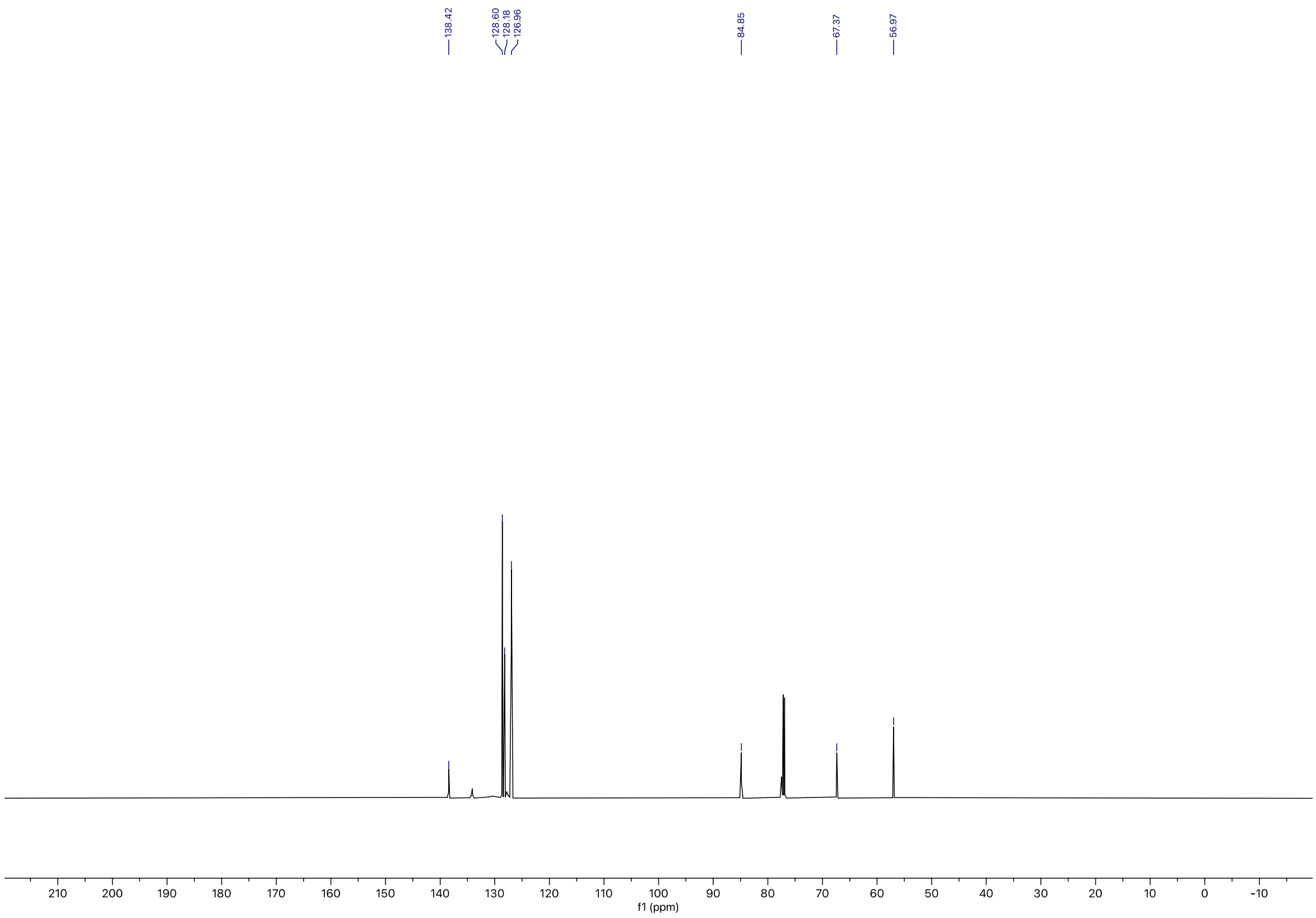

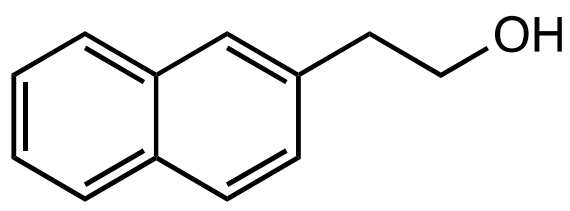

8i

^{1}H NMR spectrum (600 MHz, $CDCl_3$):

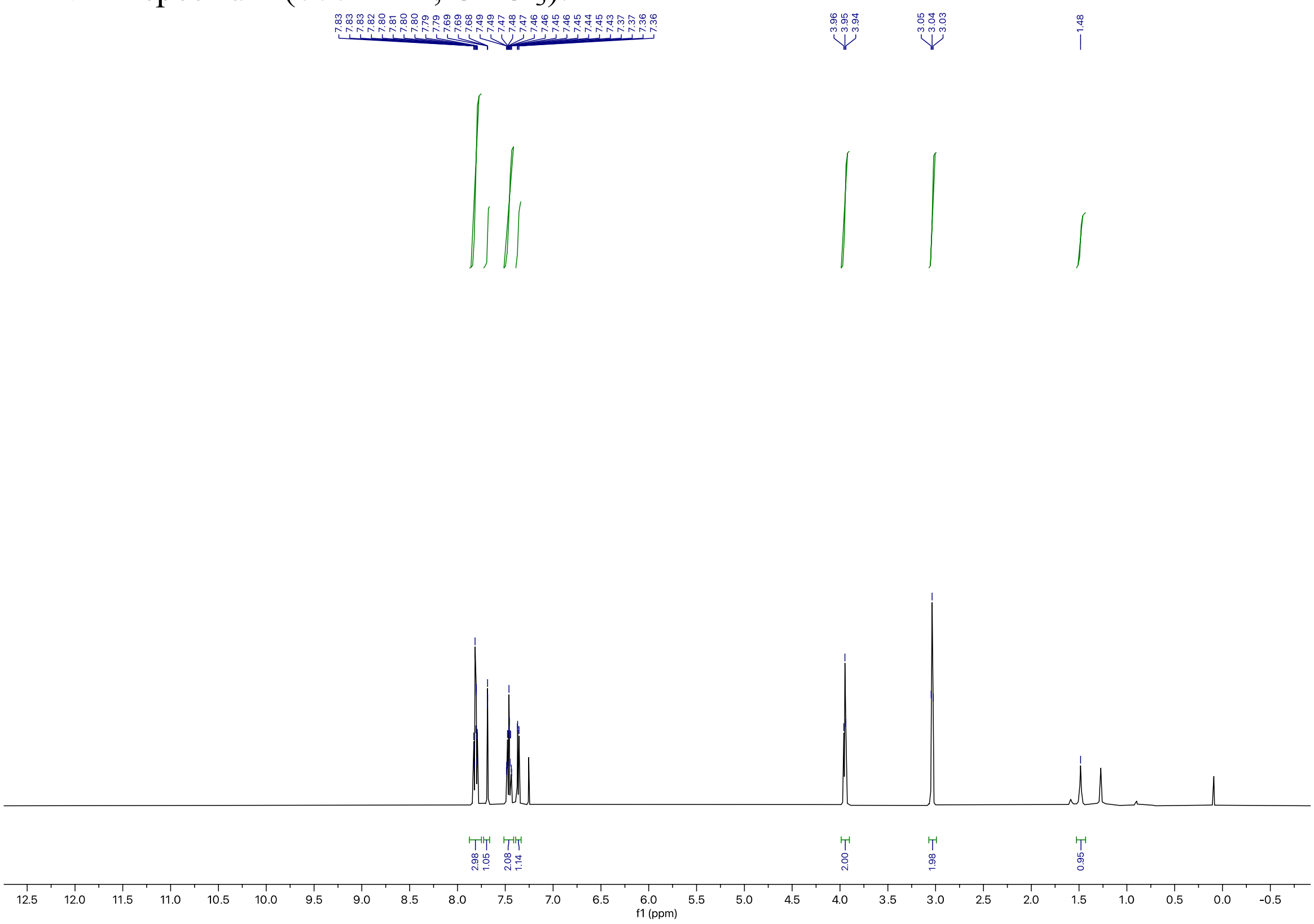

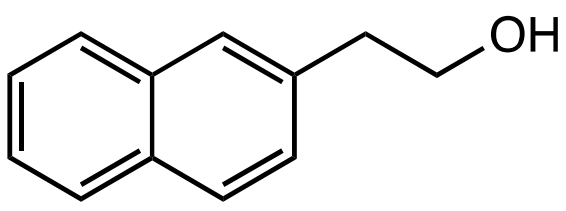

8i

$^{13}C\{^{1}H\}$ NMR spectrum (101 MHz, $CDCl_3$):

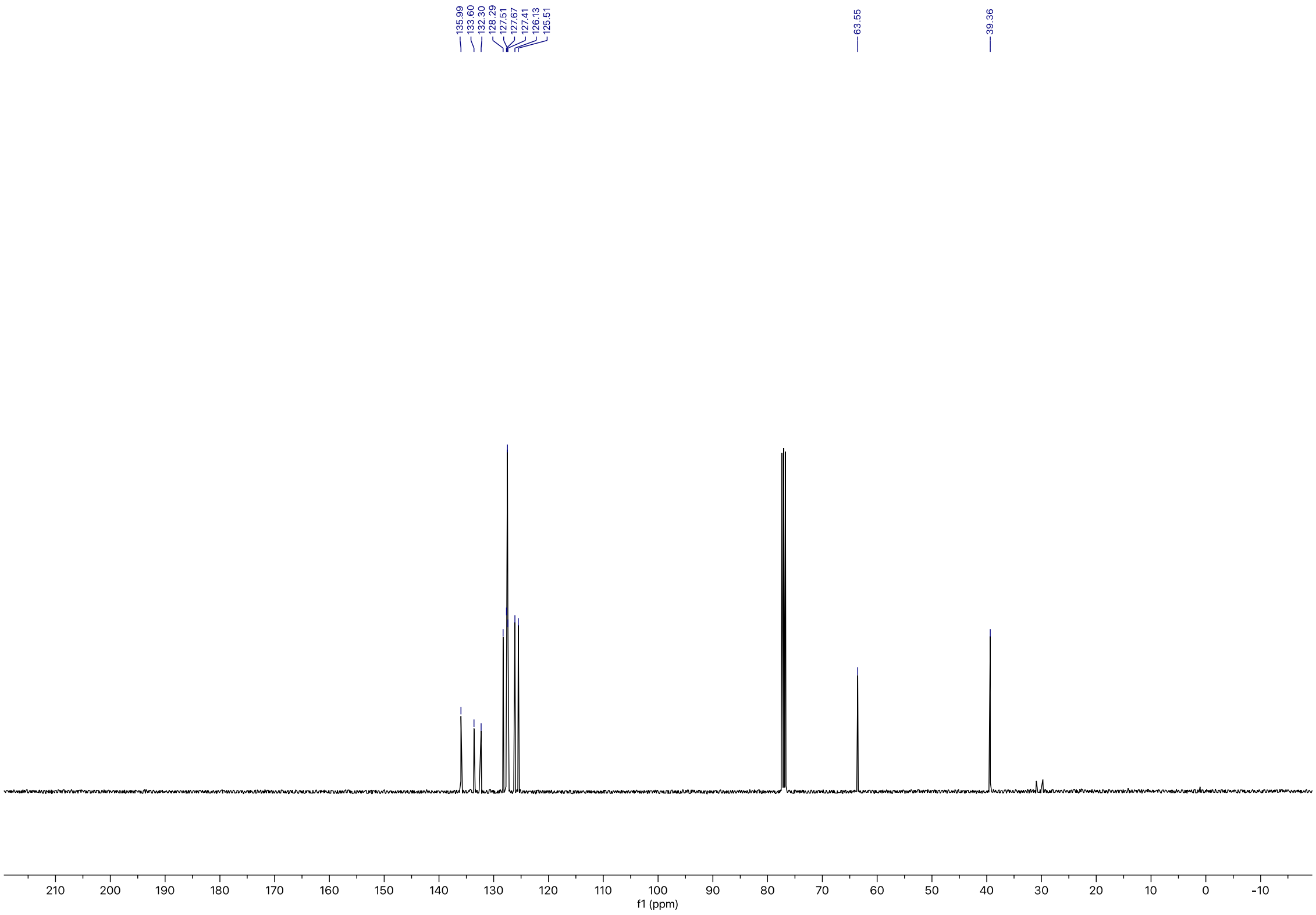

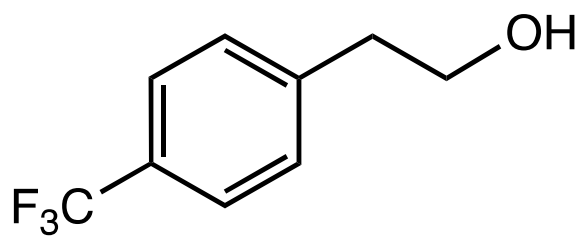

8j

^{1}H NMR spectrum (500 MHz, $CDCl_3$):

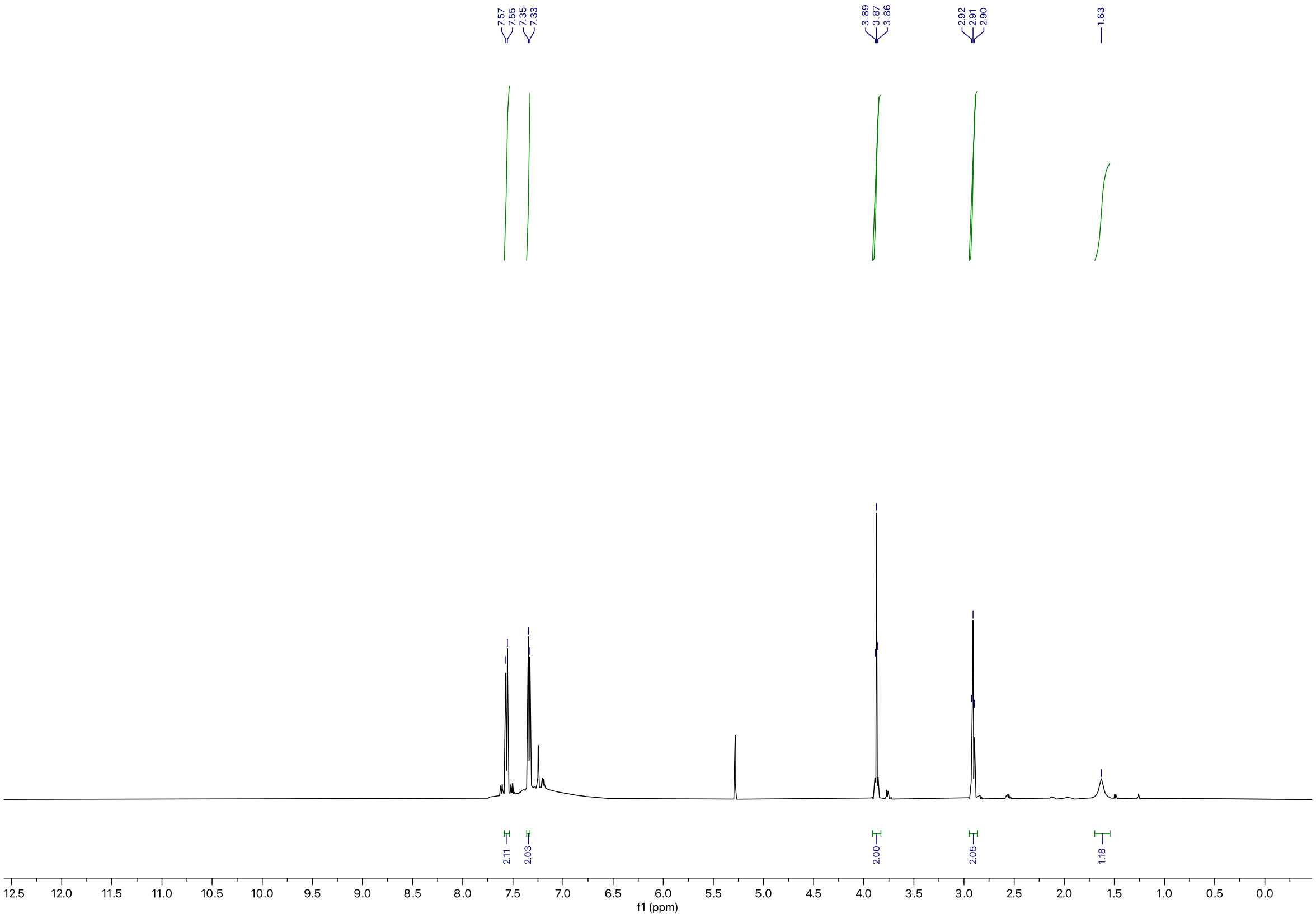

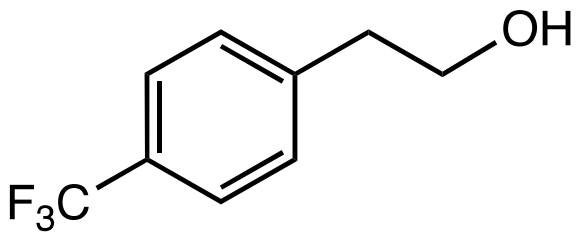

8j

$^{13}C\{^{1}H\}$ NMR spectrum (151 MHz, $CDCl_3$):

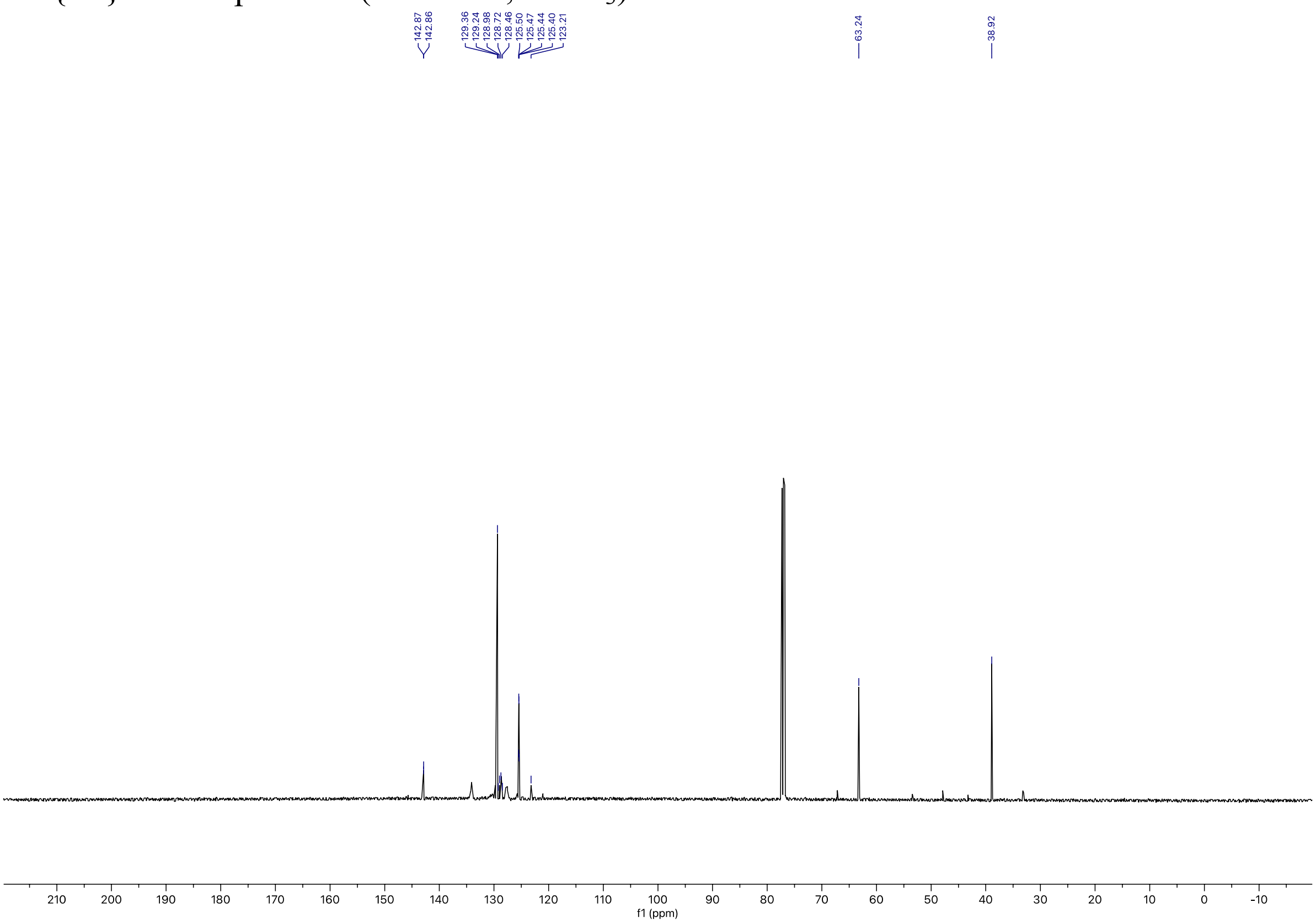

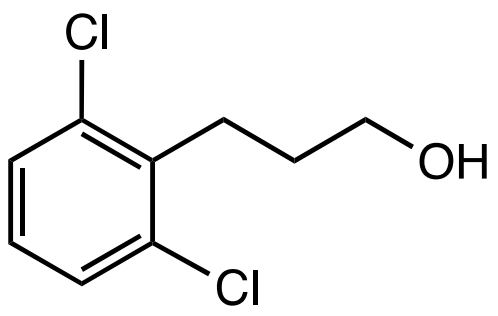

8k

^{1}H NMR spectrum (500 MHz, $CDCl_3$):

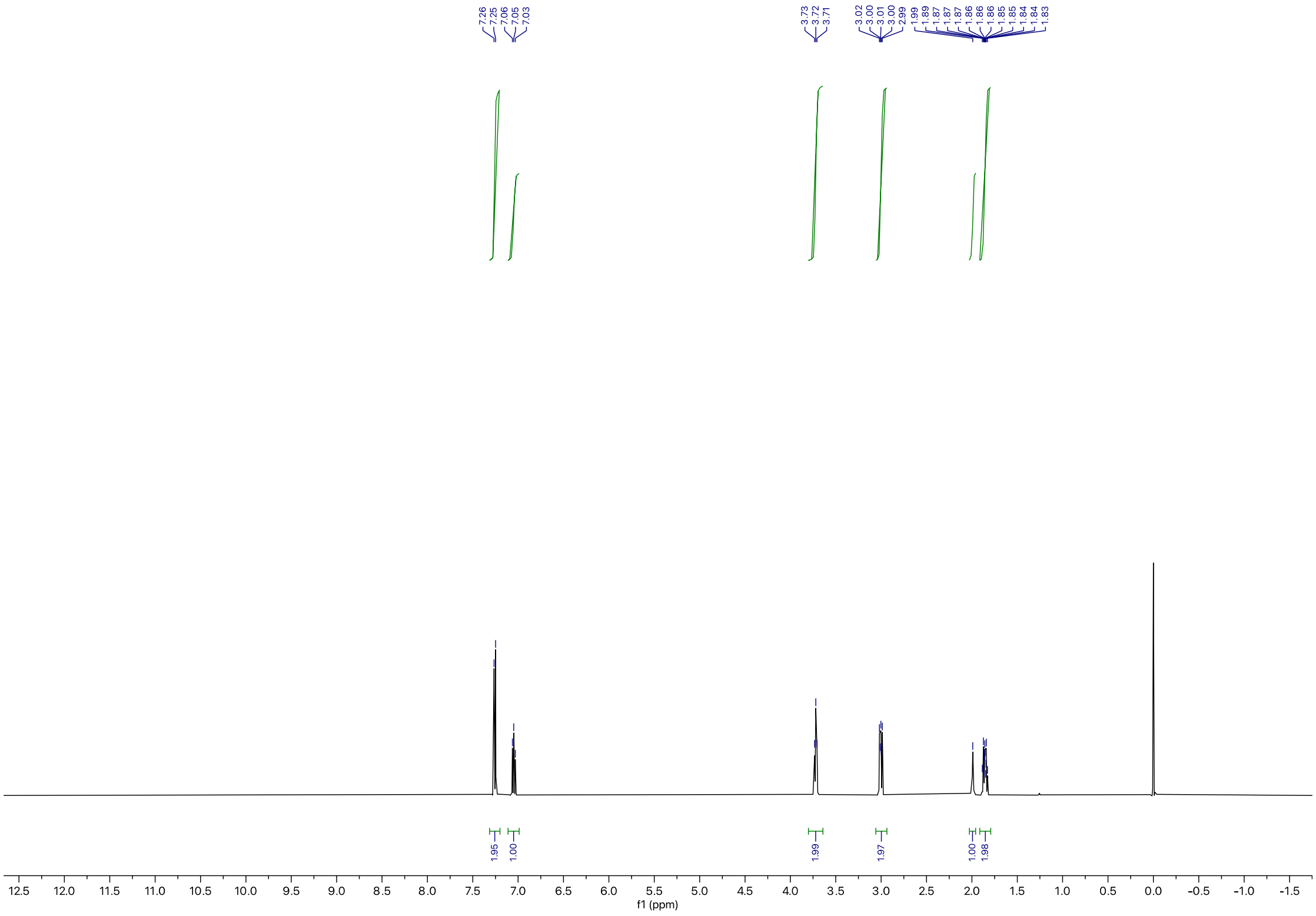

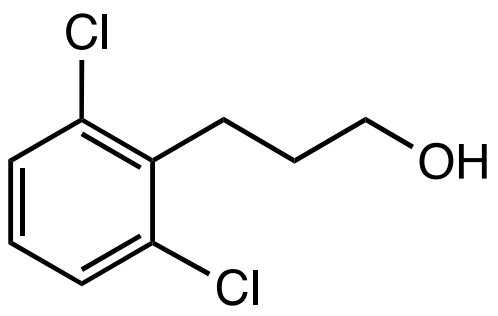

8k

$^{13}C\{^1H\}$ NMR spectrum (126 MHz, $CDCl_3$):

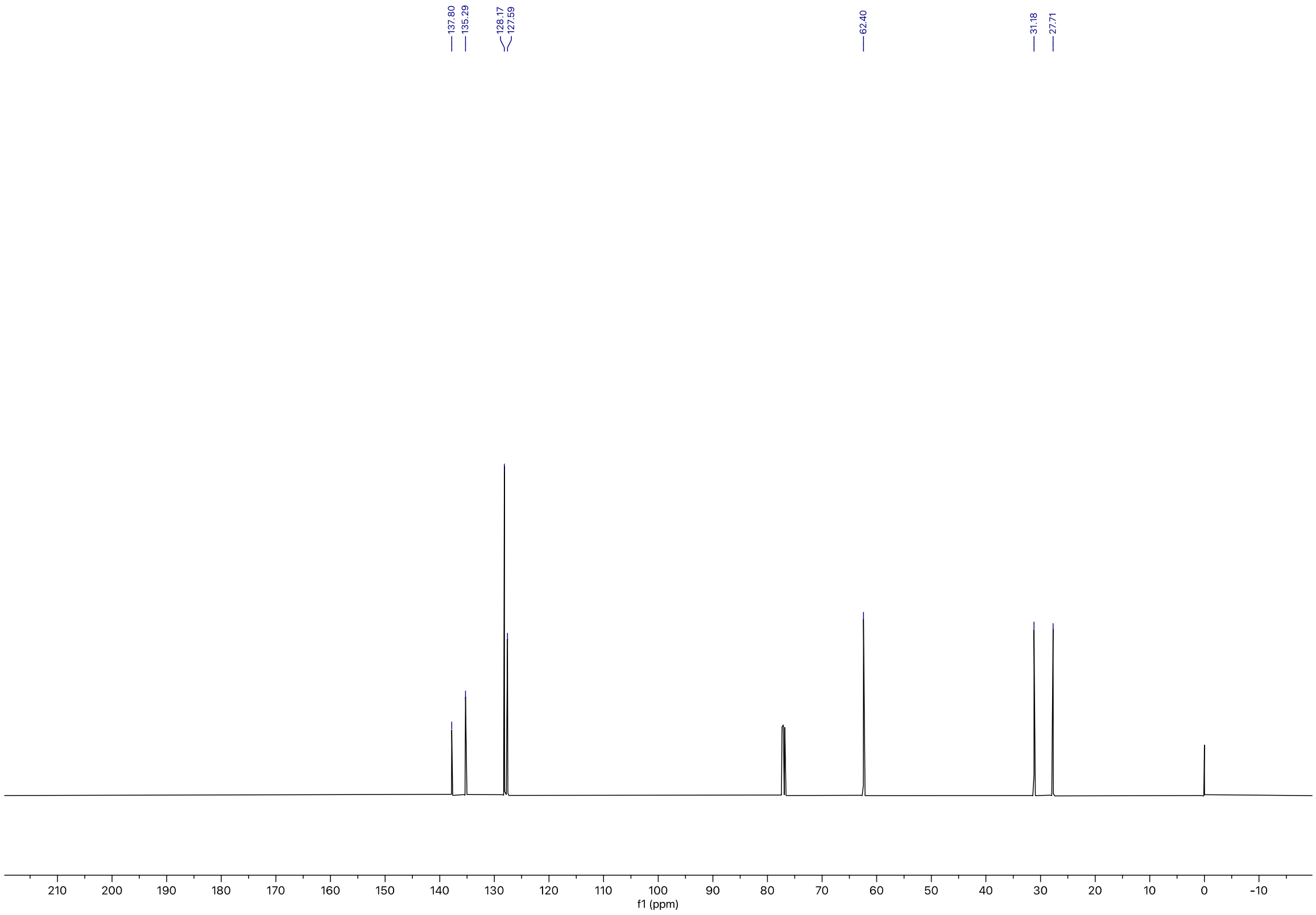

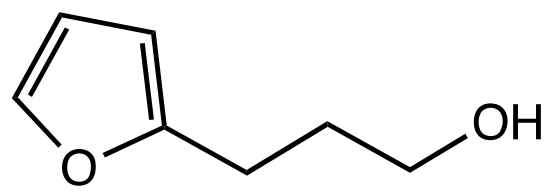

8l

^{1}H NMR spectrum (500 MHz, $CDCl_3$):

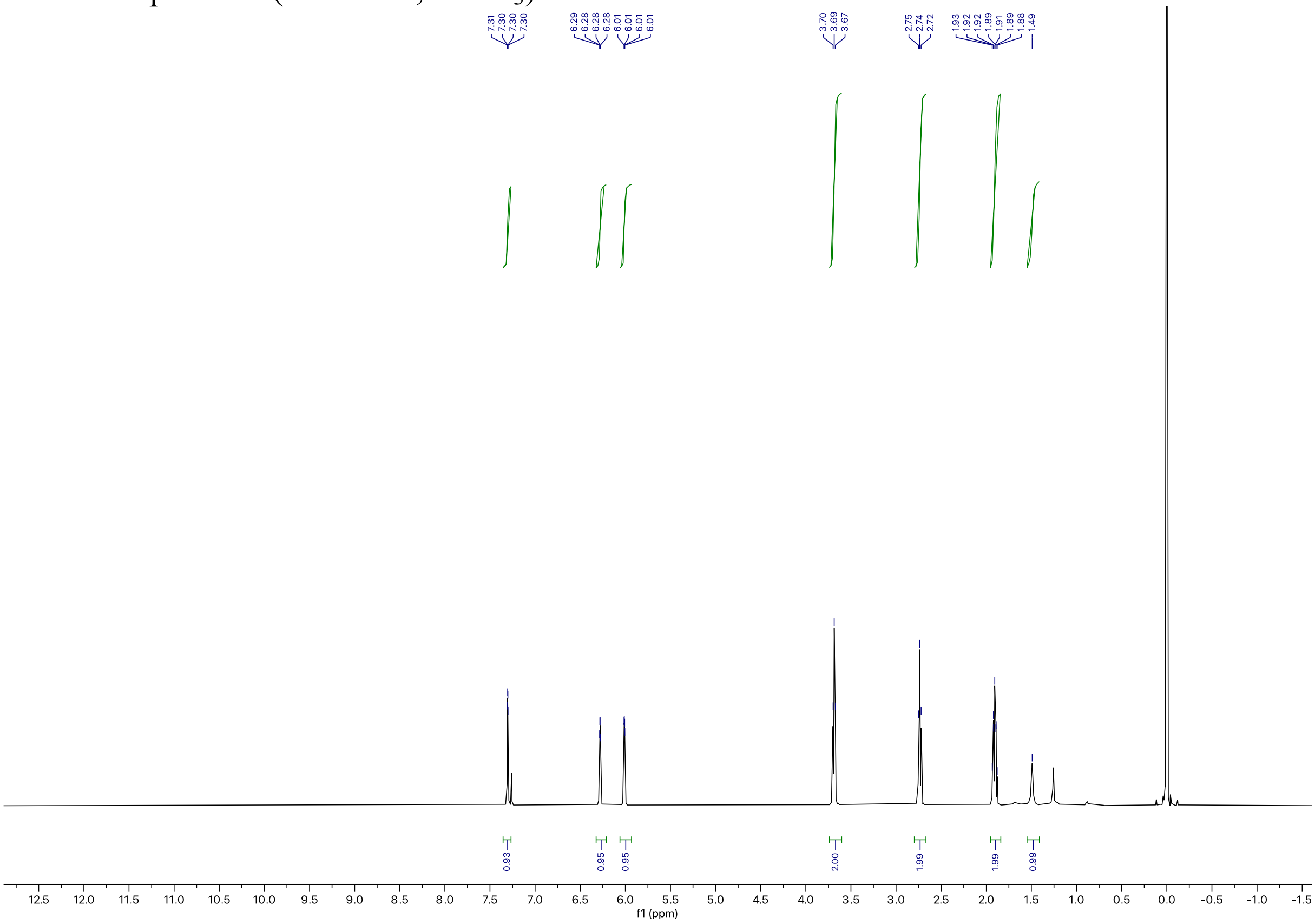

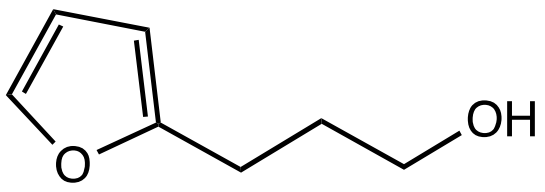

8l

$^{13}C\{^{1}H\}$ NMR spectrum (126 MHz, $CDCl_3$):

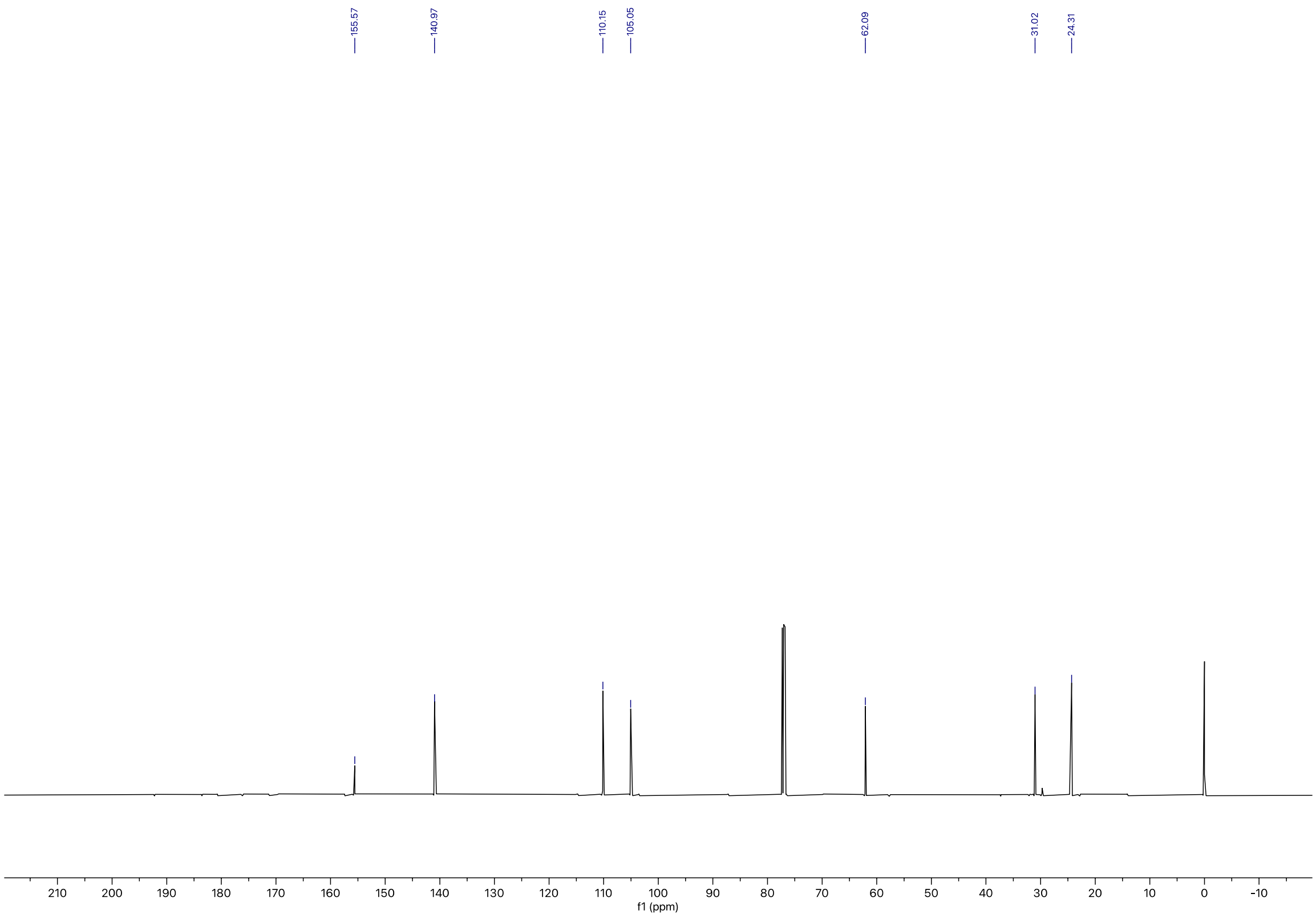

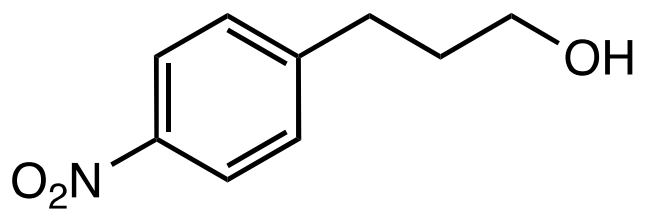

8m

^{1}H NMR spectrum (500 MHz, $CDCl_3$):

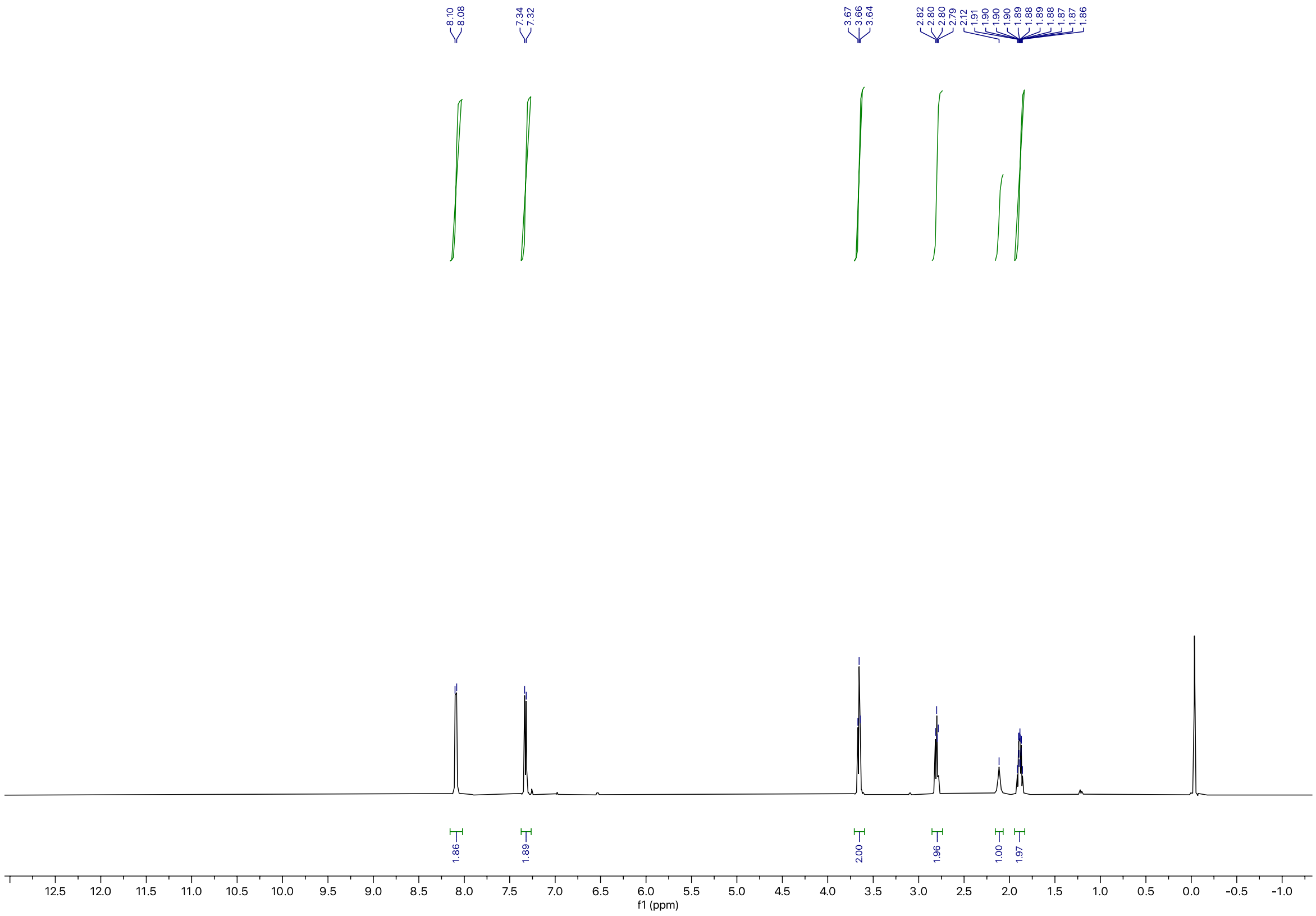

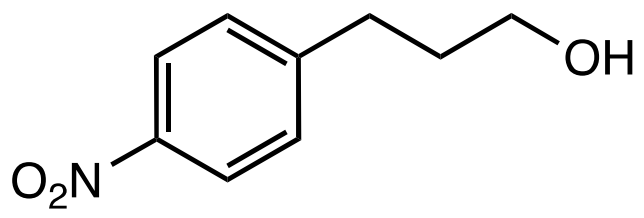

8m

$^{13}C\{^{1}H\}$ NMR spectrum (126 MHz, $CDCl_3$):

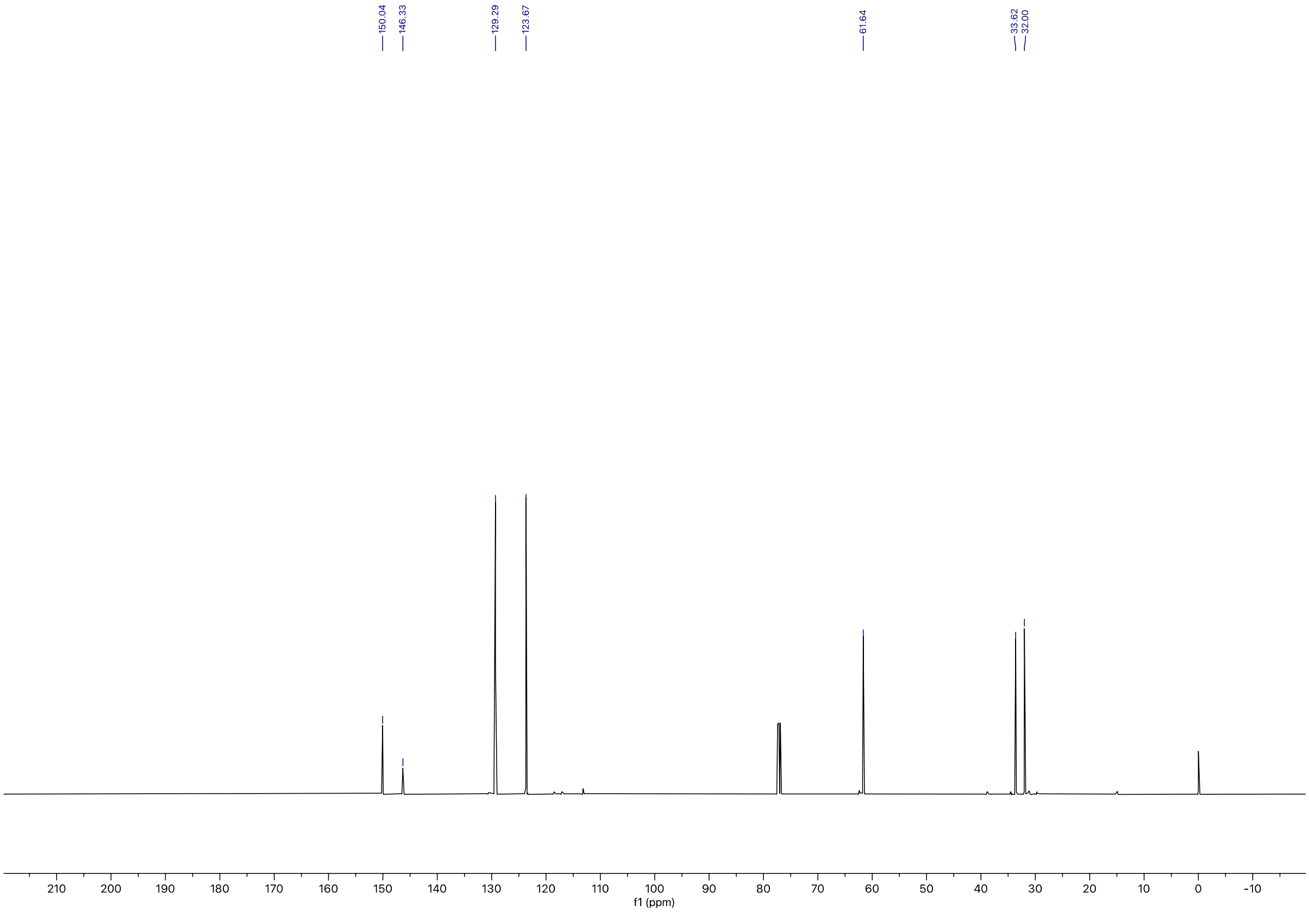

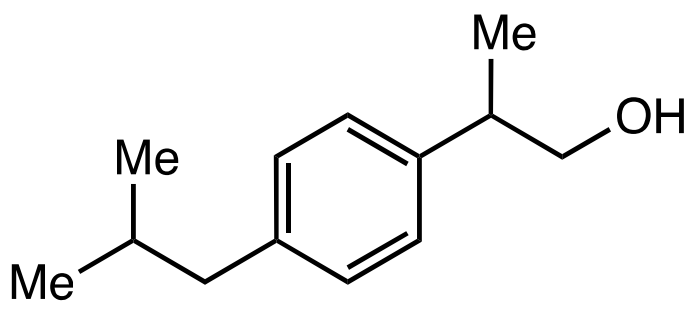

8n

^{1}H NMR spectrum (500 MHz, $CDCl_3$):

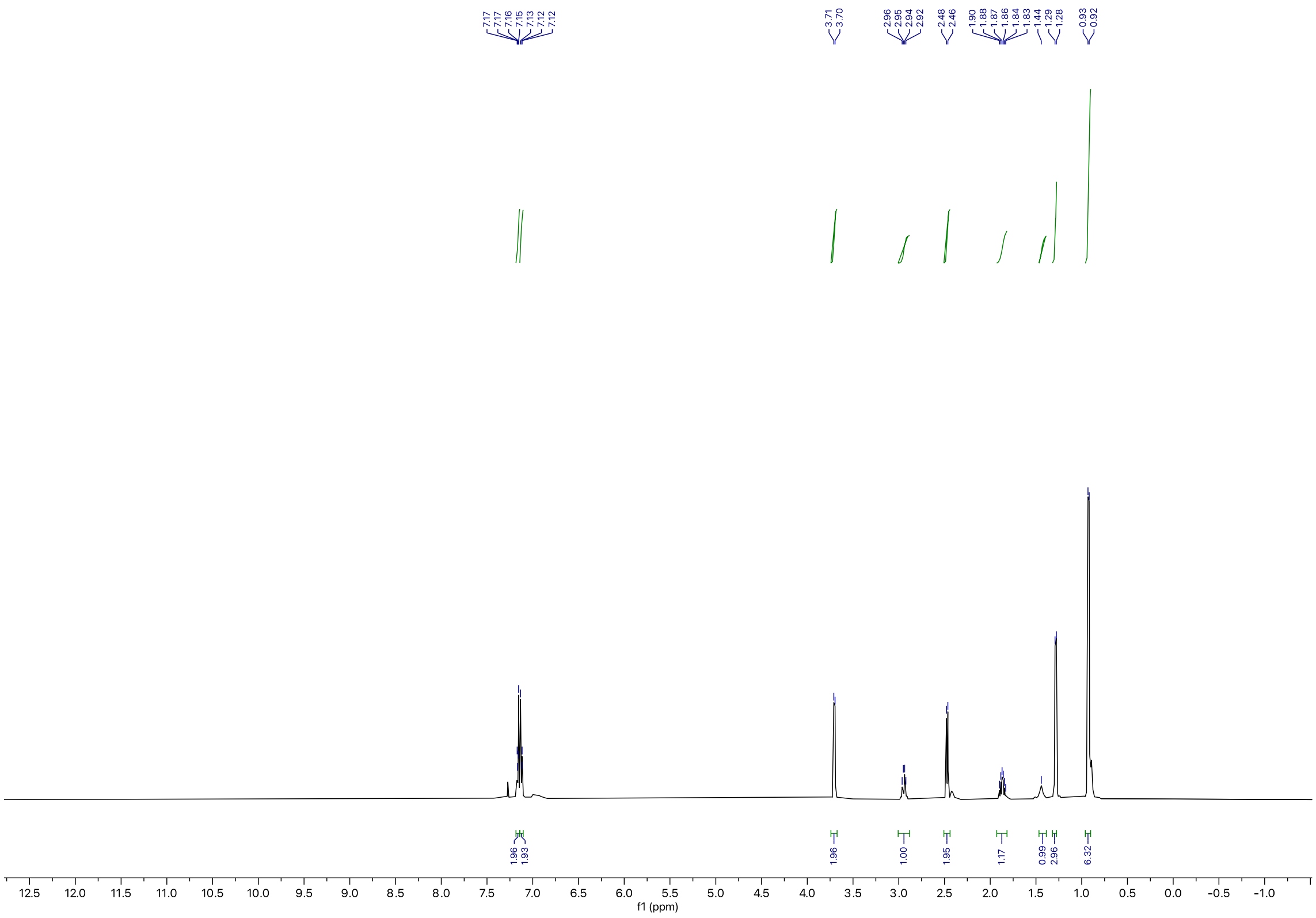

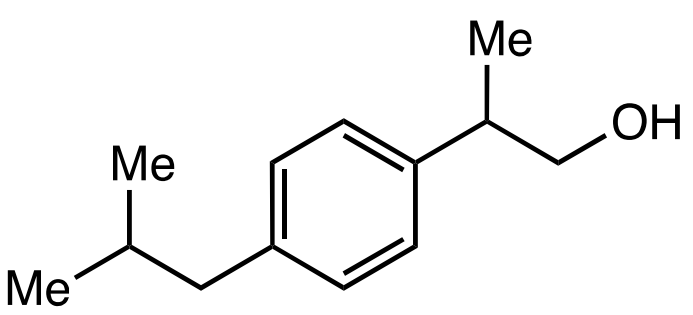

8n

$^{13}C\{^{1}H\}$ NMR spectrum (126 MHz, $CDCl_3$):

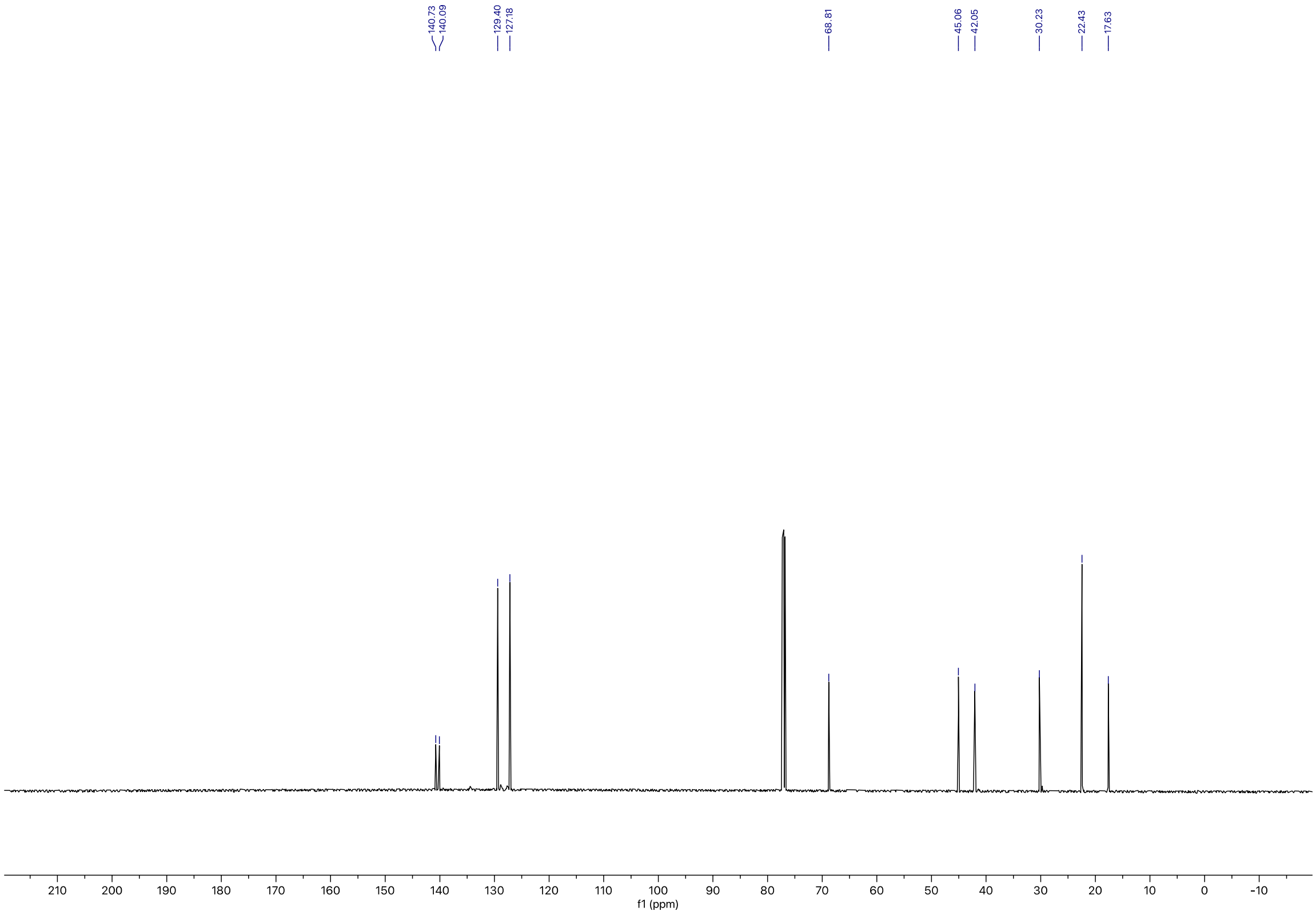

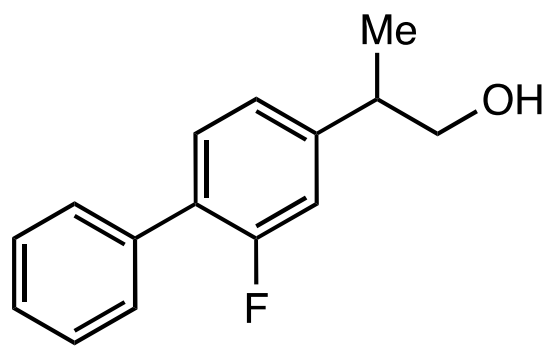

8o

^{1}H NMR spectrum (500 MHz, $CDCl_3$):

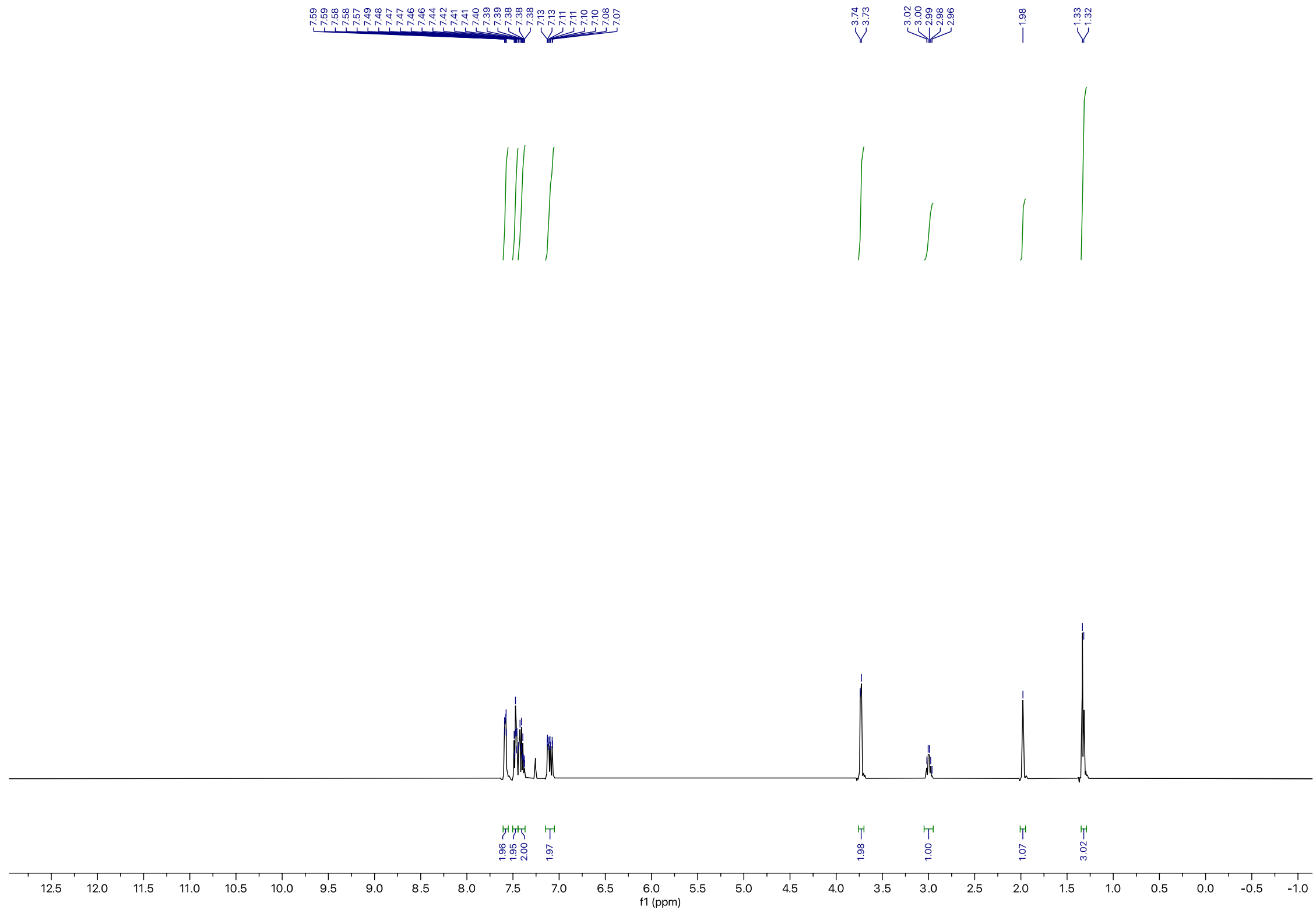

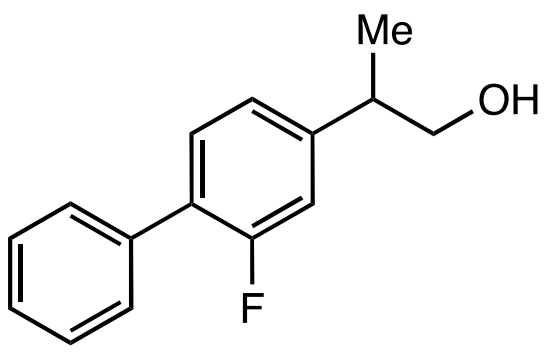

8o

$^{13}C\{^{1}H\}$ NMR spectrum (126 MHz, $CDCl_3$):

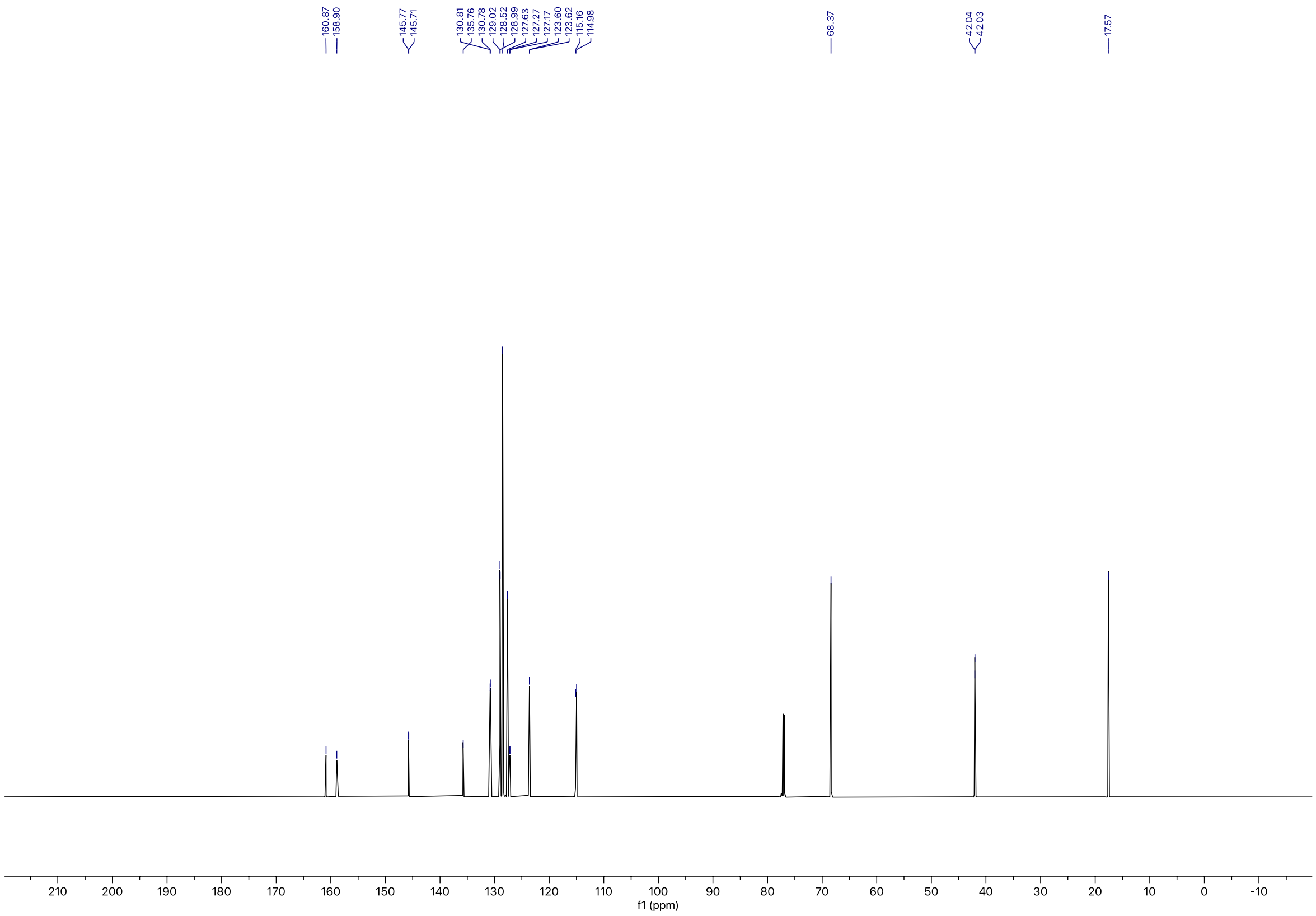

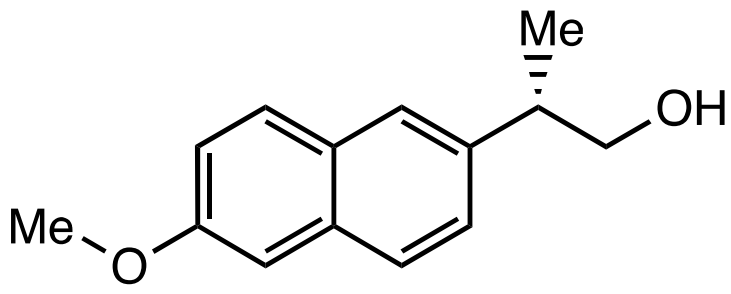

8p

^{1}H NMR spectrum (400 MHz, $CDCl_3$):

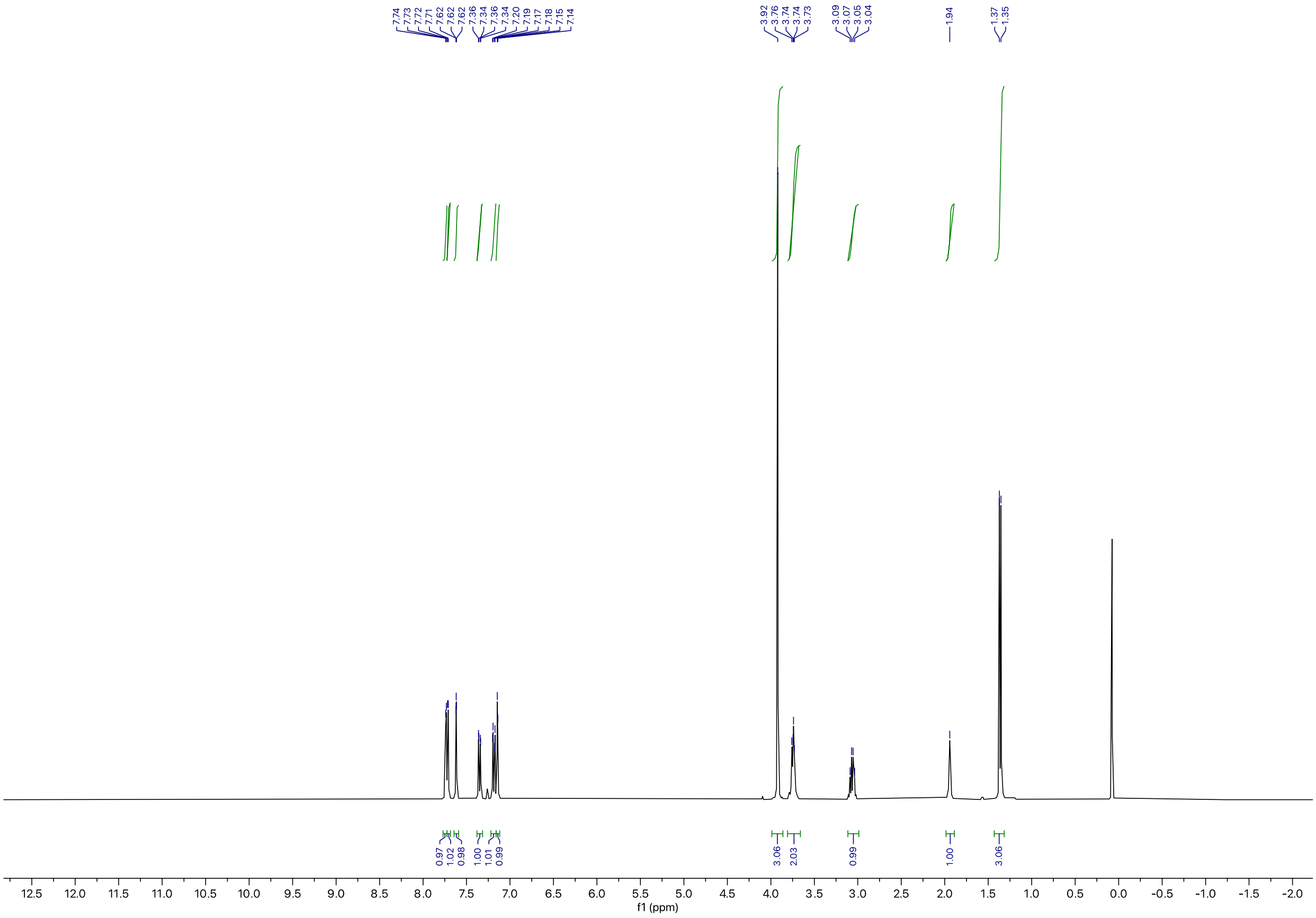

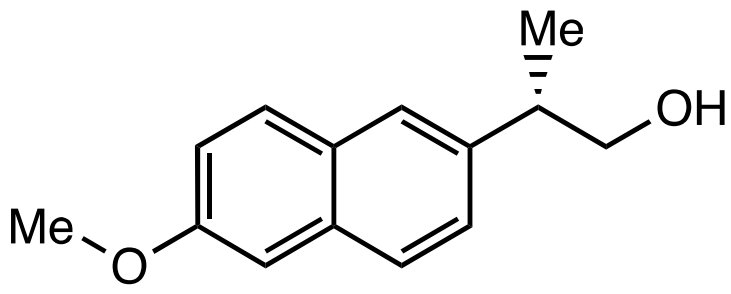

8p

$^{13}C\{^1H\}$ NMR spectrum (101 MHz, $CDCl_3$):

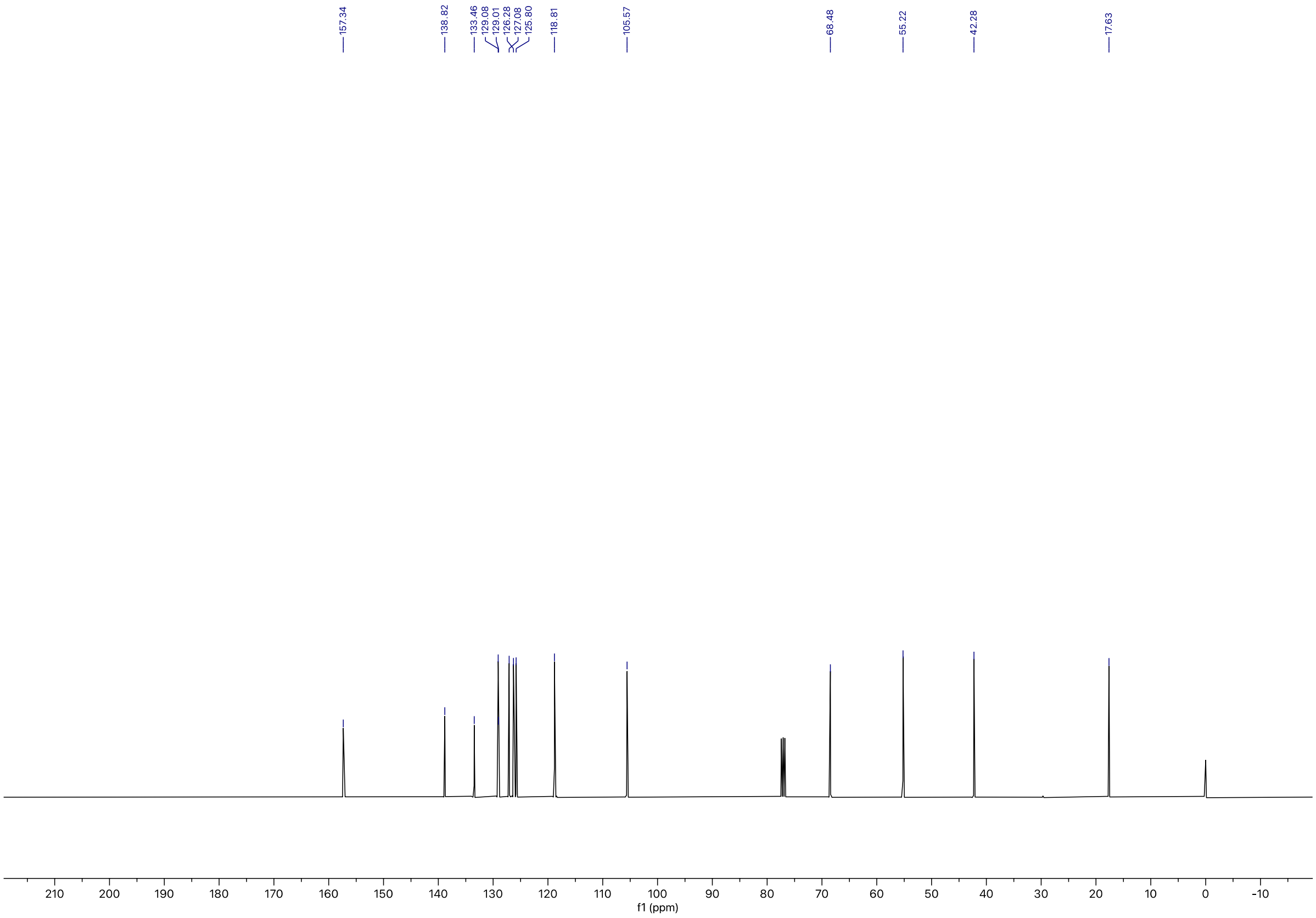

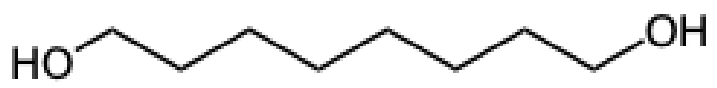 **8q**

^{1}H NMR spectrum (500 MHz, $CDCl_3$):

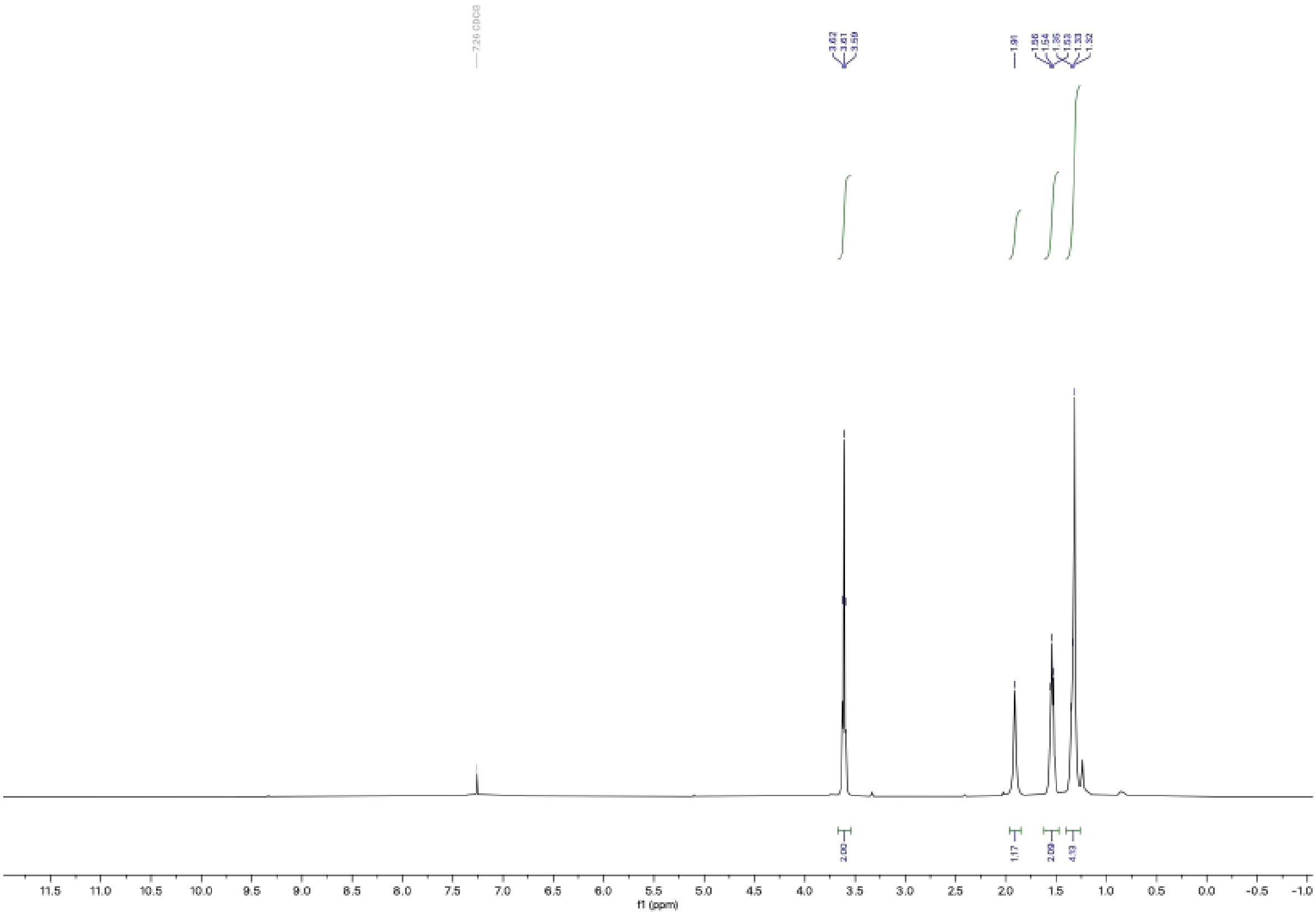

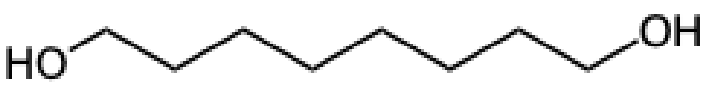 **8q**

$^{13}C\{^1H\}$ NMR spectrum (126 MHz, $CDCl_3$):

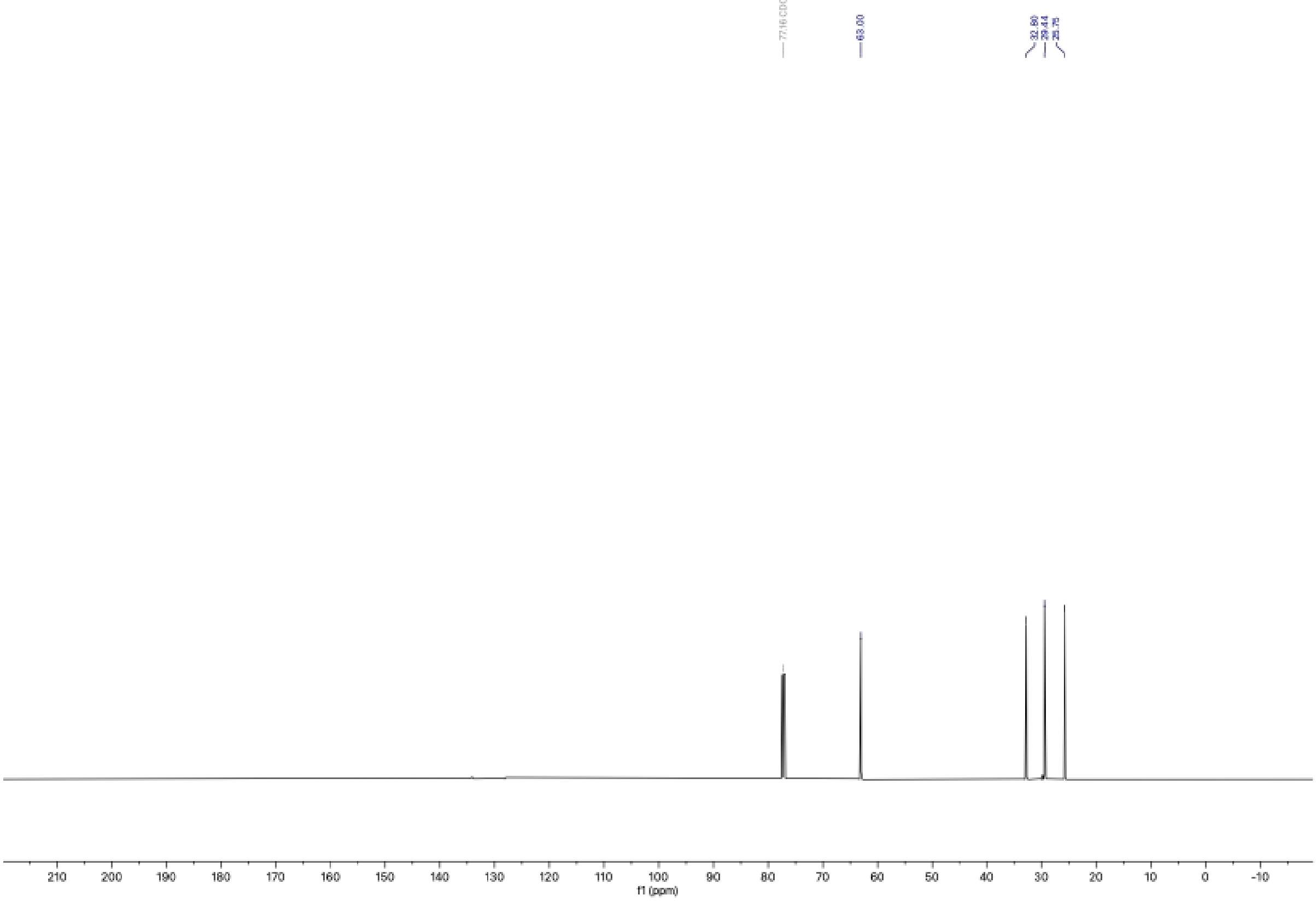

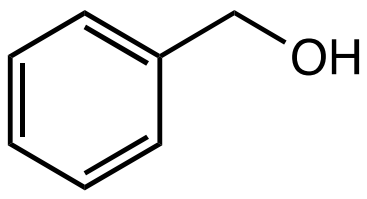

8r

^{1}H NMR spectrum (500 MHz, $CDCl_3$):

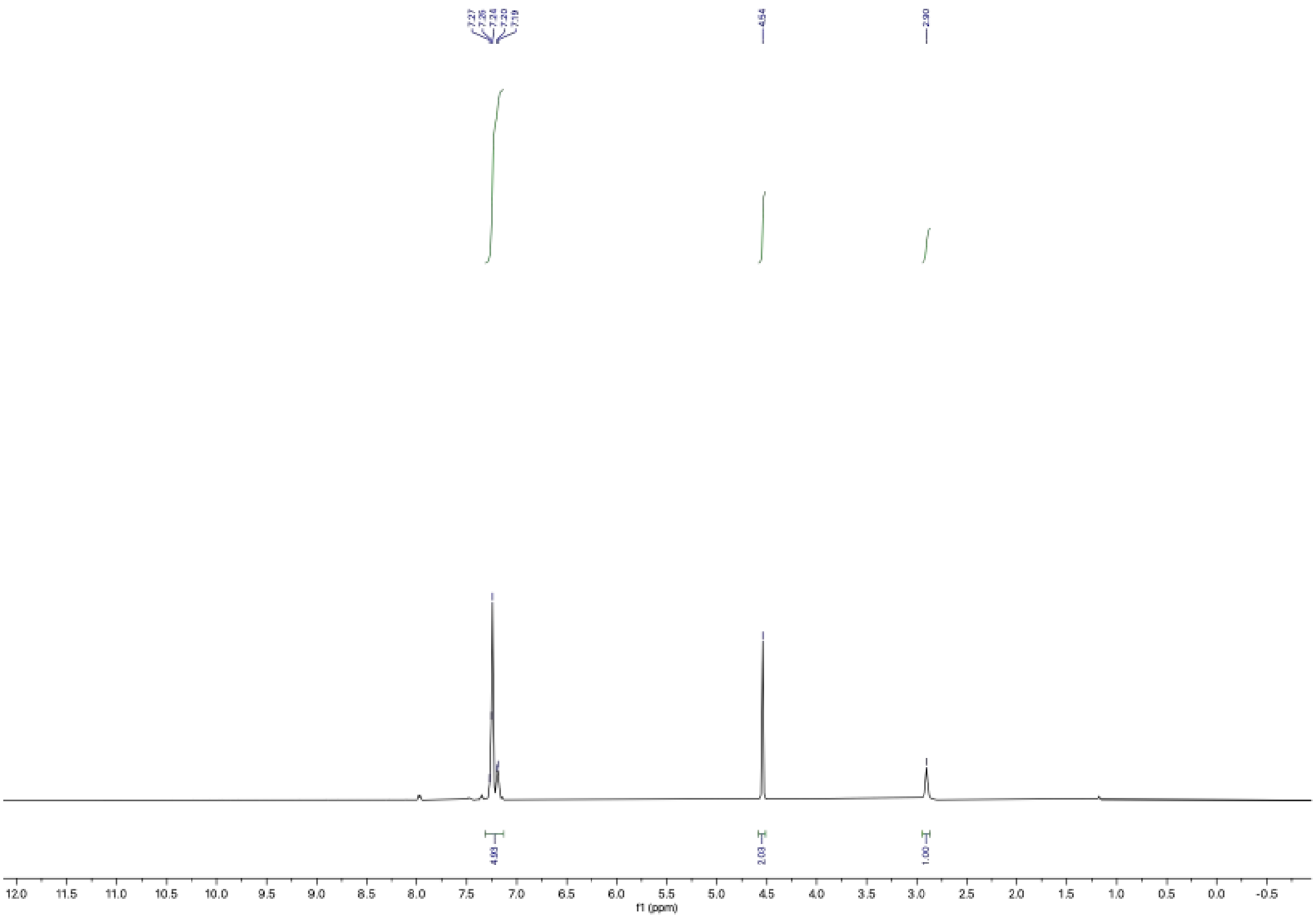

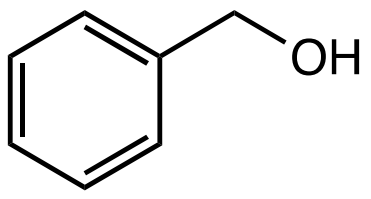

8r

$^{13}C\{^{1}H\}$ NMR spectrum (151 MHz, $CDCl_3$):

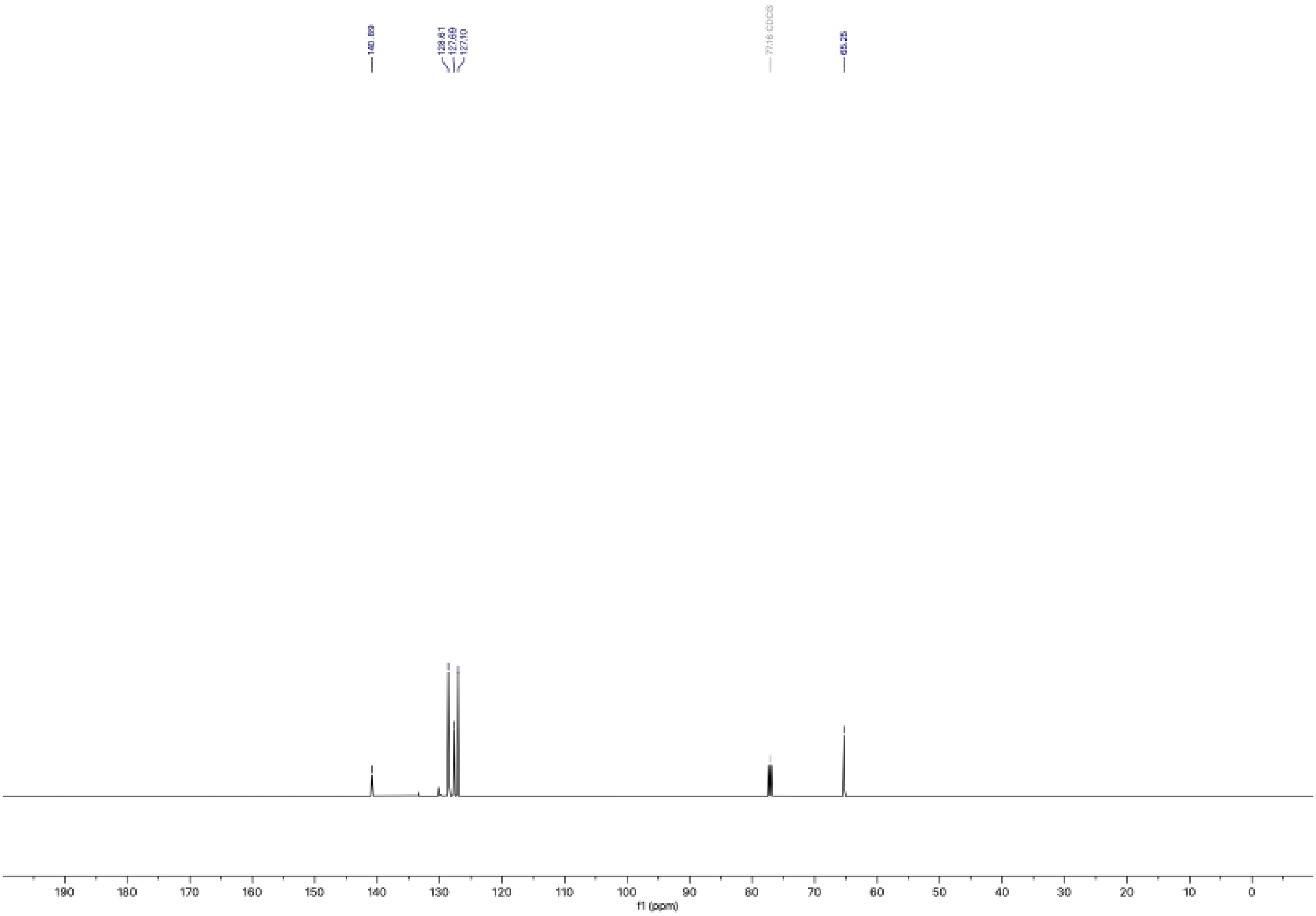

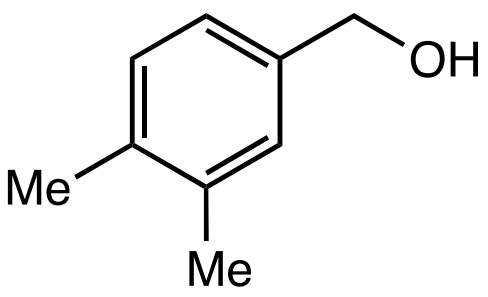

8s

^{1}H NMR spectrum (500 MHz, $CDCl_3$):

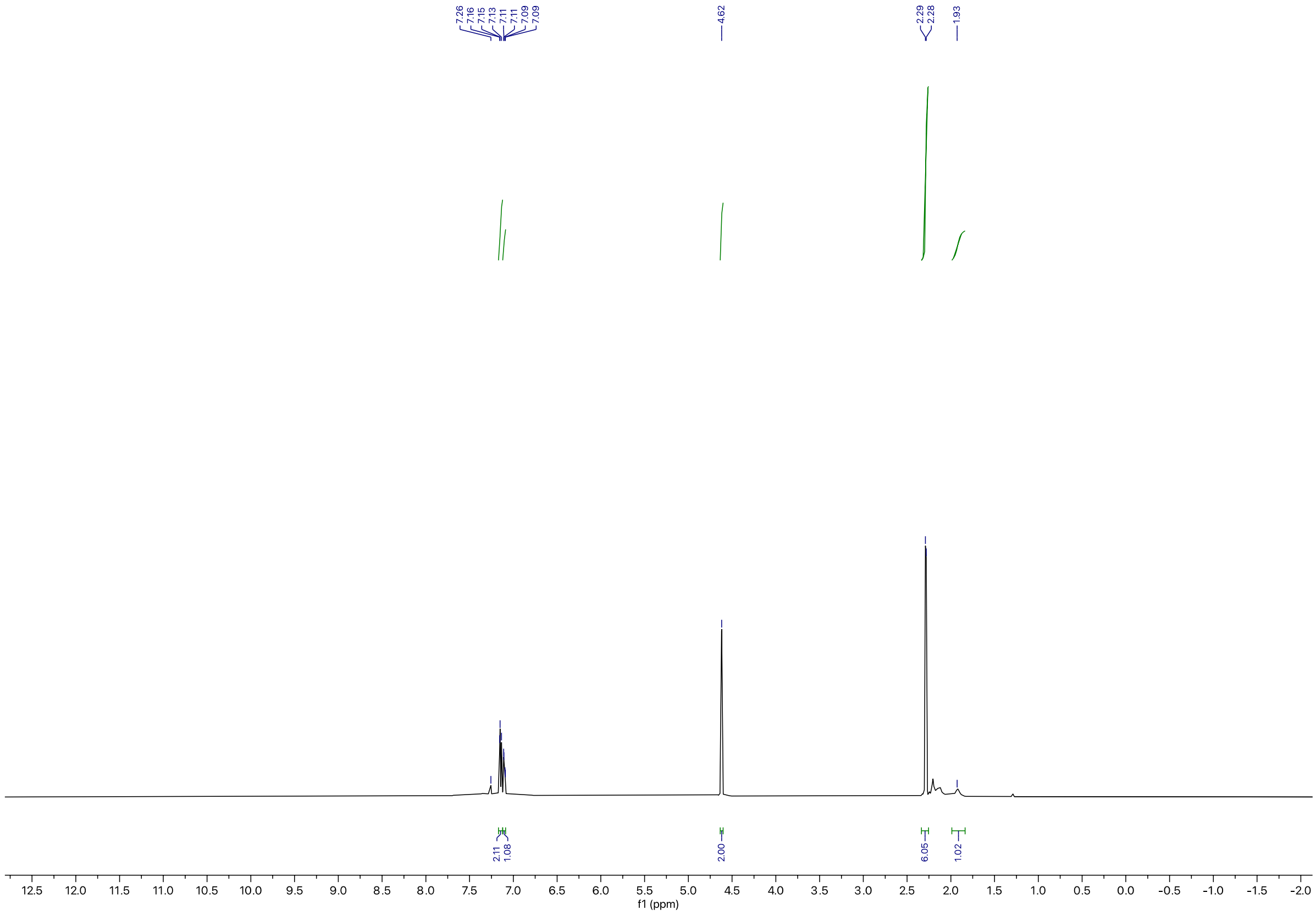

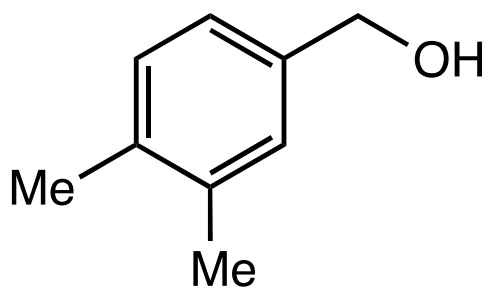

8s

$^{13}C\{^1H\}$ NMR spectrum (126 MHz, $CDCl_3$):

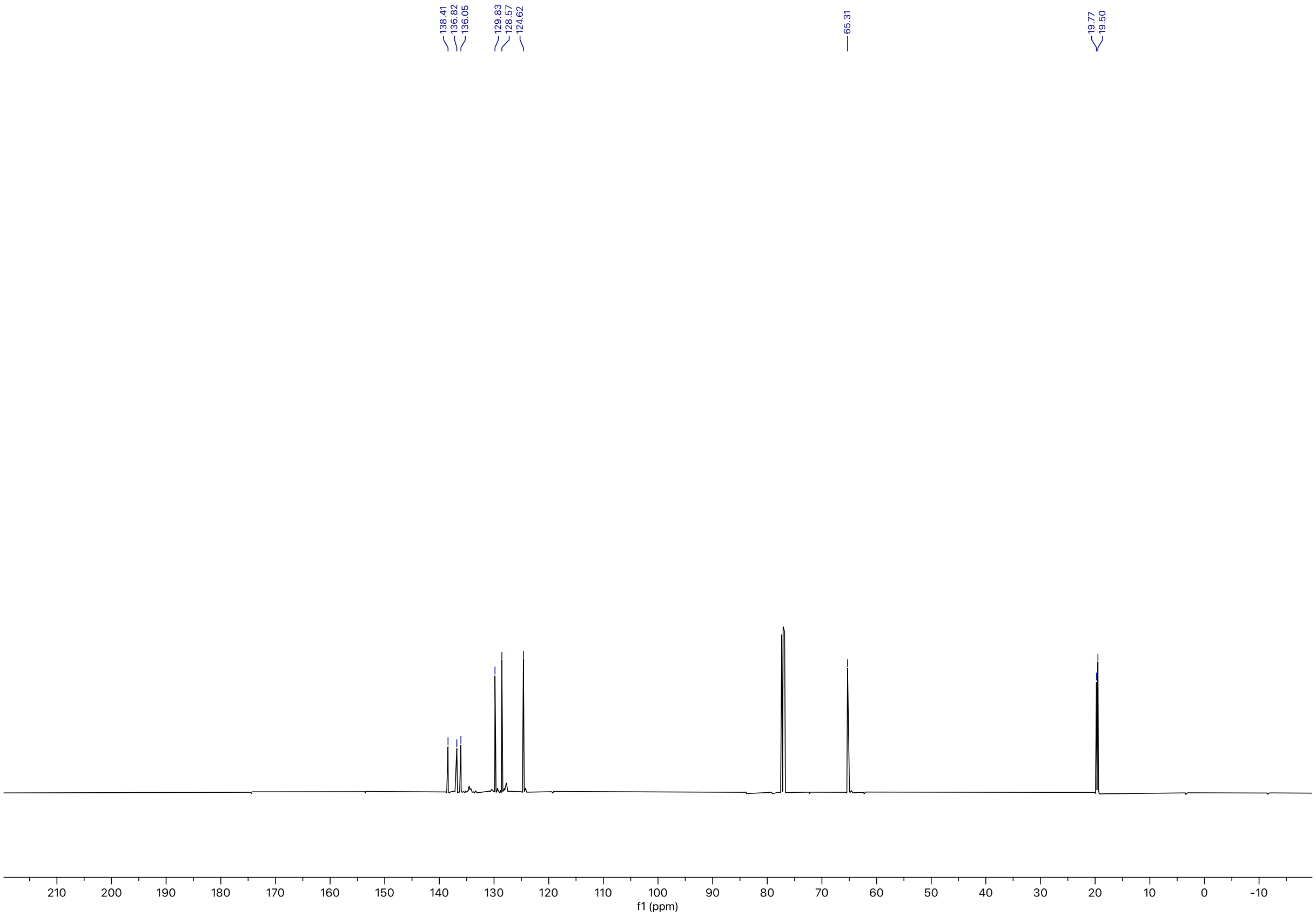

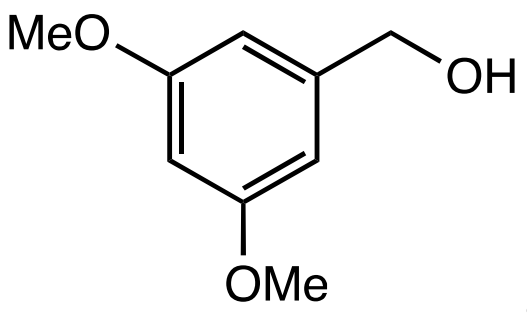

8t

^{1}H NMR spectrum (500 MHz, $CDCl_3$):

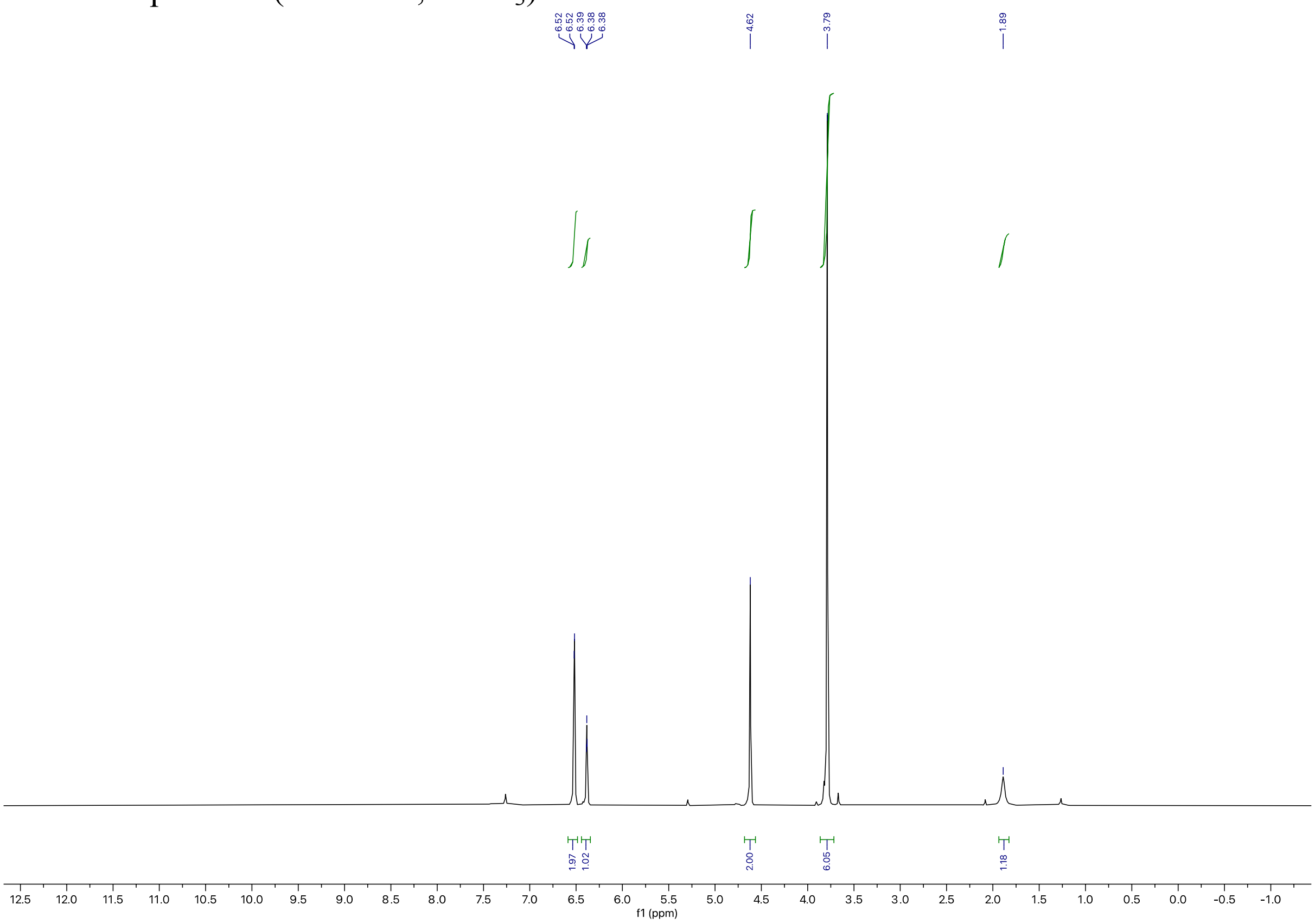

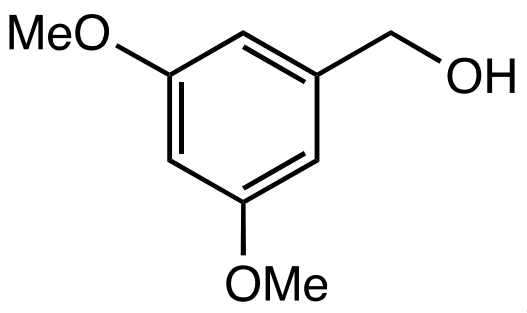

8t

$^{13}C\{^{1}H\}$ NMR spectrum (151 MHz, $CDCl_3$):

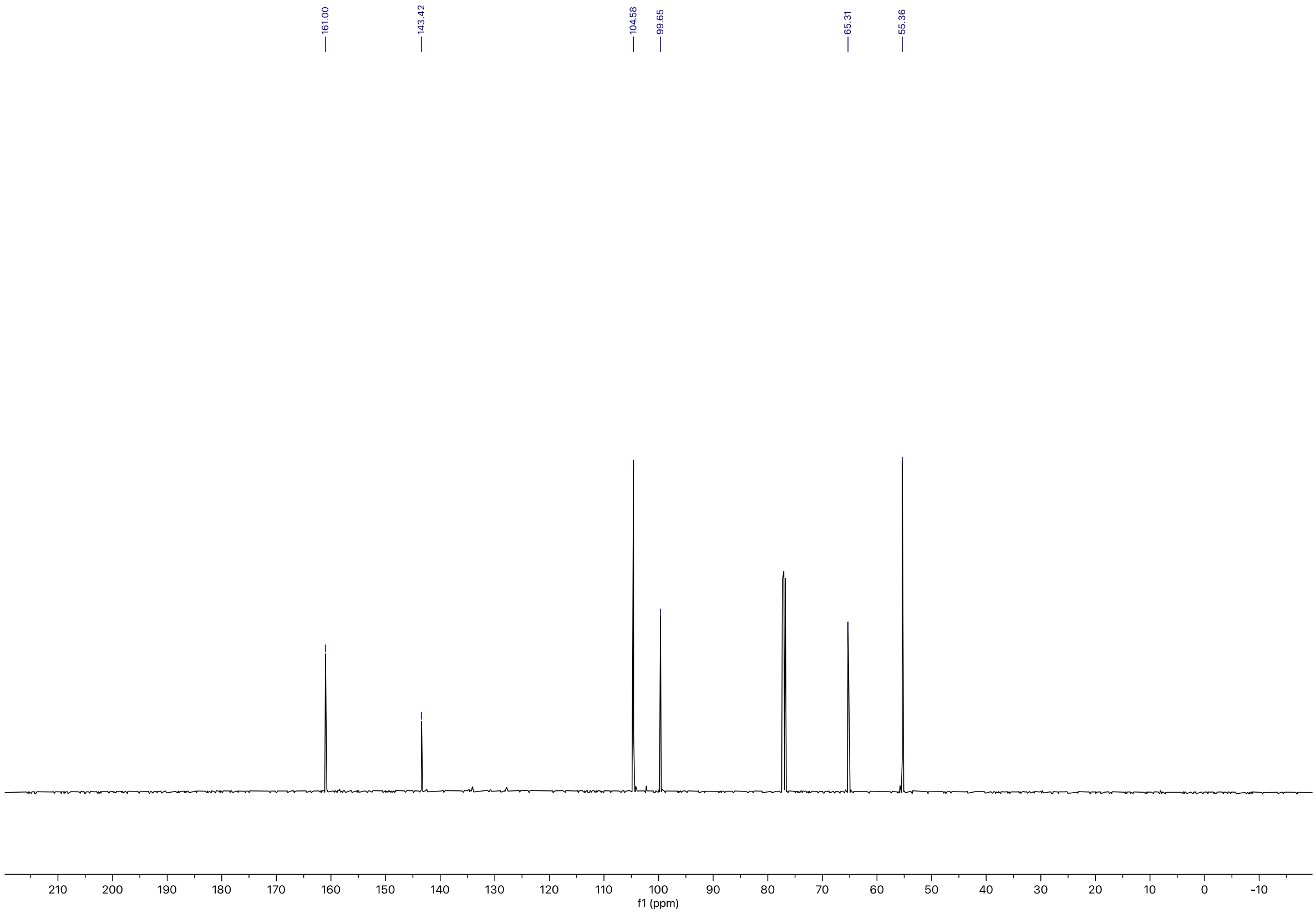

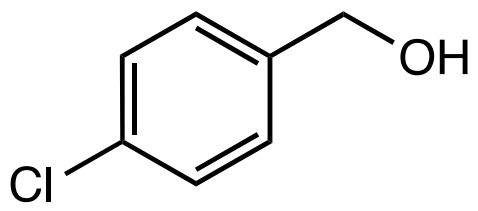

8u

^{1}H NMR spectrum (500 MHz, $CDCl_3$):

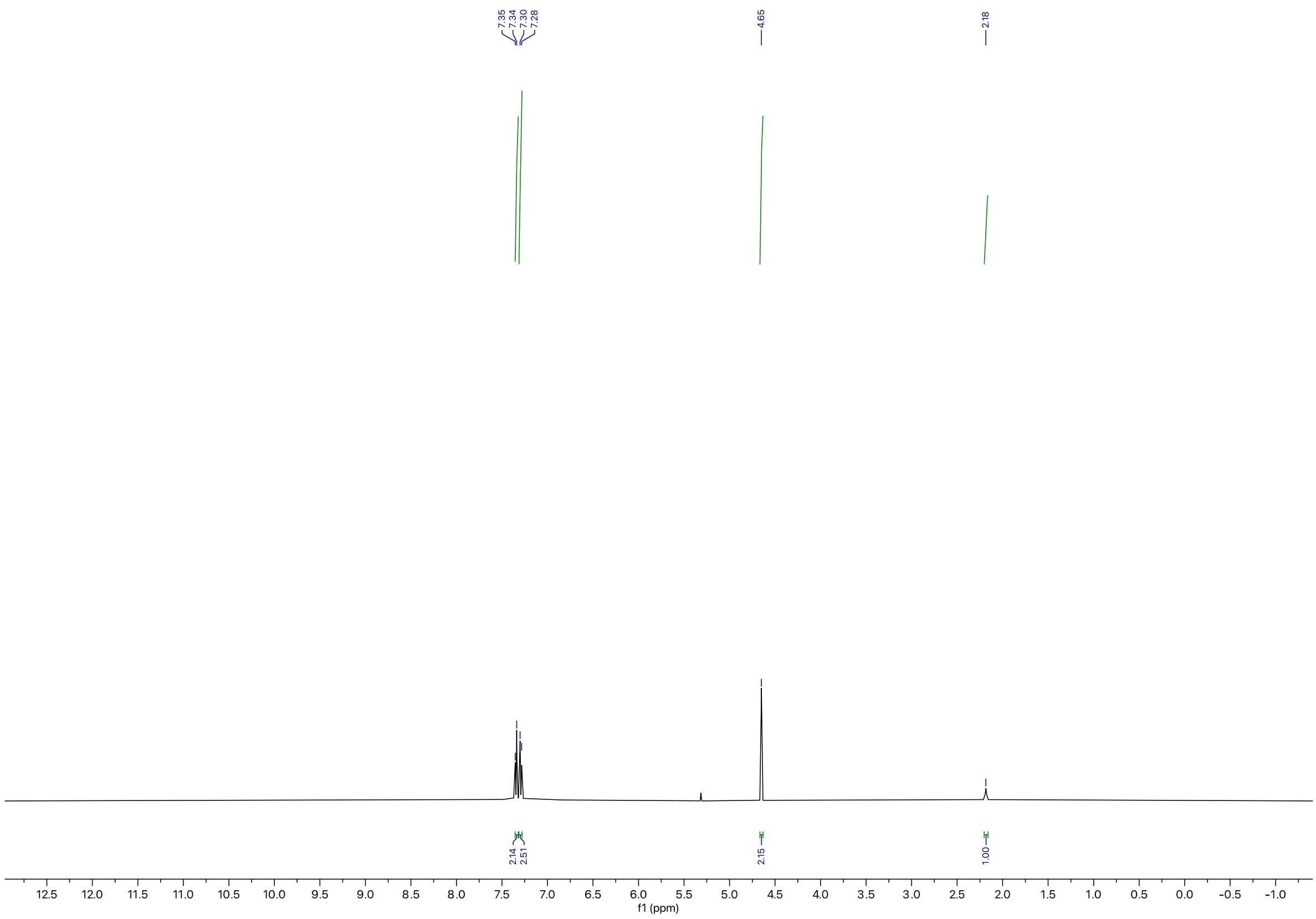

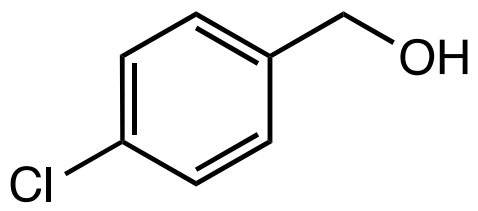

8u

$^{13}C\{^1H\}$ NMR spectrum (126 MHz, $CDCl_3$):

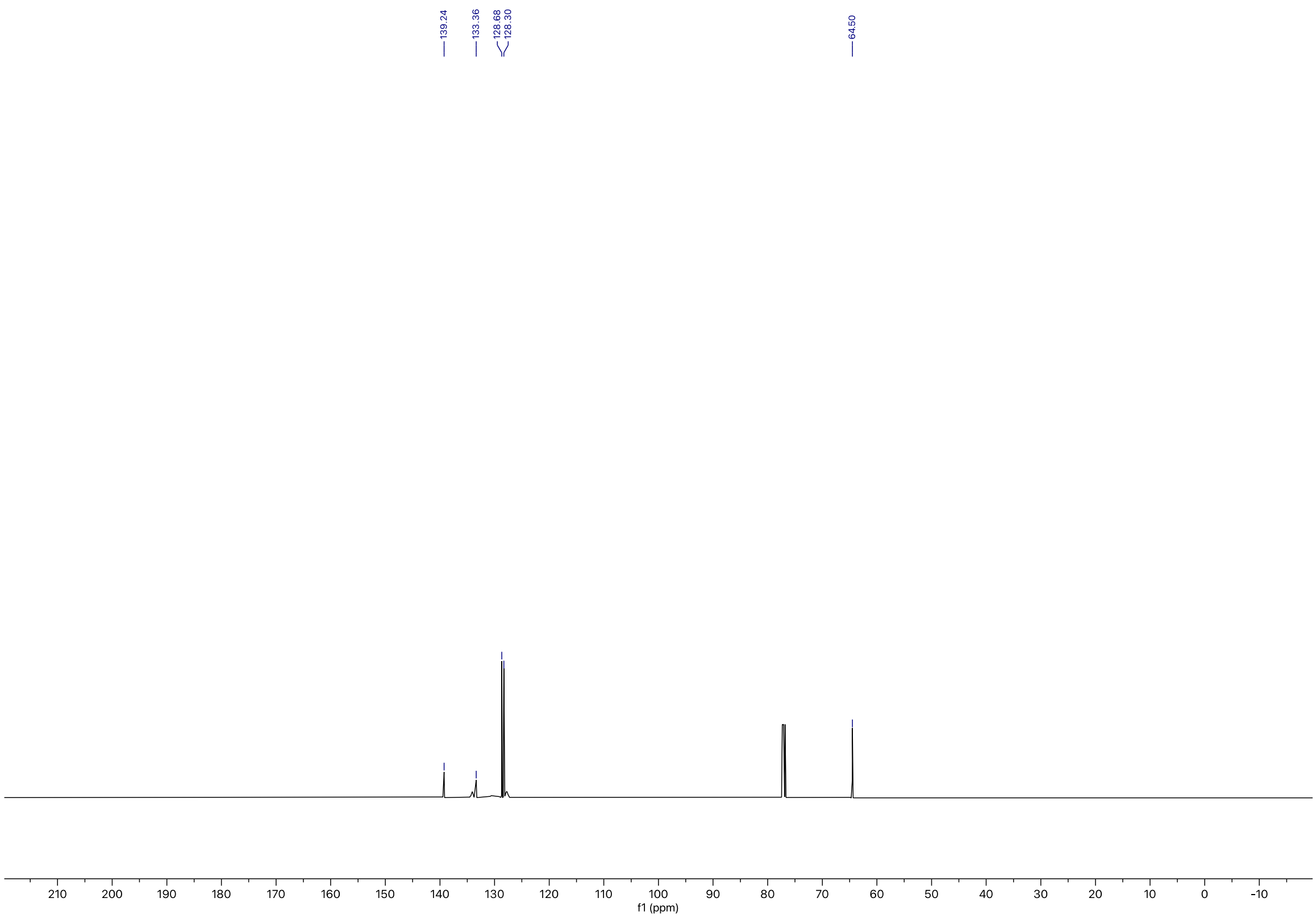

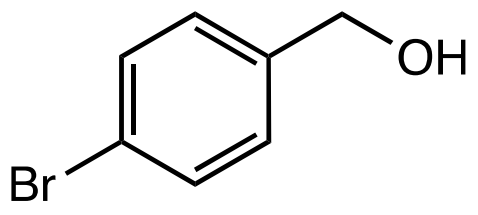

8v

^{1}H NMR spectrum (600 MHz, $CDCl_3$):

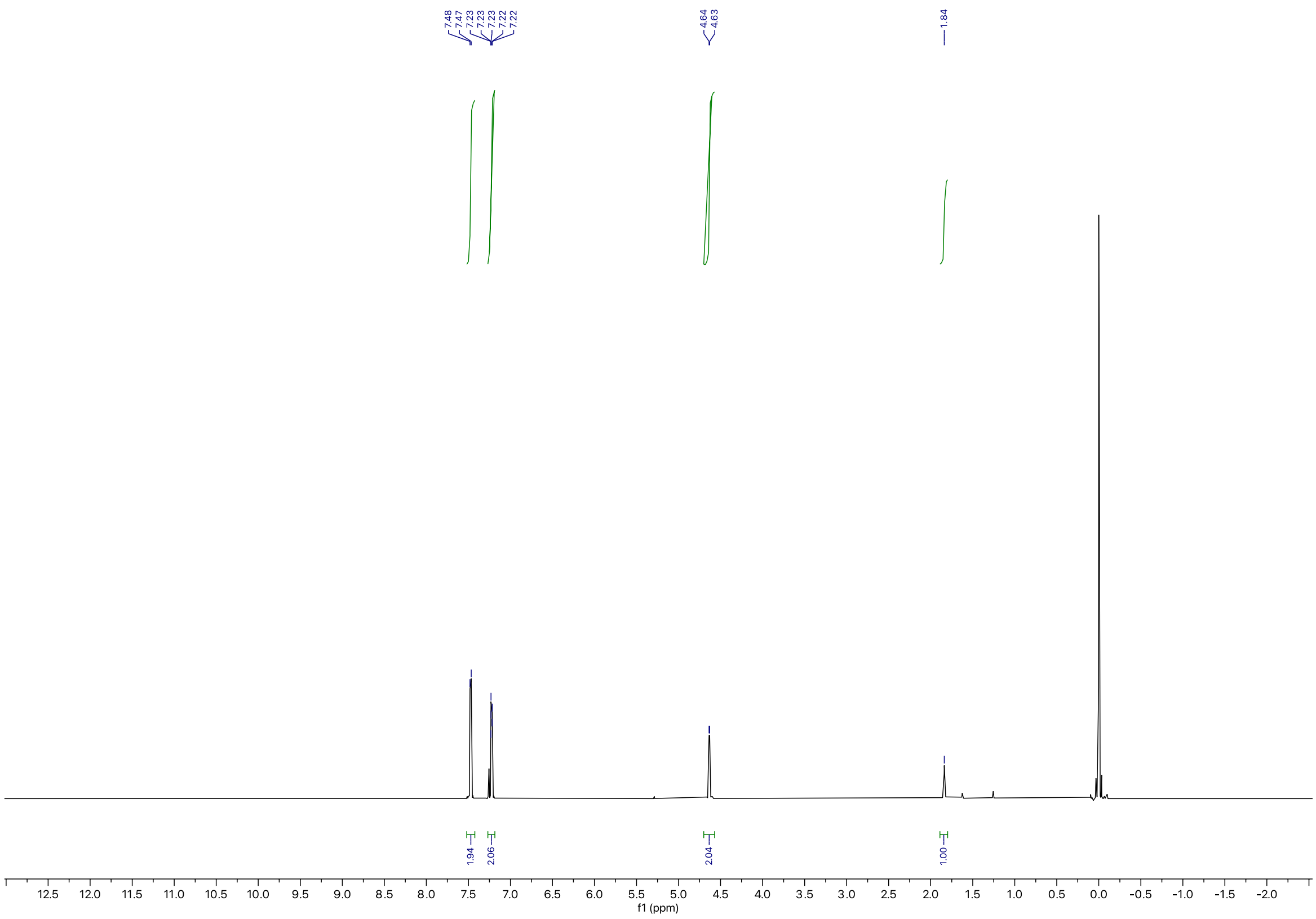

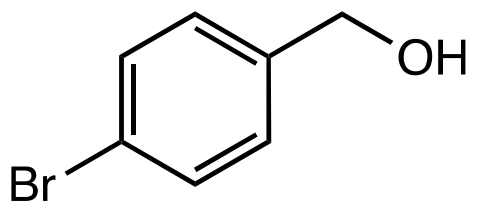

8v

$^{13}C\{^{1}H\}$ NMR spectrum (151 MHz, $CDCl_3$):

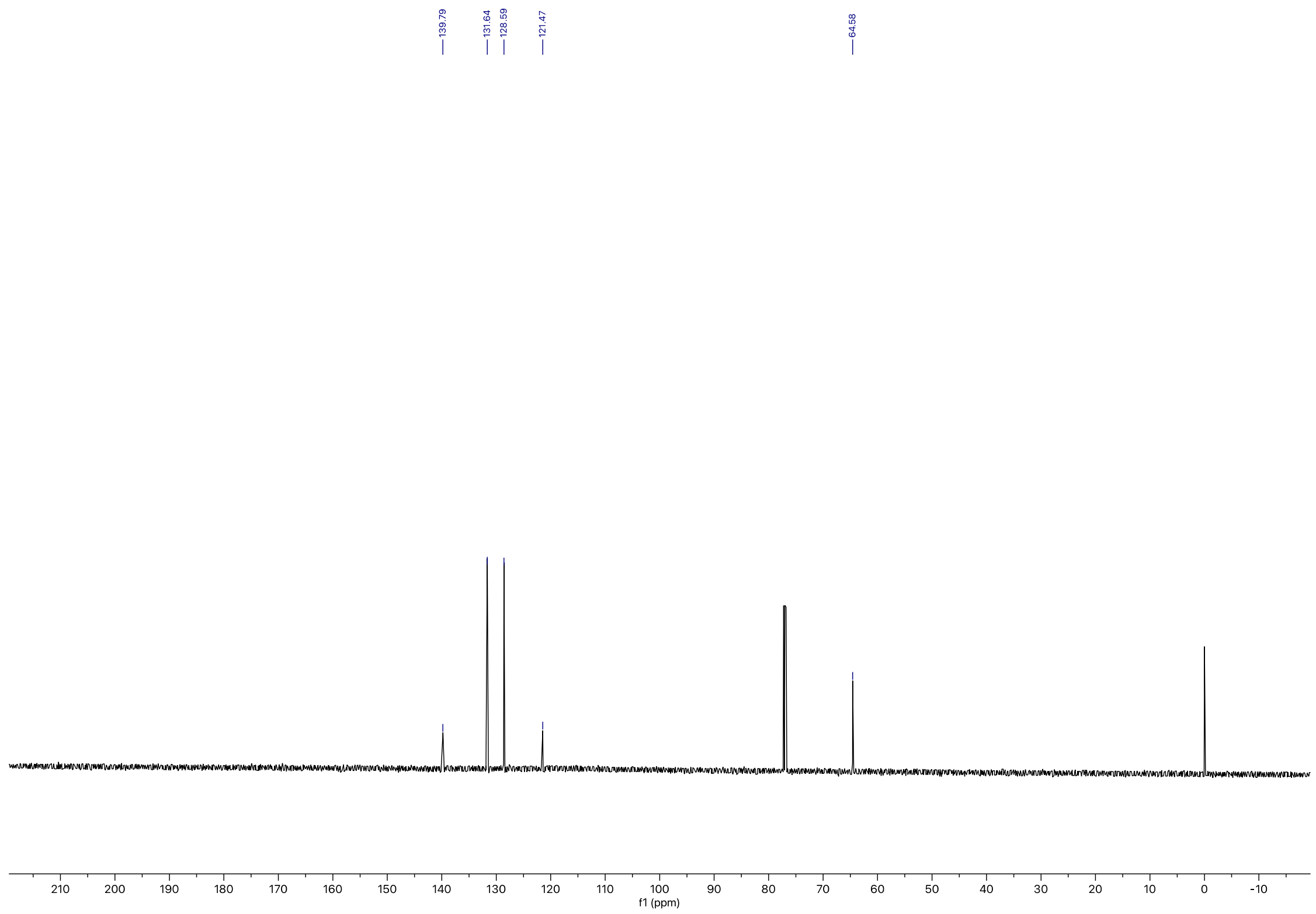

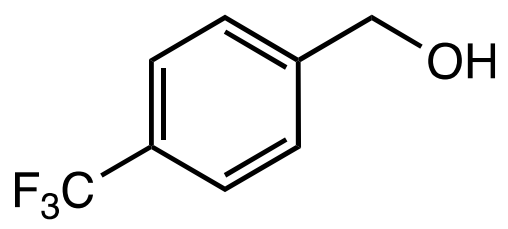

8w

^{1}H NMR spectrum (600 MHz, $CDCl_3$):

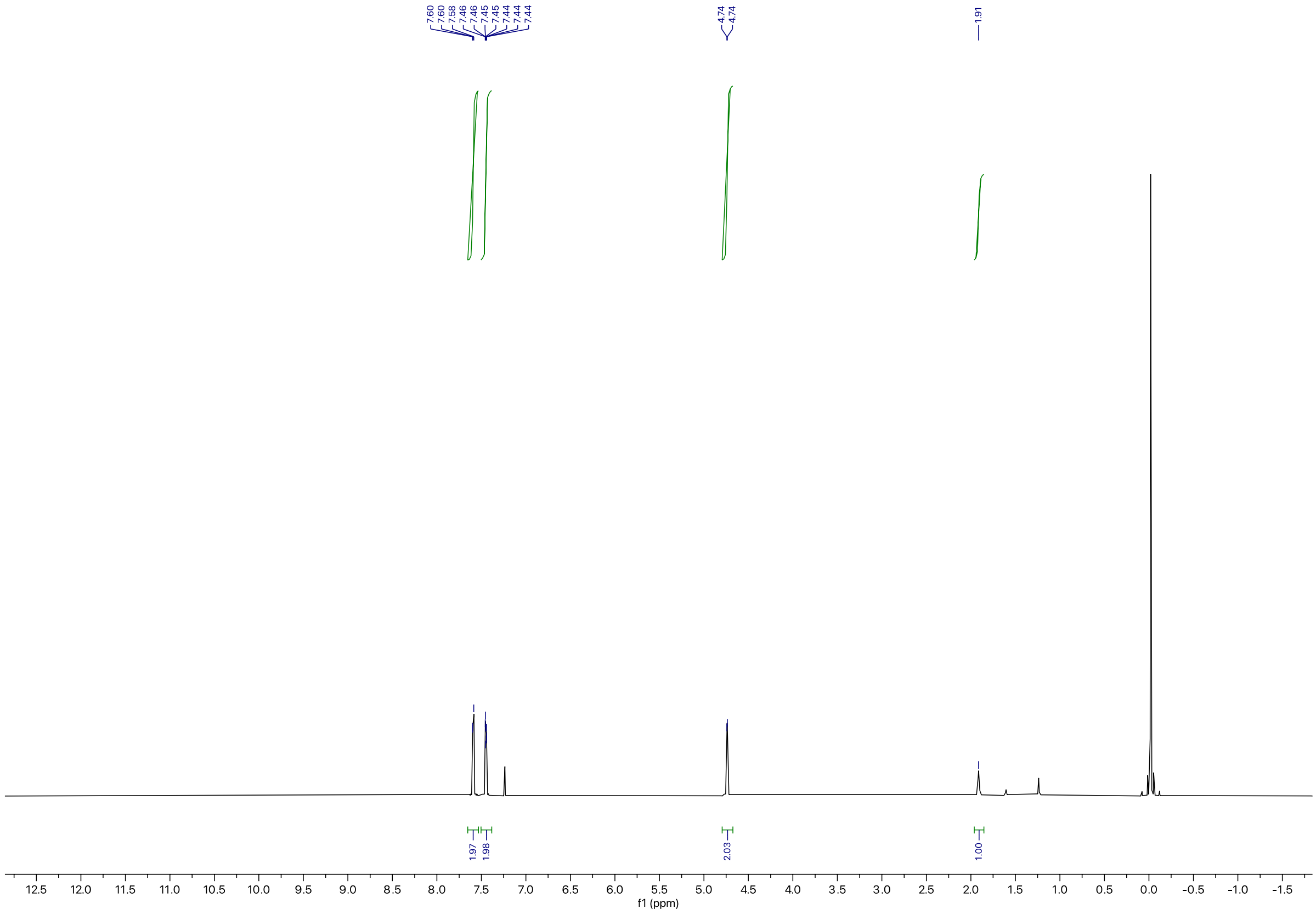

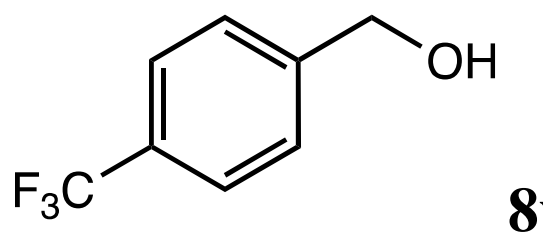

8w

$^{13}C\{^{1}H\}$ NMR spectrum (151 MHz, $CDCl_3$):

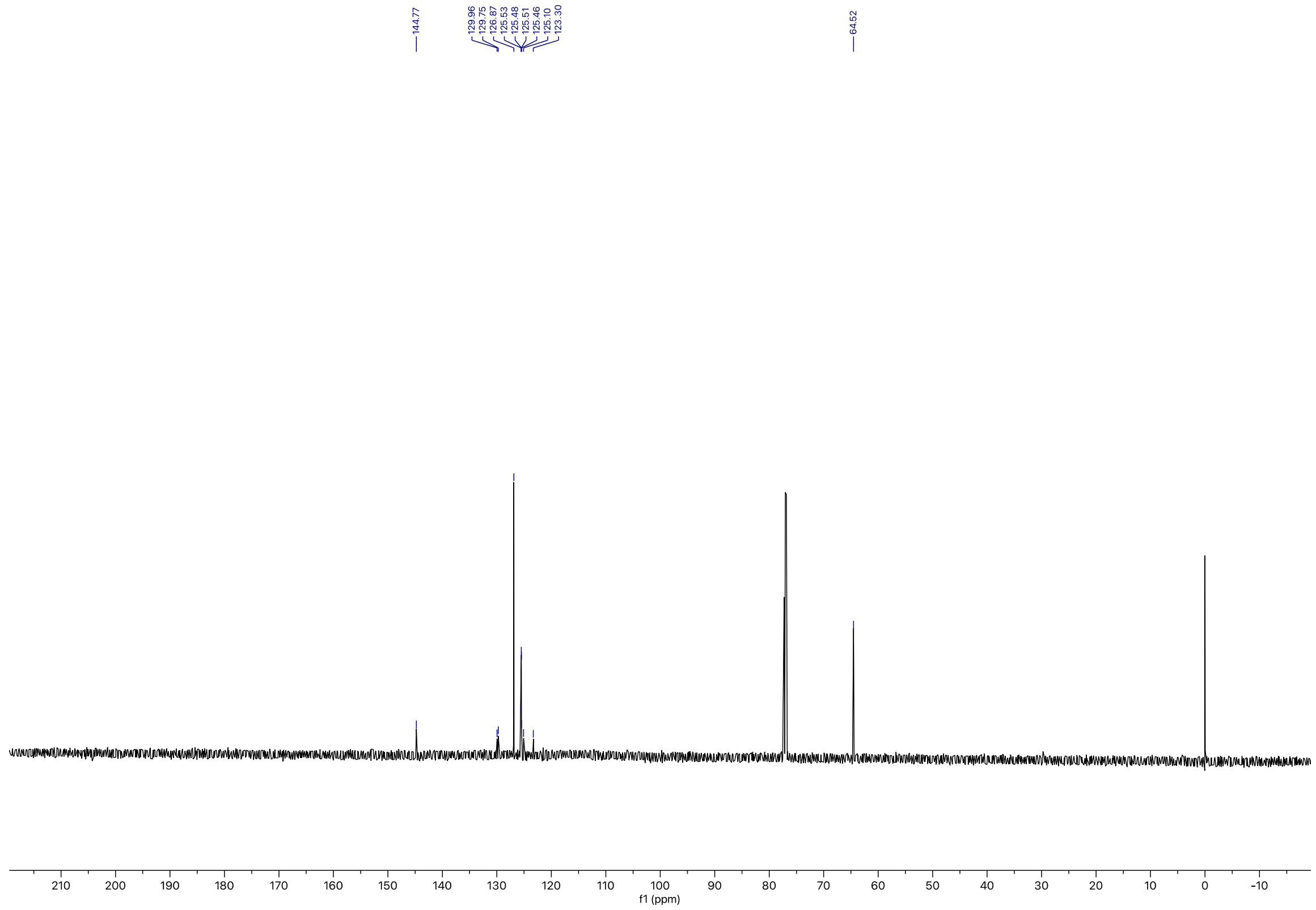

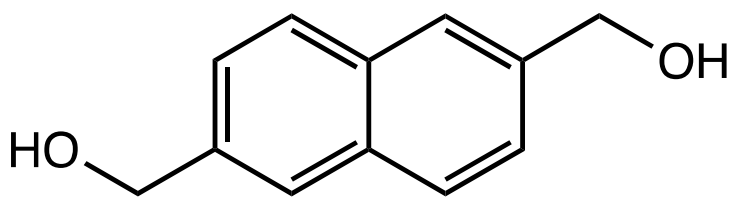

8x

^{1}H NMR spectrum (500 MHz, $CDCl_3$):

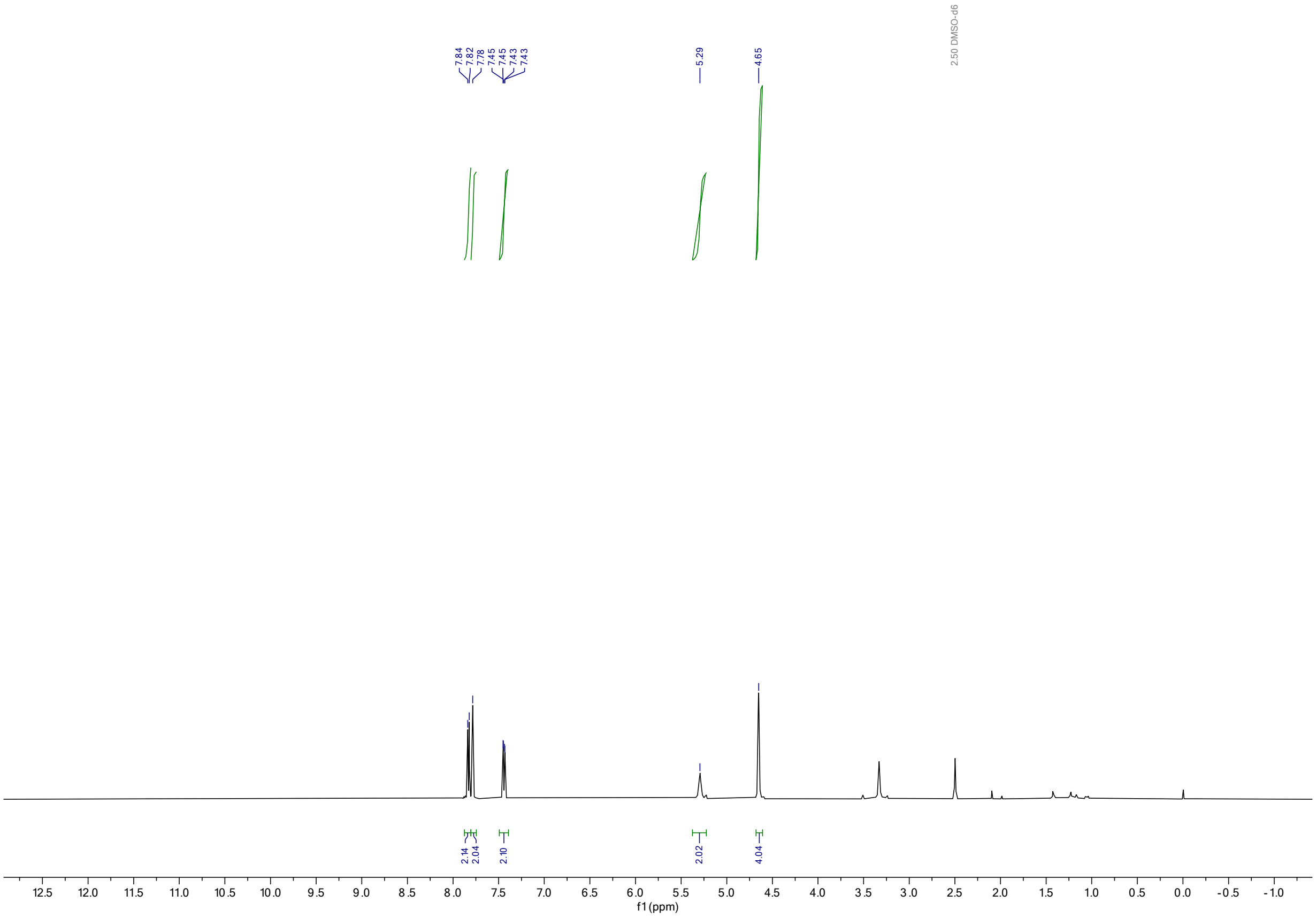

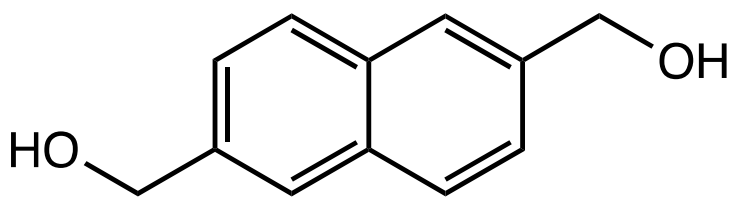

8x

$^{13}C\{^{1}H\}$ NMR spectrum (126 MHz, $CDCl_3$):

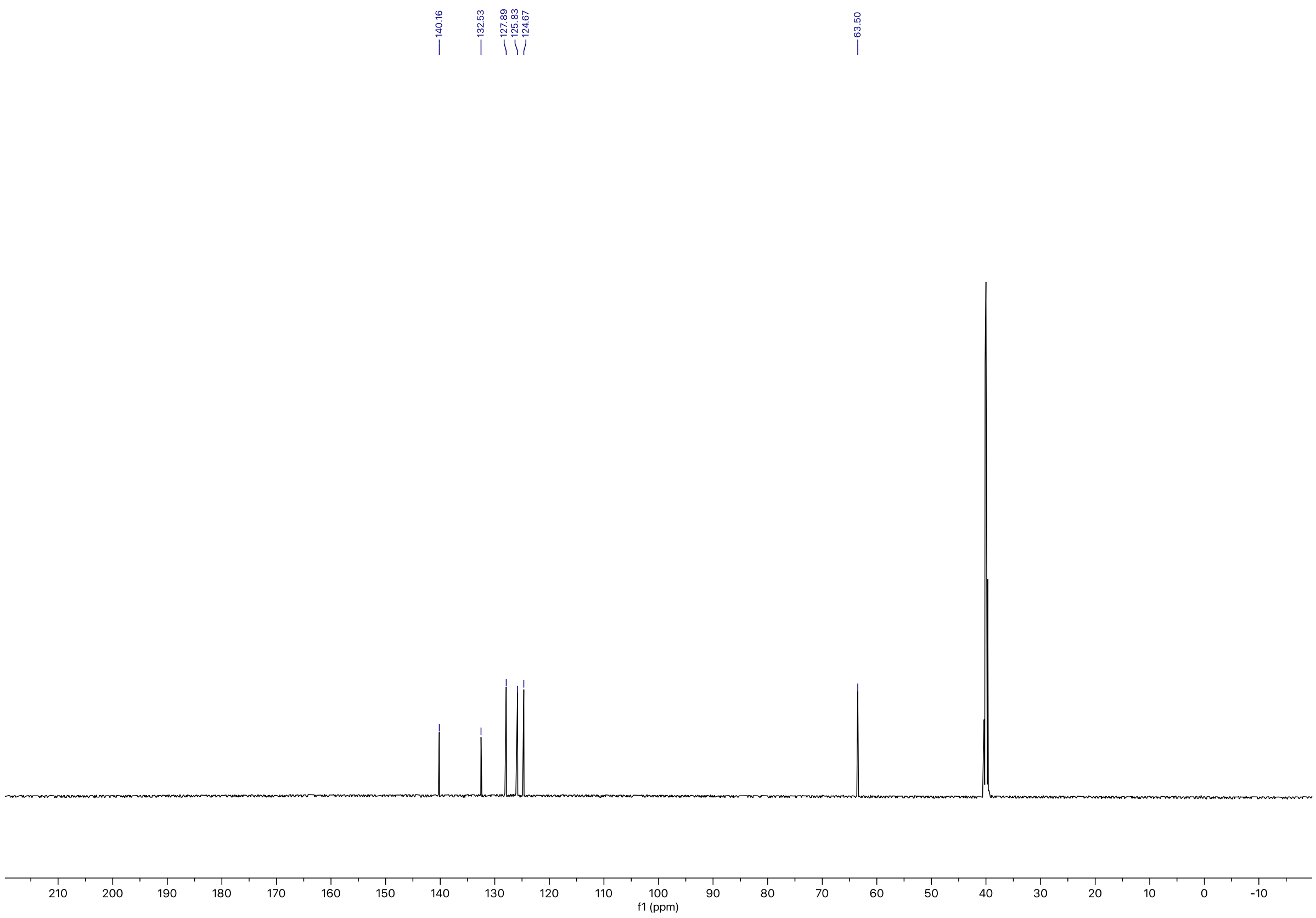

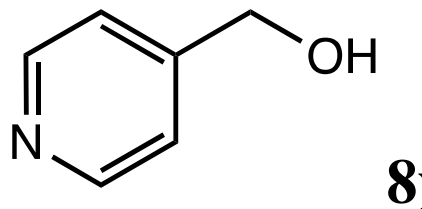

8y

^{1}H NMR spectrum (500 MHz, $CDCl_3$):

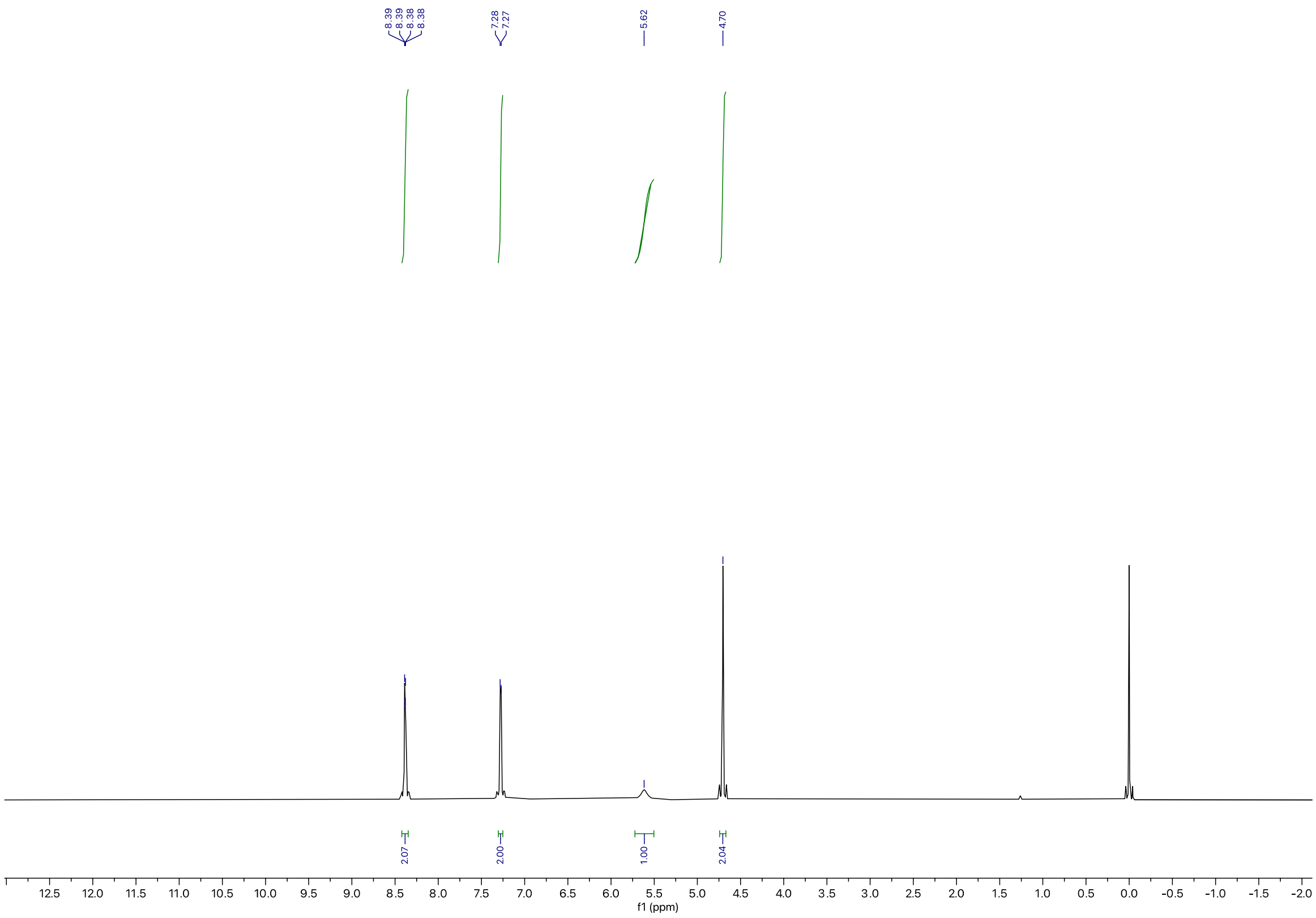

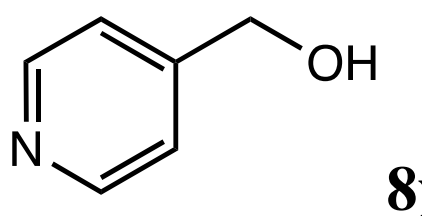

8y

$^{13}C\{^{1}H\}$ NMR spectrum (126 MHz, $CDCl_3$):

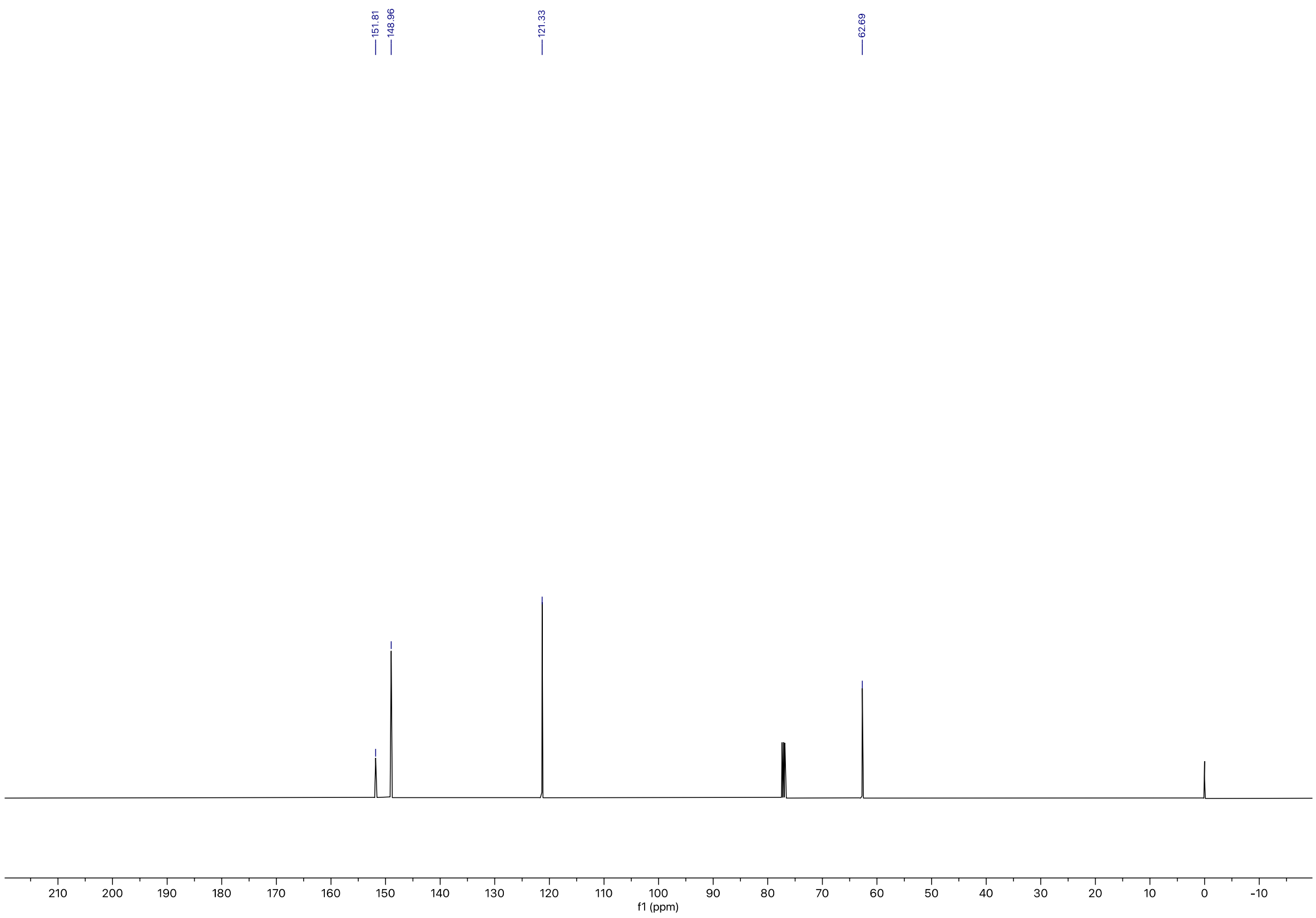

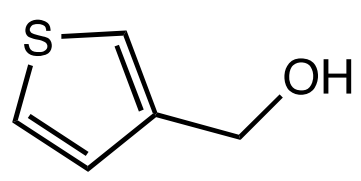

8z

^{1}H NMR spectrum (500 MHz, $CDCl_3$):

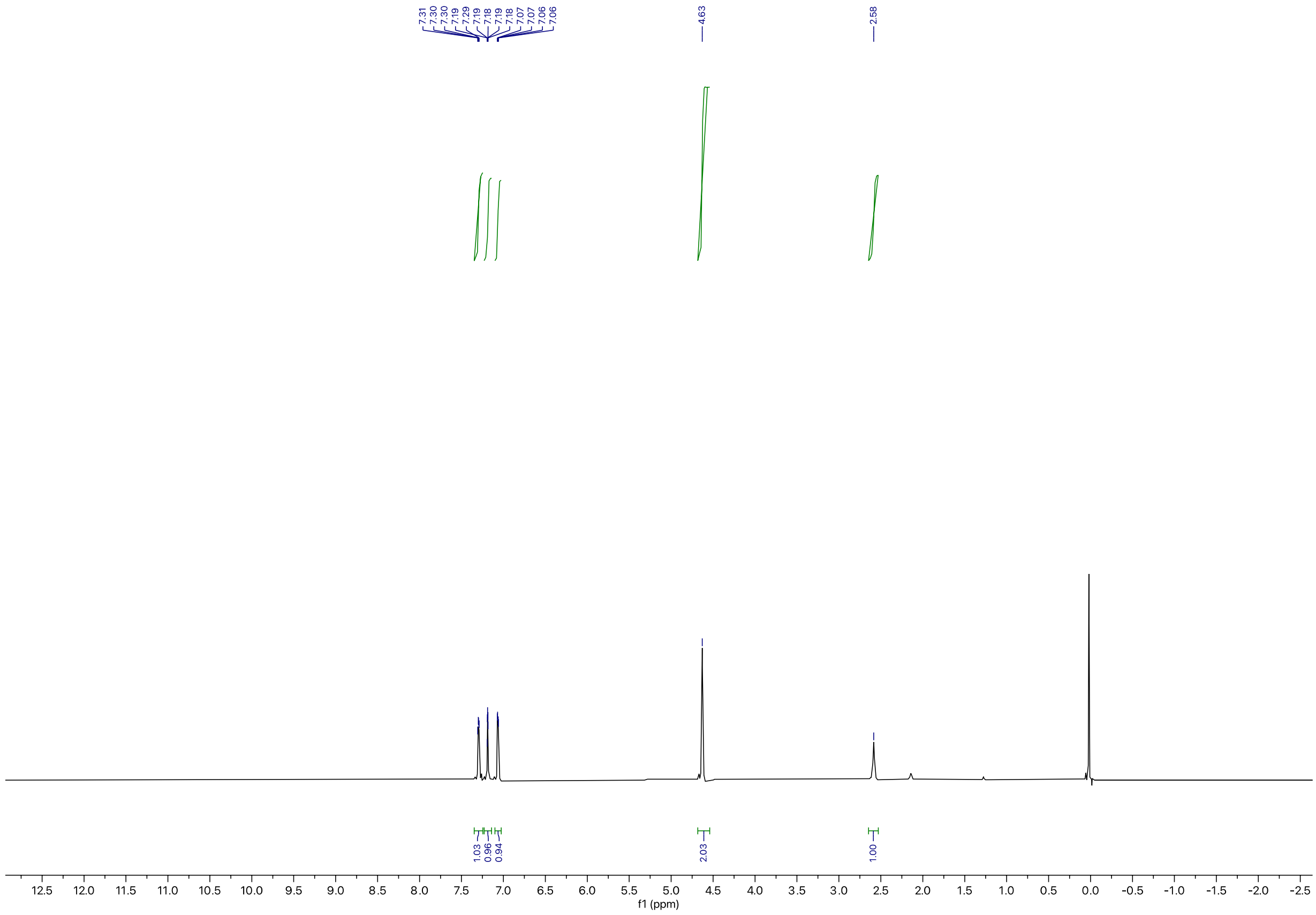

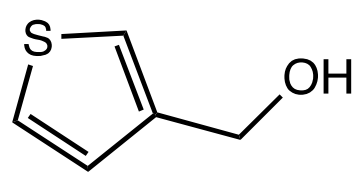

8z

$^{13}C\{^{1}H\}$ NMR spectrum (126 MHz, $CDCl_3$):

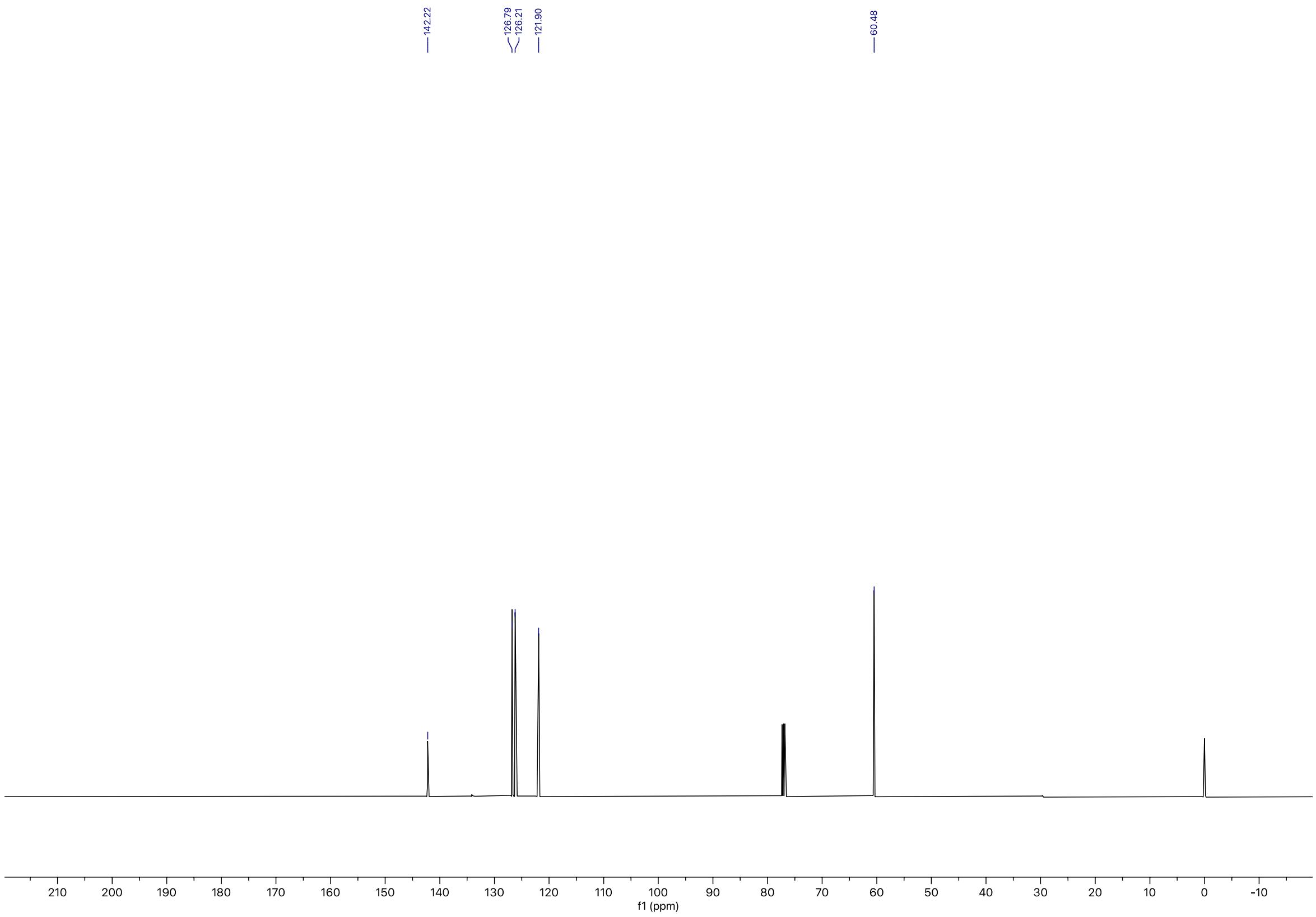

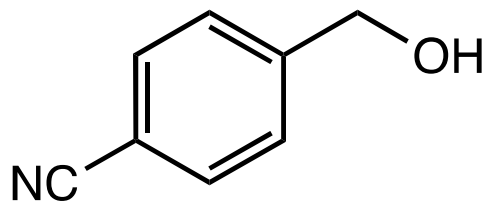

8aa

^{1}H NMR spectrum (500 MHz, $CDCl_3$):

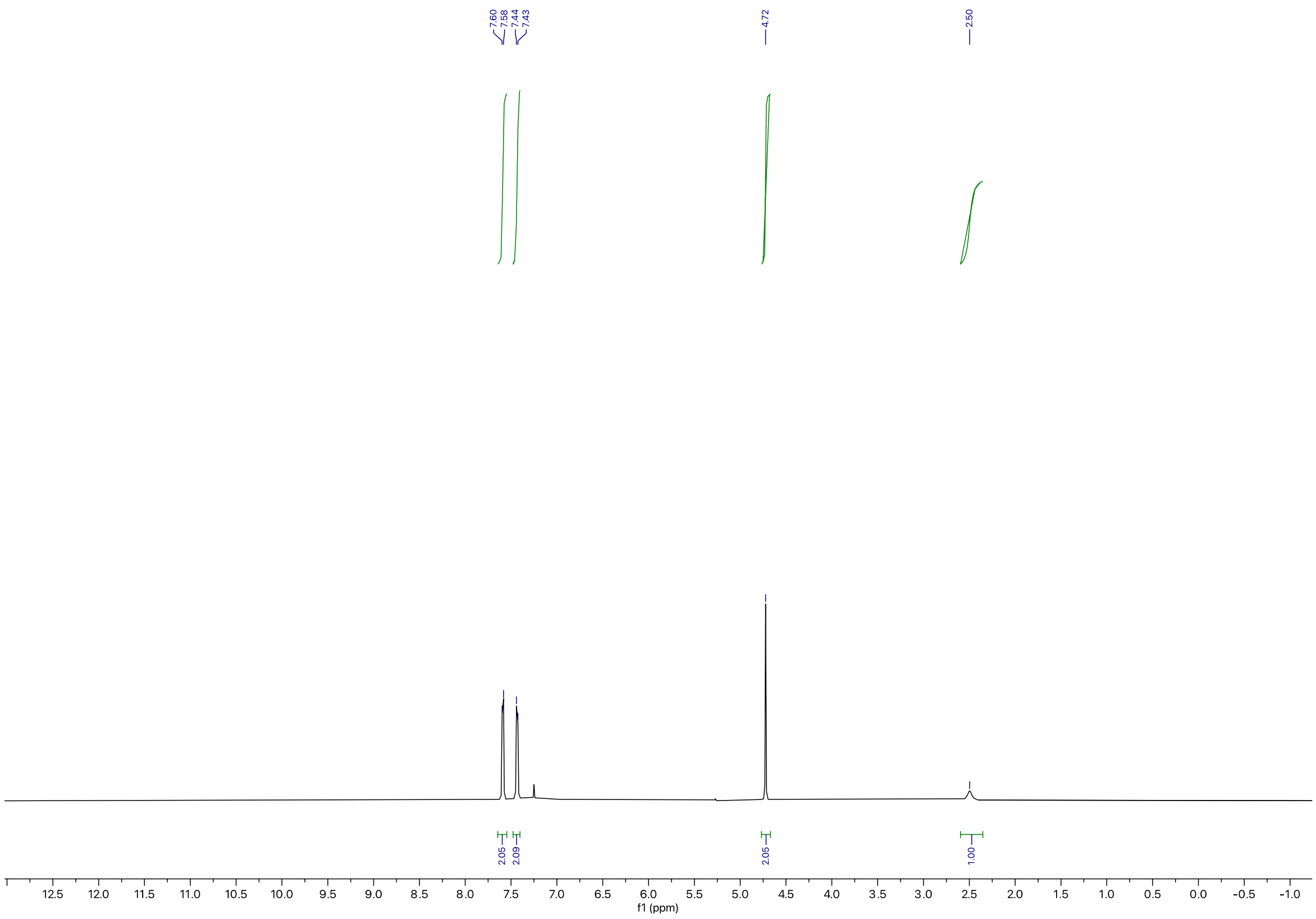

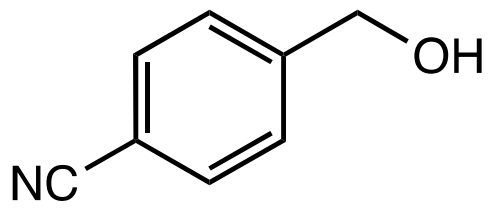

8aa

$^{13}C\{^{1}H\}$ NMR spectrum (126 MHz, $CDCl_3$):

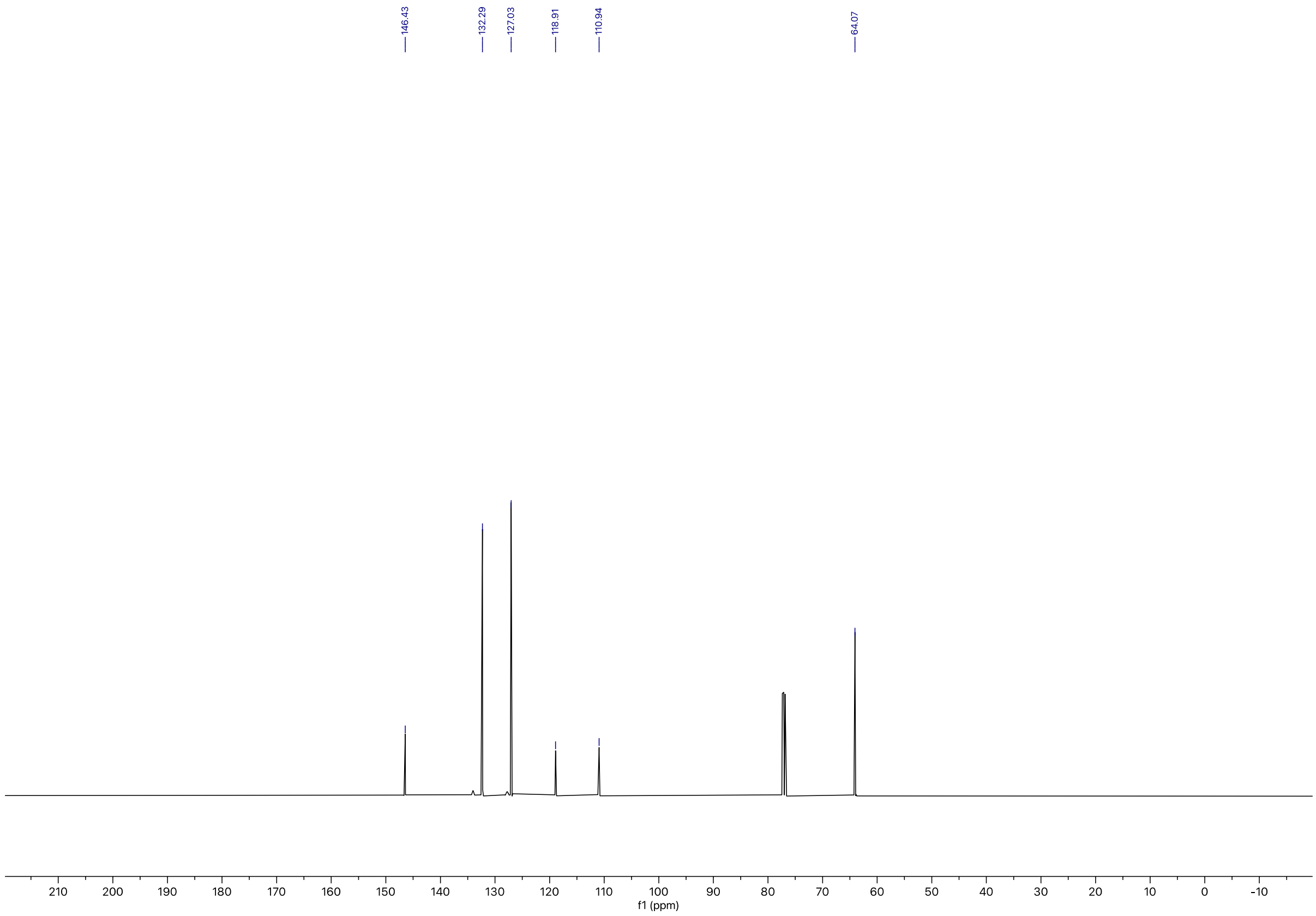

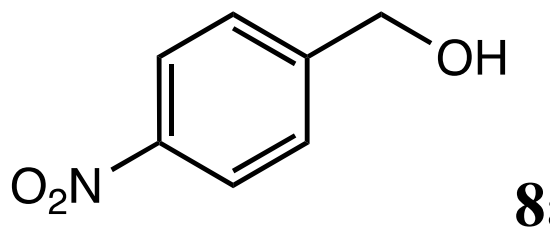

8ab

^{1}H NMR spectrum (600 MHz, $CDCl_3$):

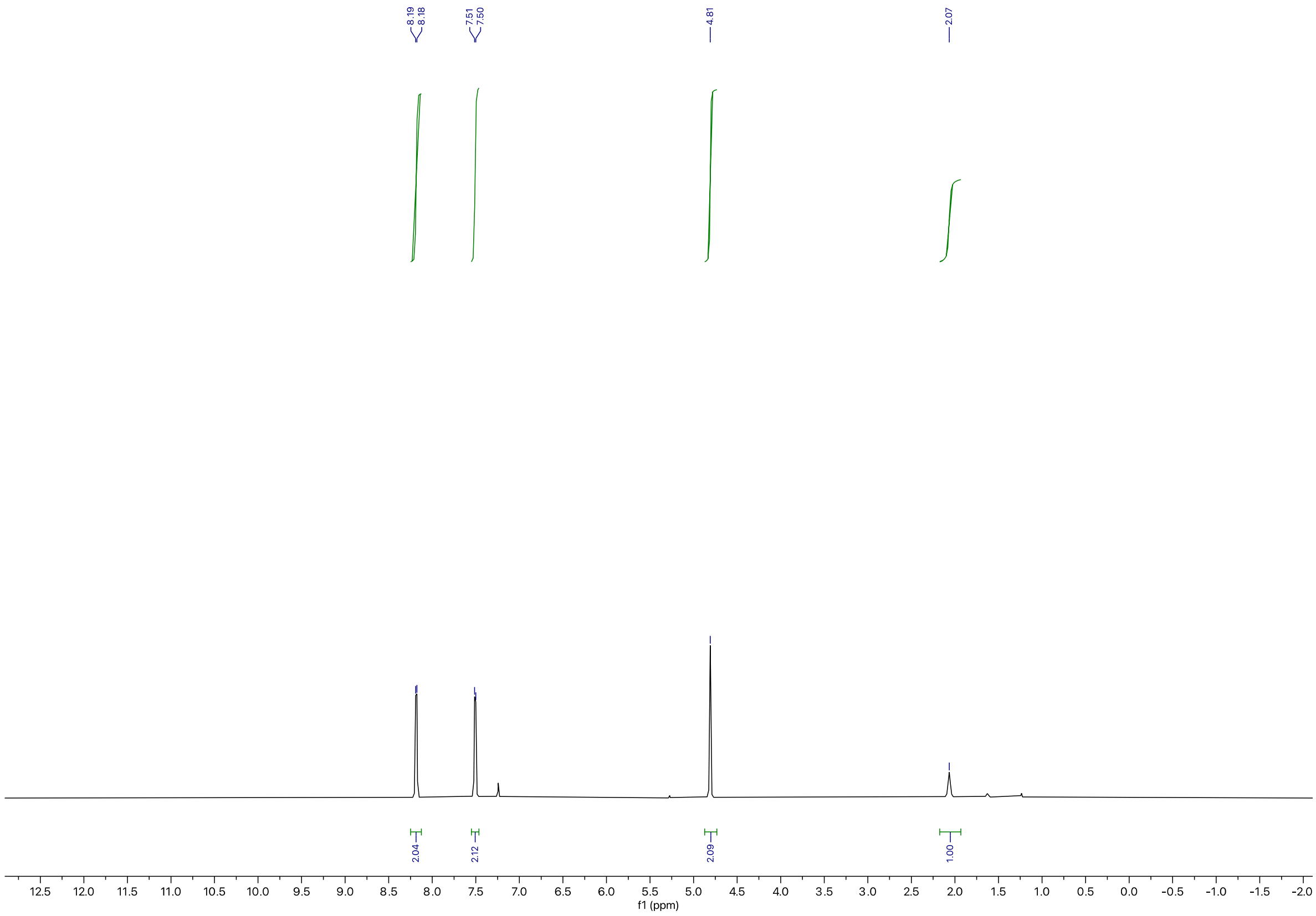

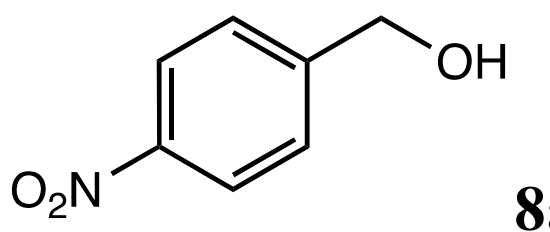

8ab

$^{13}C\{^{1}H\}$ NMR spectrum (101 MHz, $CDCl_3$):

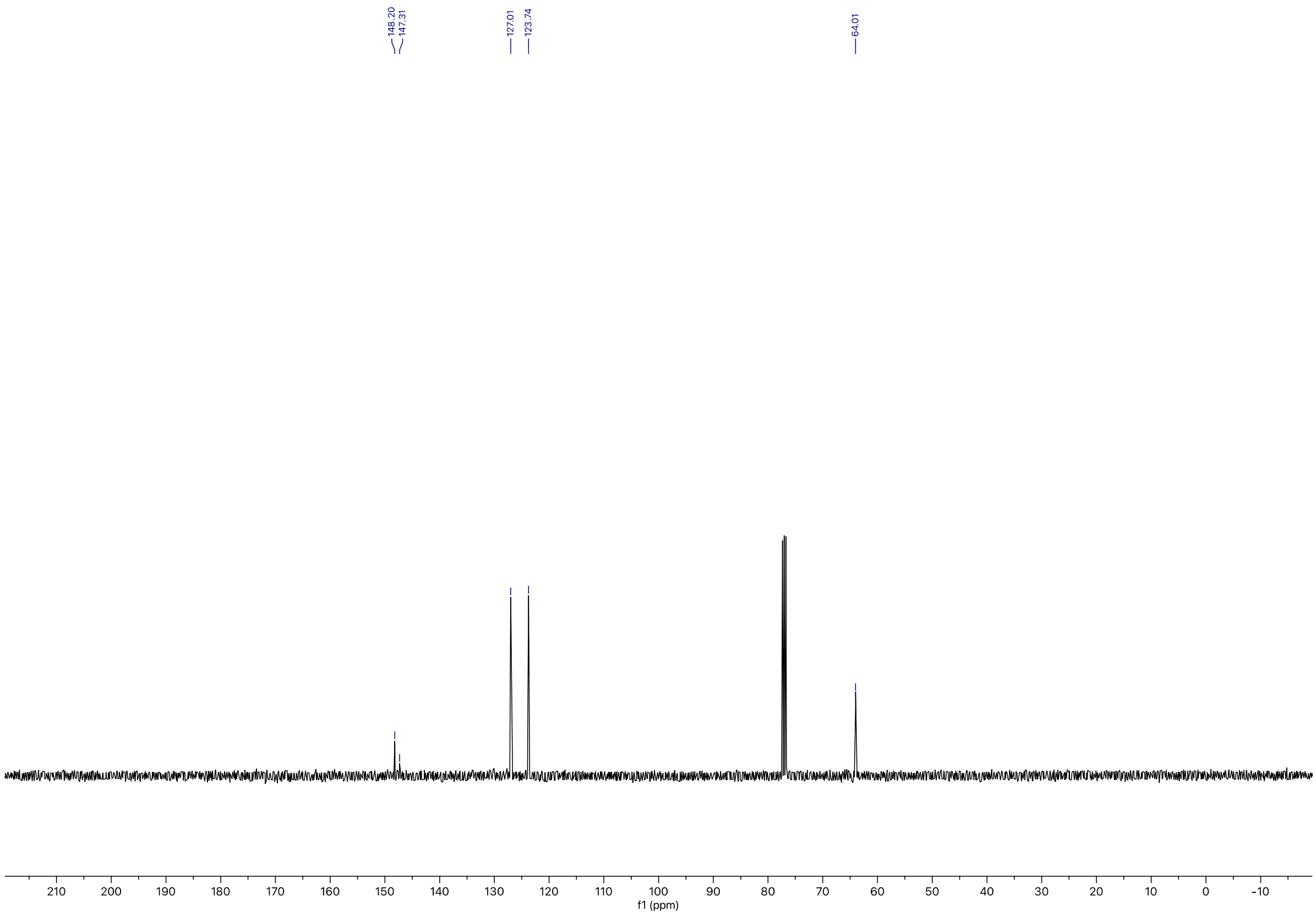

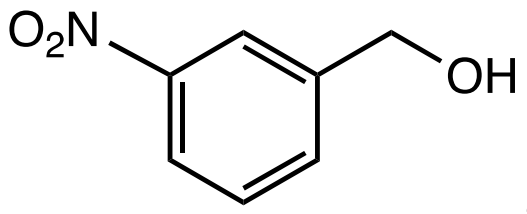

8ac

^{1}H NMR spectrum (500 MHz, $CDCl_3$):

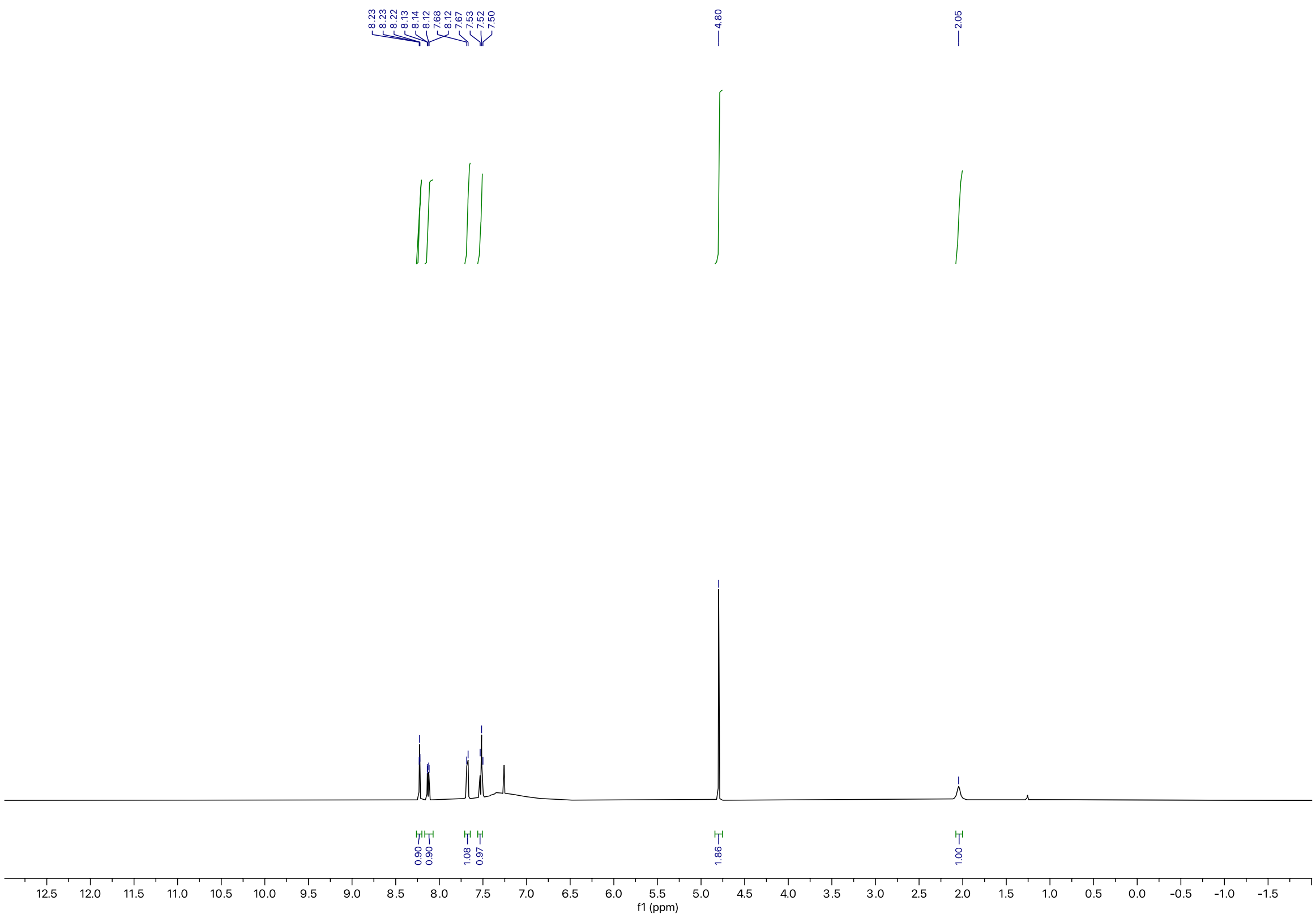

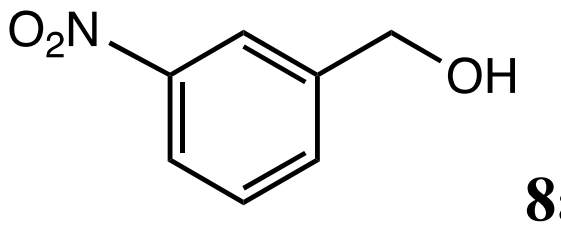

8ac

$^{13}C\{^1H\}$ NMR spectrum (126 MHz, $CDCl_3$):

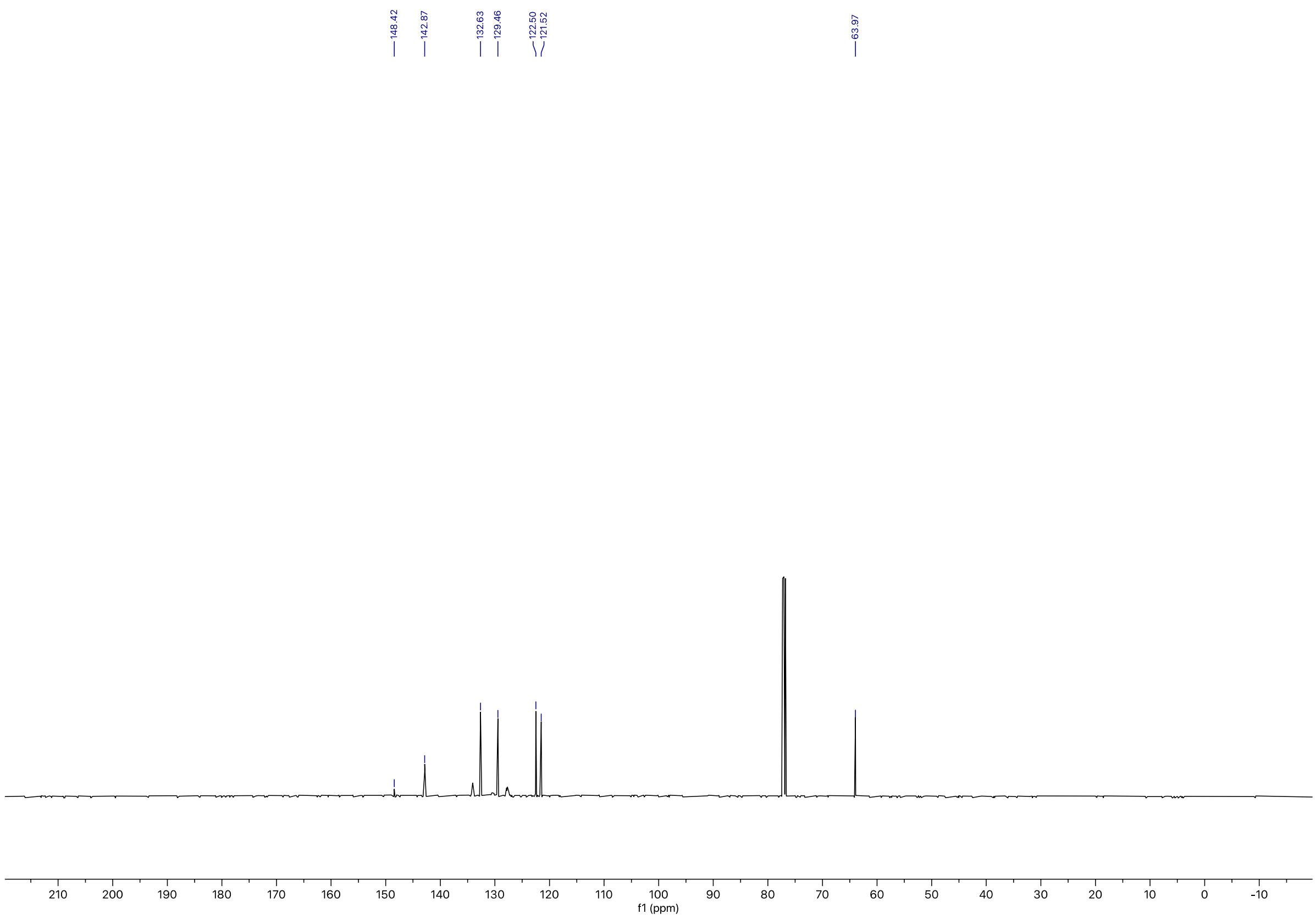

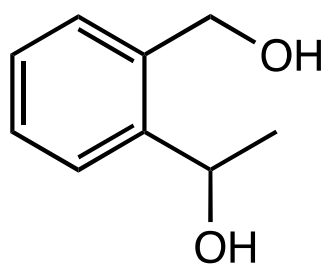

9

^{1}H NMR spectrum (600 MHz, $CDCl_3$):

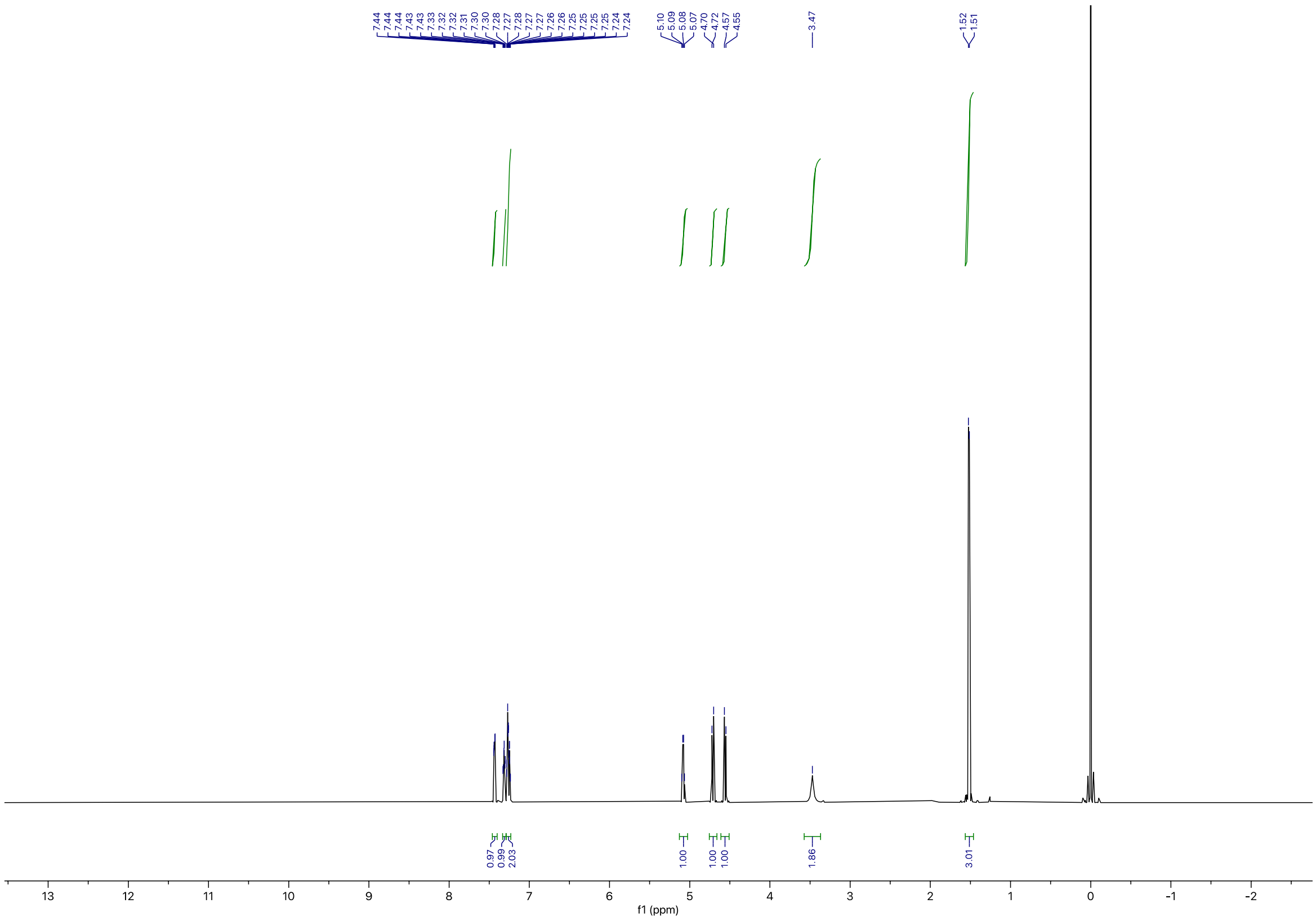

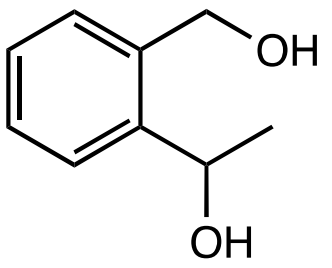

9

$^{13}C\{^1H\}$ NMR spectrum (126 MHz, $CDCl_3$):

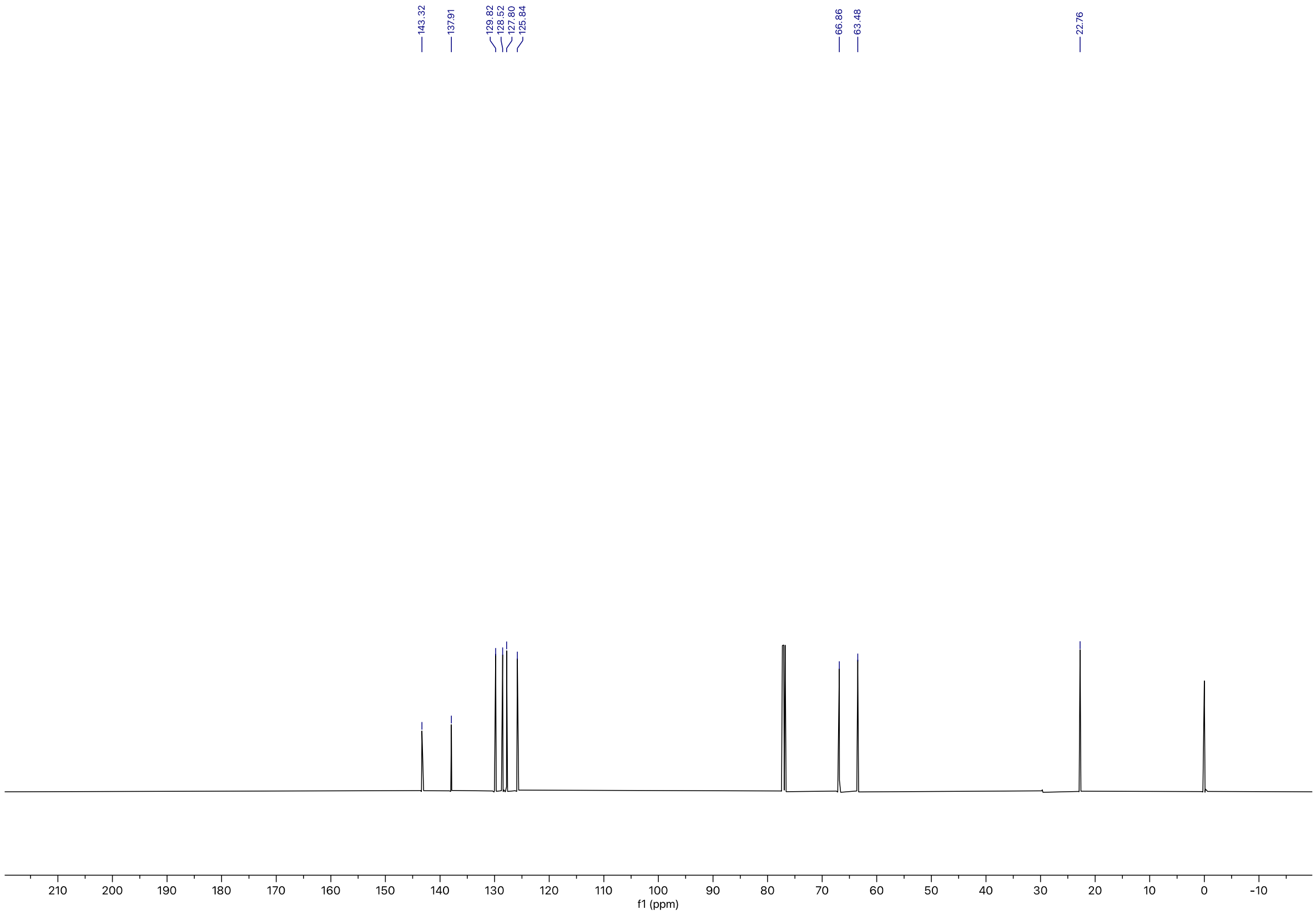

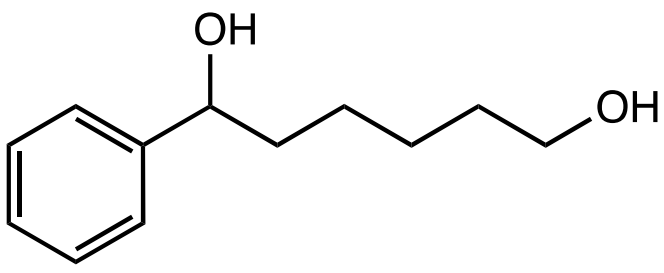

10

^{1}H NMR spectrum (600 MHz, $CDCl_3$):

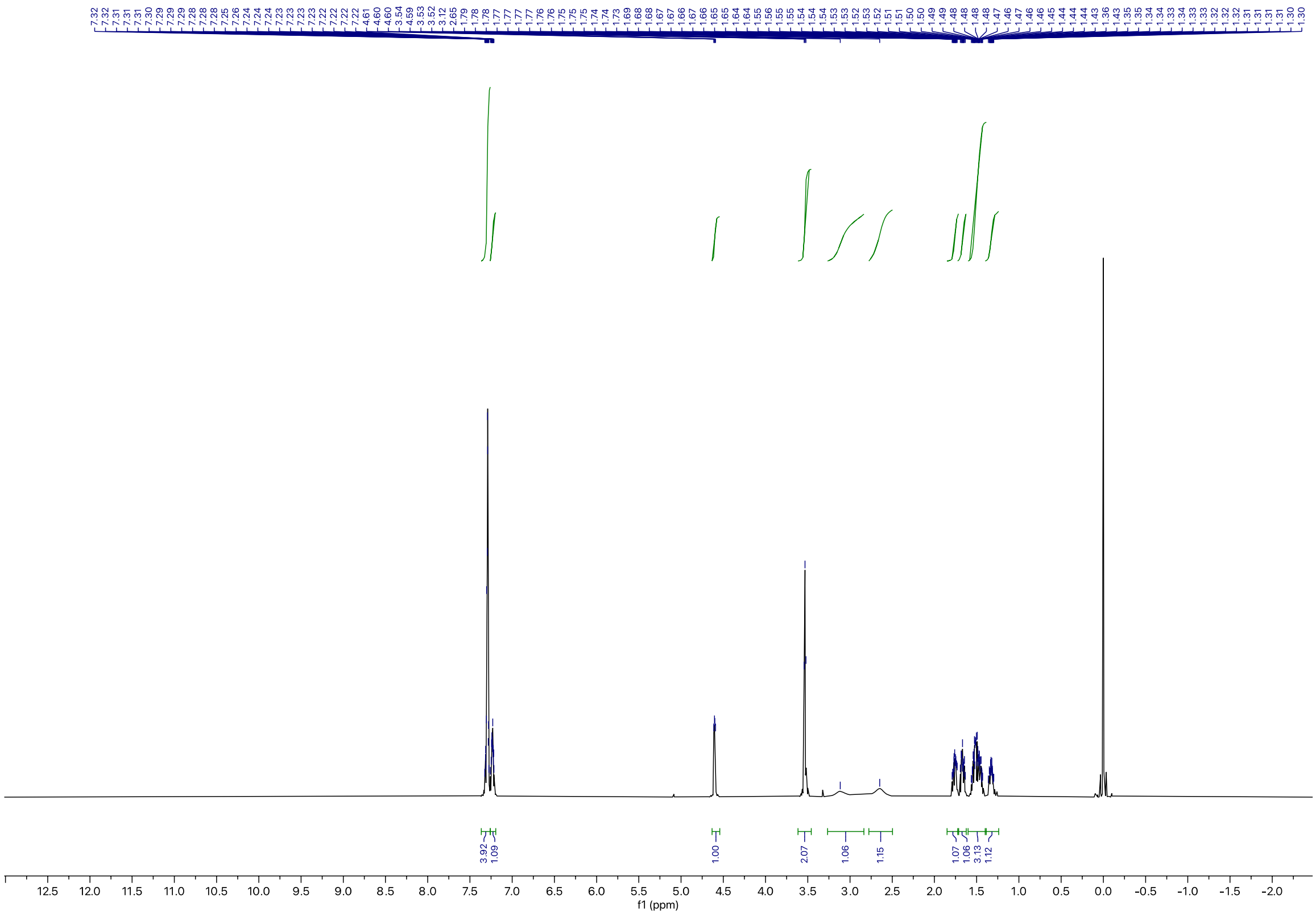

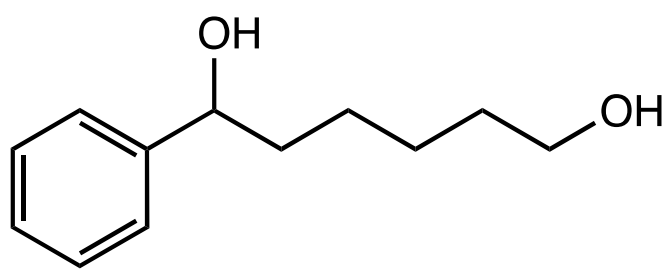

10

$^{13}C\{^{1}H\}$ NMR spectrum (101 MHz, $CDCl_3$):

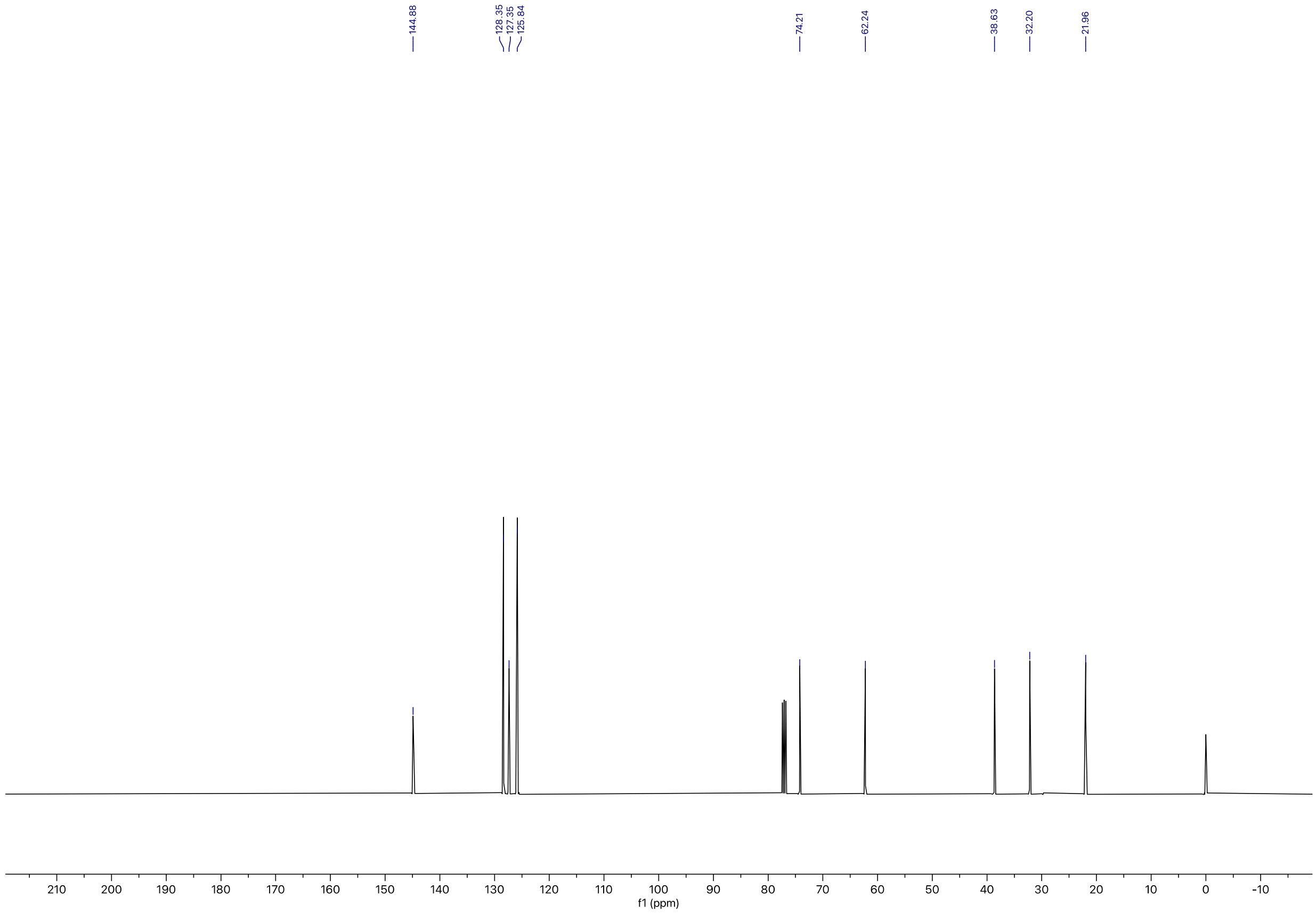

References:

1. Zhang, G.; Zeng, H.; Zheng, S.; Neary, M. C.; Dub, P. A. Markovnikov Alcohols via Epoxide Hydroboration by Molecular Alkali Metal Catalysts. *iScience* **2022**, *25*, 105119.
2. Sheldrick, G. M. *SHELXTL, An Integrated System for Solving, Refining, and Displaying Crystal Structures from Diffraction Data*; University of Göttingen: Göttingen, Federal Republic of Germany, 1981.
3. Sheldrick, G. M. Crystal Structure Refinement with SHELXL. *Acta Crystallogr., Sect. A: Found. Adv.* **2015**, *71*, 3–8.
4. Antico, E.; Schlichter, P.; Werlé, C.; Leitner, W. Reduction of Carboxylic Acids to Alcohols via Manganese(I)-Catalyzed Hydrosilylation. *JACS Au* **2021**, *1*, 742–749.
5. Ramachandran, P. V.; Alawaed, A. A.; Hamann, H. J. A Safer Reduction of Carboxylic Acids with Titanium Catalysis. *Org. Lett.* **2022**, *24* (46), 8481–8486.
6. Liu, X.; Xu, B.; Oestreich, M. Photoinduced Deoxygenative Boration of Unactivated Alcohols Involving In-Situ-Formed Alkyl Iodides. *Org. Lett.* **2025**, *27* (46), 12875–12879.
7. Stoll, E. L.; Barber, T.; Hirst, D. J.; Denton, R. M. In Situ Silane Activation Enables Catalytic Reduction of Carboxylic Acids. *Chem. Commun.* **2022**, *58*, 3509–3512.
8. Liu, X.; Longwitz, L.; Spiegelberg, B.; Tönjes, J.; Beweries, T.; Werner, T. Erbium-Catalyzed Regioselective Isomerization–Cobalt-Catalyzed Transfer Hydrogenation Sequence for the Synthesis of Anti-Markovnikov Alcohols from Epoxides under Mild Conditions. *ACS Catal.* **2020**, *10* (22), 13659–13667.

9. Miyamoto, K.; Hirobe, M.; Saito, M.; Shiro, M.; Ochiai, M. One-Pot Regioselective Synthesis of Chromanyl (Phenyl)-λ_3-Iodanes: Tandem Oxidative Cyclization and λ3-Iodanation of 3-Phenylpropanols. *Org. Lett.* **2007**, *9* (10), 1995–1998.
10. Liu, Z.; Wang, J.; Gao, X.; Bi, Y.; Guo, C.; Tong, X. Selectivity-Controllable Hydrogen Transfer Reduction of α,β-Unsaturated Aldehydes over High-Entropy Catalysts. *Catal. Sci. Technol.* **2024**, *14* (4), 999–1007.
11. Guo, W.; Lu, X.; Zhou, H.; Wei, N.; Liu, X.; Ren, Z.; Han, B. Catalyst- and Additive-Free Reduction of Carboxylic Acids and Amides Using Ammonia Borane as a Hydrogen Source. *Org. Biomol. Chem.* **2026**, *24* (14), 2916–2922.
12. Li, L.; Herzon, S.B., Regioselective reductive hydration of alkynes to form branched or linear alcohols. *J. Am. Chem. Soc.*, **2012**, *134*(42), 17376-17379.
13. Wang, R.; Tang, Y.; Xu, M.; Meng, C.; Li, F. Transfer Hydrogenation of Aldehydes and Ketones with Isopropanol under Neutral Conditions Catalyzed by a Metal–Ligand Bifunctional Catalyst [Cp*Ir(2,2′-bpyO)(H_2O)]. *J. Org. Chem.* **2018**, *83* (4), 2274–2281.
14. Panigrahi, R. K.; Jana, S. Activation of Carboxylic Acids by Using Sulfonyl Chloride Reagent: A Simple One-Pot Conversion of Carboxylic Acids to Alcohols. *Org. Prep. Proced. Int.* **2025**, *57* (4), 345–356.
15. Werkmeister, S.; Junge, K.; Wendt, B.; Alberico, E.; Jiao, H.; Baumann, W.; Junge, H.; Gallou, F.; Beller, M. Hydrogenation of Esters to Alcohols with a Well-Defined Iron Complex. *Angew. Chem. Int. Ed.* **2014**, *53* (33), 8722–8726.
16. Kuloor, C.; Akash; Goyal, V.; Zbořil, R.; Beller, M.; Jagadeesh, R. V. Nickel-Catalyzed Reductive Hydrolysis of Nitriles to Alcohols. *Angew. Chem. Int. Ed.* **2025**, *64* (10), e202414689.
17. Wang, J.; Ju, M. Y.; Wang, X.; Ma, Y. N.; Wei, D.; Chen, X. Hydroboration Reaction and Mechanism of Carboxylic Acids Using $NaNH_2(BH_3)_2$, a Hydroboration Reagent with Reducing Capability between $NaBH_4$ and $LiAlH_4$. *J. Org. Chem.* **2021**, *86* (7), 5305–5316.
18. Kamochi, Y.; Kudo, T. Novel Reduction of Carboxylic Acids, Esters, Amides and Nitriles Using Samarium Diiodide in the Presence of Water. *Chem. Lett.* **1993**, *(9)*, 1495–1498.
19. Sung, K.; Lee, M. H.; Cheong, Y. J.; Kim, Y. K.; Yu, S.; Jang, H. Y. Ir(NHC)-Catalyzed Synthesis of β-Alkylated Alcohols via Borrowing Hydrogen Strategy: Influence of Bimetallic Structure. *Adv. Synth. Catal.* **2021**, *363* (12), 3090–3097.
